# α-Perfluoroalkyl-β-alkynylation of alkenes *via* radical alkynyl migration†
†Electronic supplementary information (ESI) available. See DOI: 10.1039/c7sc02175e



**DOI:** 10.1039/c7sc02175e

**Published:** 2017-08-04

**Authors:** Xinjun Tang, Armido Studer

**Affiliations:** a Organisch-Chemisches Institut , Westfälische Wilhelms-Universität , Corrensstraβe 40 , 48149 Münster , Germany . Email: studer@uni-muenster.de

## Abstract

Transition metal-free radical α-perfluoroalkylation with concomitant β-alkynylation of unactivated alkenes is presented. These cascades proceed *via* electron-catalysis and comprise a radical 1,4- or 1,5-alkynyl migration from tertiary propargylic alcoholates to secondary or tertiary C-radicals as the key step. Alkynyl migration leads to a ketyl radical anion that sustains the chain as a single electron transfer reducing reagent.

## Abstract 2

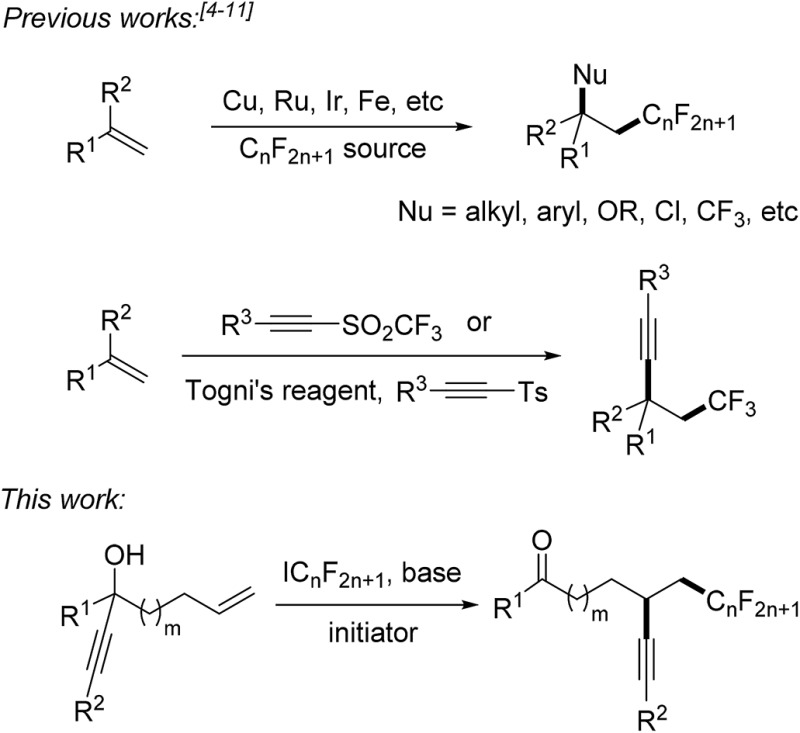
Transition metal-free radical α-perfluoroalkylation with concomitant β-alkynylation of unactivated alkenes is presented.

## Introduction

**Scheme 1:**
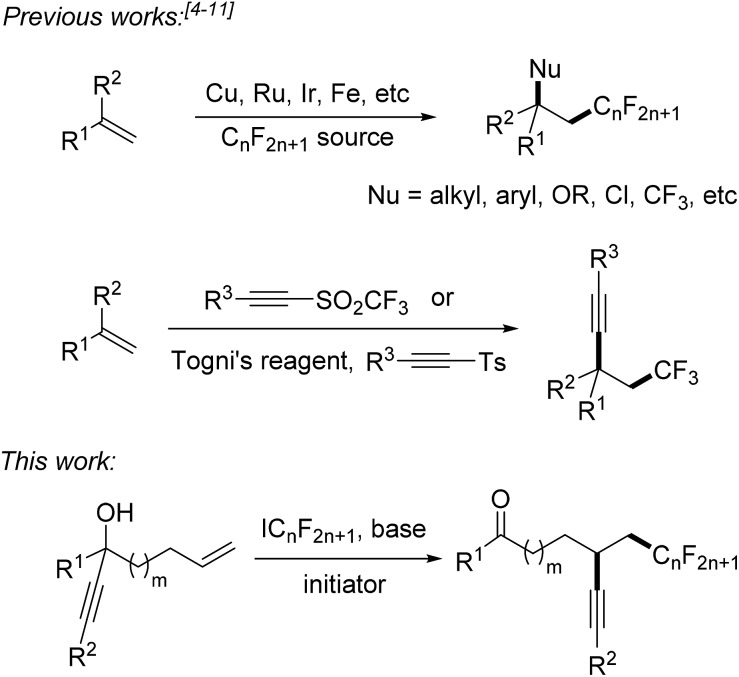
Vicinal alkene difunctionalization comprising a perfluoroalkylation.

Fluorinated compounds are highly abundant in agrochemical industry, medicinal chemistry and materials science.^1^ The introduction of fluorine atoms and of perfluoroalkyl groups into organic compounds increases their solubility, lipophilicity, bioavailability and metabolic stability.^2,3^ Therefore, it is important to develop novel synthetic methods for fluorination or perfluoroalkylation. Significant progress has been made on alkene perfluoroalkylation with concomitant β-functionalization in recent years.^4^ In most cases, transition-metal catalysts such as Cu(i),^5^ Ru(ii),^6^ Ir(iii),^7^ and Fe(ii)^8^ are required to run these reactions and perfluoroalkylated products bearing alkyl, aryl, OR, Cl or CF_3_ groups at the β-position can be obtained (Scheme 1). However, alkene perfluoroalkylation with accompanying β-alkynylation is rare and only four papers were published along these lines. Guided by pioneering studies of the Fuchs group,^9^ Li^10^ and Yu^11^ recently reported radical alkene trifluoromethylation and subsequent intermolecular alkynylation using phenylethynyl *p*-tolyl sulfone and acetylenic triflone as the alkynylating reagents. As drawbacks of these processes, alkynyl transfer is limited to phenylalkynylation and trimethylsilylalkynylation and the methods can only be used for trifluoromethylation. During preparation of this manuscript, Zhu and co-workers reported an elegant photoredox catalyzed alkene α-trifluoromethylation-β-alkynylation comprising an intramolecular alkynyl migration.^12^ However, the scope of this process is limited to the trifluoromethylation and the costly Umemoto reagent has to be applied along with a photoredox catalyst. Thus, novel and efficient general methods for alkene α-perfluoroalkylation-β-alkynylation are highly demanded. Considering the economy, transition metal free processes are highly valuable.

Herein we disclose a simple and efficient novel method for the α-perfluoroalkylation with concomitant β-alkynylation of unactivated alkenes proceeding *via* radical 1,4- or 1,5-alkynyl migration using perfluoroalkyl iodides^13,14^ as cheap and commercially available perfluoroalkyl radical sources in combination with readily prepared tertiary propargylic alcohols as substrates (Scheme 1). Notably, the potential of radical alkene perfluoroalkylation with concomitant β-functionalization *via* intramolecular formyl,^15*a*^ aryl,^15*b*^ and heteroaryl^4*e*^ migration has recently been documented.^15c,d^

## Results and discussion

Initial investigations were performed with 3-methyl-1-phenylhept-6-en-1-yn-3-ol **1a** and perfluorobutyl iodide using Li-hexamethyldisilazide (LiHMDS) (1.2 equiv.) as a base in DME. The alcohol was first deprotonated with LiHMDS at room temperature (0.5 h). After complete deprotonation, DABCO (1.5 equiv., to mediate chain initiation)^14^ and perfluorobutyl iodide **2a** (1.8 equiv.) were added sequentially and the mixture was stirred under visible-light irradiation (using a Philips Master HPI-T Plus (400 W) bulb) at 50 °C for 18 hours. To our delight, the target ketone **3a** was obtained in 53% yield (Table 1, entry 1). The base heavily affects the reaction outcome and the yield dropped to 42% upon using LiOH (Table 1, entry 2) but a better result was achieved with NaOH (63%) (Table 1, entry 3). Surprisingly, perfluoroalkylation alkynyl migration failed using KOH as a base (Table 1, entry 4).

**Table 1:** Reaction optimization^*a*^

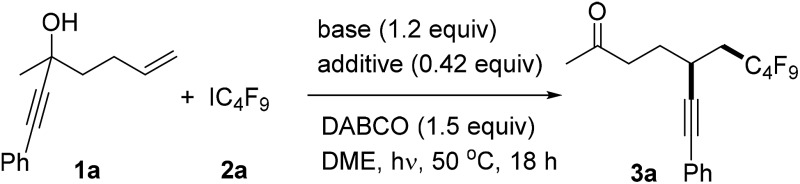				
Entry	Base	Additive	Solvent	Yield of **3a**^*b*^ [%]
1	LiHMDS	—	DME	53
2	LiOH	—	DME	42
3	NaOH	—	DME	63
4	KOH	—	DME	—
5	LiHMDS	NaOH	DME	58
**6**	**LiHMDS**	**LiOH**	**DME**	**79 (79)** ^*c*^
7	LiHMDS	KOH	DME	6
8	LiHMDS	LiOH	THF	45
9	LiHMDS	LiOH	1,4-Dioxane	28
10^*d*^	LiHMDS	LiOH	DME	39
11^*e*^	LiHMDS	LiOH	DME	8
12^*f*^	LiHMDS	LiOH	DME	27
13^*g*^	LiHMDS	LiOH	DME	—
14^*h*^	—	—	DME	6

^*a*^The reaction was conducted with **1a** (0.1 mmol), **2a** (1.8 equiv.), DABCO (1.5 equiv.), base (1.2 equiv.), and additive (0.42 equiv.) in 1.25 mL of DME under visible-light irradiation (using a Philips Master HPI-T Plus (400 W) bulb) at 50 °C for 18 h.

^*b*^Determined by ^1^H NMR analysis using 1-fluoro-4-methylbenzene as the internal standard.

^*c*^Isolated yield in parenthesis.

^*d*^Bn_2_NH was used instead of DABCO.

^*e*^Et_3_N was used instead of DABCO.

^*f*^The reaction was conducted without DABCO.

^*g*^The reaction was conducted without visible-light irradiation.

^*h*^No base and additive were used.

Having noted the importance of base, we started to screen mixtures of two bases. The addition of NaOH (0.42 equiv.) to LiHMDS did not affect the reaction outcome to a large extent (Table 1, entry 5) but with additional LiOH (0.42 equiv.) the yield significantly improved (79%) (Table 1, entry 6). However, with KOH as an additional base the yield dropped to 6% (Table 1, entry 7). The reaction proceeded less efficiently using THF and 1,4-dioxane as solvents (Table 1, entries 8 and 9). Replacing DABCO with Bn_2_NH or Et_3_N, or without DABCO, lower yields were obtained (Table 1, entries 10–12) and the cascade did not work without any visible-light irradiation (Table 1, entry 13). Base was found to be important and the target **3a** was formed in only 6% in the absence of any base (Table 1, entry 14). Thus, **1a** (0.1 mmol), **2a** (1.8 equiv.), LiHMDS (1.2 equiv.), LiOH (0.42 equiv.), and DABCO (1.5 equiv.) in 1.25 mL of DME with stirring under visible-light irradiation (using a Philips Master HPI-T Plus (400 W) bulb) at 50 °C for 18 h were identified as the optimal reaction conditions for this sequence.

With the optimized reaction conditions in hand, we then turned to examine the scope with respect to the alkene component, keeping perfluorobutyl iodide **2a** as the C-radical precursor (Table 2). The aryl group in these propargylic alcohols was first varied by replacing the phenyl substituent in **1a** with differently substituted aryl groups. Electronic effects at the *para*-position do not play an important role as good results were achieved for both electron-rich and also electron-poor systems (**3b–f**, Table 2, entries 1–5). A similar result was noted for the *meta*-methyl substituted alcohol **1g** to provide **3g**, and also its *ortho*-congener **1g** gave the target ketone **3h** in a good yield (Table 2, entries 6 and 7). Thus, steric effects at the aryl moiety are not important, a fact which is further supported by the successful transformations of *ortho*, *ortho*′-disubstituted aryl alkynes **1i** and **1j** (see **3i**, **j**, Table 2, entries 8 and 9). Notably, the 1-naphthyl and 2-pyridyl groups are both tolerated as R-substituents, and the corresponding ketones **3k** and **3l** were obtained in good yields (Table 2, entries 10 and 11).

**Table 2:** Variation of the alkynyl substituent^*a*^

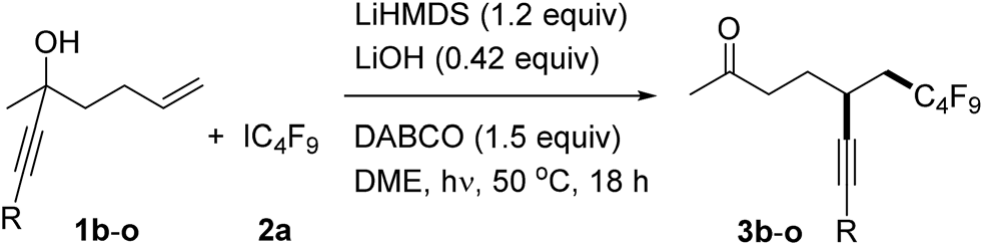			
Entry	R	Product	Yield [%]^*b*^
1	**1b**, 4-MeC_6_H_4_	**3b**	76
2	**1c**, 4-*^t^*BuC_6_H_4_	**3c**	56
3	**1d**, 4-MeOC_6_H_4_	**3d**	67
4	**1e**, 4-FC_6_H_4_	**3e**	68
5	**1f**, 4-ClC_6_H_4_	**3f**	83
6	**1g**, 3-MeC_6_H_4_	**3g**	68
7	**1h**, 2-MeC_6_H_4_	**3h**	78
8	**1i**, 2,4,6-Me_3_C_6_H_2_	**3i**	61
9	**1j**, 3,5-(MeO)_2_C_6_H_3_	**3j**	57
10	**1k**, 1-naphthyl	**3k**	71
11	**1l**, 2-pyridyl	**3l**	60
12	**1m**, 1-cyclohexenyl	**3m**	44
13	**1n**, ^*n*^C_4_H_9_	**3n**	40
14	**1o**, ^i^Pr_3_Si	**3o**	82

^*a*^The reaction was conducted with **1** (0.1 mmol), **2a** (1.8 equiv.), DABCO (1.5 equiv.), LiHMDS (1.2 equiv.), and LiOH (0.42 equiv.) in 1.25 mL of DME under visible-light irradiation (using a Philips Master HPI-T Plus (400 W) bulb) at 50 °C for 18 h.

^*b*^Isolated yield.

However, replacing the phenyl group in **1a** by a 1-cyclohexenyl (**1m**) or *n*-butyl substituent (**1n**) led to significantly reduced yields of the corresponding alkynylated ketones **3m** and **3n** (Table 2, entries 12 and 13). Likely the rate constant for the radical cyclization is smaller for these less activated alkynes. A good result was also achieved in the transformation of the silylated alkyne **1o** to provide **3o** in 82% isolated yield.

**Scheme 2:**
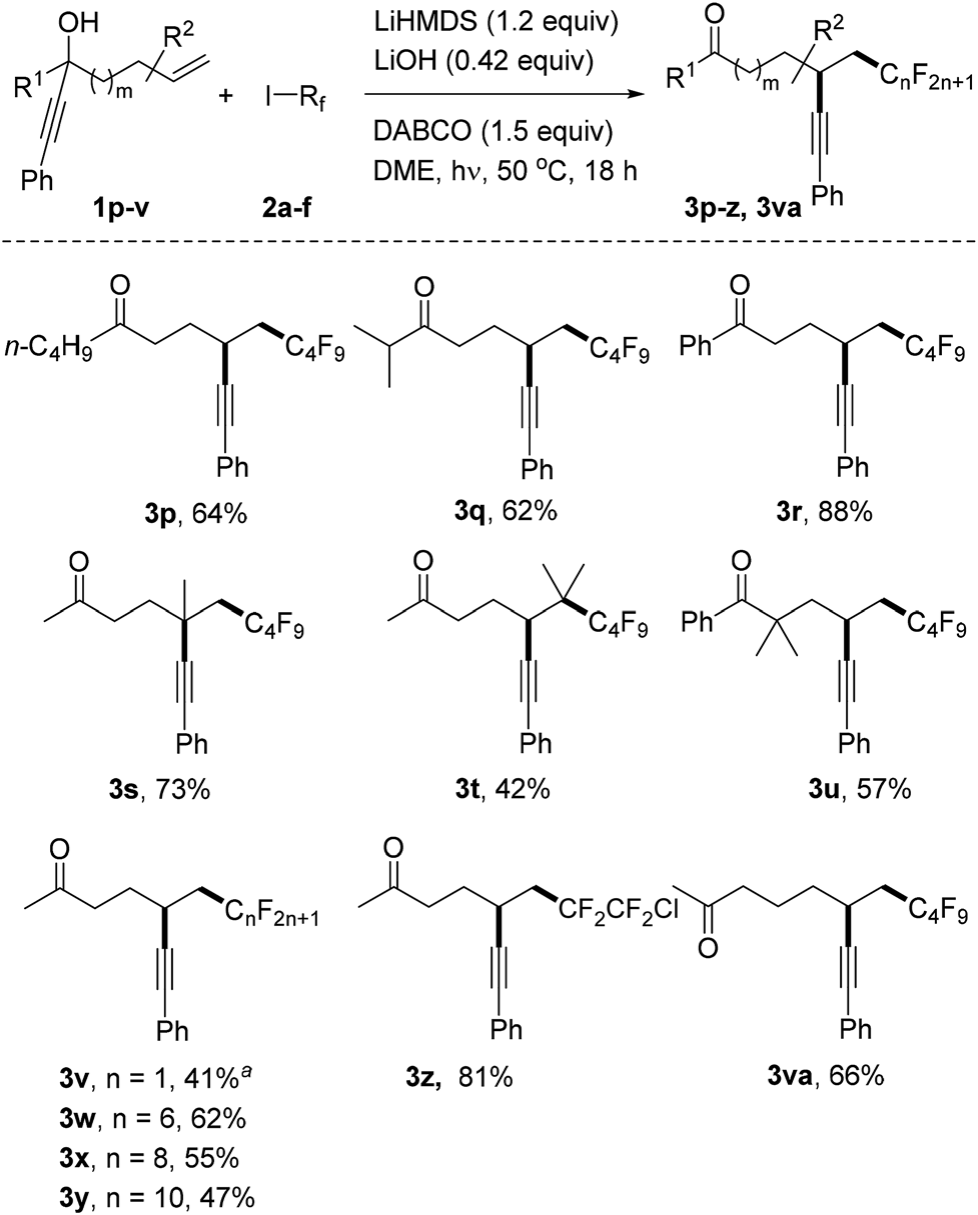
Variation of the radical acceptor and the perfluoroalkyl iodides. ^*a*^3.6 equivalents of CF_3_I were added in two parts and the reaction time was 24 hours.

We continued the studies by replacing the methyl group in **1a** with other alkyl and aryl substituents in combination with **2a** as the C-radical precursor (Scheme 2). The 1-butyl and 1-i-isopropyl propargylic alcohols **1p** and **1q** worked well and the dialkyl ketones **3p** and **3q** were isolated in 64% and 62% yield, respectively. An excellent yield was also obtained for the reaction with the tertiary benzylic alcohol **1r** to give **3r** (88%). Pleasingly, quaternary centres can be built up *via* the alkynyl migration as documented by the successful preparation of **3s** (73%). The trisubstituted alkene **1t** also acted as an acceptor and surprisingly an acceptable yield of the alkynyl migration product **3t** derived from initial addition at the more hindered alkene position was obtained. The geminally dimethylated alcohol **1u** provided the ketone **3u** in 57% yield. Notably, the perfluoroalkyl group in these product ketones is readily varied by switching the radical precursor (see **3v–y**). ICF_2_CF_2_Cl reacted chemoselectively at the C–I bond to give **3z** and the corresponding product derived from C–Cl cleavage was not identified. We also tested other alkyl iodides such as cyclohexyl iodide and adamantyl iodide that are typical substrates in radical I-atom transfer reactions. As expected, alkylation/alkynyl migration did not work due to the fact that such non-fluorinated alkyl iodides cannot be readily SET-reduced. In addition, we tested α-bromo acetophenone but could not identify the target product, and large amounts of starting material remained unreacted.^16^ We are currently not sure whether the problem lies in the initiation step or in the intermolecular SET transfer from the ketyl radical anion to the bromo ketone (see mechanistic discussion below). Importantly, the novel sequence is not restricted to 1,4-alkynyl migration and reaction of alcohol **1v** with perfluorobutyl iodide gave the ketone **3va** resulting from a 1,5-alkynyl migration in 66% yield.

To test whether alkynyl migration proceeds stereospecifically, we repeated the reaction with enantioenriched propargylic alcohol **1r** (25% ee) as the starting material in combination with perfluorobutyl iodide and obtained the desired product **3r** in 82% yield and 7% ee showing that only very low stereospecificity is achieved for this sequence (for details, see the ESI†).

**Scheme 3:**
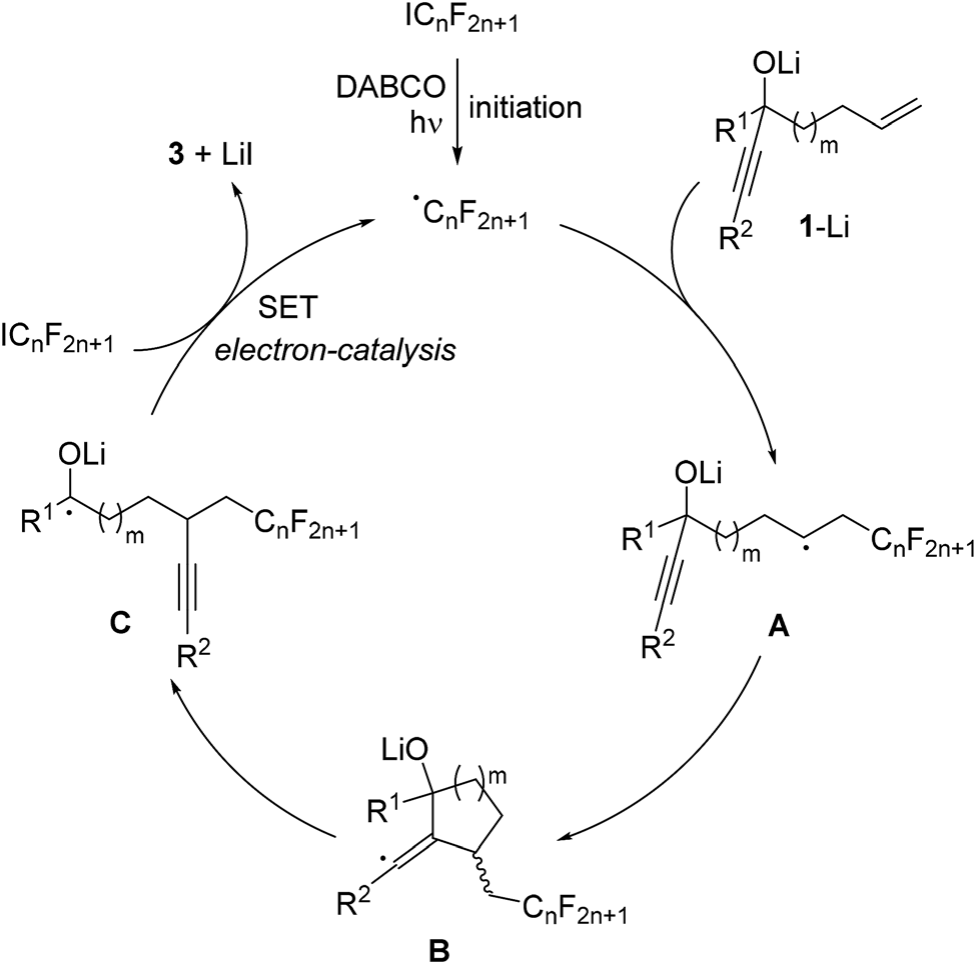
Proposed mechanism.

The proposed mechanism is depicted in Scheme 3. Initiation occurs by irradiation of the perfluoroalkyl iodide in the presence of DABCO^11^ to give the corresponding perfluoroalkyl radical. This C-radical then adds to the terminal position of the alkene in the deprotonated alcoholate **1**–Li to give the alkyl radical **A**. 5- or 6-exo radical cyclization leads to vinyl radical **B**, which further reacts by regioselective β-C–C bond cleavage to generate the ketyl radical anion **C**. As recently shown by us, such ketyl radical anions are good SET reducing reagents.^17^ Hence, electron transfer from **C** to the starting perfluoroalkyl iodide gives the product ketone **3** along with the corresponding perfluoroalkyl radical thereby sustaining the chain, and the overall cascade therefore belongs to an electron-catalyzed process.^18^

**Scheme 4:**
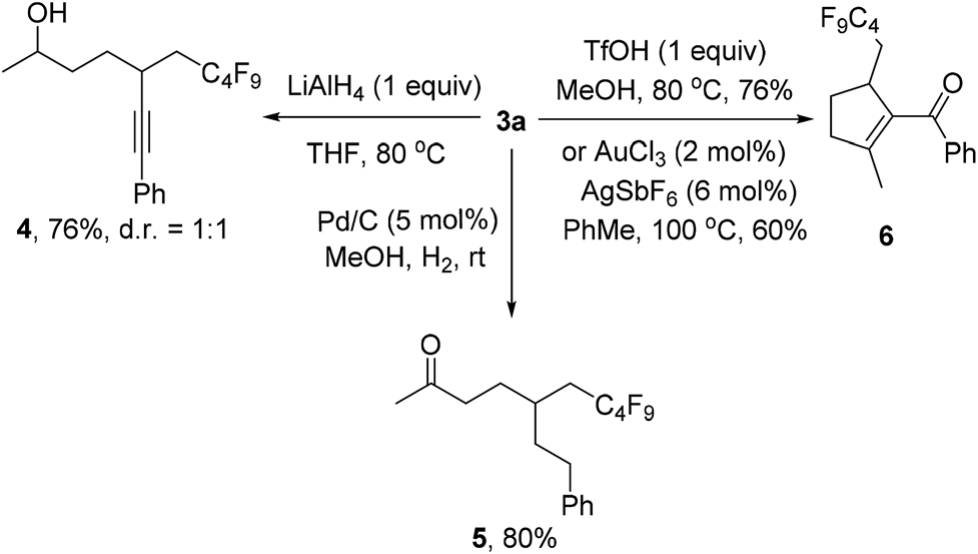
Follow-up chemistry.

We also investigated the follow-up chemistry using **3a** as the starting material (Scheme 4). Chemoselective reduction of the carbonyl group in **3a** was achieved using LiAlH_4_ at 80 °C to provide alcohol **4** in 76% yield as a 1 : 1 diastereoisomeric mixture. The triple bond in **3a** was chemoselectively hydrogenated with Pd/C and H_2_ to form ketone **5** in 80% yield. TfOH-catalyzed cyclization of **3a** afforded the α,β-unsaturated ketone **6** in 76% isolated yield.^19^ The same product could also be obtained using Au-catalysis, albeit in a slightly lower yield.^20^

## Conclusions

In summary, we have developed a novel and efficient method for the transition metal-free α-perfluoroalkylation β-alkynylation of unactivated alkenes *via* radical 1,4- or 1,5-alkynyl migration. Reaction conditions are mild and the substrate scope is broad. The starting propargylic alcohols are readily prepared and commercially available perfluoroalkyl iodides are used as C-radical precursors.

## Conflicts of interest

There are no conflicts of interest to declare.

## Supplementary Material

Supplementary information
