# Supplementary material for: α-Perfluoroalkyl-β-alkynylation of alkenes via radical alkynyl migration
Source: Chem Sci. 2017 Aug 4;8(10):6888–92. doi: 10.1039/c7sc02175e (PMC5637125; doi:10.1039/c7sc02175e)
Supplement: Supplementary file 1 [file SC-008-C7SC02175E-s001.pdf]

Electronic Supplementary Material (ESI) for Chemical Science.
This journal is © The Royal Society of Chemistry 2017

α-Perfluoroalkyl-β-alkynylation of Alkenes *via* Radical Alkynyl Migration

Xinjun Tang,[†] Armido Studer[*†]

[†] Organisch-Chemisches Institut, Westfälische Wilhelms-Universität, Corrensstraße 40, 48149 Münster, Germany

*E-mail: studer@uni-muenster.de

Supporting information

Table of Contents

1. Instrumentation and chemicals……………………………………………………………S1
2. Preparation of propargylic alcohols **1**……………………………………………………S2
3. Reaction of propargylic alcohols **1** with perfluoroalkyl iodides **2**………………………S10
4. Follow-up chemistry………………………………………………………………………S23
5. References…………………………………………………………………………………S25
6. ^{1}H and ^{13}C NMR spectra of compounds **1**, **3**, **4** and **5**………………………………S26

1. Instrumentation and chemicals

All reactions involving air- or moisture-sensitive reagents or intermediates were carried out in heat-gun-dried glassware under an argon atmosphere. For reactions with visible light a *Philips Master HPI-T Plus* (400 W) bulb was used. THF was freshly distilled from potassium under argon. All other solvents and reagents were purified according to standard procedures or were used as received from Aldrich, Acros Organics, Alfa Aesar. Propargylic alcohols **1** were synthesized according to literature procedures.[1] ^{1}H NMR and ^{13}C NMR spectra were recorded on a Bruker DPX 300, a Bruker AV 300, a Bruker AV 400 at 300 K. The solvents residual proton resonance and the respective carbon resonance ($CHCl_3$, δ = 7.26 ppm for ^{1}H NMR, δ = 77.0 ppm for ^{13}C NMR) was used for calibration. Merck silica gel 60 F 254 plates were used for TLC, detection with UV light and dipping into a solution of $KMnO_4$ (1.5 g in 400 mL H_2O, 5 g of $NaHCO_3$), followed by heating. Flash column chromatography (FC) was performed using Merck or Fluka silica gel 60 (40-63 μm) with a pressure of 0.3 bar. IR spectra were recorded on a Digilab Varian 3100 FT-IR Excalibur Series. Melting points (M.P.) were determined on a *SMP 10 apparatus* (*Stuart Scientific*). Mass spectra were recorded on a Bruker MicroTof or an Orbitrap LTQ XL (Nanospray) of Thermo Scientific.

2. Preparation of propargylic alcohols 1

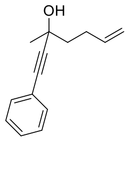

1a

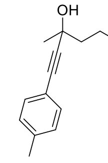

1b

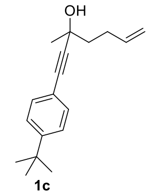

1c

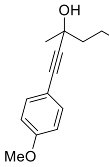

1d

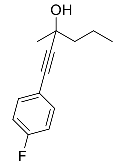

1e

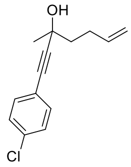

1f

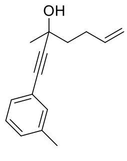

1g

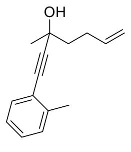

1h

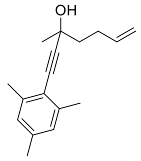

1i

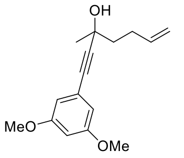

1j

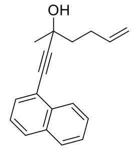

1k

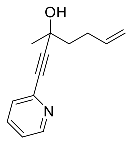

1l

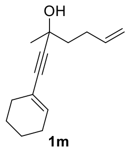

1m

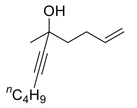

1n

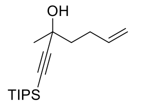

1o

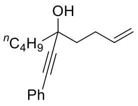

1p

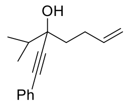

1q

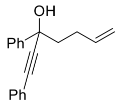

1r

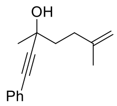

1s

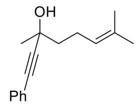

1t

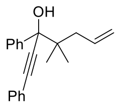

1u

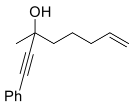

1v

General procedure (GP1): Propargylic alcohols **1** were synthesized according to literature procedures.[1] To a flame-dried flask equipped with a magnetic stir bar were added alkyne (5 mmol) and THF (35 mL). The mixture was then cooled to -78 ºC. *ⁿ*BuLi (1.2 equiv) was added to it dropwise and the mixture maintained at this temperature for 15 min. Then the ketone (1 equiv) in 5 mL of THF was added dropwise at -78 ºC. The reaction was allowed to warm to rt and stirring was continued overnight and then the reaction quenched with 50 mL of H_2O. The aqueous phase was extracted with 50 mL of ethyl acetate three times. The organic phase was combined and washed with 50 mL of brine, dried over Na_2SO_4, and concentrated. The residual was then purified by flash column chromatography (SiO_2) to obtain product **1**.

3-Methyl-1-phenylhept-6-en-1-yn-3-ol (1a)

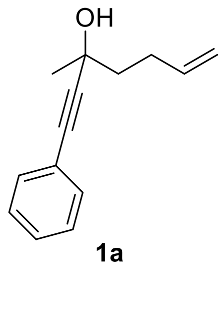

1a

1a[1] was synthesized according to GP1: **^{1}H NMR** (300 MHz, $CDCl_3$) δ 7.49 – 7.41 (m, 2H), 7.37 – 7.31 (m, 3H), 6.04 – 5.84 (m, 1H), 5.24 – 4.97 (m, 2H), 2.52 – 2.31 (m, 2H), 2.15 (s, 1H), 1.94 – 1.83 (m, 2H), 1.62 (s, 3H). **^{13}C NMR**

(100 MHz, $CDCl_3$) δ 138.37, 131.62, 128.29, 128.24, 114.92, 92.44, 83.69, 68.56, 42.67, 30.04, 29.36. **FTIR (neat)**: $\tilde{\nu}$ = 3376, 2978, 2933, 1641, 1598, 1490, 1443, 1370, 1109 cm^{-1}.

3-Methyl-1-(p-tolyl)hept-6-en-1-yn-3-ol (1b)

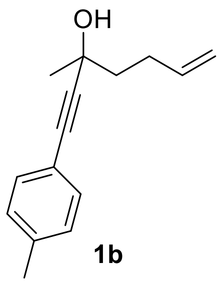

1b was synthesized according to GP1: **^{1}H NMR** (400 MHz, $CDCl_3$) δ7.35 – 7.29 (m, 2H), 7.15 – 7.07 (m, 2H), 5.99 – 5.83 (m, 1H), 5.16 – 4.96 (m, 2H), 2.47 – 2.32 (m, 5H), 2.09 (brs, 1H), 1.92 – 1.79 (m, 2H), 1.59 (s, 3H). **^{13}C NMR** (100 MHz, $CDCl_3$) δ 138.42, 138.36, 131.50, 128.98, 114.84, 91.75, 68.55, 42.71, 30.06, 29.36, 21.43. **FTIR (neat)**: $\tilde{\nu}$ = 3363, 2978, 2928, 1641, 1510, 1449, 1370, 1106 cm^{-1}. **HRMS (ESI)**: Exact mass calculated for $C_{17}H_{14}OSNa$ ($[M+Na]^+$): 237.1250, mass found: 237.1260.

1-(4-(Tert-butyl)phenyl)-3-methylhept-6-en-1-yn-3-ol (1c)

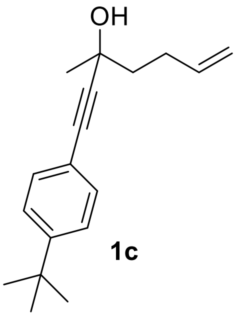

1c was synthesized according to GP1: **^{1}H NMR** (300 MHz, $CDCl_3$) δ 7.29 – 7.12 (m, 4H), 5.88 – 5.62 (m, 1H), 5.10 – 4.77 (m, 2H), 2.35 – 2.11 (m, 3H), 1.85 – 1.61 (m, 2H), 1.46 (s, 3H), 1.17 (s, 9H). **^{13}C NMR** (75 MHz, $CDCl_3$) δ 151.44, 138.40, 131.32, 125.17, 119.66, 114.75, 91.90, 83.72, 68.47, 42.76, 34.66, 31.10, 30.02, 29.32. **FTIR (neat)**: $\tilde{\nu}$ = 3358, 2964, 1641, 1507, 1462, 1364, 1267, 1105 cm^{-1}. **HRMS (ESI)**: Exact mass calculated for C14H15ClONa ($[M+Na]^+$): 279.1719, mass found: 279.1722.

3-Methyl-1-(p-tolyl)hept-6-en-1-yn-3-ol (1d)

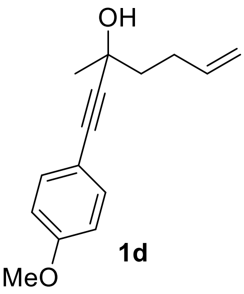

1d was synthesized according to GP1: **^{1}H NMR** (300 MHz, $CDCl_3$) δ 7.46 – 7.34 (m, 2H), 6.92 – 6.79 (m, 2H), 6.06 – 5.82 (m, 1H), 5.23 – 4.98 (m, 2H), 3.83 (s, 3H), 2.52 – 2.31 (m, 2H), 2.19 (s, 1H), 1.96 – 1.78 (m, 2H), 1.61 (s, 3H). **^{13}C NMR** (75 MHz, $CDCl_3$) δ 159.52, 138.43, 133.05, 114.83, 114.72, 113.84, 91.03, 83.54, 68.55, 55.25, 42.71, 30.09, 29.36. **FTIR (neat)**: $\tilde{\nu}$ = 3370, 2933, 1641, 1606, 1509, 1445, 1288, 1245, 1169, 1106, 1030 cm^{-1}. **HRMS (ESI)**: Exact mass calculated for $C_{17}H_{14}OSNa$ ($[M+Na]^+$): 253.1199, mass found: 253.1201.

1-(4-Fluorophenyl)-3-methylhept-6-en-1-yn-3-ol (1e)

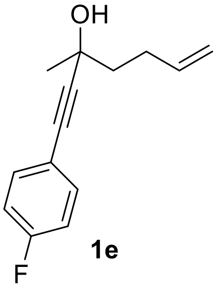

1e

1e was synthesized according to GP1: **^{1}H NMR** (300 MHz, $CDCl_3$) δ 7.51 – 7.37 (m, 2H), 7.13 – 6.94 (m, 2H), 6.06 – 5.77 (m, 1H), 5.24 – 4.97 (m, 2H), 2.50 – 2.30 (m, 2H), 2.21 (s, 1H), 1.95 – 1.83 (m, 2H), 1.61 (s, 3H). **^{13}C NMR** (75 MHz, $CDCl_3$) δ 164.14, 138.30, 133.56, 133.45, 115.65, 115.36, 114.90, 82.64, 68.51, 42.69, 29.99, 29.30. **FTIR (neat)**: $\tilde{\nu}$ = 3376, 3080, 2989, 2934, 1641, 1601, 1506, 1450, 1222, 1155, 1093 cm^{-1}. **HRMS (ESI)**: Exact mass calculated for $C_{14}H_{15}OFNa$ ($[M+Na]^+$): 241.0999, mass found: 241.1009.

1-(4-Chlorophenyl)-3-methylhept-6-en-1-yn-3-ol (1f)

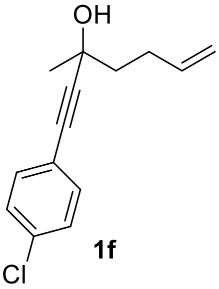

1f

1f was synthesized according to GP1: **^{1}H NMR** (300 MHz, $CDCl_3$) δ 7.39 – 7.35 (m, 2H), 7.33 – 7.28 (m, 2H), 6.00 – 5.86 (m, 1H), 5.19 – 4.99 (m, 2H), 2.48 – 2.30 (m, 2H), 2.15 (s, 1H), 1.95 – 1.82 (m, 2H), 1.61 (s, 3H). **^{13}C NMR** (75 MHz, $CDCl_3$) δ 138.24, 134.32, 132.85, 128.59, 121.14, 115.00, 93.42, 82.60, 68.55, 42.58, 29.96, 29.31. **FTIR (neat)**: $\tilde{\nu}$ = 3353, 2977, 2930, 1641, 1488, 1452, 1370, 1265, 1089, 1015 cm^{-1}. **HRMS (ESI)**: Exact mass calculated for $C_{14}H_{15}{}^{35}ClONa$ ($[M+Na]^+$): 257.0704, mass found: 257.0744.

3-Methyl-1-(m-tolyl)hept-6-en-1-yn-3-ol (1g)

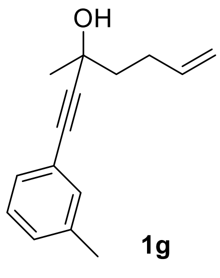

1g

1g was synthesized according to GP1: **^{1}H NMR** (300 MHz, $CDCl_3$) δ 7.31 – 7.13 (m, 4H), 6.05 – 5.85 (m, 1H), 5.23 – 4.99 (m, 2H), 2.47 – 2.31 (m, 5H), 2.21 (s, 1H), 1.98 – 1.81 (m, 2H), 1.63 (s, 3H). **^{13}C NMR** (75 MHz, $CDCl_3$) δ 138.40, 137.91, 132.21, 129.14, 128.68, 128.13, 122.48, 114.84, 92.15, 83.85, 68.53, 42.74, 30.05, 29.34, 21.14. **FTIR (neat)**: $\tilde{\nu}$ = 3374, 2970, 2927, 2856, 1641, 1601, 1582, 1481, 1449, 1369, 1111 cm^{-1}. **HRMS (ESI)**: Exact mass calculated for $C_{15}H_{18}ONa$ ($[M+Na]^+$): 237.1250, mass found: 237.1254.

3-Methyl-1-(*o*-tolyl)hept-6-en-1-yn-3-ol (1h)

1h was synthesized according to GP1: **^{1}H NMR** (300 MHz, $CDCl_3$) δ 7.43 (d, J = 7.2 Hz, 1H), 7.30 – 7.12 (m, 3H), 6.05 – 5.86 (m, 1H), 5.22 – 4.99 (m, 2H), 2.54 – 2.36 (m, 5H), 2.29 (s, 1H), 2.00 – 1.83

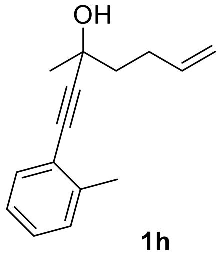

1h

(m, 2H), 1.66 (s, 3H). **^{13}C NMR** (75 MHz, $CDCl_3$) δ 140.04, 138.37, 131.90, 129.36, 128.28, 125.47, 122.42, 114.84, 96.59, 82.52, 68.69, 42.80, 30.17, 29.41, 20.65. **FTIR (neat)**: $\tilde{\nu}$ = 3349, 2977, 2926, 1641, 1485, 1452, 1370, 1117 cm^{-1}. **HRMS (ESI)**: Exact mass calculated for $C_{15}H_{18}ONa$ ($[M+Na]^+$): 237.1250, mass found: 237.1258.

1-Mesityl-3-methylhept-6-en-1-yn-3-ol (1i)

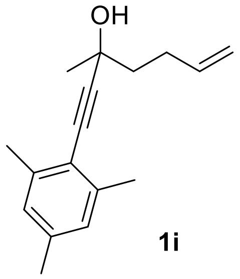

1i

1i was synthesized according to GP1: **^{1}H NMR** (300 MHz, $CDCl_3$) δ 6.89 (s, 2H), 6.05 – 5.87 (m, 1H), 5.20 – 4.98 (m, 2H), 2.51 – 2.37 (m, 8H), 2.31 (s, 3H), 2.21 (s, 1H), 2.00 – 1.84 (m, 2H), 1.67 (s, 3H). **^{13}C NMR** (75 MHz, $CDCl_3$) δ 140.06, 138.47, 137.71, 127.54, 119.36, 114.80, 100.38, 81.37, 68.92, 42.92, 30.41, 29.53, 21.24, 20.93. **FTIR (neat)**: $\tilde{\nu}$ = 3388, 2976, 2917, 2856, 1641, 1611, 1480, 1450, 1375, 1106, 1034 cm^{-1}. **HRMS (ESI)**: Exact mass calculated for $C_{17}H_{22}ONa$ ($[M+Na]^+$): 265.1563, mass found: 265.1572.

1-(3,5-Dimethoxyphenyl)-3-methylhept-6-en-1-yn-3-ol (1j)

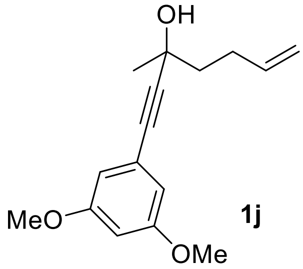

1j

1j was synthesized according to GP1: **^{1}H NMR** (300 MHz, $CDCl_3$) δ 6.57 (d, J = 2.3 Hz, 2H), 6.43 (t, J = 2.3 Hz, 1H), 6.01 – 5.81 (m, 1H), 5.18 – 4.94 (m, 2H), 3.77 (s, 6H), 2.47 – 2.30 (m, 2H), 2.24 (brs, 1H), 1.90 – 1.79 (m, 2H), 1.59 (s, 3H). **^{13}C NMR** (75 MHz, $CDCl_3$) δ 160.42, 138.32, 123.92, 114.92, 109.39, 101.69, 92.01, 83.63, 68.51, 55.39, 42.61, 29.98, 29.31. **FTIR (neat)**: $\tilde{\nu}$ = 3381, 2976, 2936, 2841, 1641, 1587, 1453, 1420, 1350, 1204, 1153, 1060 cm^{-1}. **HRMS (ESI)**: Exact mass calculated for $C_{16}H_{20}O_3Na$ ($[M+Na]^+$): 283.1305, mass found: 283.1317.

3-Methyl-1-(naphthalen-1-yl)hept-6-en-1-yn-3-ol (1k)

1k was synthesized according to GP1: **^{1}H NMR** (300 MHz, $CDCl_3$) δ 8.34 – 8.26 (m, 1H), 7.90 – 7.79 (m, 2H), 7.67 (dd, J = 7.2, 1.2 Hz, 1H), 7.63 – 7.49 (m, 2H), 7.42 (dd, J = 8.3, 7.1 Hz, 1H), 6.05 – 5.88 (m, 1H), 5.21 – 4.99 (m, 2H), 2.58 – 2.40 (m, 2H), 2.28 (brs, 1H), 2.03 – 1.93 (m, 2H), 1.72 (s, 3H). **^{13}C NMR** (75 MHz, $CDCl_3$) δ 138.34 , 133.19 , 133.09 , 130.46 , 128.78 , 128.27 , 126.77 , 126.36 ,

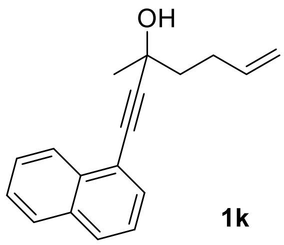

1k

125.94 , 125.12 , 120.22 , 115.01 , 97.46 , 81.73 , 68.86 , 42.78 , 30.22 , 29.50 . **FTIR (neat)**: $\tilde{\nu}$ = 3352, 3059, 2977, 2930, 2856, 1641, 1587, 1508, 1395, 1371, 1116 cm^{-1}. **HRMS (ESI)**: Exact mass calculated for $C_{18}H_{18}ONa$ ($[M+Na]^+$): 273.1250, mass found: 273.1256.

3-Methyl-1-(pyridin-2-yl)hept-6-en-1-yn-3-ol (1l)

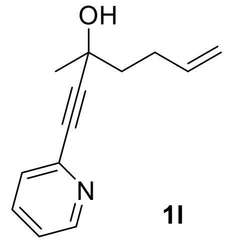

1l

1l was synthesized according to GP1: **^{1}H NMR** (300 MHz, $CDCl_3$) δδ 8.60 (d, *J* = 4.5 Hz, 2H), 7.67 (td, *J* = 7.7, 1.8 Hz, 1H), 7.44 (dt, *J* = 7.8, 1.1 Hz, 1H), 7.25 (ddd, *J* = 7.6, 4.9, 1.2 Hz, 1H), 6.03 – 5.81 (m, 1H), 5.21 – 4.92 (m, 2H), 2.76 (s, 1H), 2.53 – 2.28 (m, 1H), 1.98 – 1.85 (m, 2H), 1.65 (s, 3H). **^{13}C NMR** (75 MHz, $CDCl_3$) δ 149.87 , 142.92 , 138.24 , 136.15 , 127.09 , 122.88 , 114.88 , 92.78 , 82.95 , 68.29 , 42.43 , 29.79 , 29.17 . **FTIR (neat)**: $\tilde{\nu}$ = 3309, 2976, 2934, 1641, 1583, 1564, 1464, 1428, 1369, 1272, 1150 cm^{-1}. **HRMS (ESI)**: Exact mass calculated for $C_{13}H_{15}NONa$ ($[M+Na]^+$): 224.1046, mass found: 224.1056.

1-Cyclohexyl-3-methylhept-6-en-1-yn-3-ol (1m)

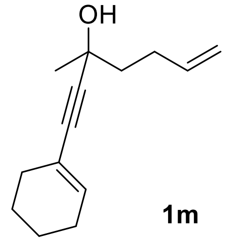

1m

1m was synthesized according to GP1: **^{1}H NMR** (300 MHz, $CDCl_3$) δ 6.15 – 6.08 (m, 1H), 6.00 – 5.82 (m, 1H), 5.17 – 4.94 (m, 2H), 2.44 – 2.22 (m, 2H), 2.19 – 2.04 (m, 5H), 1.87 – 1.74 (m, 2H), 1.71 – 1.56 (m, 4H), 1.53 (s, 3H). **^{13}C NMR** (75 MHz, $CDCl_3$) δ 138.49 , 134.90 , 120.13 , 114.68 , 89.82 , 85.46 , 68.42 , 42.79 , 30.13 , 29.33 , 29.25 , 25.57 , 22.26 , 21.46 . **FTIR (neat)**: $\tilde{\nu}$ = 3397, 2929, 1641, 1437, 1369, 1136, 1110 cm^{-1}. **HRMS (ESI)**: Exact mass calculated for $C_{14}H_{20}ONa$ ($[M+Na]^+$): 227.1406, mass found: 227.1414.

5-Methylundec-1-en-6-yn-5-ol (1n)

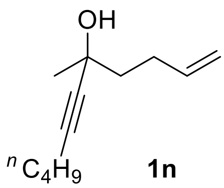

1n

1n was synthesized according to GP1: **^{1}H NMR** (300 MHz, $CDCl_3$) δ 6.02 – 5.76 (m, 1H), 5.19 – 4.91 (m, 2H), 2.42 – 2.16 (m, 4H), 2.06 (s, 1H), 1.85 – 1.65 (m, 2H), 1.60 – 1.35 (m, 7H), 0.93 (t, *J* = 7.1 Hz, 3H). **^{13}C NMR** (75 MHz, $CDCl_3$) δ 138.57 , 114.56 , 84.09 , 83.70 , 68.16 , 42.90 , 30.77 , 30.26 , 29.33 , 21.87 , 18.24 , 13.53 . **FTIR (neat)**: $\tilde{\nu}$ = 3350, 2959, 2932, 1642, 1455, 1369, 1125 cm^{-1}. **HRMS (ESI)**:

Exact mass calculated for $C_{12}H_{20}ONa$ ($[M+Na]^+$): 203.1406, mass found: 203.1413.

3-Methyl-1-(triisopropylsilyl)hept-6-en-1-yn-3-ol (1o)

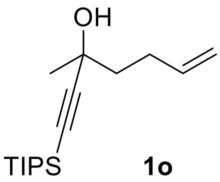

1o was synthesized according to GP1: **^{1}H NMR** (300 MHz, $CDCl_3$) δ 5.99 – 5.76 (m, 1H), 5.17 – 4.91 (m, 2H), 2.45 – 2.21 (m, 2H), 2.02 (s, 1H), 1.85 – 1.66 (m, 2H), 1.50 (s, 3H), 1.12 – 1.01 (m, 21H). **^{13}C NMR** (75 MHz, $CDCl_3$) 13C NMR (75 MHz, $CDCl_3$) δ 138.50, 114.79, 111.41, 83.80, 68.51, 42.77, 30.17, 29.47, 18.58, 11.15. **FTIR (neat)**: $\tilde{\nu}$ = 3381, 2943, 2865, 1642, 1463, 1367, 1119, 1073 cm^{-1}. **HRMS (ESI)**: Exact mass calculated for $C_{17}H_{32}OSiNa$ ($[M+Na]^+$): 303.2115, mass found: 303.2124.

5-(Phenylethynyl)non-1-en-5-ol (1p)

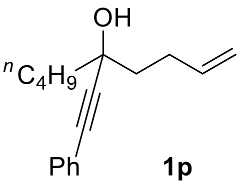

1p was synthesized according to GP1: **^{1}H NMR** (300 MHz, $CDCl_3$) δ 7.51 – 7.43 (m, 2H), 7.40 – 7.30 (m, 3H), 6.06 – 5.84 (m, 1H), 5.21 – 4.99 (m, 2H), 2.52 – 2.33 (m, 2H), 2.14 (s, 1H), 1.93 – 1.73 (m, 4H), 1.68 – 1.52 (m, 2H), 1.51 – 1.35 (m, 2H), 0.99 (t, J = 7.3 Hz, 3H).. **^{13}C NMR** (75 MHz, $CDCl_3$) δ 138.57 , 131.65 , 128.24 , 122.82 , 114.79 , 91.82 , 84.72 , 71.60 , 42.10 , 41.14 , 28.97 , 26.51 , 22.88 , 14.05 . **FTIR (neat)**: $\tilde{\nu}$ = 3374, 2956, 2934, 2862, 1641, 1599, 1490, 1443, 1379, 1340, 1256, 1135 cm^{-1}. **HRMS (ESI)**: Exact mass calculated for $C_{17}H_{22}ONa$ ($[M+Na]^+$): 265.1563, mass found: 265.1566.

3-Isopropyl-1-phenylhept-6-en-1-yn-3-ol (1q)

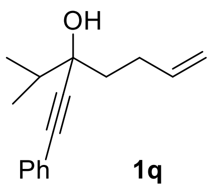

1q was synthesized according to GP1: **^{1}H NMR** (300 MHz, $CDCl_3$) δ 7.48 – 7.40 (m, 2H), 7.35 – 7.28 (m, 3H), 6.01 – 5.85 (m, 1H), 5.20 – 4.96 (m, 2H), 2.52 – 2.35 (m, 2H), 2.11 (s, 1H), 2.01 – 1.70 (m, 3H), 1.12 (d, J = 5.1 Hz, 3H), 1.09 (d, J = 5.1 Hz, 3H). **^{13}C NMR** (75 MHz, $CDCl_3$) δ 138.77 , 131.64 , 128.22 , 128.19 , 122.86 , 114.73 , 90.86 , 85.38 , 74.96 , 38.37 , 37.88 , 28.87 , 17.97 , 17.09 . **FTIR (neat)**: $\tilde{\nu}$ = 3424, 2864, 1641, 1598, 1490, 1444, 1369, 1140, 1029 cm^{-1}. **HRMS (ESI)**: Exact mass calculated for $C_{16}H_{20}ONa$ ($[M+Na]^+$): 251.1406, mass found: 251.1410.

1,3-Diphenylhept-6-en-1-yn-3-ol (1r)

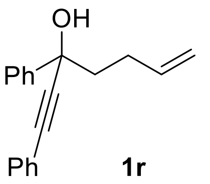

1r

1r was synthesized according to GP1: yellow solid, **MP**: 69 ºC; **^{1}H NMR** (300 MHz, $CDCl_3$) δ 7.77 – 7.67 (m, 2H), 7.56 – 7.45 (m, 2H), 7.47 – 7.27 (m, 6H), 5.94 – 5.76 (m, 1H), 5.11 – 4.92 (m, 2H), 2.50 (s, 1H), 2.45 – 2.31 (m, 1H), 2.31 – 2.00 (m, 3H). **^{13}C NMR** (75 MHz, $CDCl_3$) δ 144.62 , 138.09 , 131.74 , 128.54 , 128.32 , 128.25 , 127.77 , 125.47 , 122.54 , 114.77 , 91.24 , 86.38 , 73.60 , 44.52 , 29.30 . **FTIR (neat)**: $\tilde{\nu}$ = 3273, 3070, 2945, 1641, 1489, 1449, 1388, 1306, 1014 cm^{-1}. **HRMS (ESI)**: Exact mass calculated for $C_{19}H_{18}ONa$ ($[M+Na]^+$): 285.1250, mass found: 285.1256.

Chiral **1r** was synthesized according to GP1 and literature[2]: **^{1}H NMR** (300 MHz, $CDCl_3$) δ 7.76 – 7.68 (m, 2H), 7.55 – 7.46 (m, 2H), 7.44 – 7.29 (m, 6H), 5.96 – 5.76 (m, 1H), 5.12 – 4.93 (m, 2H), 2.54 (s, 1H), 2.45 – 2.32 (m, 1H), 2.31 – 2.01 (m, 3H). The pure product was analyzed by chiral HPLC. Chiral HPLC (Chiralcel OD-H column with guard, 25 °C, 1 mL/min, cyclohexane:isopropanol = 90:10); Elution time: t_{major} = 14.6 min and t_{minor} = 23.0 min, enantiomeric excess (25%).

3,6-Dimethyl-1-phenylhept-6-en-1-yn-3-ol (1s)

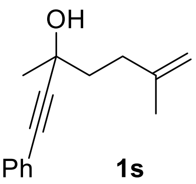

1s

1s was synthesized according to GP1: **^{1}H NMR** (300 MHz, $CDCl_3$) δ 7.47 – 7.38 (m, 2H), 7.35 – 7.27 (m, 3H), 4.83 – 4.73 (m, 2H), 2.45 – 2.22 (m, 3H), 2.02 – 1.84 (m, 2H), 1.79 (s, 3H), 1.61 (s, 3H). **^{13}C NMR** (75 MHz, $CDCl_3$) δ 145.72 , 131.61 , 128.21 , 122.71 , 110.17 , 92.53 , 83.65 , 68.63 , 41.61 , 33.13 , 29.97 , 22.58 . **FTIR (neat)**: $\tilde{\nu}$ = 3355, 2978, 2931, 1649, 1599, 1490, 1444, 1374, 1341, 1261, 1201, 1118, 1099 cm^{-1}. **HRMS (ESI)**: Exact mass calculated for $C_{15}H_{18}ONa$ ($[M+Na]^+$): 237.1250, mass found: 237.1271.

3,7-Dimethyl-1-phenyloct-6-en-1-yn-3-ol (1t)

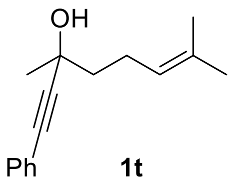

1t

1t was synthesized according to GP1: **^{1}H NMR** (300 MHz, $CDCl_3$) δ 7.48 – 7.39 (m, 2H), 7.35 – 7.27 (m, 3H), 5.27 – 5.16 (m, 1H), 2.46 – 2.14 (m, 3H), 1.84 – 1.76 (m, 2H), 1.73 – 1.70 (m, 3H), 1.68 (s, 3H), 1.59 (s, 3H). **^{13}C NMR** (75 MHz, $CDCl_3$) δ 132.44 , 131.62 , 128.21 , 128.17 , 123.87 , 122.84 , 92.78 , 83.53 , 68.82 , 43.49 , 29.92 , 25.69 , 23.83 , 17.71 . **FTIR (neat)**: $\tilde{\nu}$ = 3363, 2971, 2919, 2854, 1598, 1490, 1444, 1375, 1267, 1186, 1117 cm^{-1}. **HRMS (ESI)**: Exact mass calculated for $C_{16}H_{20}ONa$ ($[M+Na]^+$): 251.1406, mass found: 251.1405.

4,4-Dimethyl-1,3-diphenylhept-6-en-1-yn-3-ol (1u)

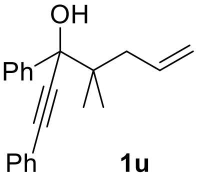

1u

1u was synthesized according to GP1: **^{1}H NMR** (300 MHz, $CDCl_3$) δ 7.74 – 7.66 (m, 2H), 7.55 – 7.47 (m, 2H), 7.40 – 7.30 (m, 6H), 5.98 – 5.81 (m, 1H), 5.13 – 5.00 (m, 2H), 2.50 (s, 1H), 2.40 – 2.27 (m, 2H), 1.05 (s, 6H). **^{13}C NMR** (75 MHz, $CDCl_3$) δ 141.88 , 135.95 , 131.65 , 128.42 , 128.31 , 127.90 , 127.46 , 127.13 , 122.77 , 117.38 , 92.18 , 86.36 , 79.72 , 42.63 , 41.92 , 28.25 , 22.48 , 21.60 . **FTIR (neat)**: $\tilde{\nu}$ = 3472, 3065, 2971, 2938, 1727, 1683, 1599, 1491, 1444, 1385, 1364, 1312, 1255, 1171, 1162, 1046 cm^{-1}. **HRMS (ESI)**: Exact mass calculated for $C_{21}H_{22}ONa$ ($[M+Na]^+$): 313.1563, mass found: 313.1568.

3-Methyl-1-phenyloct-7-en-1-yn-3-ol (1v)

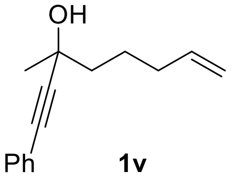

1v

1v was synthesized according to GP1: **^{1}H NMR** (300 MHz, $CDCl_3$) δ 7.50 – 7.40 (m, 2H), 7.39 – 7.30 (m, 3H), 5.97 – 5.77 (m, 1H), 5.15 – 4.96 (m, 2H), 2.24 – 2.11 (m, 2H), 2.06 (s, 1H), 1.87 – 1.67 (m, 4H), 1.61 (s, 3H). **^{13}C NMR** (75 MHz, $CDCl_3$) δ 138.51 , 131.64 , 128.23 , 122.77 , 114.76 , 92.83 , 83.40 , 68.55 , 43.19 , 33.70 , 29.94 , 24.07 . **FTIR (neat)**: $\tilde{\nu}$ = 3383, 3079, 2977, 2935, 2865, 2203, 1676, 1641, 1599, 1490, 1444, 1370, 1157 cm^{-1}. **HRMS (ESI)**: Exact mass calculated for $C_{15}H_{18}ONa$ ($[M+Na]^+$): 237.1250, mass found: 237.1256.

3. Reaction of propargylic alcohols 1 with perfluoroalkyl iodides 2

General procedure 2 (GP2): To a flame-dried Schlenk tube equipped with a magnetic stir bar LiOH (0.42 equiv), LiHMDS (1.2 equiv), **1** (0.1 mmol), and 1.25 mL of DME were added sequentially under Ar atmosphere. The mixture was allowed to stir at room temperature for 0.5 h. After that DABCO (1.5 equiv), and **2** (1.8 equiv) were added to the reaction mixture under Ar atmosphere. Then the reaction mixture was stirred under visible-light irradiation (temperature up to 50 °C) for 18 h. After that, the solvent was removed and the residue was purified by flash column chromatography (SiO_2) to obtain product **3**.

7,7,8,8,9,9,10,10,10-Nonafluoro-5-(phenylethynyl)decan-2-one (3a)

3a was synthesized according to GP2 with **1a** (20 mg, 0.100 mmol), LiHMDS (0.12 mL, 0.120 mmol, 1.0 M in THF solution), LiOH (1 mg, 0.042 mmol), DABCO (17 mg, 0.152 mmol), and **2a** (31 µL,

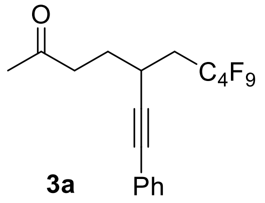

3a

0.180 mmol) in 1.25 mL of DME under visible-light at 50 ºC for 18 h. Purification by flash column chromatography (pentane/ethyl ether = 10/1) provided **3a** as a liquid (33 mg, 79%); **^{1}H NMR** (300 MHz, $CDCl_3$) δ 7.39 (dd, *J* = 6.6, 3.2 Hz, 2H), 7.34 – 7.27 (m, 3H), 3.10 (ddd, *J* = 10.2, 5.0, 3.1 Hz, 1H), 2.83 – 2.65 (m, 2H), 2.58 – 2.22 (m, 2H), 2.19 (s, 3H), 2.11 – 1.96 (m, 1H), 1.89 – 1.74 (m, 1H). **^{13}C NMR** (75 MHz, $CDCl_3$) δ 207.44 , 131.59 , 128.28 , 128.18 , 89.26 , 40.89 , 36.21 (t, *J* = 20.9 Hz), 30.09 , 29.10 , 24.71 (t, *J* = 3.0 Hz). **^{19}F NMR** (282 MHz, $CDCl_3$) δ -81.02 (tt, *J* = 9.7, 3.4 Hz, 3F), -113.32 – -113.51 (m, 2F), -124.38 – -124.58 (m, 2F), -125.73 – -125.95 (m, 2F). **FTIR (neat)**: $\tilde{\nu}$ = 2947, 1718, 1599, 1491, 1443, 1356, 1217, 1131, 1110 cm^{-1}. **HRMS (ESI)**: Exact mass calculated for $C_{18}H_{15}OF_9Na$ ([M+Na]$^+$): 441.0871, mass found: 441.0887.

7,7,8,8,9,9,10,10,10-Nonafluoro-5-(p-tolylethynyl)decan-2-one (3b)

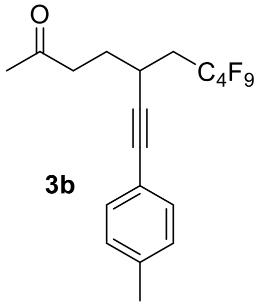

3b

3b was synthesized according to GP2 with **1b** (21 mg, 0.098 mmol), LiHMDS (0.12 mL, 0.120 mmol, 1.0 M in THF solution), LiOH (1 mg, 0.042 mmol), DABCO (17 mg, 0.152 mmol), and **2a** (31 μL, 0.180 mmol) in 1.25 mL of DME under visible-light at 50 ºC for 18 h. Purification by flash column chromatography (pentane/ethyl ether = 10/1) provided **3b** as a liquid (32 mg, 76%); **^{1}H NMR** (300 MHz, $CDCl_3$) δ 7.29 (d, *J* = 8.1 Hz, 2H), 7.14 – 7.08 (m, 2H), 3.08 (ddd, *J* = 10.2, 5.0, 3.0 Hz, 1H), 2.84 – 2.63 (m, 2H), 2.34 (brs, 5H), 2.19 (s, 3H), 2.11 – 1.95 (m, 1H), 1.88 – 1.71 (m, 1H). **^{13}C NMR** (75 MHz, $CDCl_3$) δ 207.52 , 138.27 , 131.45 , 129.02 , 88.50 , 40.90 , 36.50 (d, *J* = 21.2 Hz), 36.22 , 35.95 , 30.07 , 29.13 , 24.76 (d, *J* = 3.0 Hz), 24.71 , 24.67 , 21.39 . **^{19}F NMR** (282 MHz, $CDCl_3$) δ -81.04 (tt, *J* = 9.7, 3.4 Hz, 3F), -113.34 – -113.55 (m, 2F), -124.37 – -124.64 (m, 2F), -125.73 – -125.99 (m, 2F). **FTIR (neat)**: $\tilde{\nu}$ = 2946, 1719, 1511, 1356, 1217,1131, 1021 cm^{-1}. **HRMS (ESI)**: Exact mass calculated for $C_{19}H_{17}OF_9Na$ ([M+Na]$^+$): 455.1028, mass found: 455.1044.

5-((4-(*Tert*-butyl)phenyl)ethynyl)-7,7,8,8,9,9,10,10,10-nonafluorodecan-2-one (3c)

3c was synthesized according to GP2 with **1c** (26 mg, 0.101 mmol), LiHMDS (0.12 mL, 0.120 mmol, 1.0 M in THF solution), LiOH (1 mg, 0.042 mmol), DABCO (17 mg, 0.152 mmol), and **2a** (31 μL, 0.180 mmol) in 1.25 mL of DME under visible-light at 50 ºC for 18 h. Purification by flash column

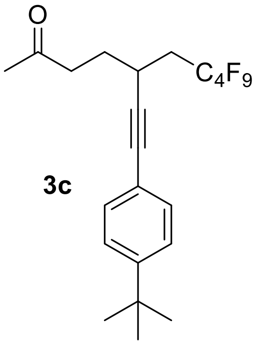

3c

chromatography (pentane/ethyl ether = 10/1) provided **3c** as a liquid (27 mg, 56%); **^{1}H NMR** (300 MHz, $CDCl_3$) δ 7.33 (s, 4H), 3.15 – 3.01 (m, 1H), 2.84 – 2.64 (m, 2H), 2.55 – 2.22 (m, 2H), 2.19 (s, 3H), 2.09 – 1.96 (m, 1H), 1.87 – 1.73 (m, 1H), 1.31 (s, 9H). **^{13}C NMR** (75 MHz, $CDCl_3$) δ 207.51 , 151.48 , 131.31 , 125.27 , 119.93 , 88.54 , 83.36 , 40.90 , 36.25 (t, *J* = 21.0 Hz), 34.73 , 31.15 , 30.08 , 29.14 , 24.71 (t, *J* = 3.0 Hz). **^{19}F NMR** (282 MHz, $CDCl_3$) δ -81.03 (tt, *J* = 9.7, 3.4 Hz, 3F), -113.33 – -113.54 (m, 2F), -124.39 – -124.62 (m, 2F), -125.76 – -125.98 (m, 2F). **FTIR (neat)**: $\tilde{\nu}$ = 2965, 1719, 1355, 1217, 1163, 1131, 1107, 1017 cm^{-1}. **HRMS (ESI)**: Exact mass calculated for $C_{22}H_{23}OF_9Na$ ($[M+Na]^+$): 497.1497, mass found: 497.1504.

7,7,8,8,9,9,10,10,10-Nonafluoro-5-((4-methoxyphenyl)ethynyl)decan-2-one (3d)

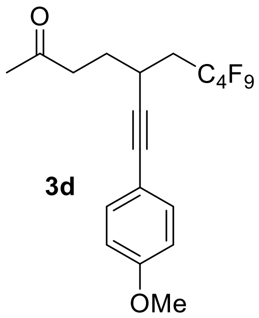

3d

3d was synthesized according to GP2 with **1f** (23 mg, 0.100 mmol), LiHMDS (0.12 mL, 0.120 mmol, 1.0 M in THF solution), LiOH (1 mg, 0.042 mmol), DABCO (17 mg, 0.152 mmol), and **2a** (31 μL, 0.180 mmol) in 1.25 mL of DME under visible-light at 50 °C for 18 h. Purification by flash column chromatography (pentane/ethyl ether = 10/1) provided **3f** as a liquid (30 mg, 67%); **^{1}H NMR** (300 MHz, $CDCl_3$) δ 7.32 (d, *J* = 8.7 Hz, 2H), 6.83 (d, *J* = 8.7 Hz, 2H), 3.80 (s, 3H), 3.14 – 3.00 (m, 1H), 2.74 (dt, *J* = 8.4, 3.5 Hz, 2H), 2.55 – 2.21 (m, 2H), 2.19 (s, 3H), 2.01 (ddd, *J* = 8.6, 6.8, 3.9 Hz, 1H), 1.80 (ddd, *J* = 10.2, 5.9, 3.8 Hz, 1H). **^{13}C NMR** (75 MHz, $CDCl_3$) δ 207.55 , 159.54 , 132.96 , 115.07 , 113.92 , 87.75 , 83.10 , 55.27 , 40.93 , 36.24 (t, *J* = 20.9 Hz), 30.08 , 29.16 , 24.72 (t, *J* = 2.9 Hz). **^{19}F NMR** (282 MHz, $CDCl_3$) δ -81.04 (tt, *J* = 9.7, 3.3 Hz, 3F), -113.37 – -113.56 (m, 2F), -124.42 – -124.60 (m, 2F), -125.76 – -125.98 (m, 2F). **FTIR (neat)**: $\tilde{\nu}$ = 2938, 1718, 1608, 1511, 1444, 1365, 1290, 1232, 1219, 1172, 1132, 1107, 1031 cm^{-1}. **HRMS (ESI)**: Exact mass calculated for $C_{19}H_{17}O_2F_9Na$ ($[M+Na]^+$): 471.0977, mass found: 471.0991.

7,7,8,8,9,9,10,10,10-Nonafluoro-5-((4-fluorophenyl)ethynyl)decan-2-one (3e)

3e was synthesized according to GP2 with **1e** (23 mg, 0.100 mmol), LiHMDS (0.12 mL, 0.120 mmol, 1.0 M in THF solution), LiOH (1 mg, 0.042 mmol), DABCO (17 mg, 0.152 mmol), and **2a** (31 μL, 0.180 mmol) in 1.25 mL of DME under visible-light at 50 °C for 18 h. Purification by flash column

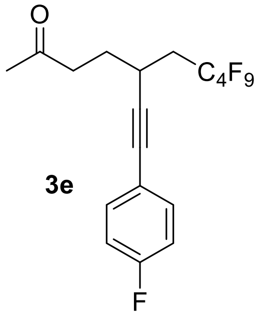

3e

chromatography (pentane/ethyl ether = 10/1) provided **3e** as a liquid (30 mg, 68%); **^{1}H NMR** (300 MHz, $CDCl_3$) δ 7.33 – 7.24 (m, 2H), 6.97 – 6.87 (m, 2H), 3.00 (ddt, *J* = 9.9, 8.2, 4.9 Hz, 1H), 2.77 – 2.55 (m, 2H), 2.50 – 2.15 (m, 2H), 2.12 (s, 3H), 2.02 – 1.88 (m, 1H), 1.81 – 1.65 (m, 1H). **^{13}C NMR** (75 MHz, $CDCl_3$) δ 207.36 , 162.43 (d, *J* = 249.2 Hz), 133.43 (d, *J* = 8.3 Hz), 118.99 (d, *J* = 3.5 Hz), 115.52 (d, *J* = 22.2 Hz), 88.92 (d, *J* = 1.5 Hz), 82.25 , 40.84 , 36.14 (t, *J* = 21.0 Hz), 30.06 , 29.04 , 24.65 (t, *J* = 3.0 Hz). **^{19}F NMR** (282 MHz, $CDCl_3$) δ -81.04 (tt, *J* = 9.7, 3.3 Hz, 3F), -111.14 – -111.24 (m, 1F), -113.36 – -113.59 (m, 2F), -124.41 – -124.62 (m, 2F), -125.76 – -125.99 (m, 2F). **FTIR (neat)**: $\tilde{\nu}$ = 2949, 1718, 1603, 1508, 1356, 1218, 1157, 1132, 1015 cm^{-1}. **HRMS (ESI)**: Exact mass calculated for $C_{18}H_{14}OF_{10}Na$ ($[M+Na]^+$): 459.0777, mass found: 459.0788.

5-((4-Chlorophenyl)ethynyl)-7,7,8,8,9,9,10,10,10-nonafluorodecan-2-one (3f)

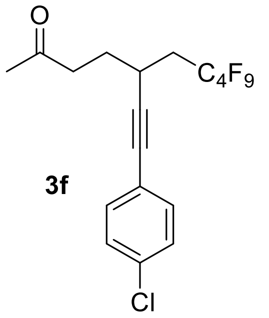

3f

3f was synthesized according to GP2 with **1c** (23 mg, 0.098 mmol), LiHMDS (0.12 mL, 0.120 mmol, 1.0 M in THF solution), LiOH (1 mg, 0.042 mmol), DABCO (17 mg, 0.152 mmol), and **2a** (31 μL, 0.180 mmol) in 1.25 mL of DME under visible-light at 50 °C for 18 h. Purification by flash column chromatography (pentane/ethyl ether = 10/1) provided **3c** as a liquid (37 mg, 83%); **^{1}H NMR** (300 MHz, $CDCl_3$) δ 7.26 – 7.18 (m, 4H), 3.01 (ddt, *J* = 9.9, 8.2, 4.9 Hz, 1H), 2.75 – 2.54 (m, 2H), 2.48 – 2.15 (m, 2H), 2.12 (s, 3H), 1.94 (dq, *J* = 10.9, 3.9, 3.4 Hz, 1H), 1.80 – 1.68 (m, 1H). **^{13}C NMR** (75 MHz, $CDCl_3$) δ 207.30 , 134.22 , 132.80 , 128.60 , 121.40 , 90.28 , 82.23 , 40.81 , 36.08 (t, *J* = 21.0 Hz), 30.07 , 28.98 , 24.69 (t, *J* = 3.3 Hz). **^{19}F NMR** (282 MHz, $CDCl_3$) δ -81.03 (tt, *J* = 9.7, 3.3 Hz, 3F), -113.28 – -113.65 (m, 2F), -124.39 – -124.59 (m, 2F), -125.77 – -125.96 (m, 2F). **FTIR (neat)**: $\tilde{\nu}$ = 2947, 1718, 1491, 1357, 1234, 1222, 1165, 1134, 10921016 cm^{-1}. **HRMS (ESI)**: Exact mass calculated for $C_{18}H_{14}O^{35}ClF_9Na$ ($[M+Na]^+$): 475.0482, mass found: 475.0484.

7,7,8,8,9,9,10,10,10-Nonafluoro-5-(*m*-tolylethynyl)decan-2-one (3g)

3g was synthesized according to GP2 with **1g** (21 mg, 0.098 mmol), LiHMDS (0.12 mL, 0.120 mmol, 1.0 M in THF solution), LiOH (1 mg, 0.042 mmol), DABCO (17 mg, 0.152 mmol), and **2a** (31 μL,

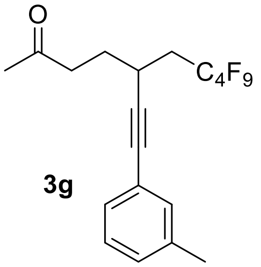

3g

0.180 mmol) in 1.25 mL of DME under visible-light at 50 ºC for 18 h. Purification by flash column chromatography (pentane/ethyl ether = 10/1) provided **3g** as a liquid (29 mg, 68%); **^{1}H NMR** (300 MHz, $CDCl_3$) δ 7.24 – 7.15 (m, 3H), 7.15 – 7.09 (m, 1H), 3.09 (ddt, *J* = 9.9, 8.0, 4.9 Hz, 1H), 2.83 – 2.64 (m, 2H), 2.55-2.23 (m, 5H), 2.19 (s, 3H), 2.07 – 1.96 (m, 1H), 1.80 (dd, *J* = 10.2, 6.0 Hz, 1H). **^{13}C NMR** (75 MHz, $CDCl_3$) δ 207.48 , 137.97 , 132.13 , 129.05 , 128.64 , 128.16 , 122.72 , 88.83 , 83.43 , 40.87 , 36.19 (t, *J* = 21.0 Hz), 30.07 , 29.10 , 24.67 (t, *J* = 3.2 Hz), 21.14 . **^{19}F NMR** (282 MHz, $CDCl_3$) δ -81.02 (tt, *J* = 9.7, 3.3 Hz, 3F), -113.37 – -113.57 (m, 2F), -124.39 – -124.62 (m, 2F), -125.75 – -126.01 (m, 2F). **FTIR (neat)**: $\tilde{\nu}$ = 2946, 2361, 1719, 1487, 1356, 1234, 1134 cm^{-1}. **HRMS (ESI)**: Exact mass calculated for $C_{19}H_{17}OF_9Na$ ($[M+Na]^+$): 455.1028, mass found: 455.1044.

7,7,8,8,9,9,10,10,10-Nonafluoro-5-(*o*-tolylethynyl)decan-2-one (3h)

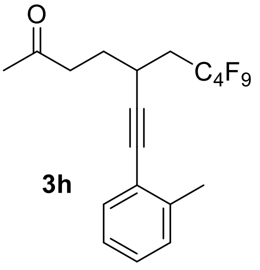

3h

3h was synthesized according to GP2 with **1h** (21 mg, 0.098 mmol), LiHMDS (0.12 mL, 0.120 mmol, 1.0 M in THF solution), LiOH (1 mg, 0.042 mmol), DABCO (17 mg, 0.152 mmol), and **2a** (31 μL, 0.180 mmol) in 1.25 mL of DME under visible-light at 50 ºC for 18 h. Purification by flash column chromatography (pentane/ethyl ether = 10/1) provided **3h** as a liquid (33 mg, 78%); **^{1}H NMR** (300 MHz, $CDCl_3$) δ 7.36 (dd, *J* = 7.3, 1.2 Hz, 1H), 7.23 – 7.16 (m, 2H), 7.16 – 7.08 (m, 1H), 3.14 (ddt, *J* = 9.8, 8.4, 4.8 Hz, 1H), 2.77 (td, *J* = 7.6, 6.9, 1.9 Hz, 2H), 2.54-2.23 (m, 5H), 2.19 (s, 3H), 2.11 – 1.98 (m, 1H), 1.87 – 1.76 (m, 1H). **^{13}C NMR** (75 MHz, $CDCl_3$) δ 207.44 , 140.02 , 131.94 , 129.42 , 128.18 , 125.51 , 122.70 , 93.19 , 82.20 , 40.88 , 36.37 (t, *J* = 21.1 Hz), 30.08 , 29.26 , 24.82 (t, *J* = 3.1 Hz), 20.57 . **^{19}F NMR** (282 MHz, $CDCl_3$) δ -81.03 (tt, *J* = 9.7, 3.3 Hz, 3F), -113.12 – -113.51 (m, 2F), -124.43 – -124.65 (m, 2F), -125.75 – -126.00 (m, 2F). **FTIR (neat)**: $\tilde{\nu}$ = 2954, 1719, 1487, 1356, 1220, 1165, 1133, 1037 cm^{-1}. **HRMS (ESI)**: Exact mass calculated for $C_{19}H_{17}OF_9Na$ ($[M+Na]^+$): 455.1028, mass found: 455.1045.

7,7,8,8,9,9,10,10,10-Nonafluoro-5-(mesitylethynyl)decan-2-one (3i)

3i was synthesized according to GP2 with **1i** (24 mg, 0.099 mmol), LiHMDS (0.12 mL, 0.120 mmol, 1.0 M in THF solution), LiOH (1 mg, 0.042 mmol), DABCO (17 mg, 0.152 mmol), and **2a** (31 μL,

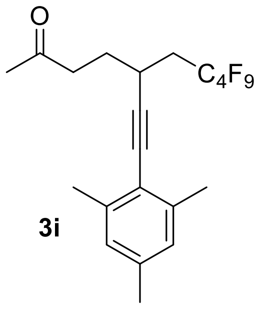

3i

0.180 mmol) in 1.25 mL of DME under visible-light at 50 ºC for 18 h. Purification by flash column chromatography (pentane/ethyl ether = 10/1) provided **3i** as a liquid (28 mg, 61%); **^{1}H NMR** (300 MHz, $CDCl_3$) δ 6.86 (s, 2H), 3.18 (dq, *J* = 13.2, 4.6 Hz, 1H), 2.88 – 2.70 (m, 2H), 2.55-2.23 (m, 11H), 2.18 (s, 3H), 2.12 – 1.98 (m, 1H), 1.88 – 1.74 (m, 1H). **^{13}C NMR** (75 MHz, $CDCl_3$) δ 207.45 , 140.05 , 137.57 , 127.58 , 119.64 , 96.95 , 80.97 , 40.93 , 36.60 (t, *J* = 21.1 Hz), 30.04 , 29.49 , 24.99 (t, *J* = 3.2 Hz), 21.22 , 20.87 . **^{19}F NMR** (282 MHz, $CDCl_3$) δ -81.03 (tt, *J* = 9.7, 3.4 Hz, 3F), -112.86 – -113.59 (m, 2F), -124.44 – -124.68 (m, 2F), -125.78 – -125.98 (m, 2F). **FTIR (neat)**: $\tilde{\nu}$ = 2921, 2362, 2336, 1718, 1375, 1365, 1217, 1131, 1109, 1034, 1014 cm^{-1}. **HRMS (ESI)**: Exact mass calculated for $C_{19}H_{17}OF_9Na$ ($[M+Na]^+$): 483.1341, mass found: 483.1353.

5-((3,5-Dimethoxyphenyl)ethynyl)-7,7,8,8,9,9,10,10,10-nonafluorodecan-2-one (3j)

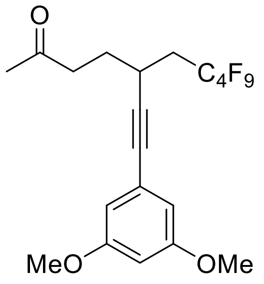

3j

3j was synthesized according to GP2 with **1j** (26 mg, 0.100 mmol), LiHMDS (0.12 mL, 0.120 mmol, 1.0 M in THF solution), LiOH (1 mg, 0.042 mmol), DABCO (17 mg, 0.152 mmol), and **2a** (31 μL, 0.180 mmol) in 1.25 mL of DME under visible-light at 50 ºC for 18 h. Purification by flash column chromatography (pentane/ethyl ether = 6/1) provided **3j** as a liquid (27 mg, 57%); **^{1}H NMR** (300 MHz, $CDCl_3$) δ 6.54 (d, *J* = 2.4 Hz, 2H), 6.43 (t, *J* = 2.3 Hz, 1H), 3.78 (s, 6H), 3.08 (dt, *J* = 7.9, 5.0 Hz, 1H), 2.80 – 2.64 (m, 2H), 2.57 – 2.22 (m, 2H), 2.19 (s, 3H), 2.11 – 1.95 (m, 1H), 1.87 – 1.75 (m, 1H). **^{13}C NMR** (75 MHz, $CDCl_3$) δ 207.47 , 160.55 , 124.22 , 109.50 , 101.53 , 88.86 , 83.26 , 55.39 , 40.87 , 36.13 (t, *J* = 21.2 Hz), 30.11 , 29.03 , 24.67 (t, *J* = 2.9 Hz). **^{19}F NMR** (282 MHz, $CDCl_3$) δ -81.02 (tt, *J* = 9.7, 3.3 Hz, 3F), -113.29 – -113.57 (m, 2F), -124.34 – -124.66 (m, 2F), -125.75 – -125.96 (m, 2F). **FTIR (neat)**: $\tilde{\nu}$ = 2945, 1720, 1590, 1454, 1422, 1355, 1233, 1207, 1157, 1134, 1066 cm^{-1}. **HRMS (ESI)**: Exact mass calculated for $C_{20}H_{19}O_3F_9Na$ ($[M+Na]^+$): 501.1083, mass found: 501.1098.

7,7,8,8,9,9,10,10,10-Nonafluoro-5-(naphthalen-1-ylethynyl)decan-2-one (3k)

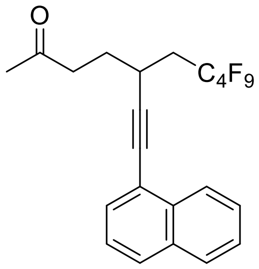

3k

3k was synthesized according to GP2 with **1k** (25 mg, 0.100 mmol), LiHMDS (0.12 mL, 0.120 mmol, 1.0 M in THF solution), LiOH (1 mg, 0.042 mmol), DABCO (17 mg, 0.152 mmol), and **2a** (31 μL, 0.180 mmol) in 1.25 mL of DME under visible-light at 50 ºC for 18 h. Purification by flash column chromatography (pentane/ethyl ether = 10/1) provided **3k** as a liquid (33 mg, 71%); **^{1}H NMR** (300 MHz, $CDCl_3$) δ 8.28 – 8.20 (m, 1H), 7.90 – 7.79 (m, 2H), 7.64 (dd, J = 7.2, 1.2 Hz, 1H), 7.61 – 7.48 (m, 2H), 7.42 (dd, J = 8.3, 7.1 Hz, 1H), 3.27 (ddd, J = 10.1, 5.0, 3.5 Hz, 1H), 2.92 – 2.74 (m, 2H), 2.65 – 2.32 (m, 2H), 2.21 (s, 3H), 2.16 – 2.07 (m, 1H), 1.98 – 1.84 (m, 1H). **^{13}C NMR** (75 MHz, $CDCl_3$) δ 207.44 , 133.33 , 133.18 , 130.47 , 128.66 , 128.31 , 126.77 , 126.38 , 125.86 , 125.15 , 120.56 , 94.17 , 81.45 , 40.95 , 36.37 (t, J = 21.1 Hz), 30.11 , 29.27 , 25.01 (t, J = 3.0 Hz). **^{19}F NMR** (282 MHz, $CDCl_3$) δ -80.91 – -81.09 (m, 3F), -112.99 – -113.43 (m, 2F), -124.26 – -124.61 (m, 2F), -125.64 – -126.01 (m, 2F). **FTIR (neat)**: $\tilde{\nu}$ = 3063, 1718, 1508, 1397, 1355, 1219, 1165, 1132, 1017 cm^{-1}. **HRMS (ESI)**: Exact mass calculated for $C_{22}H_{17}OF_9Na$ ($[M+Na]^+$): 491.1028, mass found: 491.1030.

7,7,8,8,9,9,10,10,10-Nonafluoro-5-(pyridin-2-ylethynyl)decan-2-one (3l)

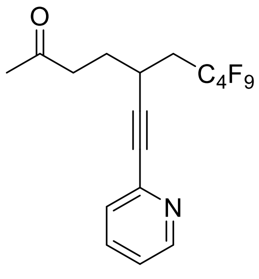

3l

3l was synthesized according to GP2 with **1l** (20 mg, 0.099 mmol), LiHMDS (0.12 mL, 0.120 mmol, 1.0 M in THF solution), LiOH (1 mg, 0.042 mmol), DABCO (17 mg, 0.152 mmol), and **2a** (31 μL, 0.180 mmol) in 1.25 mL of DME under visible-light at 50 ºC for 18 h. Purification by flash column chromatography (pentane/ethyl ether = 1/3) provided **3l** as a liquid (25 mg, 60%); **^{1}H NMR** (300 MHz, $CDCl_3$) δ 8.56 (ddd, J = 4.9, 1.9, 1.0 Hz, 1H), 7.64 (td, J = 7.7, 1.8 Hz, 1H), 7.39 (dt, J = 7.8, 1.1 Hz, 1H), 7.22 (ddd, J = 7.6, 4.9, 1.2 Hz, 1H), 3.13 (ddd, J = 10.0, 8.0, 5.0 Hz, 1H), 2.87 – 2.67 (m, 2H), 2.63 – 2.25 (m, 2H), 2.18 (s, 3H), 2.13 – 1.98 (m, 1H), 1.93 – 1.77 (m, 1H). **^{13}C NMR** (75 MHz, $CDCl_3$) δ 207.32 , 149.92 , 143.04 , 136.15 , 127.18 , 122.86 , 89.53 , 82.83 , 40.81 , 35.91 (t, J = 21.1 Hz), 30.06 , 28.76 , 24.59 (t, J = 3.1 Hz). **^{19}F NMR** (282 MHz, $CDCl_3$) δ -81.03 (tt, J = 9.8, 3.3 Hz, 3F), -113.29 – -113.57 (m, 2F), -124.31 – -124.58 (m, 2F), -125.73 – -126.00 (m, 2F). **FTIR (neat)**: $\tilde{\nu}$ = 2931, 1716, 1583, 1564, 1466, 1429, 1356, 1217, 1165, 1131, 1016 cm^{-1}. **HRMS (ESI)**: Exact mass calculated for $C_{17}H_{14}NOF_9Na$ ($[M+Na]^+$): 442.0824, mass found: 442.0823.

5-(Cyclohexylethynyl)-7,7,8,8,9,9,10,10,10-nonafluorodecan-2-one (3m)

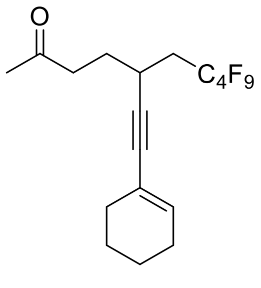

3m

3m was synthesized according to GP2 with **1m** (21 mg, 0.103 mmol), LiHMDS (0.12 mL, 0.120 mmol, 1.0 M in THF solution), LiOH (1 mg, 0.042 mmol), DABCO (17 mg, 0.152 mmol), and **2a** (31 μL, 0.180 mmol) in 1.25 mL of DME under visible-light at 50 °C for 18 h. Purification by flash column chromatography (pentane/ethyl ether = 10/1) provided **3m** as a liquid (19 mg, 44%); **^{1}H NMR** (300 MHz, $CDCl_3$) δ 6.04 (p, J = 2.1 Hz, 1H), 2.96 (td, J = 10.0, 9.3, 5.0 Hz, 1H), 2.77 – 2.56 (m, 2H), 2.49 – 1.86 (m, 11H), 1.77 – 1.52 (m, 6H). **^{13}C NMR** (75 MHz, $CDCl_3$) δ 207.61 , 134.45 , 120.39 , 86.42 , 85.08 , 40.93 , 36.31 (t, J = 21.1 Hz), 30.07 , 29.29 , 29.19 , 25.54 , 24.59 (t, J = 3.0 Hz), 22.28 , 21.49 . **^{19}F NMR** (282 MHz, $CDCl_3$) δ -81.04 (tt, J = 9.7, 3.3 Hz, 3F), -113.33 – -113.66 (m, 2F), -124.40 – -124.64 (m, 2F), -125.75 – -126.00 (m, 2F). **FTIR (neat)**: $\tilde{\nu}$ = 2934, 2364, 2335, 1718, 1437, 1355, 1217, 1164, 1131, 1108, 1022 cm^{-1}. **HRMS (ESI)**: Exact mass calculated for $C_{18}H_{19}OF_9Na$ ($[M+Na]^+$): 445.1184, mass found: 445.1188.

5-(2,2,3,3,4,4,5,5,5-Nonafluoropentyl)undec-6-yn-2-one (3n)

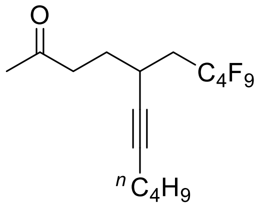

3n

3n was synthesized according to GP2 with **1n** (18 mg, 0.100 mmol), LiHMDS (0.12 mL, 0.120 mmol, 1.0 M in THF solution), LiOH (1 mg, 0.042 mmol), DABCO (17 mg, 0.152 mmol), and **2a** (31 μL, 0.180 mmol) in 1.25 mL of DME under visible-light at 50 °C for 18 h. Purification by flash column chromatography (pentane/ethyl ether = 10/1) provided **3n** as a liquid (16 mg, 40%); **^{1}H NMR** (300 MHz, $CDCl_3$) δ 2.81 (ddd, J = 7.1, 4.8, 2.4 Hz, 1H), 2.65 (dt, J = 8.2, 6.3 Hz, 2H), 2.38 – 2.21 (m, 1H), 2.20 – 2.11 (m, 5H), 1.97 – 1.83 (m, 1H), 1.72 – 1.32 (m, 6H), 0.90 (t, J = 7.2 Hz, 3H). **^{13}C NMR** (75 MHz, $CDCl_3$) δ 207.72 , 83.43 , 79.82 , 40.97 , 36.47 (t, J = 20.8 Hz), 30.89 , 30.03 , 29.37 , 24.15 (t, J = 3.1 Hz), 21.85 , 18.28 , 13.52 . **^{19}F NMR** (282 MHz, $CDCl_3$) δ -81.05 (tt, J = 9.7, 3.4 Hz, 3F), -113.37 – -113.67 (m, 2F), -124.47 – -124.72 (m, 2F), -125.73 – -126.03 (m, 2F). **FTIR (neat)**: $\tilde{\nu}$ = 2960, 1720, 1437, 1356, 1218, 1203, 1165, 1132, 1109 cm^{-1}. **HRMS (ESI)**: Exact mass calculated for $C_{17}H_{14}NOF_9Na$ ($[M+Na]^+$): 421.1184, mass found: 421.1195.

5-(2,2,3,3,4,4,5,5,5-Nonafluoropentyl)undec-6-yn-2-one (3o)

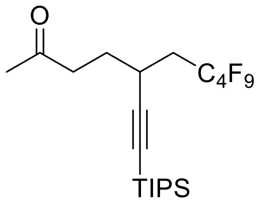

3o

3o was synthesized according to GP2 with **1o** (28 mg, 0.100 mmol), LiHMDS (0.12 mL, 0.120 mmol, 1.0 M in THF solution), LiOH (1 mg, 0.042 mmol), DABCO (17 mg, 0.152 mmol), and **2a** (31 μL, 0.180 mmol) in 1.25 mL of DME under visible-light at 50 °C for 18 h. Purification by flash column chromatography (pentane/ethyl ether = 10/1) provided **3o** as a liquid (41 mg, 82%); **^{1}H NMR** (300 MHz, $CDCl_3$) δ 2.92 (ddd, *J* = 10.3, 5.1, 3.6 Hz, 1H), 2.72 (t, *J* = 7.3 Hz, 2H), 2.47 – 2.07 (m, 5H), 2.03 – 1.90 (m, 1H), 1.72 – 1.60 (m, 1H), 1.08 – 1.03 (m, 21H). **^{13}C NMR** (75 MHz, $CDCl_3$) δ 207.42 , 107.95 , 83.78 , 40.74 , 36.49 (t, *J* = 21.1 Hz), 29.99 , 29.15 , 24.97 (t, *J* = 3.2 Hz), 18.57 , 18.49 , 11.17 . **^{19}F NMR** (282 MHz, $CDCl_3$) δ -81.08 (tt, *J* = 9.7, 3.4 Hz, 3F), -112.06 – -114.26 (m, 2F), -124.47 – -124.74 (m, 2F), -125.80 – -126.04 (m, 2F). **FTIR (neat)**: $\tilde{\nu}$ = 2944, 2893, 2867, 1721, 1464, 1356, 1234, 1133, 1018 cm^{-1}. **HRMS (ESI)**: Exact mass calculated for $C_{17}H_{14}NOF_9Na$ ($[M+Na]^+$): 521.1893, mass found: 521.1888.

10,10,11,11,12,12,13,13,13-Nonafluoro-8-(phenylethynyl)tridecan-5-one (3p)

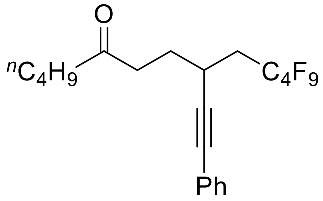

3p

3p was synthesized according to GP2 with **1p** (24 mg, 0.099 mmol), LiHMDS (0.12 mL, 0.120 mmol, 1.0 M in THF solution), LiOH (1 mg, 0.042 mmol), DABCO (17 mg, 0.152 mmol), and **2a** (31 μL, 0.180 mmol) in 1.25 mL of DME under visible-light at 50 °C for 18 h. Purification by flash column chromatography (pentane/ethyl ether = 30/1) provided **3p** as a liquid (29 mg, 64%); **^{1}H NMR** (300 MHz, $CDCl_3$) δ 7.43 – 7.36 (m, 2H), 7.30 (dt, *J* = 5.0, 2.0 Hz, 3H), 3.10 (dt, *J* = 8.0, 5.0 Hz, 1H), 2.71 (dd, *J* = 8.0, 6.5 Hz, 2H), 2.53 – 2.22 (m, 4H), 2.12 – 1.95 (m, 1H), 1.88 – 1.72 (m, 1H), 1.63 – 1.52 (m, 2H), 1.38 – 1.27 (m, 2H), 0.90 (t, *J* = 7.3 Hz, 3H). **^{13}C NMR** (75 MHz, $CDCl_3$) δ 209.97 , 131.59 , 128.26 , 128.15 , 123.00 , 89.36 , 83.28 , 42.78 , 39.87 , 36.23 (t, *J* = 21.0 Hz), 29.12 , 26.00 , 24.74 (t, *J* = 3.1 Hz), 22.34 , 13.79 . **^{19}F NMR** (282 MHz, $CDCl_3$) δ -80.93 – -81.11 (m, 3F), -113.22 – -113.54 (m, 2F), -124.29 – -124.63 (m, 2F), -125.68 – -126.01 (m, 2F). **FTIR (neat)**: $\tilde{\nu}$ = 2964, 1715, 1599, 1491, 1444, 1413, 1380, 1355, 1220, 1169, 1132, 1070, 1019 cm^{-1}. **HRMS (ESI)**: Exact mass calculated for $C_{21}H_{21}OF_9Na$ ($[M+Na]^+$): 483.1341, mass found: 483.1351.

8,8,9,9,10,10,11,11,11-Nonafluoro-2-methyl-6-(phenylethynyl)undecan-3-one (3q)

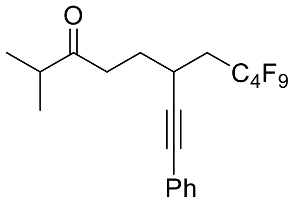

3q

3q was synthesized according to GP2 with **1q** (23 mg, 0.101 mmol), LiHMDS (0.12 mL, 0.120 mmol, 1.0 M in THF solution), LiOH (1 mg, 0.042 mmol), DABCO (17 mg, 0.152 mmol), and **2a** (31 μL, 0.180 mmol) in 1.25 mL of DME under visible-light at 50 °C for 18 h. Purification by flash column chromatography (pentane/ethyl ether = 30/1) provided **3q** as a liquid (28 mg, 62%); **^{1}H NMR** (300 MHz, $CDCl_3$) δ 7.43 – 7.35 (m, 2H), 7.34 – 7.27 (m, 3H), 3.10 (ddd, *J* = 10.3, 5.1, 3.2 Hz, 1H), 2.76 (td, *J* = 6.8, 6.1, 1.6 Hz, 2H), 2.64 (p, *J* = 6.9 Hz, 1H), 2.56 – 2.18 (m, 2H), 2.03 (ddt, *J* = 13.4, 7.6, 3.8 Hz, 1H), 1.89 – 1.70 (m, 1H), 1.13 (dd, *J* = 7.0, 3.3 Hz, 6H). **^{13}C NMR** (75 MHz, $CDCl_3$) δ 213.50 , 131.58 , 128.27 , 128.14 , 123.03 , 89.40 , 83.26 , 41.04 , 37.46 , 36.27 (t, *J* = 21.0 Hz), 29.17 , 24.72 (t, *J* = 3.0 Hz), 18.33 , 18.21 . **^{19}F NMR** (282 MHz, $CDCl_3$) δ -81.03 (tt, *J* = 9.6, 3.3 Hz, 3F), -113.30 – -113.51 (m, 2F), -124.38 – -124.59 (m, 2F), -125.75 – -125.95 (m, 2F). **FTIR (neat)**: $\tilde{\nu}$ = 2975, 1713, 1491, 1355, 1217, 1132, 1019 cm^{-1}. **HRMS (ESI)**: Exact mass calculated for $C_{21}H_{21}OF_9Na$ ($[M+Na]^+$): 469.1184, mass found: 469.1189.

6,6,7,7,8,8,9,9,9-Nonafluoro-1-phenyl-4-(phenylethynyl)nonan-1-one (3r)

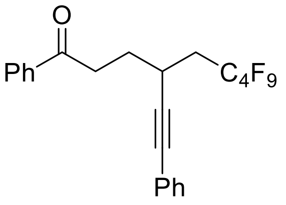

3r

3r was synthesized according to GP2 with **1r** (26 mg, 0.099 mmol), LiHMDS (0.12 mL, 0.120 mmol, 1.0 M in THF solution), LiOH (1 mg, 0.042 mmol), DABCO (17 mg, 0.152 mmol), and **2a** (31 μL, 0.180 mmol) in 1.25 mL of DME under visible-light at 50 °C for 18 h. Purification by flash column chromatography (pentane/ethyl ether = 45/1) provided **3r** as a yellow solid (42 mg, 88%); **MP**: 43 °C; **^{1}H NMR** (300 MHz, $CDCl_3$) δ 8.05 – 7.96 (m, 2H), 7.62 – 7.53 (m, 1H), 7.52 – 7.43 (m, 2H), 7.42 – 7.34 (m, 2H), 7.32 – 7.26 (m, 3H), 3.35 – 3.26 (m, 2H), 3.26 – 3.16 (m, 1H), 2.65 – 2.30 (m, 2H), 2.29 – 2.16 (m, 1H), 2.08 – 1.91 (m, 1H). **^{13}C NMR** (75 MHz, $CDCl_3$) δ 199.03 , 136.83 , 133.16 , 131.60 , 128.64 , 128.25 , 128.15 , 128.04 , 122.97 , 89.43 , 83.44 , 36.31 (t, *J* = 21.1 Hz), 35.95 , 29.70 , 24.92 . **^{19}F NMR** (282 MHz, $CDCl_3$) δ -81.01 (tt, *J* = 9.7, 3.3 Hz, 3F), -113.25 – -113.47 (m, 2F), -124.32 – -124.57 (m, 2F), -125.72 – -125.94 (m, 2F). **FTIR (neat)**: $\tilde{\nu}$ = 2951, 1680, 1599, 1491, 1451, 1354, 1282, 1229, 1196, 1129, 1102, 1013 cm^{-1}. **HRMS (ESI)**: Exact mass calculated for $C_{23}H_{17}OF_9Na$ ($[M+Na]^+$): 503.1028, mass found: 503.1037.

Chiral **3r** was synthesized according to GP2 with chiral **1r** (26 mg, 0.099 mmol), LiHMDS (0.12 mL, 0.120 mmol, 1.0 M in THF solution), LiOH (1 mg, 0.042 mmol), DABCO (17 mg, 0.152 mmol), and

2a (31 µL, 0.180 mmol) in 1.25 mL of DME under visible-light at 50 ºC for 18 h. Purification by flash column chromatography (pentane/ethyl ether = 45/1) provided chiral **3r** as a yellow solid (39 mg, 82%); **^{1}H NMR** (300 MHz, $CDCl_3$) δ 8.05 – 7.97 (m, 2H), 7.62 – 7.54 (m, 1H), 7.52 – 7.43 (m, 2H), 7.43 – 7.34 (m, 2H), 7.33 – 7.26 (m, 3H), 3.35 – 3.16 (m, 3H), 2.66 – 2.16 (m, 3H), 2.08 – 1.94 (m, 1H). The pure product was analyzed by chiral HPLC. Chiral HPLC (Chiralcel OJ-RH column with guard, 20 °C, 1 mL/min, MeCN:H_2O = 65:35); Elution time: t_{major} = 8.734 min and t_{minor} = 10.047 min, enantiomeric excess (7%).

7,7,8,8,9,9,10,10,10-Nonafluoro-5-methyl-5-(phenylethynyl)decan-2-one (3s)

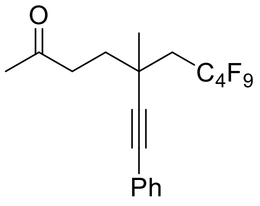

3s

3s was synthesized according to GP2 with **1s** (21 mg, 0.098 mmol), LiHMDS (0.12 mL, 0.120 mmol, 1.0 M in THF solution), LiOH (1 mg, 0.042 mmol), DABCO (17 mg, 0.152 mmol), and **2a** (31 µL, 0.180 mmol) in 1.25 mL of DME under visible-light at 50 ºC for 18 h. Purification by flash column chromatography (pentane/ethyl ether = 15/1) provided **3s** as a liquid (31 mg, 73%); **^{1}H NMR** (300 MHz, $CDCl_3$) δ 7.41 – 7.34 (m, 2H), 7.30 (dt, J = 5.1, 1.6 Hz, 3H), 2.72 (dd, J = 8.7, 7.0 Hz, 2H), 2.51 – 2.23 (m, 2H), 2.20 (s, 3H), 2.10 – 1.86 (m, 2H), 1.48 (d, J = 1.1 Hz, 3H). **^{13}C NMR** (75 MHz, $CDCl_3$) δ 207.54 , 131.54 , 128.25 , 128.11 , 123.02 , 92.25 , 83.06 , 40.24 (t, J = 20.0 Hz), 39.43 , 35.71 , 32.79 , 30.06 , 26.97 (t, J = 2.1 Hz). **^{19}F NMR** (282 MHz, $CDCl_3$) δ -81.06 (td, J = 9.7, 4.8 Hz, 3F), -110.35 – -112.84 (m, 2F), -124.41 – -124.69 (m, 2F), -125.54 – -125.77 (m, 2F). **FTIR (neat)**: $\tilde{\nu}$ = 2942, 1719, 1491, 1354, 1218, 1131, 1071, 1025 cm^{-1}. **HRMS (ESI)**: Exact mass calculated for $C_{19}H_{17}OF_9Na$ ([M+Na]$^+$): 455.1028, mass found: 455.1046.

7,7,8,8,9,9,10,10,10-Nonafluoro-6,6-dimethyl-5-(phenylethynyl)decan-2-one (3t)

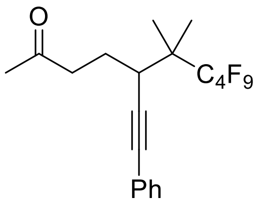

3t

3t was synthesized according to GP2 with **1t** (23 mg, 0.101 mmol), LiHMDS (0.12 mL, 0.120 mmol, 1.0 M in THF solution), LiOH (1 mg, 0.042 mmol), DABCO (17 mg, 0.152 mmol), and **2a** (31 µL, 0.180 mmol) in 1.25 mL of DME under visible-light at 50 ºC for 18 h. Purification by flash column chromatography (pentane/ethyl ether = 15/1) provided **3t** as a liquid (19 mg, 42%); **^{1}H NMR** (300 MHz, $CDCl_3$) δ 7.38 – 7.32 (m, 2H), 7.29 (dt, J = 5.1, 1.8 Hz, 3H), 2.86 – 2.60 (m, 2H), 2.41 – 2.22 (m, 1H), 2.20 – 1.98 (m, 5H), 1.52 (d, J = 2.0 Hz, 3H), 1.48 (s, 3H). **^{13}C**

NMR (75 MHz, $CDCl_3$) δ 207.17 , 131.43 , 128.26 , 127.97 , 123.22 , 95.04 , 81.73 , 48.60 – 47.83 (m), 43.66 , 35.51 (d, *J* = 2.4 Hz), 29.76 , 29.47 (d, *J* = 5.7 Hz), 26.93 – 26.61 (m), 20.97 – 20.60 (m). **^{19}F NMR** (282 MHz, $CDCl_3$) δ -80.90 (tt, *J* = 9.9, 3.5 Hz, 3F), -108.57 – -112.47 (m, 2F), -119.98 – -123.35 (m, 2F), -124.34 – -127.10 (m, 2F). **FTIR (neat)**: $\tilde{\nu}$ = 2984, 1720, 1598, 1491, 1419, 1351, 1233, 1217, 1166, 1016 cm^{-1}. **HRMS (ESI)**: Exact mass calculated for $C_{20}H_{19}OF_9Na$ ($[M+Na]^+$): 469.1184, mass found: 469.1192.

6,6,7,7,8,8,9,9,9-Nonafluoro-2,2-dimethyl-1-phenyl-4-(phenylethynyl)nonan-1-one (3u)

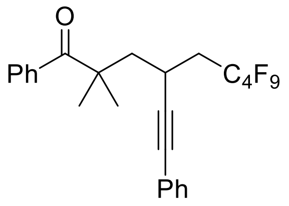

3u

3u was synthesized according to GP2 with **1u** (29 mg, 0.100 mmol), LiHMDS (0.12 mL, 0.120 mmol, 1.0 M in THF solution), LiOH (1 mg, 0.042 mmol), DABCO (17 mg, 0.152 mmol), and **2a** (31 μL, 0.180 mmol) in 1.25 mL of DME under visible-light at 50 °C for 18 h. Purification by flash column chromatography (pentane/ethyl ether = 50/1) provided **3u** as a liquid (23 mg, 57%); **^{1}H NMR** (300 MHz, $CDCl_3$) δ 7.71 – 7.64 (m, 2H), 7.43 – 7.30 (m, 3H), 7.22 (dd, *J* = 6.1, 2.4 Hz, 5H), 3.23 – 3.10 (m, 1H), 2.57 – 2.23 (m, 3H), 2.00 (dd, *J* = 13.8, 3.7 Hz, 1H), 1.48 (s, 3H), 1.46 (s, 3H). **^{13}C NMR** (75 MHz, $CDCl_3$) δ 207.83 , 138.74 , 131.39 , 130.79 , 128.07 , 128.02 , 127.99 , 123.00 , 90.42 , 83.90 , 47.58 , 45.62 , 37.24 (t, *J* = 20.8 Hz), 27.44 , 25.94 , 22.25 (t, *J* = 2.9 Hz). **^{19}F NMR** (282 MHz, $CDCl_3$) δ -81.01 (tt, *J* = 9.7, 3.3 Hz, 3F), -113.08 – -113.54 (m, 2F), -124.35 – -124.76 (m, 2F), -125.60 – -126.06 (m, 2F). **FTIR (neat)**: $\tilde{\nu}$ = 2971, 1674, 1491, 1353, 1217, 1131, 1019 cm^{-1}. **HRMS (ESI)**: Exact mass calculated for $C_{25}H_{21}OF_9Na$ ($[M+Na]^+$): 531.1341, mass found: 531.1342.

7-Phenyl-5-(2,2,2-trifluoroethyl)hept-6-yn-2-one (3v)

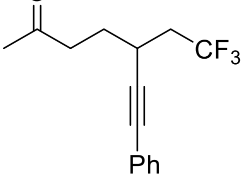

3v

3v was synthesized according to GP2 with **1a** (20 mg, 0.100 mmol), LiHMDS (0.12 mL, 0.120 mmol, 1.0 M in THF solution), LiOH (1 mg, 0.042 mmol), DABCO (17 mg, 0.152 mmol), and **2b** (60 μL, 0.180 mmol, 0.588 g/mL in DME) in 1.25 mL of DME under visible-light at 50 °C for 8 h. Then second part of **2b (**60 μL, 0.180 mmol, 0.588 g/mL in DME**)**was added and the reaction was reacted for another 16 h. Purification by flash column chromatography (pentane/ethyl ether = 10/1) provided **3v** as a liquid (11 mg, 41%); **^{1}H NMR** (300

MHz, $CDCl_3$) δ 7.42 – 7.36 (m, 2H), 7.34 – 7.27 (m, 3H), 3.05 – 2.93 (m, 1H), 2.83 – 2.63 (m, 2H), 2.52 – 2.39 (m, 1H), 2.37 – 2.25 (m, 1H), 2.19 (s, 3H), 2.06 – 1.94 (m, 1H), 1.83 – 1.73 (m, 1H). **^{13}C NMR** (75 MHz, $CDCl_3$) δ 207.51, 131.59, 128.27, 128.16, 126.54 (q, J = 117.8 Hz), 122.99, 88.95, 83.42, 40.87, 39.34 (q, J = 27.9 Hz), 30.10, 28.51, 26.96 (q, J = 3.0 Hz). **^{19}F NMR** (282 MHz, $CDCl_3$) δ -64.12. **FTIR (neat)**: $\tilde{\nu}$ = 2929, 1718, 1491, 1381, 1255, 1164, 1140, 1111 cm^{-1}. **HRMS (ESI)**: Exact mass calculated for $C_{15}H_{15}OF_3Na$ ($[M+Na]^+$): 291.0966, mass found: 291.0967.

7,7,8,8,9,9,10,10,11,11,12,12,12-Tridecafluoro-5-(phenylethynyl)dodecan-2-one (3w)

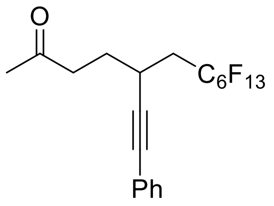

3w

3w was synthesized according to GP2 with **1a** (20 mg, 0.100 mmol), LiHMDS (0.12 mL, 0.120 mmol, 1.0 M in THF solution), LiOH (1 mg, 0.042 mmol), DABCO (17 mg, 0.152 mmol), and **2c** (39 μL, 0.180 mmol) in 1.25 mL of DME under visible-light at 50 °C for 18 h. Purification by flash column chromatography (pentane/ethyl ether = 10/1) provided **3w** as a liquid (32 mg, 62%); **^{1}H NMR** (300 MHz, $CDCl_3$) δ 7.42 – 7.37 (m, 2H), 7.33 – 7.27 (m, 3H), 3.10 (ddt, J = 9.9, 8.0, 5.0 Hz, 1H), 2.84 – 2.65 (m, 2H), 2.59 – 2.23 (m, 2H), 2.19 (s, 3H), 2.03 (dtd, J = 15.3, 7.3, 4.5 Hz, 1H), 1.81 (dtd, J = 10.1, 7.6, 3.9 Hz, 1H). **^{13}C NMR** (75 MHz, $CDCl_3$) δ 207.45 , 131.58 , 128.27 , 128.18 , 122.95 , 89.26 , 83.32 , 40.88 , 36.29 (t, J = 21.0 Hz), 30.08 , 29.09 , 24.73 (t, J = 2.9 Hz). **^{19}F NMR** (282 MHz, $CDCl_3$) δ -80.80 (tt, J = 10.0, 5.0 Hz, 3F), -113.09 – -113.30 (m, 2F), -121.58 – -121.93 (m, 2F), -122.66 – -123.00 (m, 2F), -123.42 – -123.69 (m, 2F), -125.97 – -126.23 (m, 2F). **FTIR (neat)**: $\tilde{\nu}$ = 2954, 1719, 1491, 1444, 1360, 1318, 1235, 1190, 1164, 1144, 1122, 1049, 1027 cm^{-1}. **HRMS (ESI)**: Exact mass calculated for $C_{20}H_{15}OF_{13}Na$ ($[M+Na]^+$): 541.0808, mass found: 541.0819.

7,7,8,8,9,9,10,10,11,11,12,12,13,13,14,14,14-Heptadecafluoro-5-(phenylethynyl)tetradecan-2-one (3x)

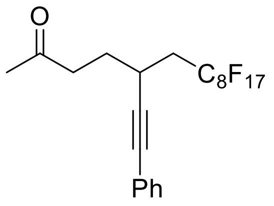

3x

3x was synthesized according to GP2 with **1a** (20 mg, 0.100 mmol), LiHMDS (0.12 mL, 0.120 mmol, 1.0 M in THF solution), LiOH (1 mg, 0.042 mmol), DABCO (17 mg, 0.152 mmol), and **2d** (48 μL, 0.182 mmol) in 1.25 mL of DME under visible-light at 50 °C for 18 h. Purification by

flash column chromatography (pentane/ethyl ether = 10/1) provided **3x** as a yellow solid (34 mg, 55%); **MP**: 39 °C; **^{1}H NMR** (300 MHz, $CDCl_3$) δ 7.43 – 7.35 (m, 2H), 7.30 (dt, *J* = 3.8, 2.7 Hz, 3H), 3.09 (ddd, *J* = 9.9, 8.0, 4.9 Hz, 1H), 2.84 – 2.64 (m, 2H), 2.53 – 2.23 (m, 2H), 2.19 (s, 3H), 2.11 – 1.97 (m, 1H), 1.88 – 1.75 (m, 1H). **^{13}C NMR** (75 MHz, $CDCl_3$) δ 207.45 , 131.59 , 128.27 , 128.18 , 122.96 , 89.27 , 83.32 , 40.89 , 36.30 (t, *J* = 21.0 Hz), 30.08 , 29.09 , 24.73 (t, *J* = 3.1 Hz). **^{19}F NMR** (282 MHz, $CDCl_3$) δ -80.79 (t, *J* = 9.8 Hz, 3F), -113.18 (t, *J* = 14.5 Hz, 2F), -121.37 – -121.68 (m, 2F), -121.70 – -122.04 (m, 4F), -122.45 – -122.90 (m, 2F), -123.30 – -123.72 (m, 2F), -125.90 – -126.26 (m, 2F). **FTIR (neat)**: $\tilde{\nu}$ = 2944, 1718, 1600, 191, 1443, 1370, 1329, 1236, 1200, 1145, 1134, 1113, 1027 cm^{-1}. **HRMS (ESI)**: Exact mass calculated for $C_{22}H_{15}OF_{17}Na$ ($[M+Na]^+$): 641.0744, mass found: 641.0740.

7,7,8,8,9,9,10,10,11,11,12,12,13,13,14,14,15,15,16,16,16-Henicosafluoro-5-(phenylethynyl)hexadecan-2-one (3y)

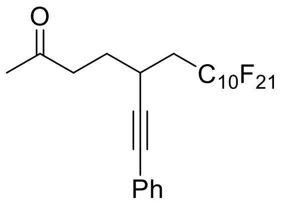

3y

3y was synthesized according to GP2 with **1a** (20 mg, 0.100 mmol), LiHMDS (0.12 mL, 0.120 mmol, 1.0 M in THF solution), LiOH (1 mg, 0.042 mmol), DABCO (17 mg, 0.152 mmol), and **2e** (116 mg, 0.180 mmol) in 1.25 mL of DME under visible-light at 50 °C for 18 h. Purification by flash column chromatography (pentane/ethyl ether = 10/1) provided **3y** as a white solid (34 mg, 47%); **MP**: 69 °C; **^{1}H NMR** (300 MHz, $CDCl_3$) δ 7.43 – 7.35 (m, 2H), 7.34 – 7.27 (m, 3H), 3.10 (ddt, *J* = 9.9, 8.0, 4.9 Hz, 1H), 2.86 – 2.64 (m, 2H), 2.60 – 2.23 (m, 2H), 2.19 (s, 3H), 2.11 – 1.96 (m, 1H), 1.90 – 1.72 (m, 1H). **^{13}C NMR** (75 MHz, $CDCl_3$) δ 207.46 , 131.59 , 128.28 , 128.18 , 122.96 , 89.27 , 83.32 , 40.89 , 36.31 (t, *J* = 21.0 Hz), 30.09 , 29.10 , 24.74 (t, *J* = 3.0 Hz). **^{19}F NMR** (282 MHz, $CDCl_3$) δ -80.71 – -80.84 (m, 3F), -113.08 – -113.30 (m, 2F), -121.31 – -122.07 (m, 10F), -122.68 (brs, 2F), -123.51 (brs, 2F), -125.92 – -126.24 (m, 2F). **FTIR (neat)**: $\tilde{\nu}$ = 2962, 1718, 1491, 1444, 1372, 1211, 1150, 1110, 1053 cm^{-1}. **HRMS (ESI)**: Exact mass calculated for $C_{24}H_{15}OF_{21}Na$ ($[M+Na]^+$): 741.0680, mass found: 741.0680.

8-Chloro-7,7,8,8-tetrafluoro-5-(phenylethynyl)octan-2-one (3z)

3z was synthesized according to GP2 with **1a** (20 mg, 0.100 mmol), LiHMDS (0.12 mL, 0.120 mmol, 1.0 M in THF solution), LiOH (1 mg, 0.042 mmol), DABCO (17 mg, 0.152 mmol), and **2f** (47 mg, 0.179 mmol) in 1.25 mL of DME under visible-light at 50 °C for 18 h. Purification by flash column

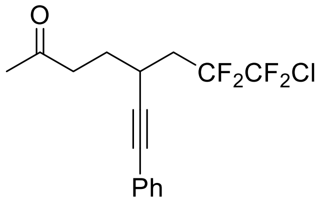

3z

chromatography (pentane/ethyl ether = 10/1) provided **3z** as a liquid (27 mg, 81%); **^{1}H NMR** (300 MHz, $CDCl_3$) δ 7.43 – 7.36 (m, 2H), 7.34 – 7.27 (m, 3H), 3.08 (ddt, *J* = 10.0, 8.0, 5.0 Hz, 1H), 2.85 – 2.63 (m, 2H), 2.60 – 2.23 (m, 2H), 2.19 (s, 3H), 2.11 – 1.96 (m, 1H), 1.88 – 1.76 (m, 1H). **^{13}C NMR** (75 MHz, $CDCl_3$) δ 207.51 , 131.58 , 128.26 , 128.14 , 123.00 , 89.39 , 83.28 , 40.91 , 36.02 (t, *J* = 21.4 Hz), 30.09 , 29.03 , 25.08 (t, *J* = 2.8 Hz). **^{19}F NMR** (282 MHz, $CDCl_3$) δ -71.45 (t, *J* = 2.3 Hz, 2F), -113.02 – -113.09 (m, 2F). **FTIR (neat)**: $\tilde{\nu}$ = 2958, 1717, 1599, 1419, 1443, 1374, 1252, 1212, 1148, 1094, 1050, 1026 cm^{-1}. **HRMS (ESI)**: Exact mass calculated for $C_{16}H_{15}O^{35}ClF_4Na$ ($[M+Na]^+$): 357.0640, mass found: 357.0650.

8,8,9,9,10,10,11,11,11-Nonafluoro-6-(phenylethynyl)undecan-2-one (3va)

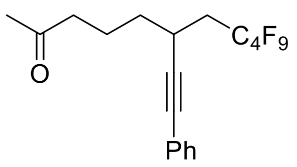

3va

3va was synthesized according to GP2 with **1v** (21 mg, 0.098 mmol), LiHMDS (0.12 mL, 0.120 mmol, 1.0 M in THF solution), LiOH (1 mg, 0.042 mmol), DABCO (17 mg, 0.152 mmol), and **2a** (31 μL, 0.180 mmol) in 1.25 mL of DME under visible-light at 50 °C for 18 h. Purification by flash column chromatography (pentane/ethyl ether = 10/1) provided **3va** as a liquid (28 mg, 66%); **^{1}H NMR** (300 MHz, $CDCl_3$) δ 7.43 – 7.35 (m, 2H), 7.29 (ddd, *J* = 3.4, 2.5, 1.5 Hz, 3H), 3.06 (tt, *J* = 8.3, 5.3 Hz, 1H), 2.57 – 2.21 (m, 4H), 2.16 (s, 3H), 1.96 – 1.86 (m, 1H), 1.86 – 1.72 (m, 1H), 1.71 – 1.55 (m, 2H). **^{13}C NMR** (75 MHz, $CDCl_3$) δ 208.11 , 131.60 , 128.23 , 128.03 , 123.15 , 89.79 , 82.88 , 43.01 , 35.96 (t, *J* = 21.0 Hz), 34.69 , 29.87 , 25.15 (t, *J* = 3.2 Hz), 21.15 . **^{19}F NMR** (282 MHz, $CDCl_3$) δ -81.03 (tt, *J* = 9.7, 3.3 Hz, 3F), -113.34 – -113.61 (m, 2F), -124.36 – -124.60 (m, 2F), -125.73 – -125.95 (m, 2F). **FTIR (neat)**: $\tilde{\nu}$ = 2942, 1718, 1599, 1491, 1444, 1356, 1222, 1167, 1133 cm^{-1}. **HRMS (ESI)**: Exact mass calculated for $C_{19}H_{17}OF_9Na$ ($[M+Na]^+$): 455.1028, mass found: 455.1030.

4. Follow-up chemistry

7,7,8,8,9,9,10,10,10-nonafluoro-5-(phenylethynyl)decan-2-ol (4)

To a flame-dried Schlenk tube equipped with a magnetic stir bar $LiAlH_4$ (0.050 mmol, 1 M in THF, 0.050 mL), THF (1 mL) and **3a** (21 mg, 0.050 mmol) were added sequentially under Ar atmosphere. The mixture was allowed to stir at 80 °C for 3 h. After that, the reaction was quenched with 5 mL of H_2O at 0 °C. Then 5 mL of saturated potassium sodium tartrate solution was added into this mixture.

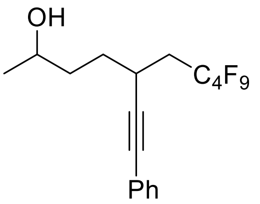

4, d.r. = 1:1

The mixture was extracted with 5 mL of ethyl ether three times. The organic phase was combined, washed with brine and dried over Na_2SO_4. The solvent was then removed and the residue was purified by flash column chromatography (pentane/ethyl acetate = 10/1) to provide **4** as a liquid (16 mg, 76%, d.r. = 1:1); **^{1}H NMR** (600 MHz, C_6D_6) δ 7.46 – 7.40 (m, 2H), 7.00 – 6.94 (m, 3H), 3.47 – 3.41 (m, 1H), 2.92 – 2.84 (m, 1H), 2.26 – 2.15 (m, 1H), 1.99 – 1.90 (m, 1H), 1.56 – 1.46 (m, 2H), 1.38 – 1.30 (m, 2H), 0.93 (d, J = 6.2 Hz, 1.46H), & 0.93 (d, J = 6.1 Hz, 1.49H), 0.65 (s, 1H). **^{13}C NMR** (75 MHz, C_6D_6) δ 131.89, 128.54, 128.19, 123.84, 90.61 & 90.48, 83.46 & 83.40, 67.45 & 66.94, 36.81 & 36.50, 36.21 , 36.72 – 35.92 (m), 32.09 & 31.76, 25.67 (t, J = 3.3 Hz) & 25.34 (t, J = 2.8 Hz), 23.86 & 23.77. **^{19}F NMR** (282 MHz, C_6D_6) δ -81.06 – -81.21 (m), -112.01 – -114.47 (m), -124.15 – -124.41 (m), -125.72 – -125.92 (m). **FTIR (neat)**: $\tilde{\nu}$ = 3349, 2969, 2928, 1355, 1217, 1131, 1070, 1016 cm^{-1}. **HRMS (ESI)**: Exact mass calculated for $C_{18}H_{17}OF_9Na$ ($[M+Na]^+$): 443.1028, mass found: 443.1002.

7,7,8,8,9,9,10,10,10-Nonafluoro-5-phenethyldecan-2-one (5)

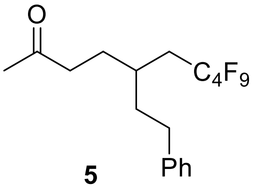

5

To a flame-dried Schlenk tube equipped with a magnetic stir bar Palladium 5% on activated carbon (5 mg, 0.0023 mmol), **3a** (21 mg, 0.050 mmol), and MeOH (1 mL) were added sequentially under H_2 atmosphere. The mixture was allowed to stir at room temperature with a H_2 balloon for 23 h. After that, the solvent was then removed and the residue was purified by flash column chromatography (pentane/ethyl ether = 20/1) to provide **5** as a liquid (17 mg, 80%); **^{1}H NMR** (600 MHz, $CDCl_3$) δ7.26 – 7.17 (m, 2H), 7.16 – 7.06 (m, 3H), 2.61 – 2.50 (m, 2H), 2.37 (t, J = 7.7 Hz, 2H), 2.08 (s, 3H), 2.05 – 1.79 (m, 3H), 1.74 – 1.59 (m, 4H). **^{13}C NMR** (75 MHz, $CDCl_3$) δ 141.55, 128.47, 128.26, 126.01, 40.44, 36.09, 33.85 (t, J = 21.0 Hz), 32.54, 30.56, 29.92, 27.80. **^{19}F NMR** (282 MHz, $CDCl_3$) δ -81.05 (tt, J = 9.7, 3.3 Hz), -112.94 – -113.11 (m), -124.33 – -124.51 (m), -125.72 – -125.91 (m). **FTIR (neat)**: $\tilde{\nu}$ = 2923, 1719, 1497, 1455, 1356, 1217, 1131, 1046, 1022 cm^{-1}. **HRMS (ESI)**: Exact mass calculated for $C_{18}H_{19}OF_9Na$ ($[M+Na]^+$): 445.1184, mass found: 45.1188.

(2-Methyl-5-(2,2,3,3,4,4,5,5,5-nonafluoropentyl)cyclopent-1-en-1-yl)(phenyl)methanone (6)

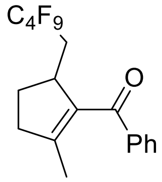

6

To a flame-dried Schlenk tube equipped with a magnetic stir bar **3a** (21 mg, 0.050 mmol), MeOH (1 mL) and TfOH (0.2 μL, 0.002 mmol) were added sequentially under Ar atmosphere. The mixture was allowed to stir at 80 °C for 5 h. After that, the solvent was removed and the residue was purified by flash column chromatography (pentane/ethyl acetate = 80/1) provided **6** as a liquid (16 mg, 76%);[3] **^{1}H NMR** (600 MHz, C_6D_6) δ 7.71 – 7.65 (m, 2H), 7.13 – 7.03 (m, 3H), 3.68 – 3.51 (m, 1H), 2.93 – 2.66 (m, 1H), 2.08 – 1.75 (m, 4H), 1.51 – 1.39 (m, 1H), 1.23 (s, 3H). **^{13}C NMR** (75 MHz, C_6D_6) δ 195.25, 149.77, 139.64, 137.59, 132.55, 129.04, 128.66, 41.66, 38.74, 34.27 (t, J = 20.8 Hz), 29.61, 16.69. **^{19}F NMR** (282 MHz, C_6D_6) δ -80.88 – -81.24 (m), -110.49 – -114.70 (m), -124.08 – -124.43 (m), -125.55 – -125.84 (m). **FTIR (neat)**: $\tilde{\nu}$ = 2935, 1640, 1349, 1216, 1131, 1101, 1017 cm^{-1}. **HRMS (ESI)**: Exact mass calculated for $C_{18}H_{15}OF_9Na$ ($[M+Na]^+$): 441.0871, mass found: 441.0847.

(5-Methyl-2-(2,2,3,3,4,4,5,5,5-nonafluoropentyl)cyclopent-1-en-1-yl)(phenyl)methanone (6)

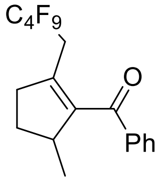

6

To a flame-dried Schlenk tube equipped with a magnetic stir bar $AuCl_3$ (0.6 mg, 0.002 mmol), $AgSbF_6$ (2.0 mg, 0.006 mmol), **3a** (42 mg, 0.100 mmol), and toluene (1 mL) were added sequentially under Ar atmosphere. The mixture was allowed to stir at 100 °C for 18 h. After that, 5 mL of H_2O was added to the mixture and then the aqueous phase was extracted with 5 mL of ethyl acetate three times. The combined organic phase was washed with brine and dried over Na_2SO_4. The solvent was then removed and the residue was purified by flash column chromatography (pentane/ethyl acetate = 350/1) provided **6** as a liquid (25 mg, 60%);[4] **^{1}H NMR** (300 MHz, $CDCl_3$) δ 7.81 – 7.72 (m, 2H), 7.60 – 7.52 (m, 1H), 7.50 – 7.42 (m, 2H), 3.70 – 3.54 (m, 1H), 2.70 – 2.44 (m, 3H), 2.40 – 2.30 (m, 1H), 2.03 – 1.86 (m, 1H), 1.85 – 1.73 (m, 1H), 1.62 (d, 7H). **^{13}C NMR** (75 MHz, $CDCl_3$) δ 196.50, 150.82, 139.00, 137.49, 132.84, 128.87, 128.61, 41.38, 39.06, 34.04 (t, J = 21.0 Hz), 29.62, 16.98. **^{19}F NMR** (282 MHz, $CDCl_3$) δ -80.98 – -81.24 (m), -111.01 – -115.27 (m), -124.37 – -124.58 (m), -125.79 – -126.05 (m).

5. References:

(1) G. A. Phillips, C. Palmer, A. C. Stevens, M. L. Piotrowski, D. S. R. Dekruyf, B. L. Pagenkopf, *Tetrahedron Letter* **2015**, *56*, 6052-6055.

(2) P. G. Cozzi, S. Alesi, *Chem. Commun.* **2004**, 2448-2449.

(3) T. Jin, F. Yang, C. Liu, Y. Yamamoto, *Chem. Commun*. **2009**, 3533-3535.
(4) T. Jin, Y. Yamamoto, *Org. Lett.* **2007**, *9*, 5259-5262.

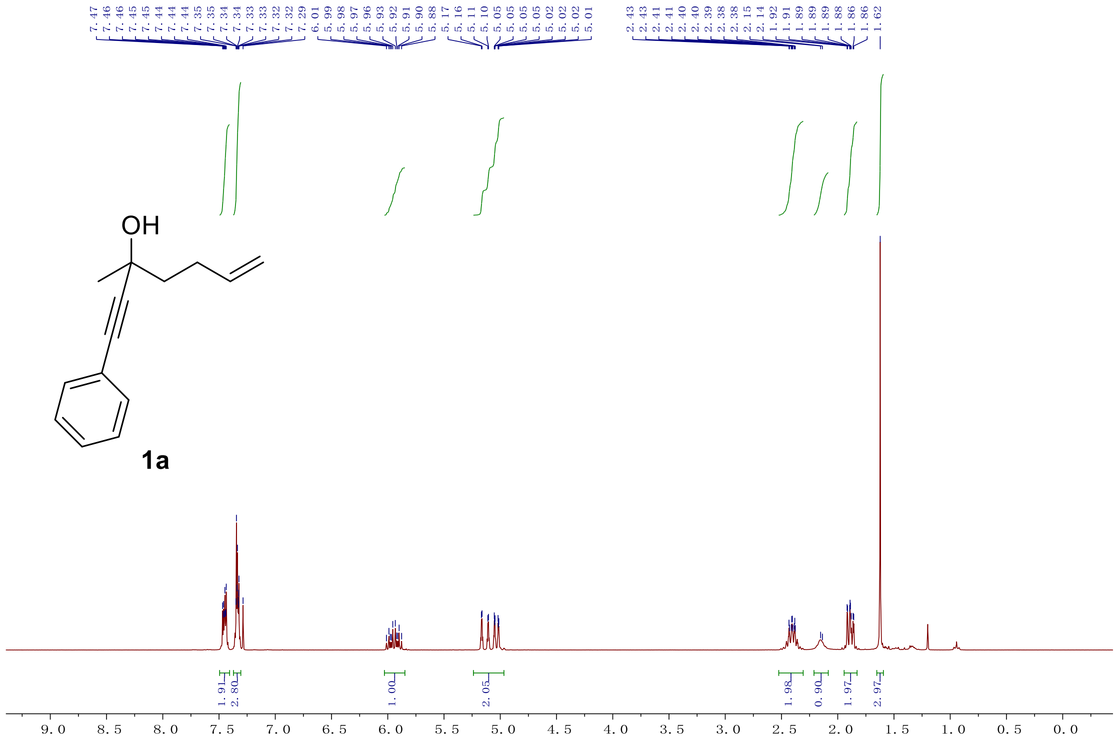

Dec20-2016.460.fid — stu tang txj 503 — proton CDCl3 /opt/topspin av1 22

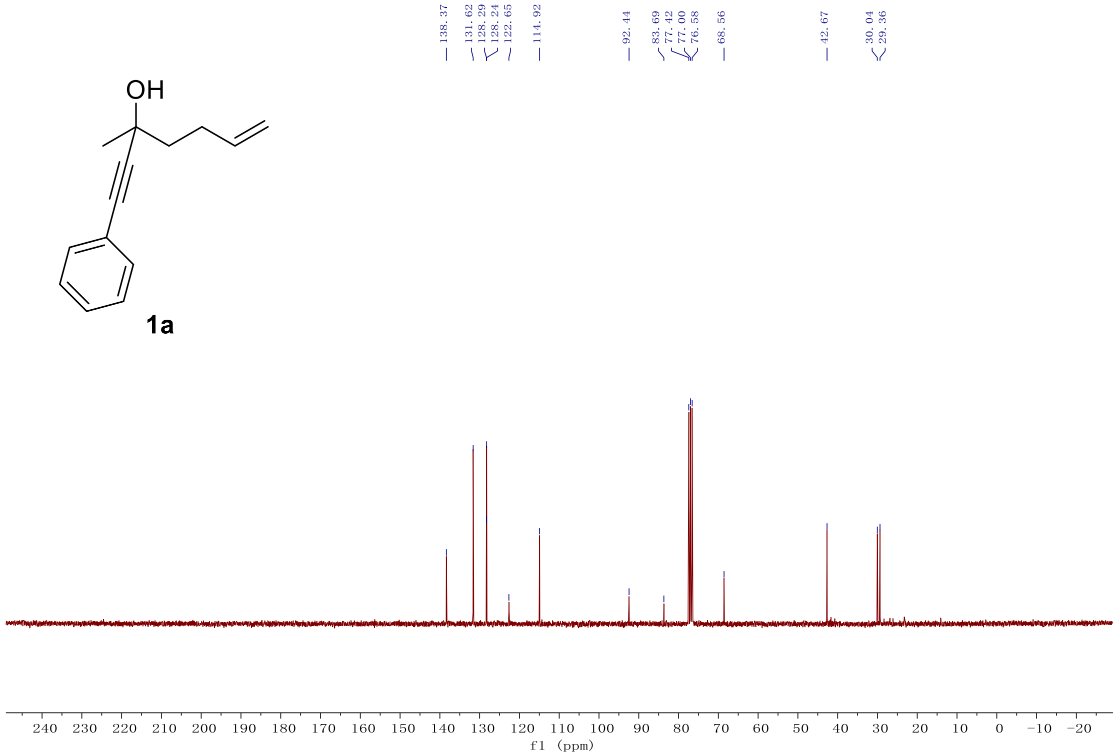

Dec20-2016.461.fid — stu tang txj 503 — carbon CDCl3 /opt/topspin av1 22

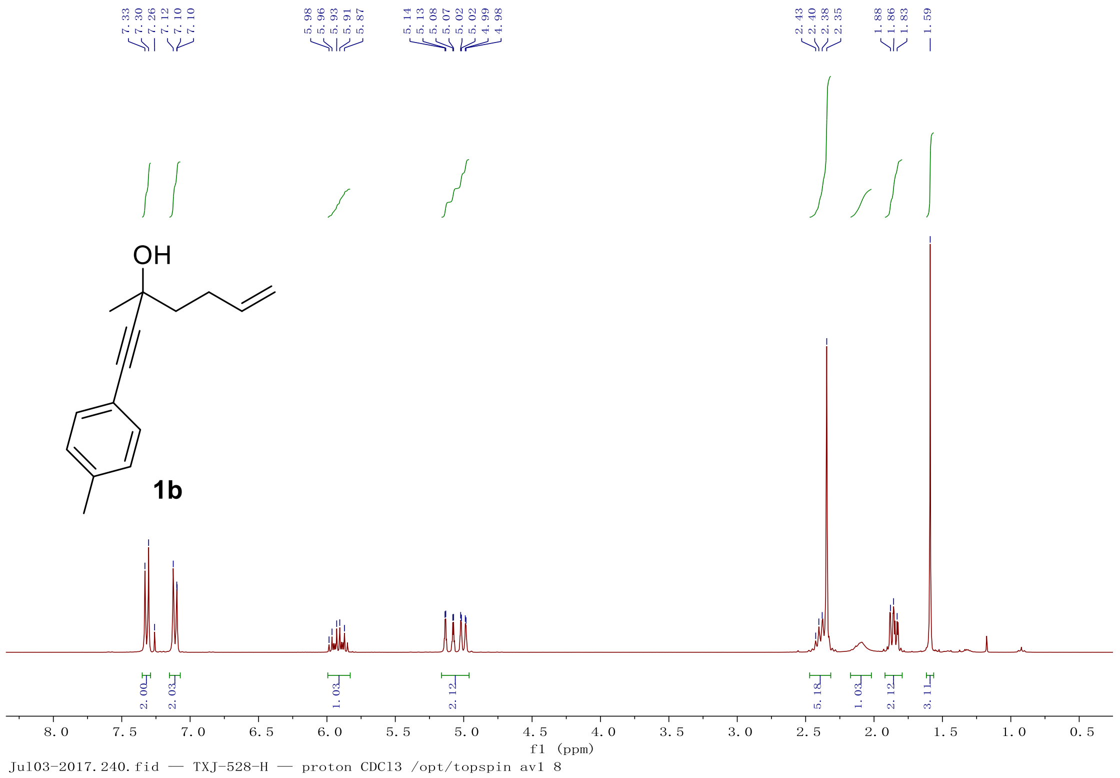
7.33
7.30
7.26
7.12
7.10
7.10
5.98
5.96
5.93
5.91
5.87
5.14
5.13
5.08
5.07
5.02
5.02
4.99
4.98
2.43
2.40
2.38
2.35
1.88
1.86
1.83
1.59
OH
1b
2.00
2.03
1.03
2.12
5.18
1.03
2.12
3.11
f1 (ppm)
Jul03-2017.240.fid — TXJ-528-H — proton CDCl3 /opt/topspin av1 8

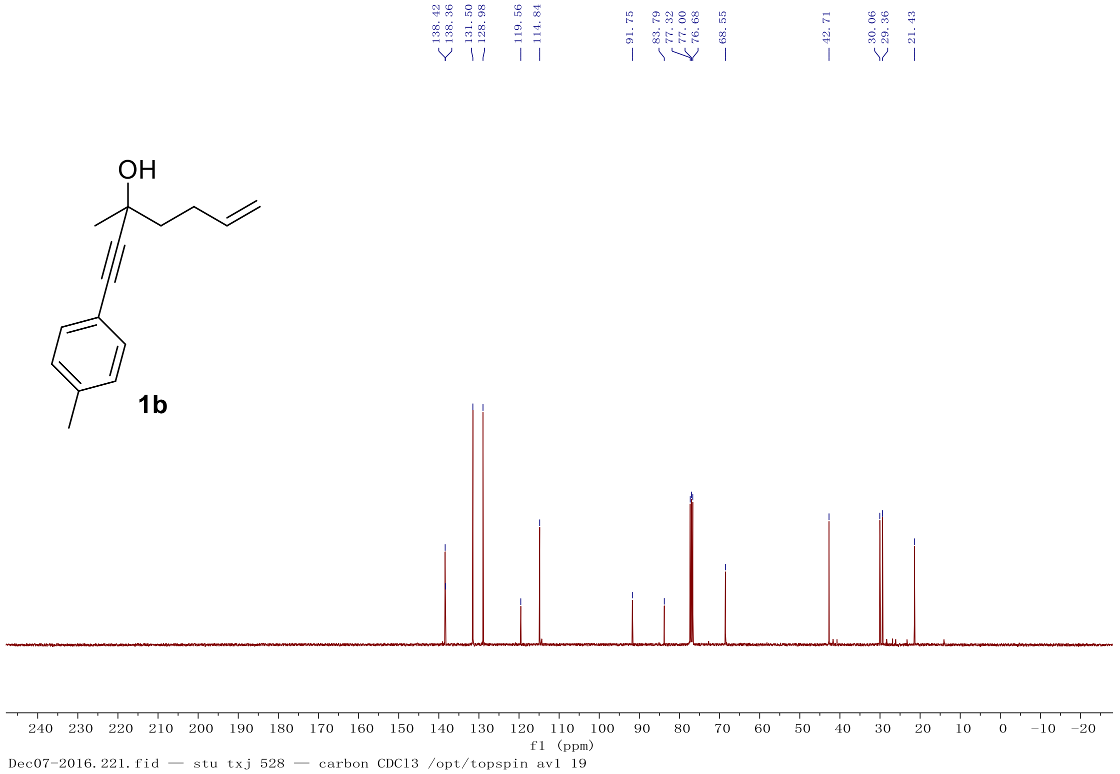
138.42
138.36
131.50
128.98
119.56
114.84
91.75
83.79
77.32
77.00
76.68
68.55
42.71
30.06
29.36
21.43
OH
1b
f1 (ppm)
Dec07-2016.221.fid — stu txj 528 — carbon CDCl3 /opt/topspin av1 19

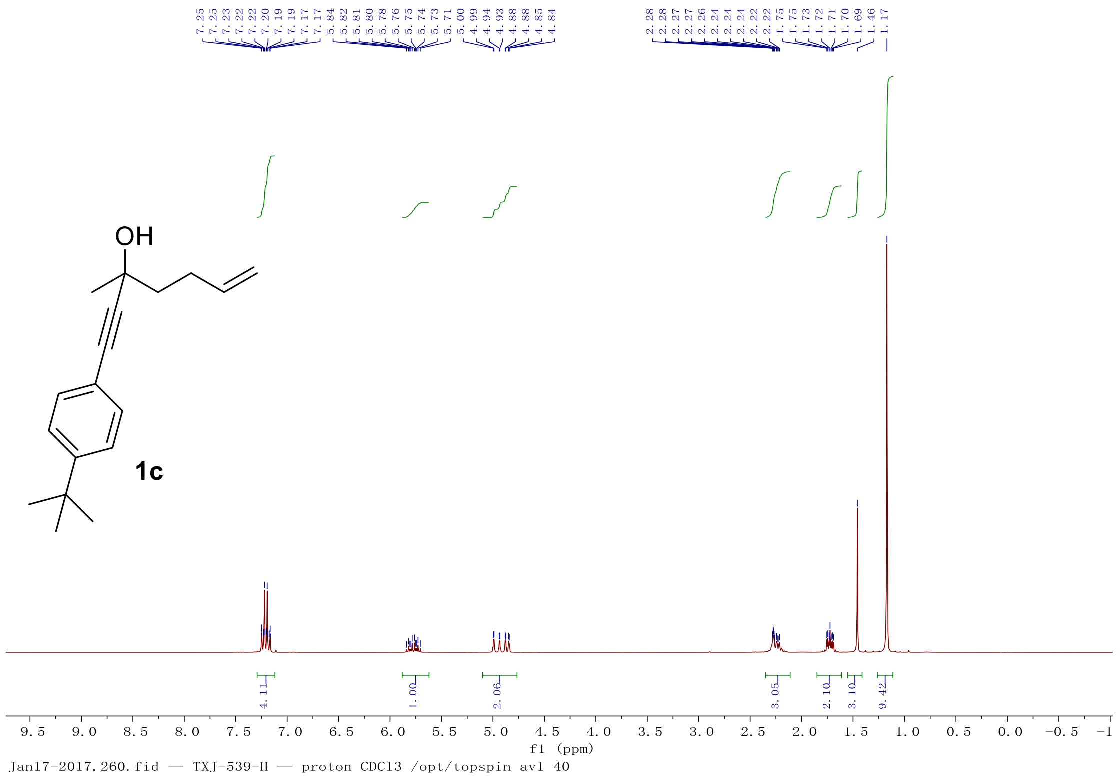
7.25 7.25 7.23 7.22 7.22 7.20 7.19 7.19 7.17 7.17 5.84 5.82 5.81 5.80 5.78 5.76 5.75 5.74 5.73 5.71 5.00 4.99 4.94 4.93 4.88 4.88 4.85 4.84
2.28 2.28 2.27 2.27 2.26 2.24 2.24 2.24 2.22 2.22 2.22 1.75 1.75 1.73 1.72 1.71 1.70 1.69 1.46 1.17
OH
1c
4.11 1.00 2.06 3.05 2.10 3.10 9.42
9.5 9.0 8.5 8.0 7.5 7.0 6.5 6.0 5.5 5.0 4.5 4.0 3.5 3.0 2.5 2.0 1.5 1.0 0.5 0.0 -0.5 -1
f1 (ppm)
Jan17-2017.260.fid — TXJ-539-H — proton CDCl3 /opt/topspin av1 40

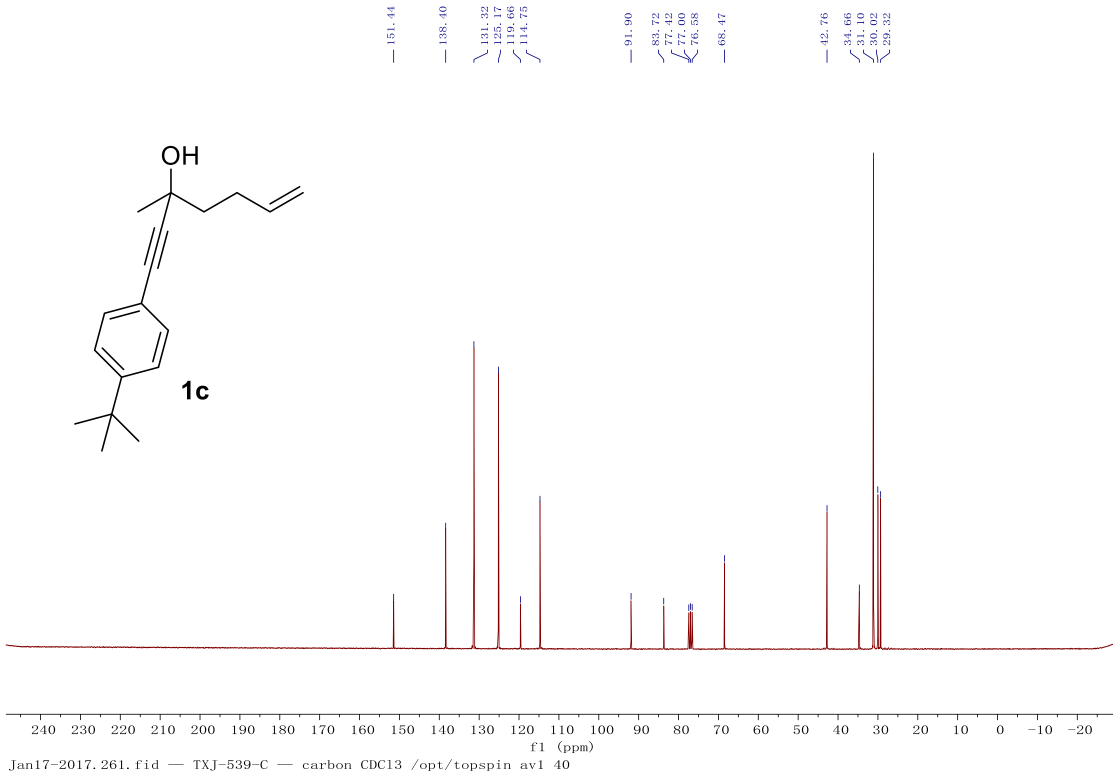
151.44 138.40 131.32 125.17 119.66 114.75 91.90 83.72 77.42 77.00 76.58 68.47 42.76 34.66 31.10 30.02 29.32
OH
1c
240 230 220 210 200 190 180 170 160 150 140 130 120 110 100 90 80 70 60 50 40 30 20 10 0 -10 -20
f1 (ppm)
Jan17-2017.261.fid — TXJ-539-C — carbon CDCl3 /opt/topspin av1 40

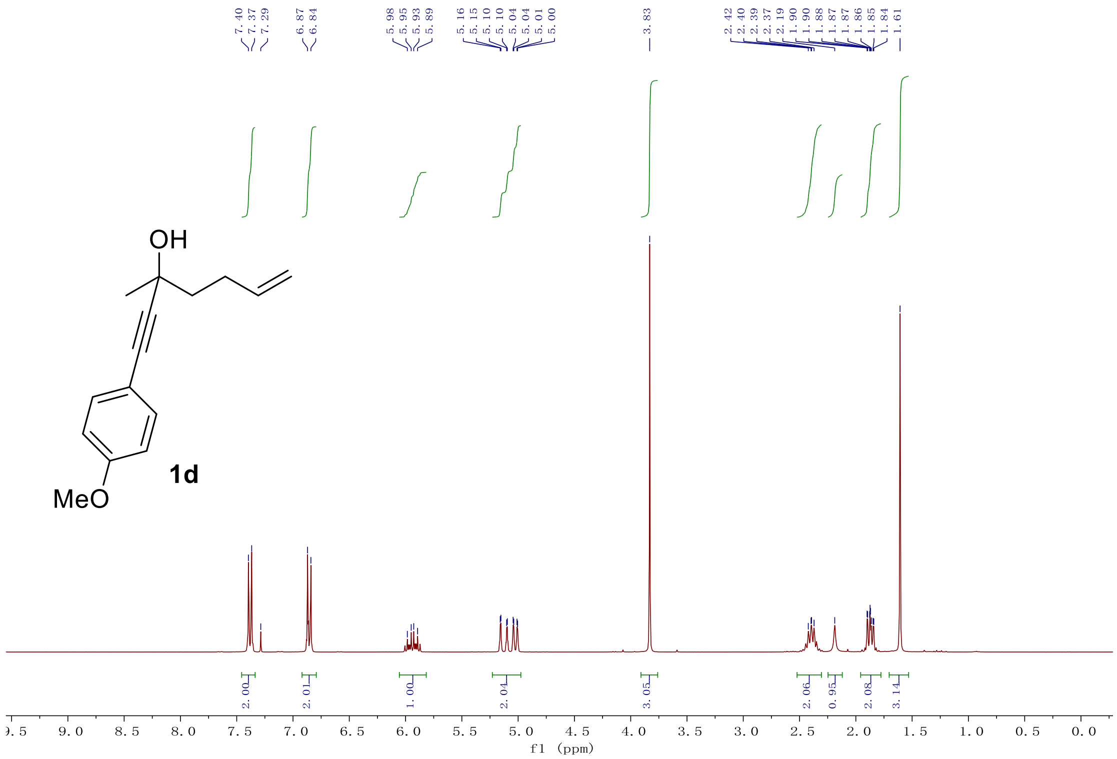

Dec09-2016.600.fid — stu tang txj 532 — proton CDCl3 /opt/topspin av1 39

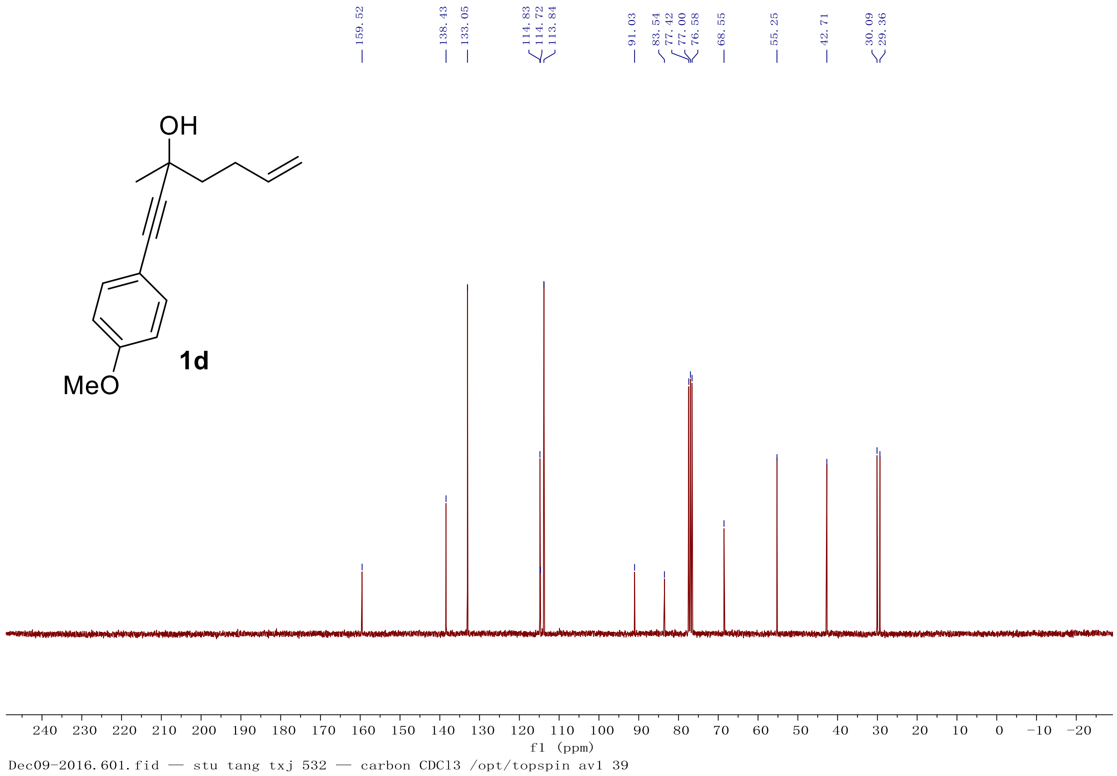

Dec09-2016.601.fid — stu tang txj 532 — carbon CDCl3 /opt/topspin av1 39

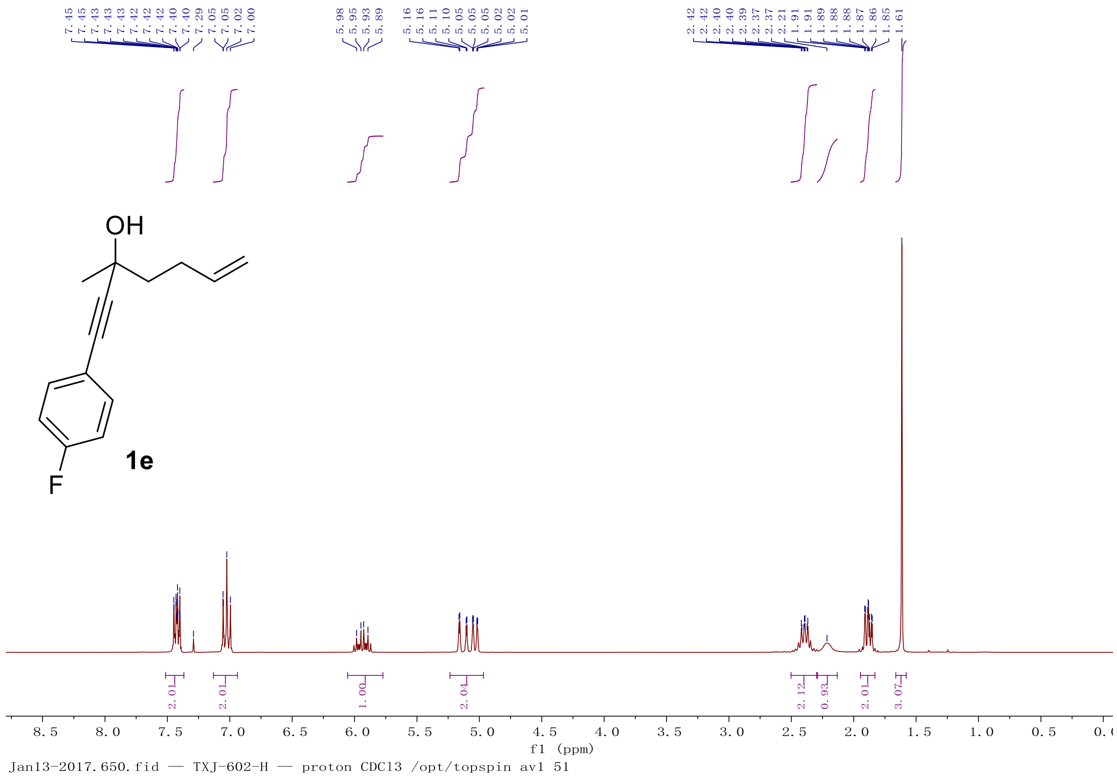
7.45 7.45 7.43 7.43 7.42 7.42 7.40 7.40 7.29 7.05 7.05 7.02 7.00
5.98 5.95 5.93 5.89
5.16 5.16 5.11 5.10 5.05 5.05 5.05 5.02 5.02 5.01
2.42 2.42 2.40 2.39 2.37 2.37 2.21 1.91 1.91 1.89 1.88 1.88 1.87 1.86 1.85 1.61
OH
F
1e
2.01 2.01 1.00 2.04 2.12 0.93 2.01 3.07
f1 (ppm)
Jan13-2017.650.fid — TXJ-602-H — proton CDCl3 /opt/topspin av1 51

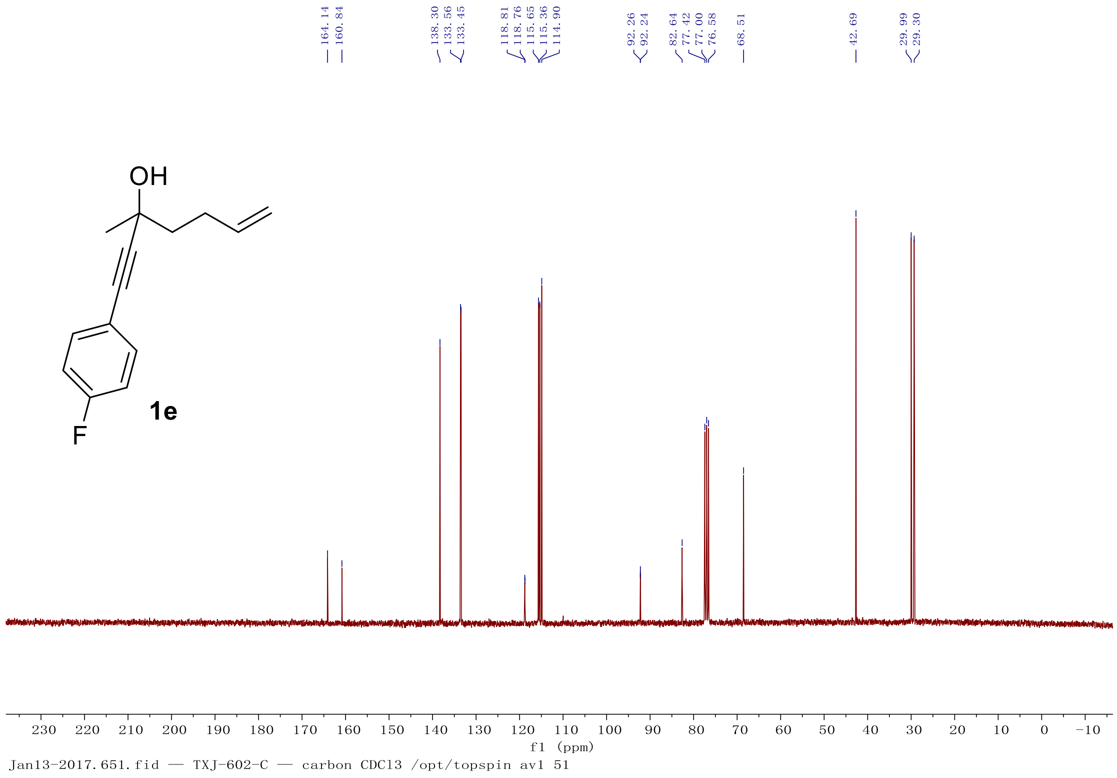
164.14 160.84
138.30 133.56 133.45
118.81 118.76 115.65 115.36 114.90
92.26 92.24
82.64 77.42 77.00 76.58
68.51
42.69
29.99 29.30
OH
F
1e
f1 (ppm)
Jan13-2017.651.fid — TXJ-602-C — carbon CDCl3 /opt/topspin av1 51

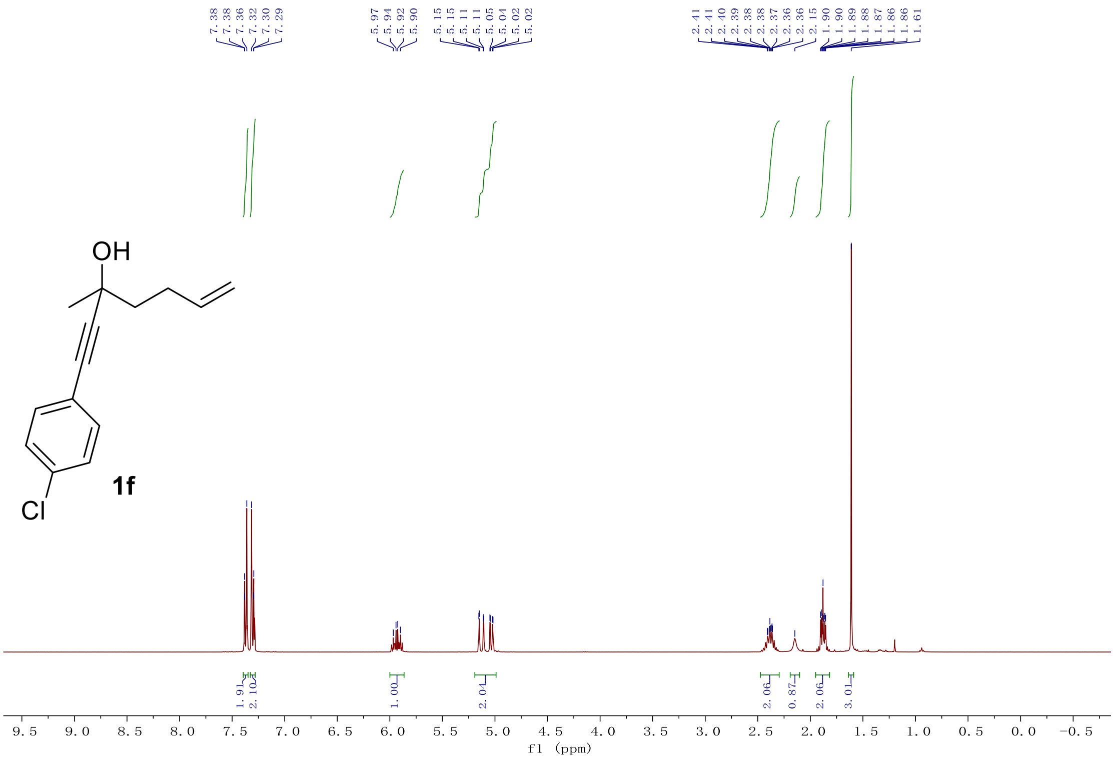

1f

Dec12-2016.80.fid — stu tang txj 533 — proton CDCl3 /opt/topspin av1 1

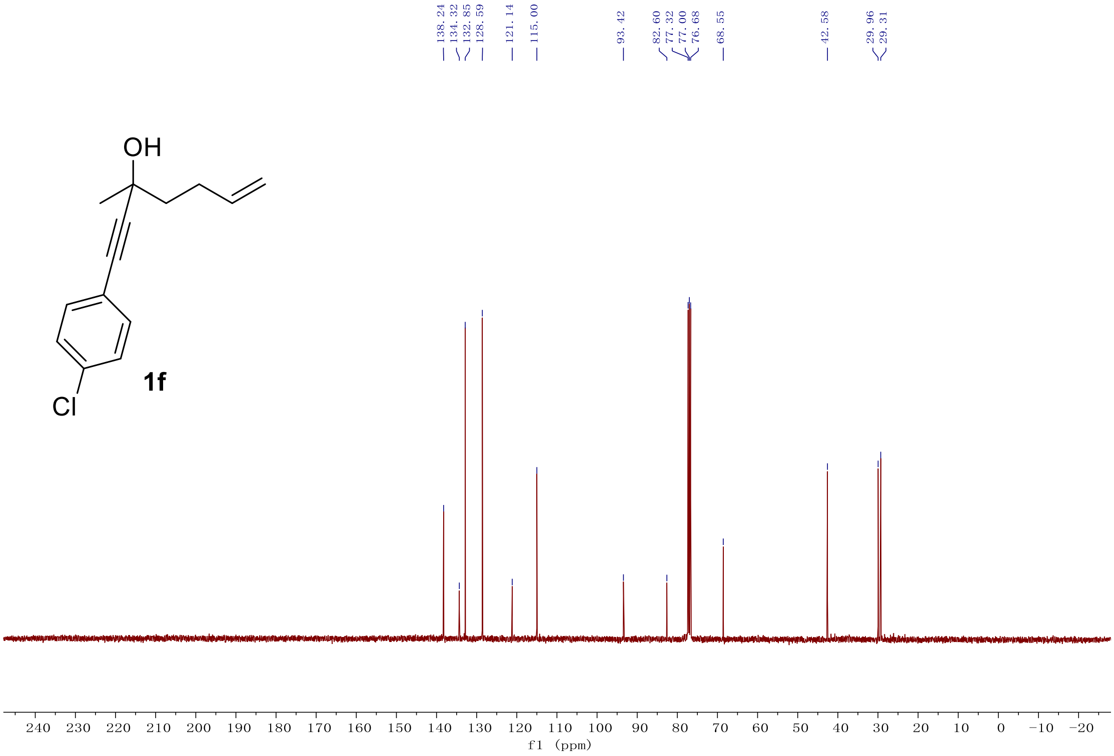

1f

Dec12-2016.81.fid — stu tang txj 533 — carbon CDCl3 /opt/topspin av1 1

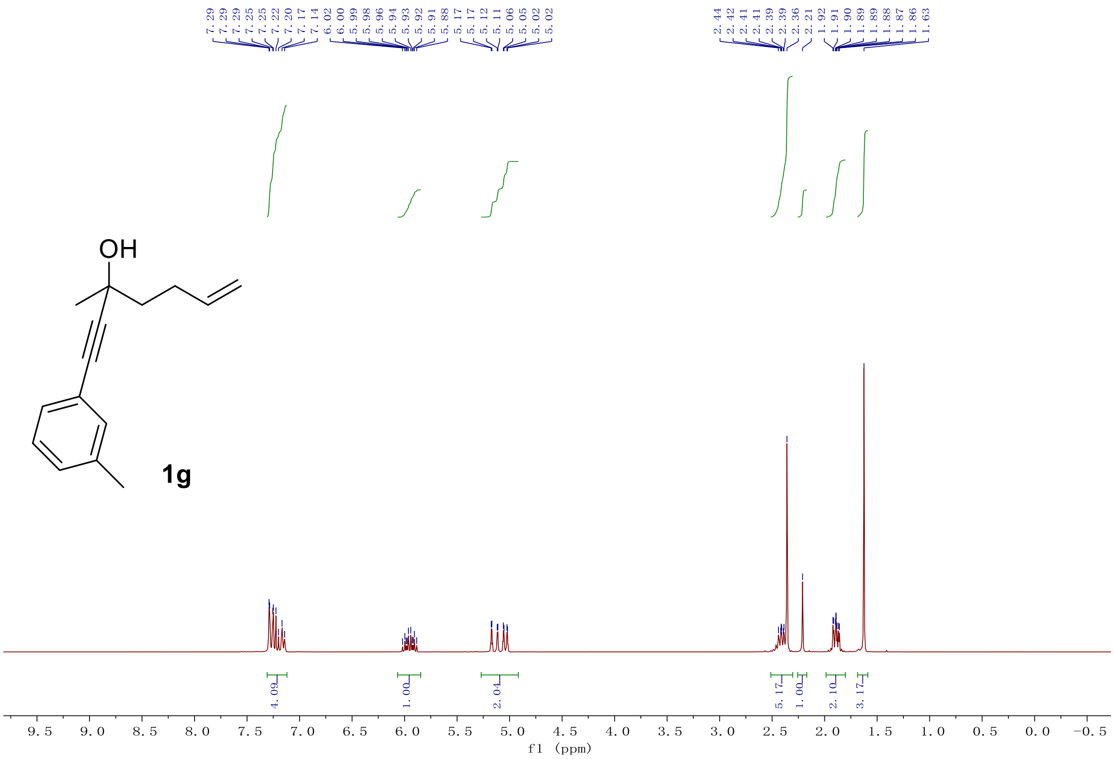

Jan13-2017.620.fid — TXJ-535-H — proton CDCl3 /opt/topspin av1 48

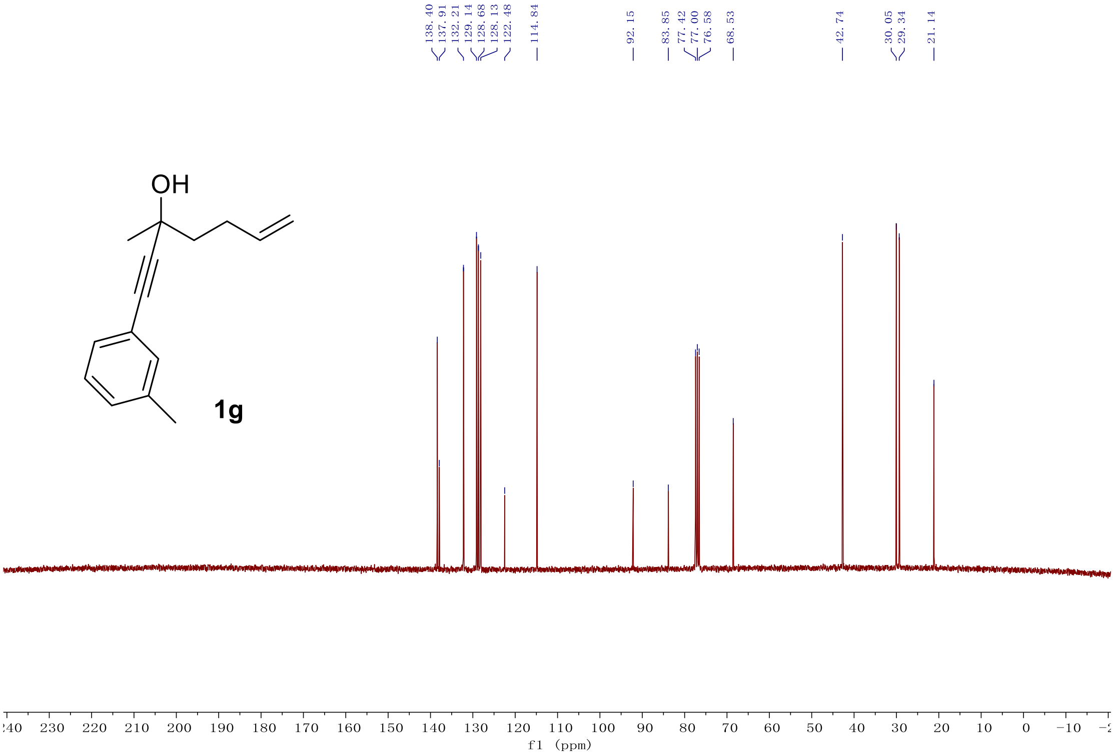

Jan13-2017.621.fid — TXJ-535-C — carbon CDCl3 /opt/topspin av1 48

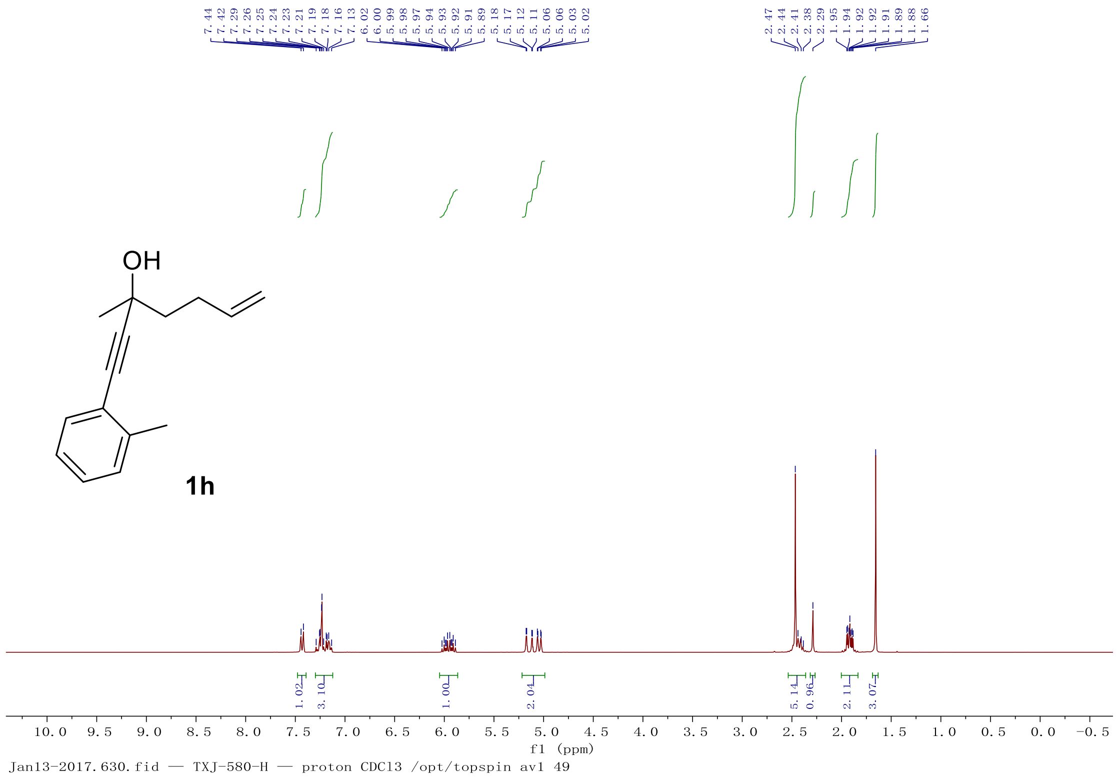

OH
1h
f1 (ppm)
Jan13-2017.630.fid — TXJ-580-H — proton CDCl3 /opt/topspin av1 49

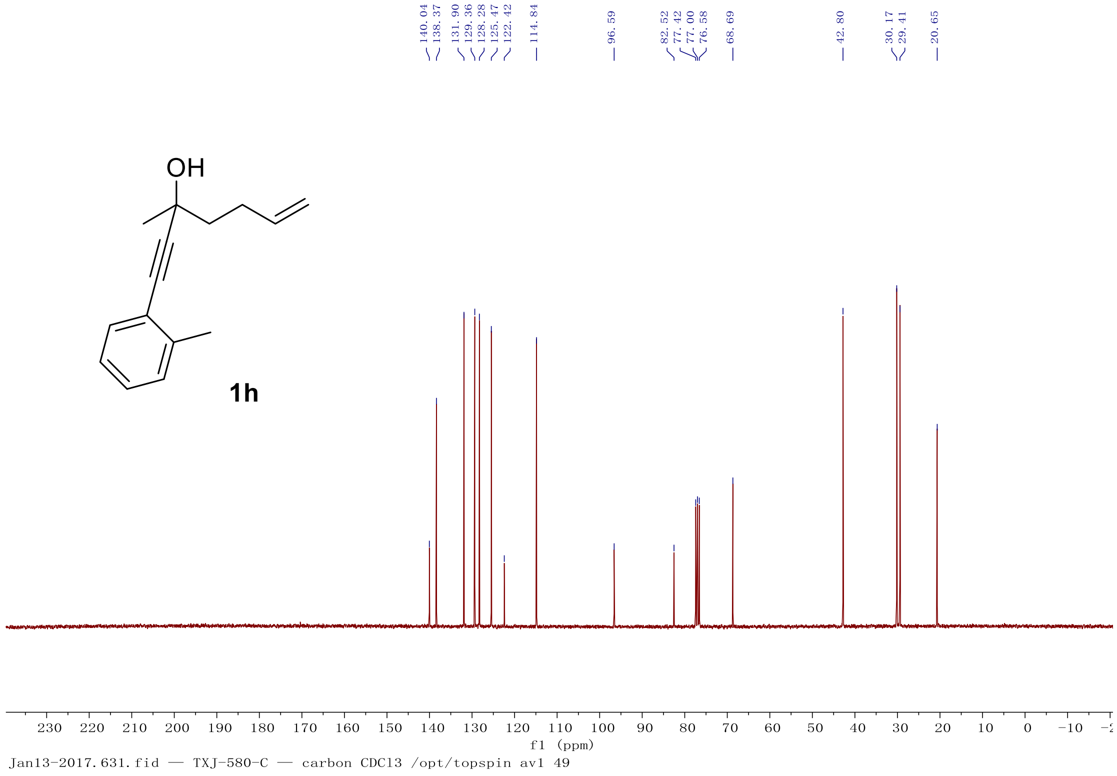

OH
1h
f1 (ppm)
Jan13-2017.631.fid — TXJ-580-C — carbon CDCl3 /opt/topspin av1 49

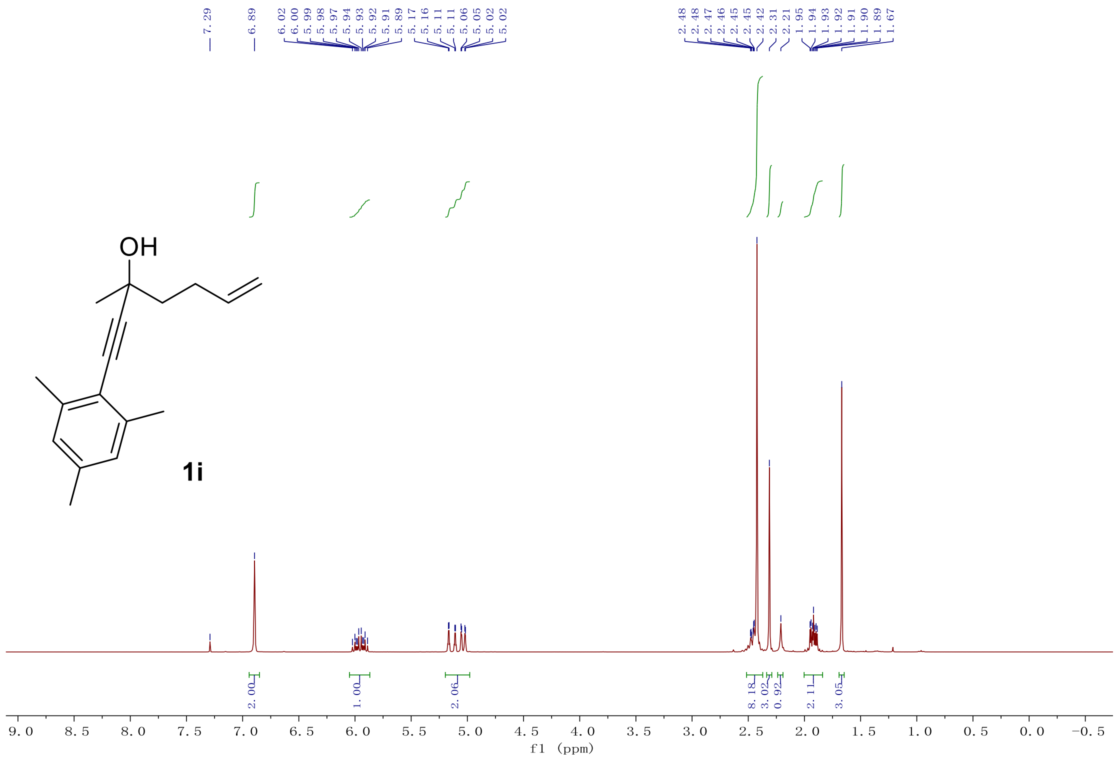

Jan13-2017.640.fid — TXJ-596-H — proton CDCl3 /opt/topspin av1 50

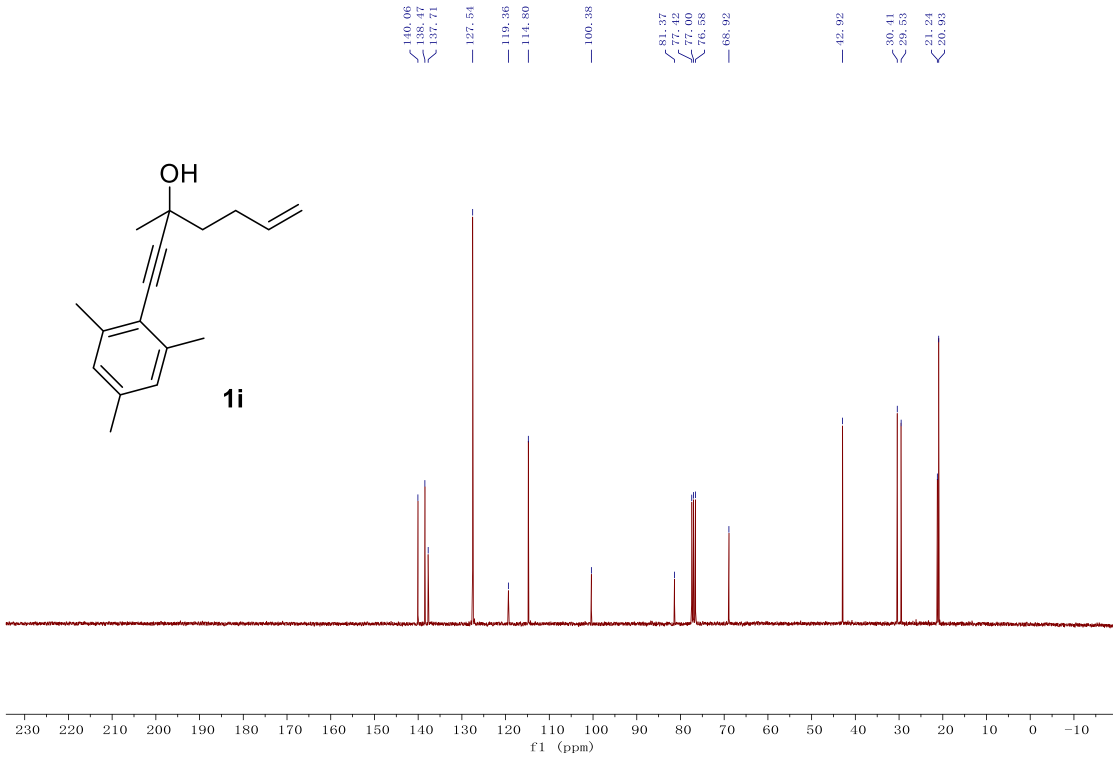

Jan13-2017.641.fid — TXJ-596-C — carbon CDCl3 /opt/topspin av1 50

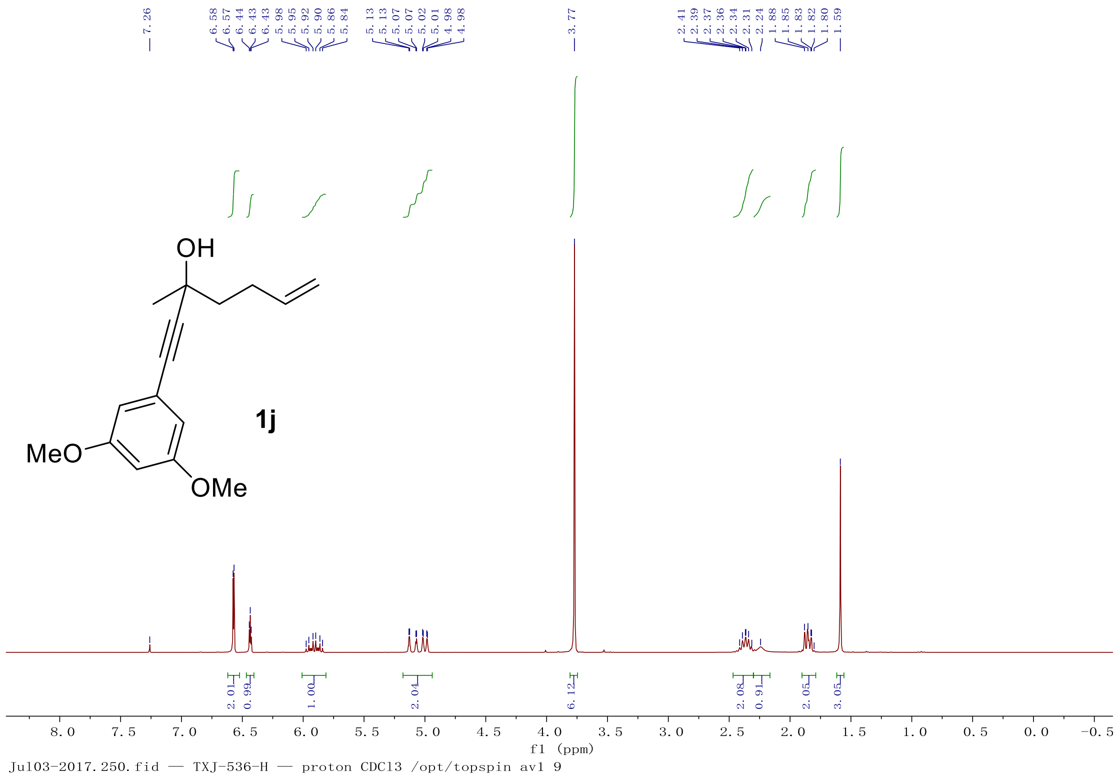
7.26
6.58
6.57
6.44
6.43
6.43
5.98
5.95
5.92
5.90
5.86
5.84
5.13
5.13
5.07
5.02
5.01
4.98
4.98
3.77
2.41
2.39
2.37
2.36
2.34
2.31
2.24
1.88
1.85
1.83
1.82
1.80
1.59
OH
1j
MeO
OMe
2.01
0.99
1.00
2.04
6.12
2.08
0.91
2.05
3.05
f1 (ppm)
Jul03-2017.250.fid — TXJ-536-H — proton CDCl3 /opt/topspin av1 9

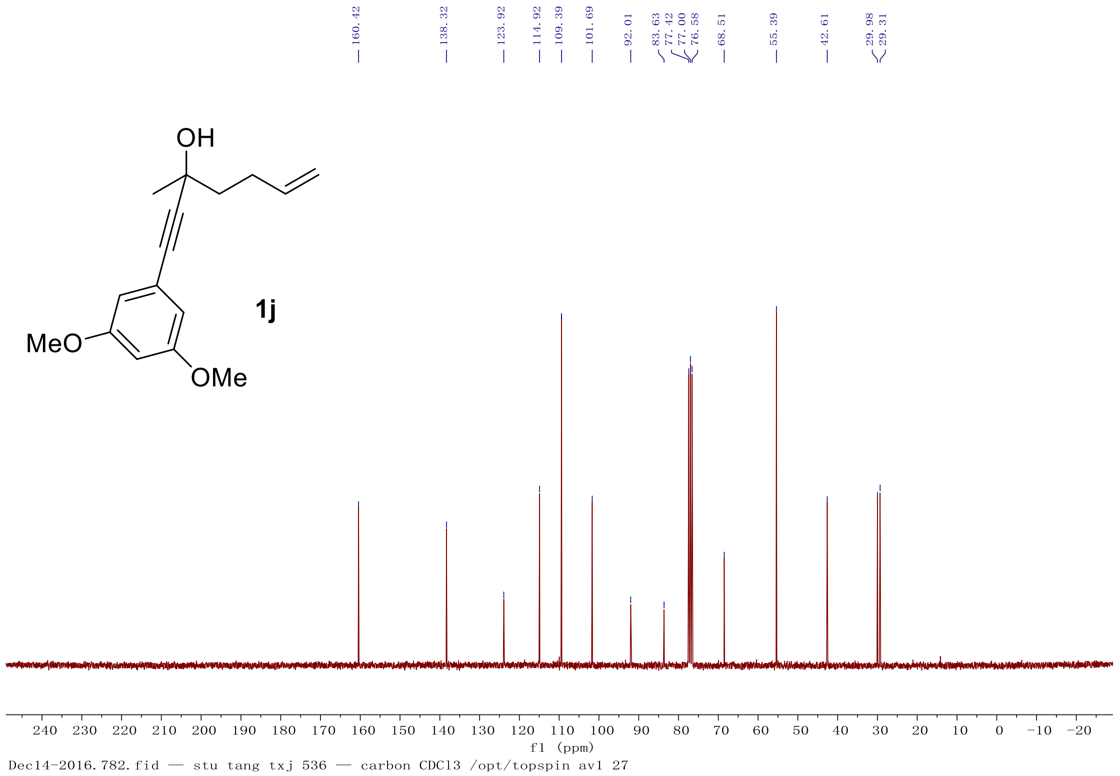
160.42
138.32
123.92
114.92
109.39
101.69
92.01
83.63
77.42
77.00
76.58
68.51
55.39
42.61
29.98
29.31
OH
1j
MeO
OMe
f1 (ppm)
Dec14-2016.782.fid — stu tang txj 536 — carbon CDCl3 /opt/topspin av1 27

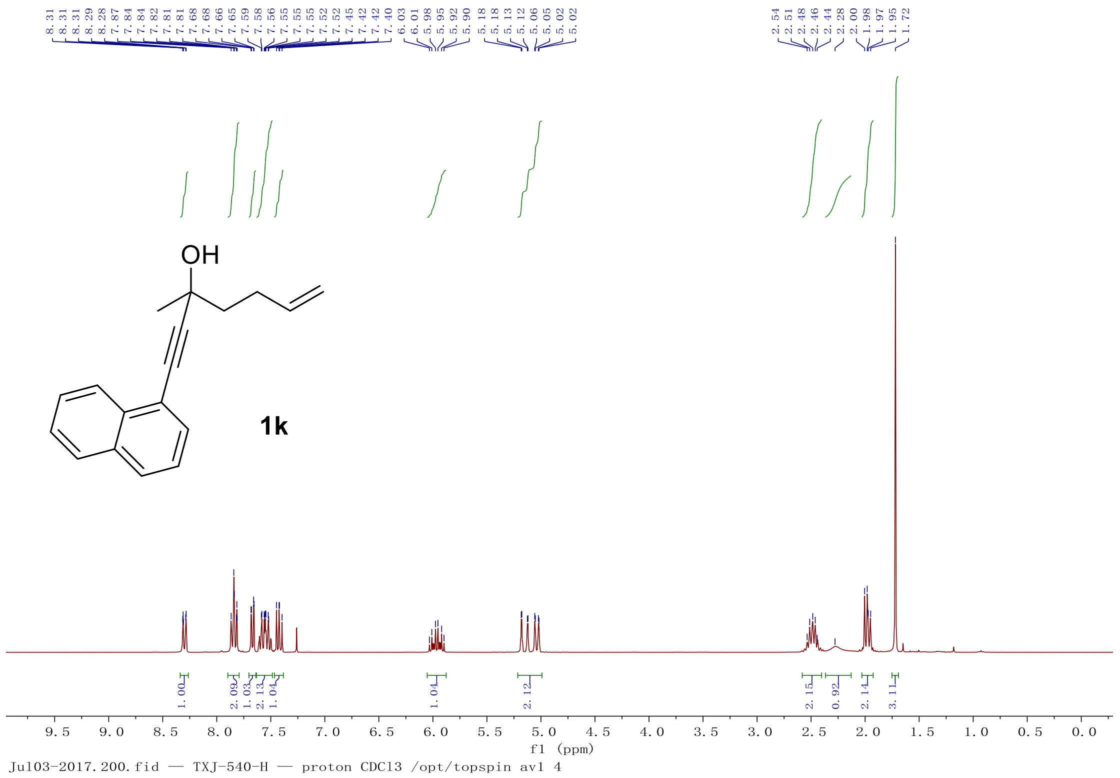
1k
f1 (ppm)
Jul03-2017.200.fid — TXJ-540-H — proton CDCl3 /opt/topspin av1 4

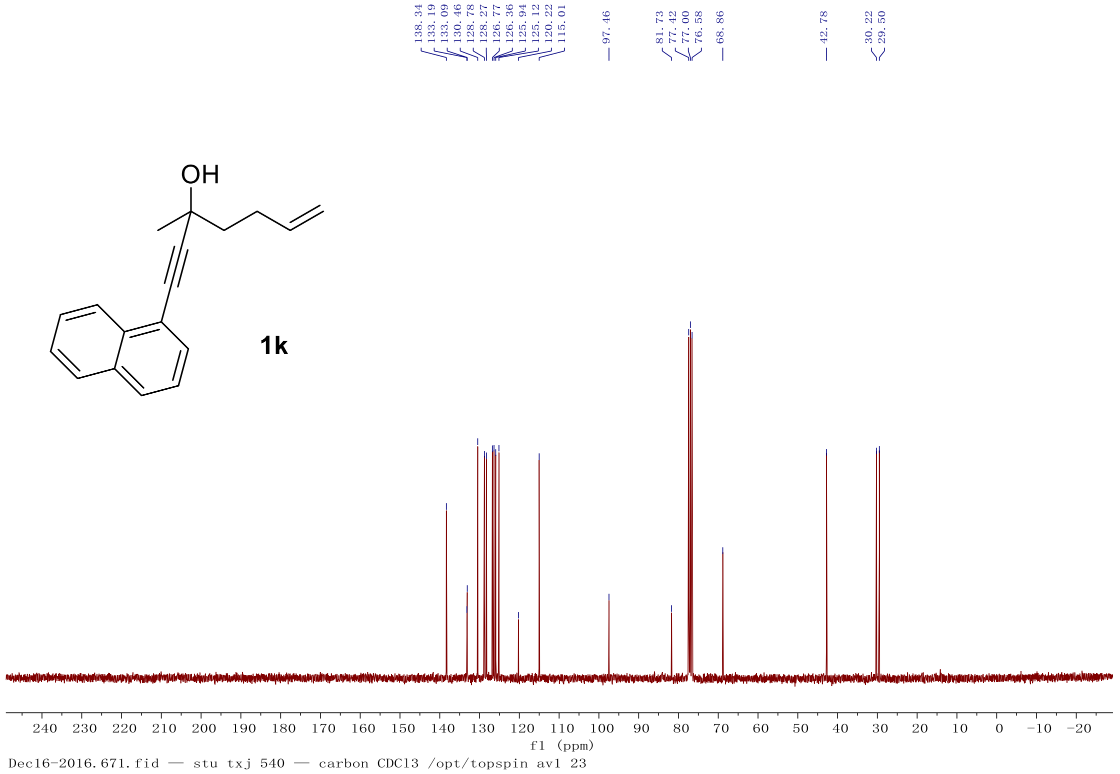
1k
f1 (ppm)
Dec16-2016.671.fid — stu txj 540 — carbon CDCl3 /opt/topspin av1 23

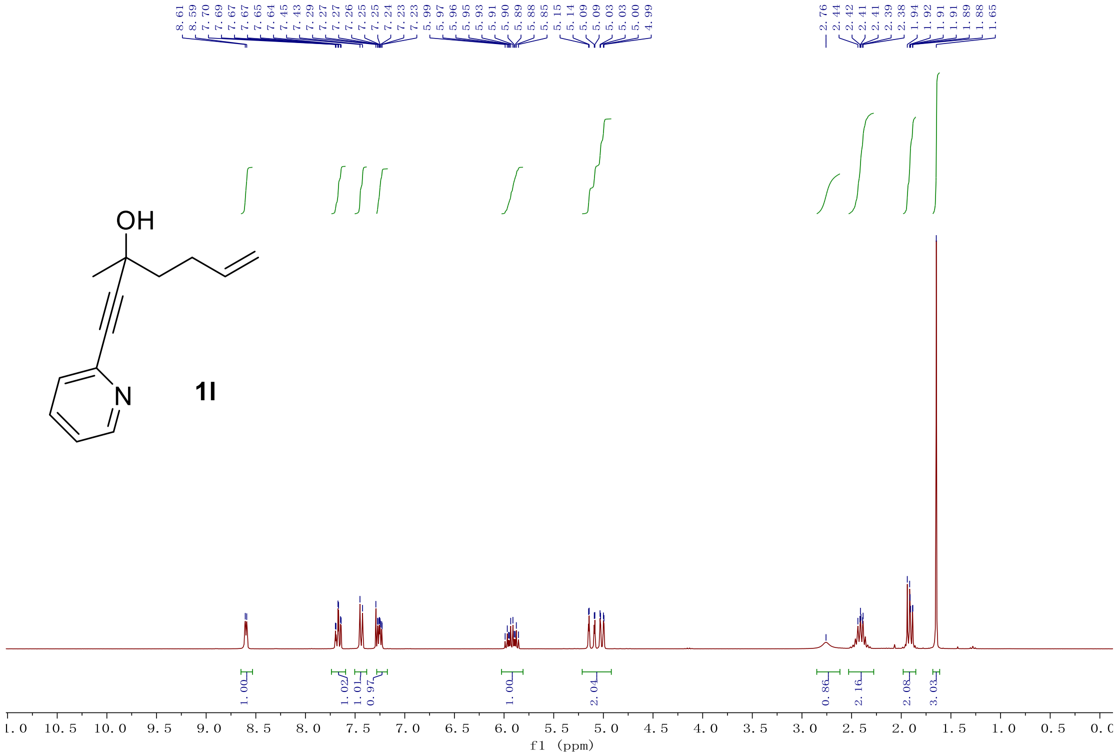

Jan29-2017.81.fid — TXJ-636-H — proton CDCl3 /opt/topspin av1 43

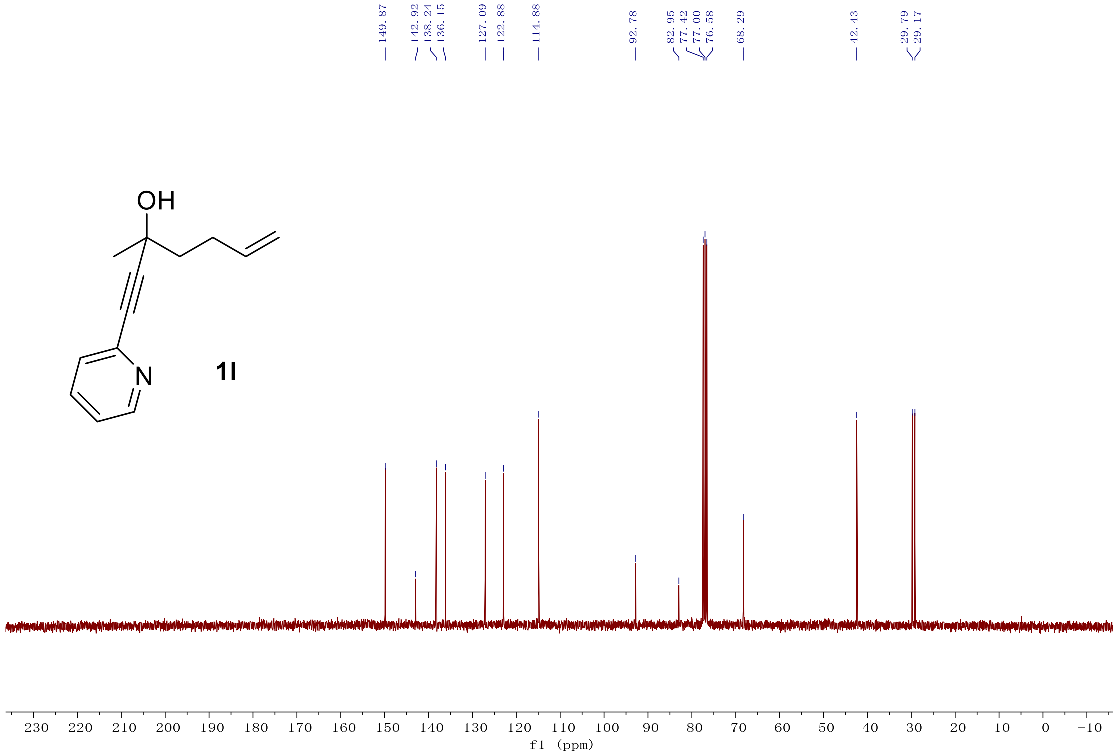

Jan29-2017.82.fid — TXJ-636-C — carbon CDCl3 /opt/topspin av1 43

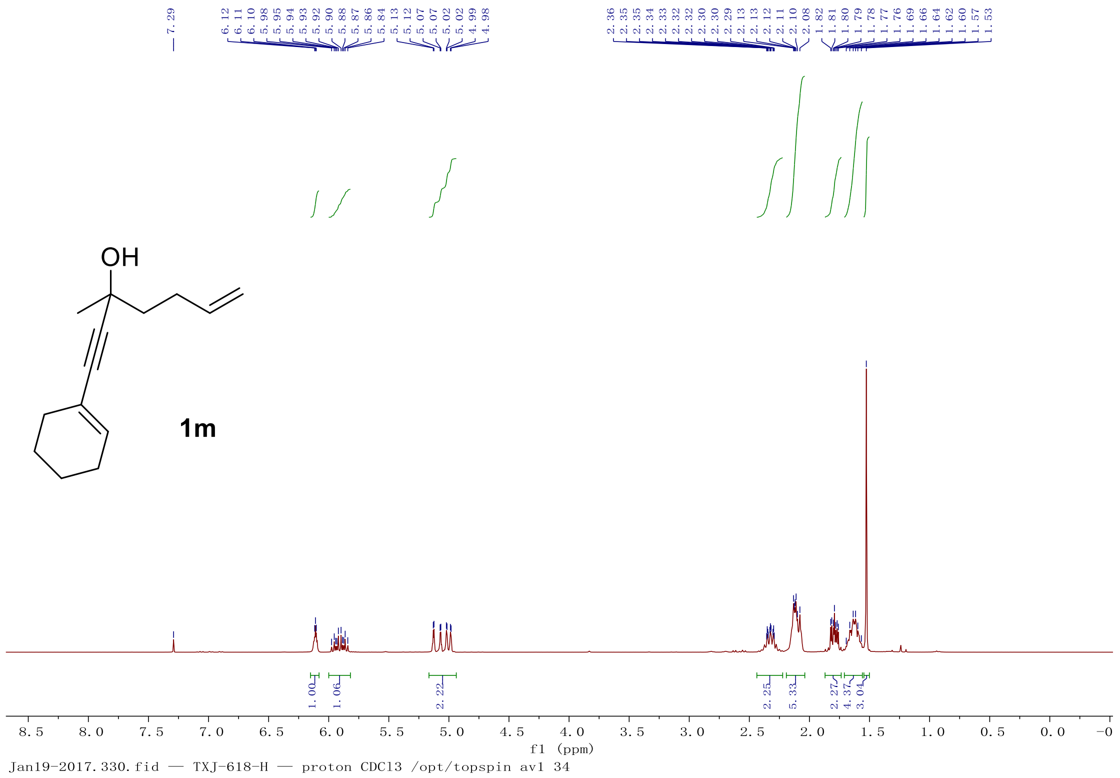
OH
1m
f1 (ppm)
Jan19-2017.330.fid — TXJ-618-H — proton CDCl3 /opt/topspin av1 34

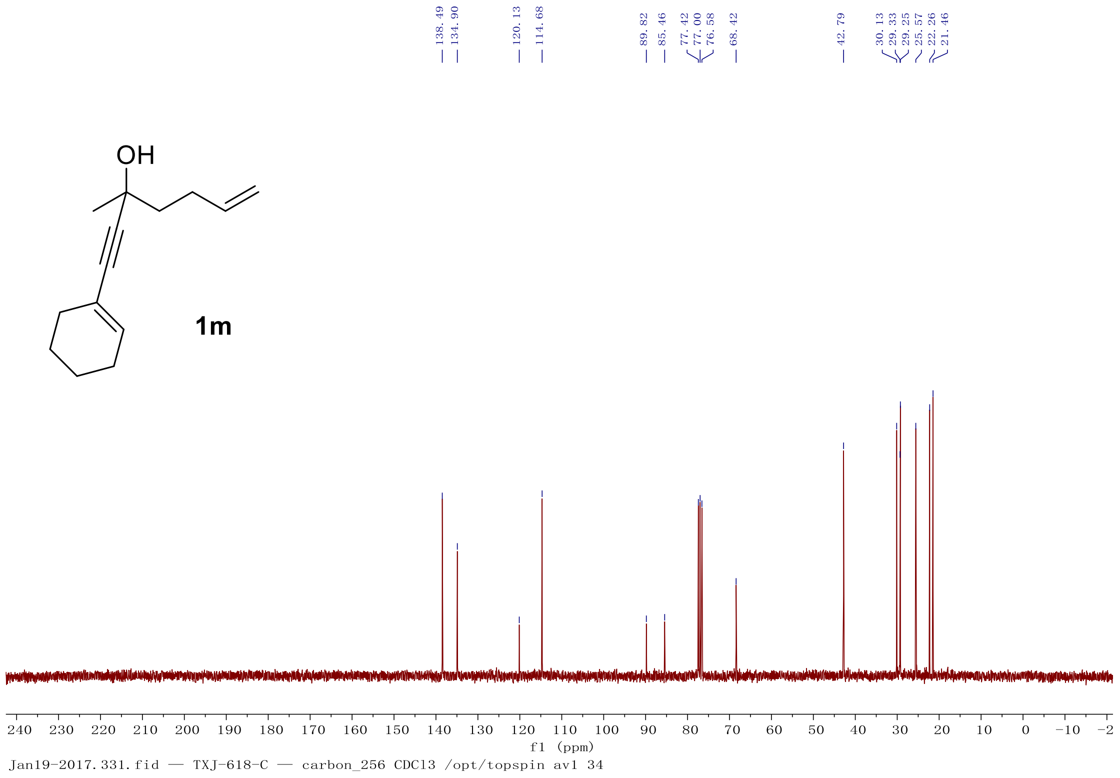
OH
1m
138.49
134.90
120.13
114.68
89.82
85.46
77.42
77.00
76.58
68.42
42.79
30.13
29.33
29.25
25.57
22.26
21.46
f1 (ppm)
Jan19-2017.331.fid — TXJ-618-C — carbon_256 CDCl3 /opt/topspin av1 34

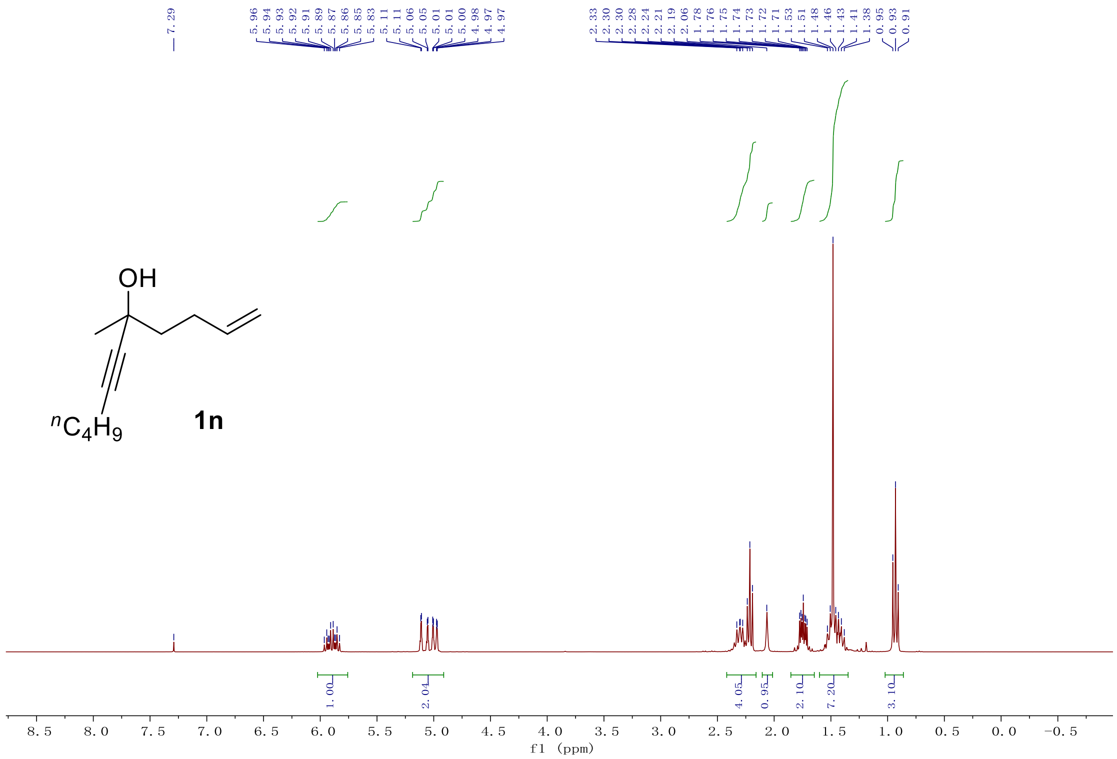

Jan18-2017.340.fid — TXJ-614-1-H — proton CDCl3 /opt/topspin av1 33

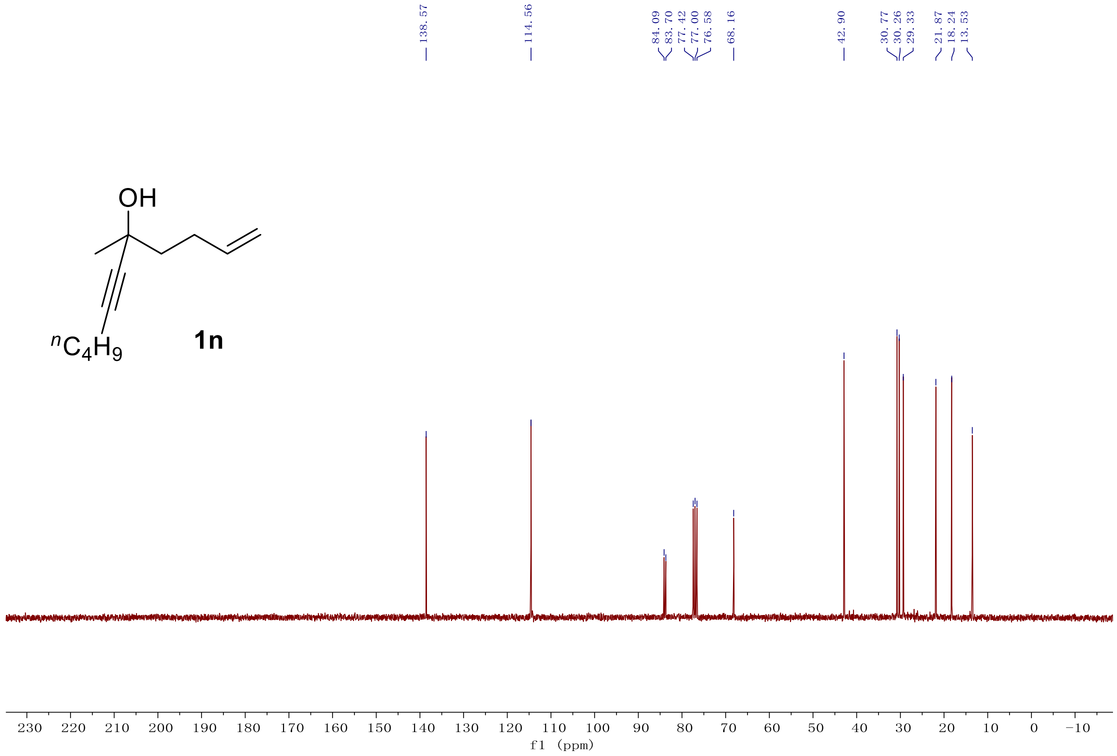

Jan18-2017.341.fid — TXJ-614-1-C — carbon_256 CDCl3 /opt/topspin av1 33

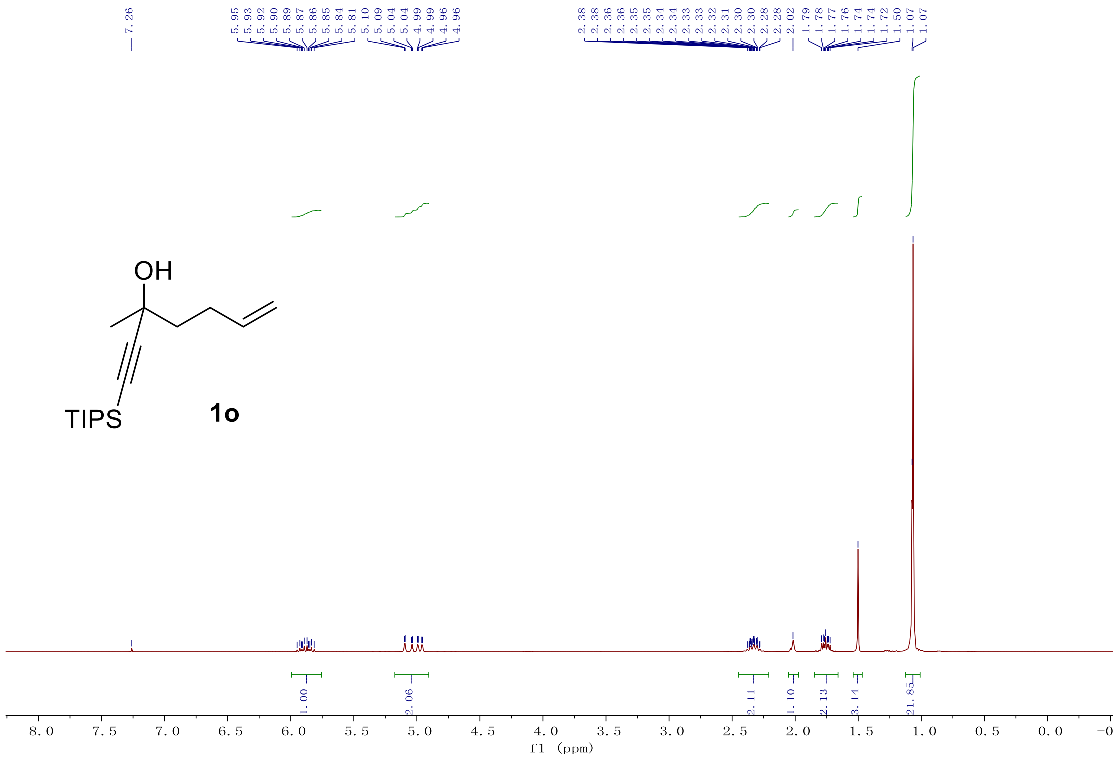

Mar12-2017.80.fid — TXJ-716-H — proton CDCl3 /opt/topspin av1 31

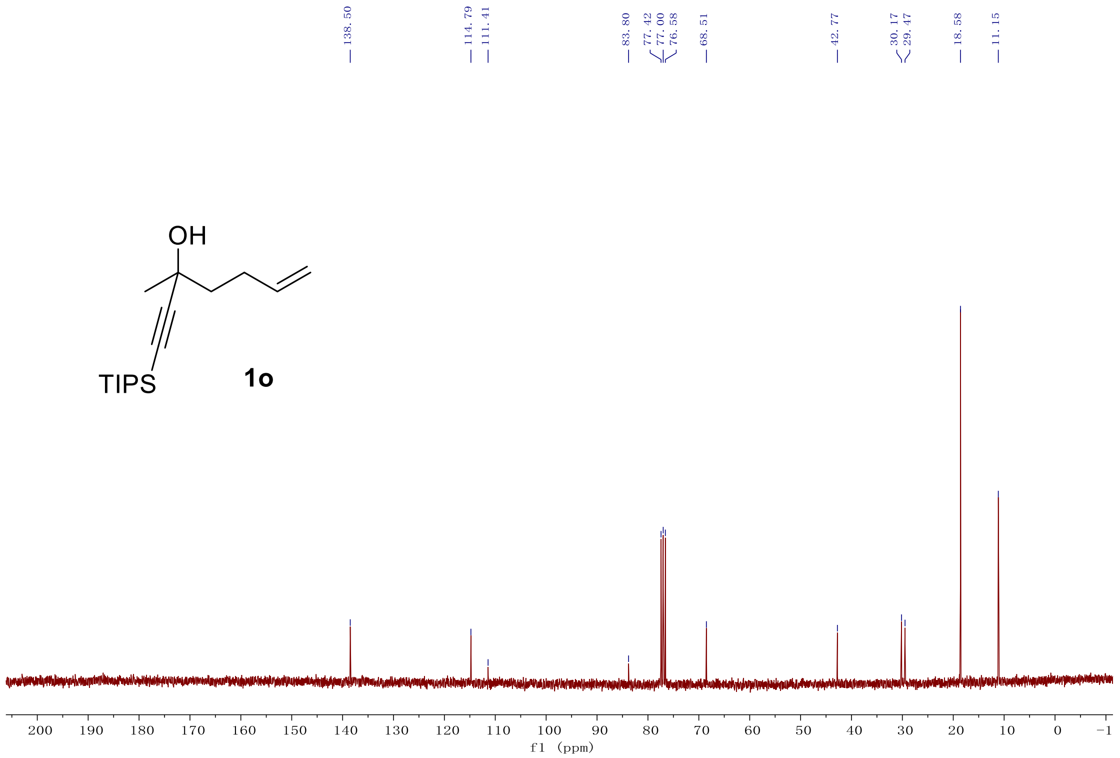

Mar12-2017.81.fid — TXJ-716-C — carbon_256 CDCl3 /opt/topspin av1 31

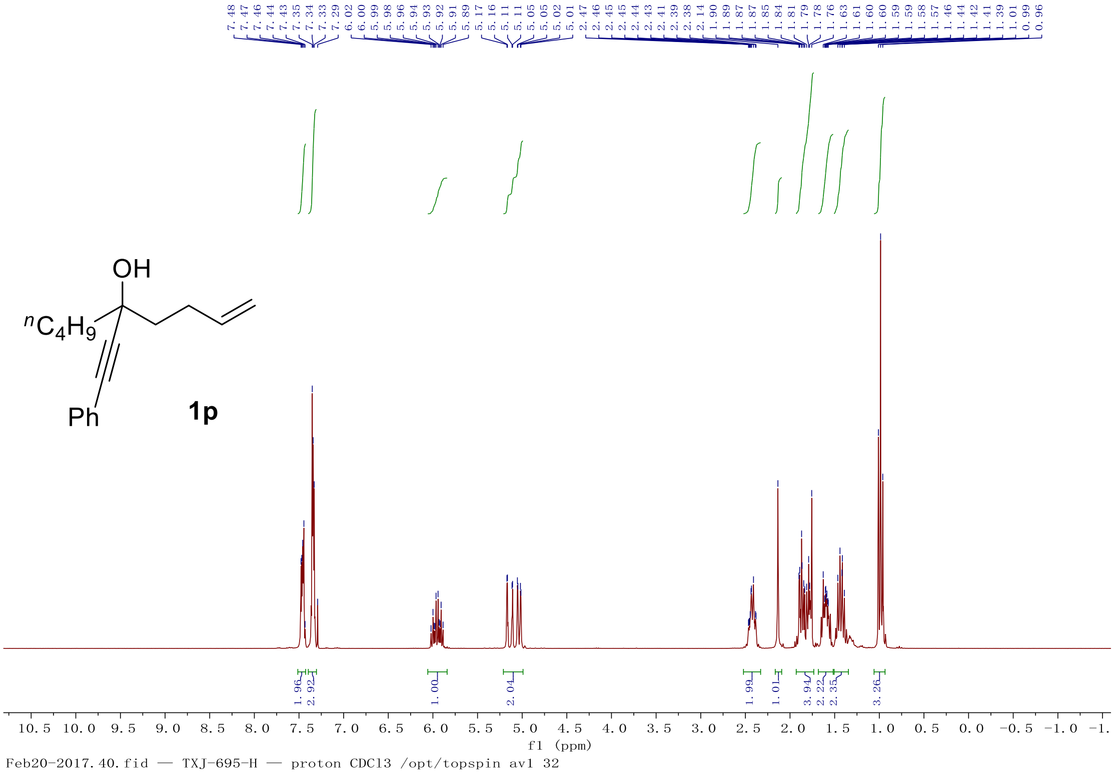
7.48 7.47 7.46 7.44 7.43 7.35 7.34 7.33 7.29 6.02 6.00 5.99 5.98 5.96 5.94 5.93 5.92 5.91 5.89 5.17 5.16 5.11 5.11 5.05 5.05 5.02 5.01 2.47 2.46 2.45 2.45 2.44 2.43 2.41 2.39 2.38 2.14 1.90 1.89 1.87 1.87 1.85 1.84 1.81 1.79 1.78 1.76 1.63 1.61 1.60 1.60 1.59 1.59 1.58 1.57 1.46 1.44 1.42 1.41 1.39 1.01 0.99 0.96
OH
$^{n}C_4H_9$
Ph
1p
1.96 2.92 1.00 2.04 1.99 1.01 3.94 2.22 2.35 3.26
10.5 10.0 9.5 9.0 8.5 8.0 7.5 7.0 6.5 6.0 5.5 5.0 4.5 4.0 3.5 3.0 2.5 2.0 1.5 1.0 0.5 0.0 −0.5 −1.0 −1.
f1 (ppm)
Feb20-2017.40.fid — TXJ-695-H — proton CDCl3 /opt/topspin av1 32

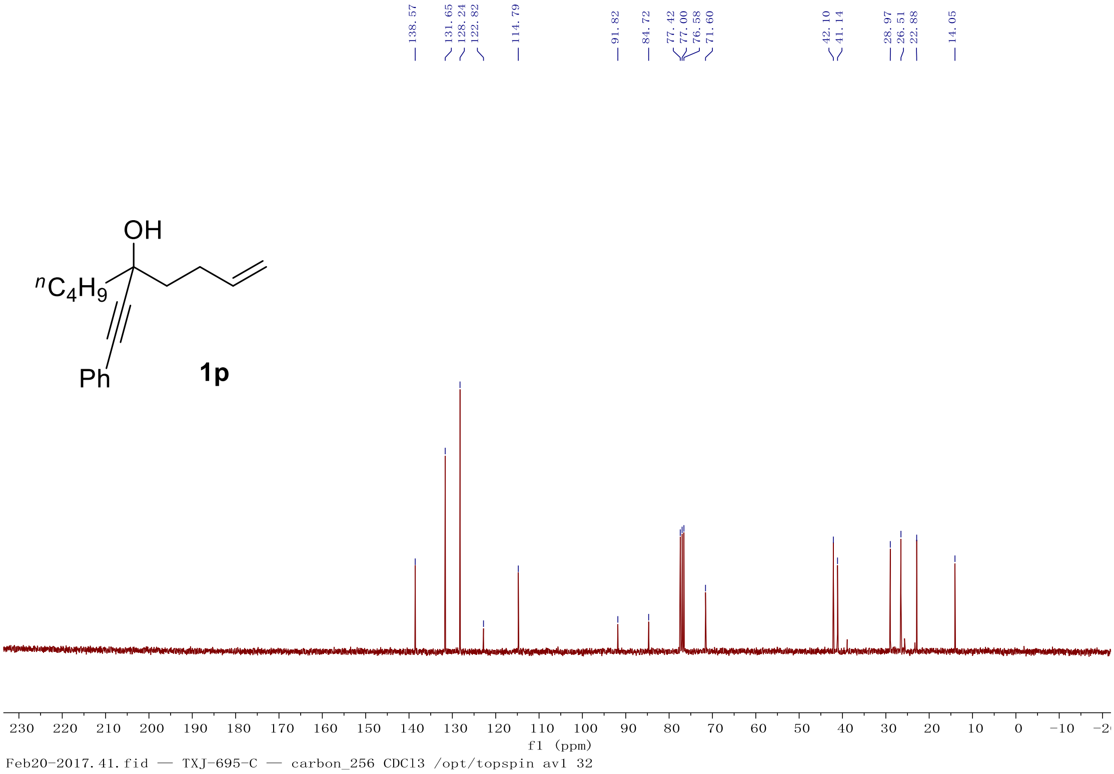
138.57 131.65 128.24 122.82 114.79 91.82 84.72 77.42 77.00 76.58 71.60 42.10 41.14 28.97 26.51 22.88 14.05
OH
$^{n}C_4H_9$
Ph
1p
230 220 210 200 190 180 170 160 150 140 130 120 110 100 90 80 70 60 50 40 30 20 10 0 −10 −2
f1 (ppm)
Feb20-2017.41.fid — TXJ-695-C — carbon_256 CDCl3 /opt/topspin av1 32

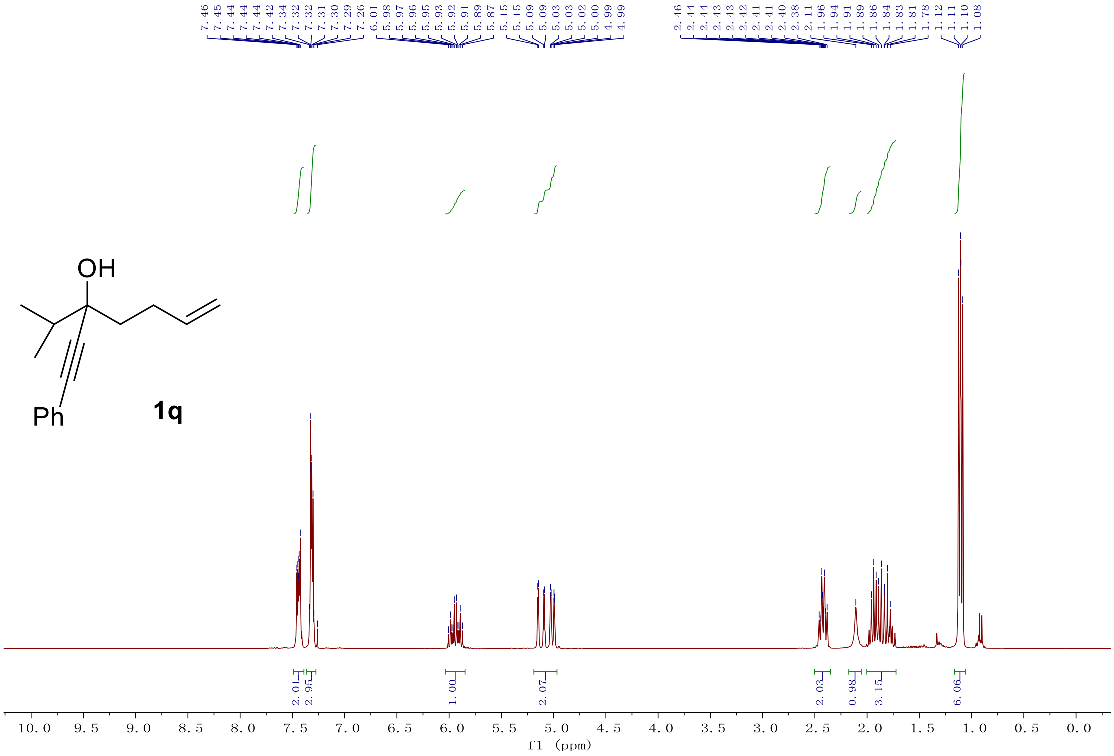

Feb22-2017.90.fid — TXJ-697-H — proton CDCl3 /opt/topspin av1 31

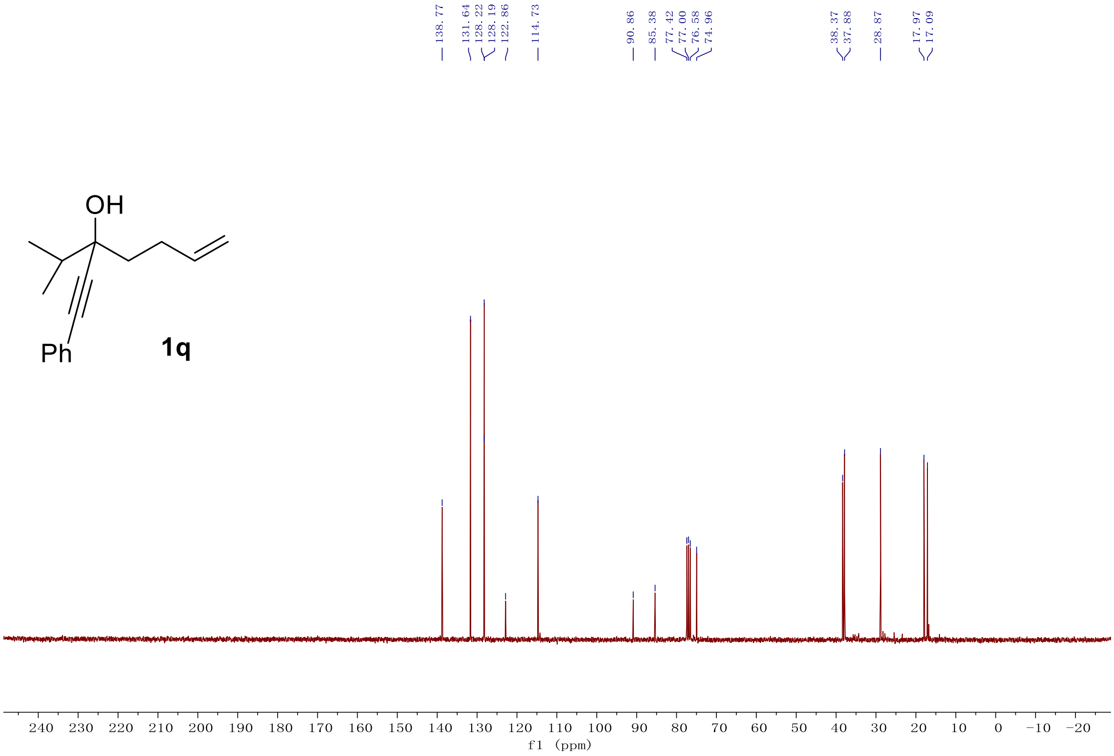

Feb22-2017.91.fid — TXJ-697-C — carbon_256 CDCl3 /opt/topspin av1 31

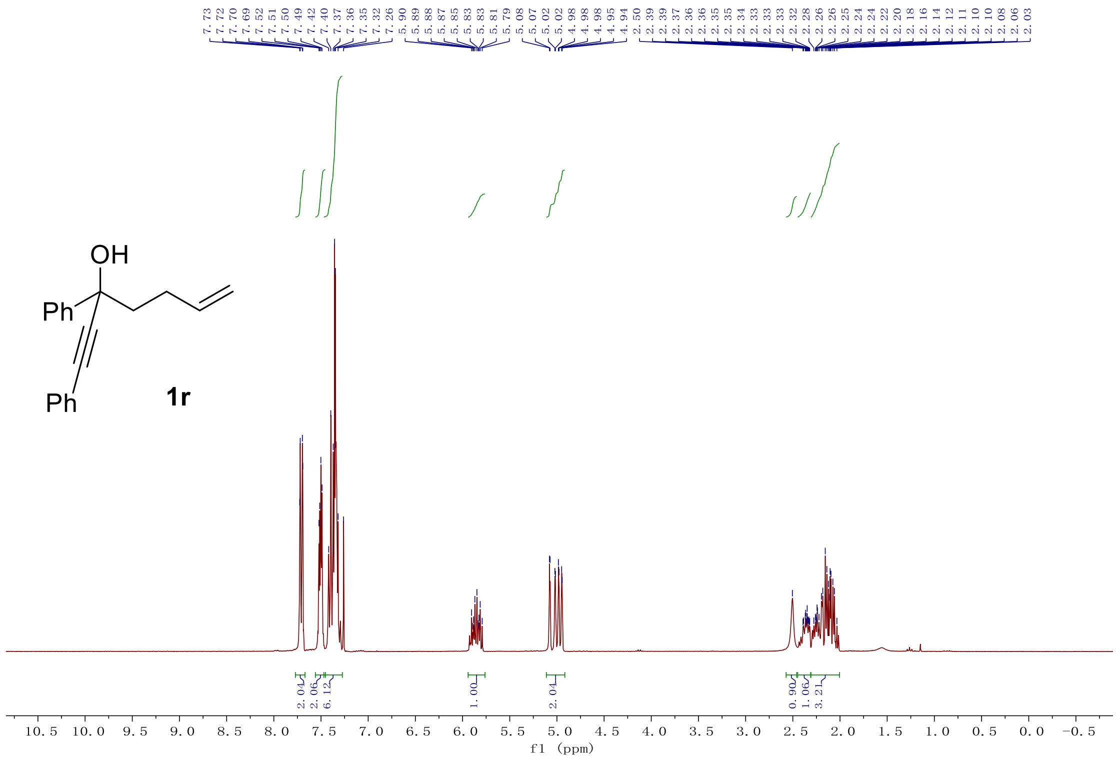

Feb03-2017.200.fid — TXj-656-H — proton CDCl3 /opt/topspin av1 32

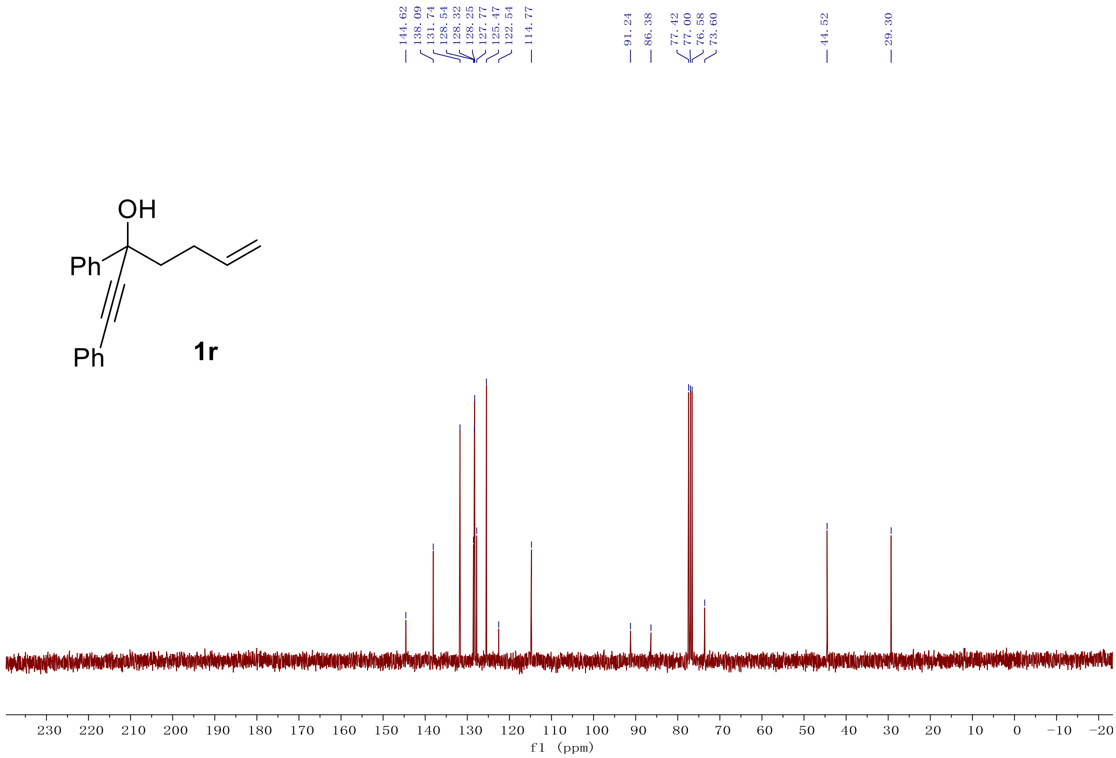

Feb03-2017.201.fid — TXj-656-C — carbon_256 CDCl3 /opt/topspin av1 32

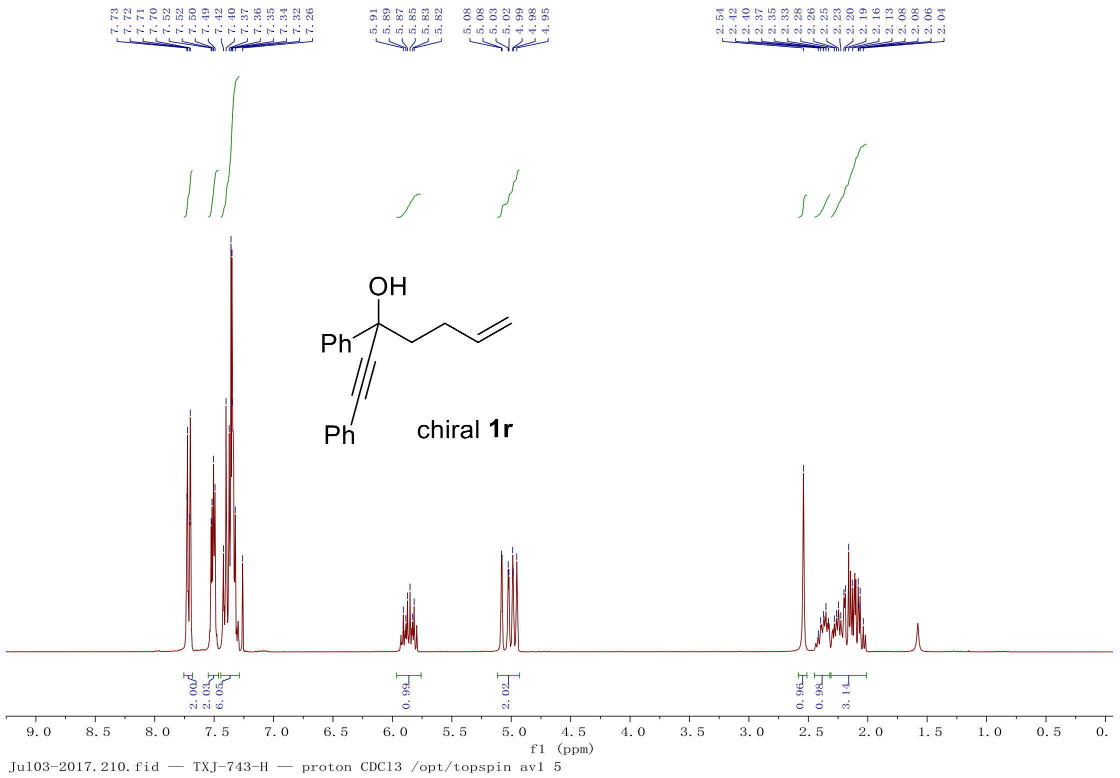
7.73 7.72 7.71 7.70 7.52 7.52 7.50 7.49 7.42 7.40 7.37 7.36 7.35 7.34 7.32 7.26
5.91 5.89 5.87 5.85 5.83 5.82
5.08 5.08 5.03 5.02 4.99 4.98 4.95
2.54 2.42 2.40 2.37 2.35 2.33 2.28 2.26 2.25 2.23 2.20 2.19 2.16 2.13 2.08 2.08 2.06 2.04
OH
Ph
Ph
chiral 1r
2.00 2.03 6.05
0.99
2.02
0.96 0.98 3.14
9.0 8.5 8.0 7.5 7.0 6.5 6.0 5.5 5.0 4.5 4.0 3.5 3.0 2.5 2.0 1.5 1.0 0.5 0.
f1 (ppm)
Jul03-2017.210.fid — TXJ-743-H — proton CDCl3 /opt/topspin av1 5

Data File C:\CHEM32\1\DATA\TANG\TXJ-656000001.D
Sample Name: TXJ-656

===

Acq. Operator : kischkewitz
Acq. Instrument : Instrument 1 Location : Vial 1
Injection Date : 11.05.2017 10:09:11
Inj Volume : 15.0 µl
Acq. Method : C:\CHEM32\1\METHODS\AK STUDER\STANDARD METHODS\M20_S.M
Last changed : 04.04.2017 13:39:48 by prekel
Analysis Method : C:\CHEM32\1\METHODS\AK STUDER\STANDARD METHODS\M1_S.M
Last changed : 11.05.2017 10:55:10 by kischkewitz
(modified after loading)
Method Info : standard method M1_S, solvent system 90:10, time 60 min, flow 1 mL/min, 25 °C, 15 microL injection vol.

Sample Info : OD-H

===

Instrument Conditions	:	At Start	At Stop	
Column Temp. (left)	:	25.0	25.0	°C
Column Temp. (right)	:	25.0	25.0	°C
Pressure	:	132.9	134.6	bar
Flow	:	1.000	1.000	ml/min

Detector Lamp Burn Times:		Current On-Time	Accumulated On-Time	
DAD 1, UV Lamp	:	0.27	3733.6	h
DAD 1, Visible Lamp	:	OFF	567.1	h

Solvent Description :
PMP1 , Solvent A : Cyclohexan
PMP1 , Solvent B : Cyclohexan:Isoprop 90:10

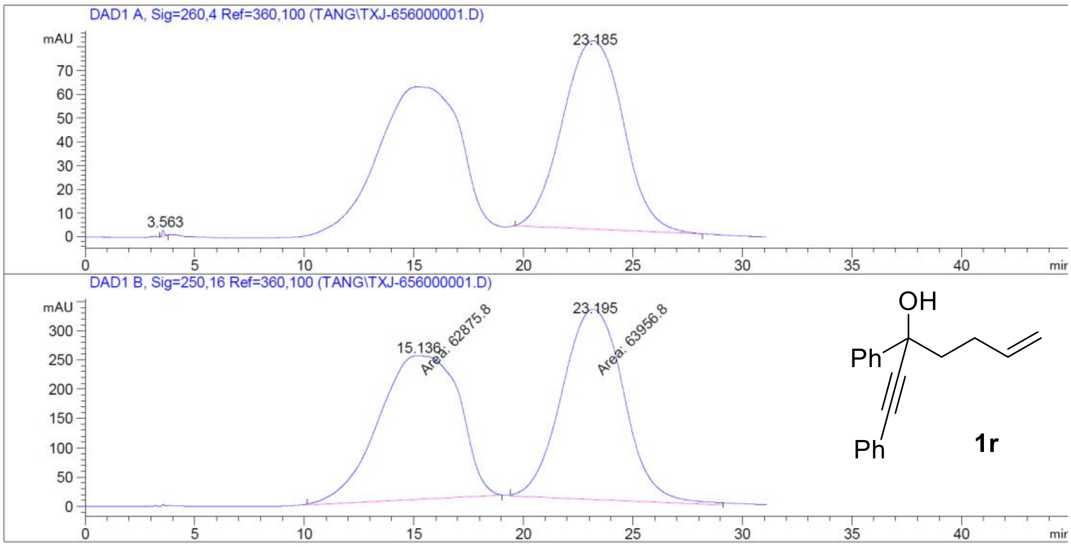

1r

Signal 2: DAD1 B, Sig=250,16 Ref=360,100

Peak #	RetTime [min]	Type	Width [min]	Area [mAU*s]	Height [mAU]	Area %
1	15.136	MM	4.2695	6.28758e4	245.44820	49.5738
2	23.195	MM	3.2754	6.39568e4	325.44220	50.4262

Totals : 1.26833e5 570.89040

```
Data File C:\CHEM32\1\DATA\TANG\TXJ-743000001.D
Sample Name: TXJ-743

    =====================================================================
    Acq. Operator   : kischkewitz
    Acq. Instrument : Instrument 1                    Location : Vial 2
    Injection Date  : 11.05.2017 10:56:21
                                                    Inj Volume : 15.0 µl
    Acq. Method     : C:\CHEM32\1\METHODS\AK STUDER\STANDARD METHODS\M20_S.M
    Last changed    : 11.05.2017 10:40:42 by kischkewitz
    Analysis Method : C:\CHEM32\1\METHODS\AK STUDER\STANDARD METHODS\M1_S.M
    Last changed    : 11.05.2017 12:56:08 by kischkewitz
                      (modified after loading)
    Method Info     : standard method M1_S, solvent system 90:10, time 60 min, flow 1 mL/min, 25
                      °C, 15 microL injection vol.

    Sample Info     : OD-H
```

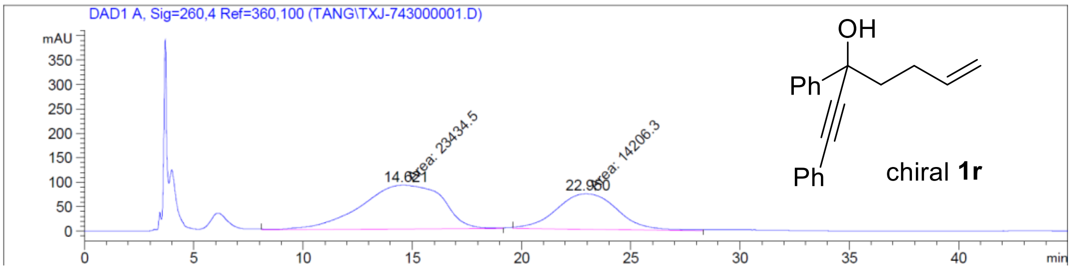

Signal 1: DAD1 A, Sig=260,4 Ref=360,100

Peak #	RetTime [min]	Type	Width [min]	Area [mAU*s]	Height [mAU]	Area %
1	14.621	MM	4.3488	2.34345e4	89.81277	62.2583
2	22.950	MM	3.2419	1.42063e4	73.03435	37.7417

Totals : 3.76407e4 162.84712

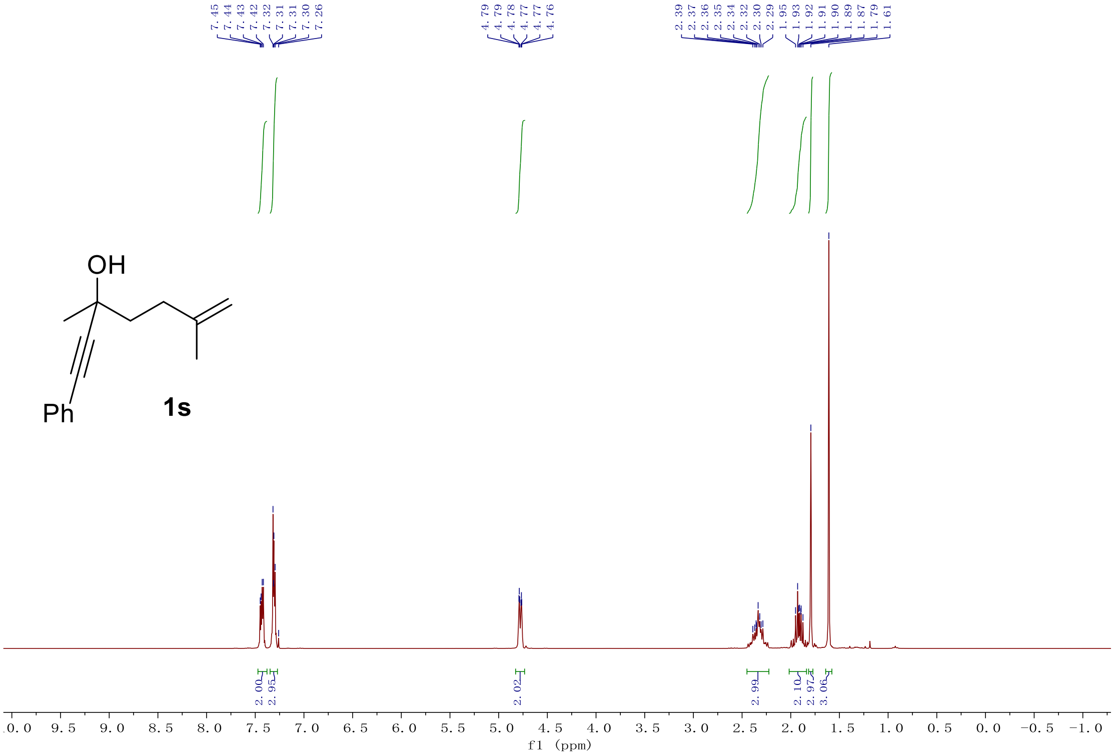

Feb01-2017.280.fid — TXJ-643-H — proton CDCl3 /opt/topspin av1 30

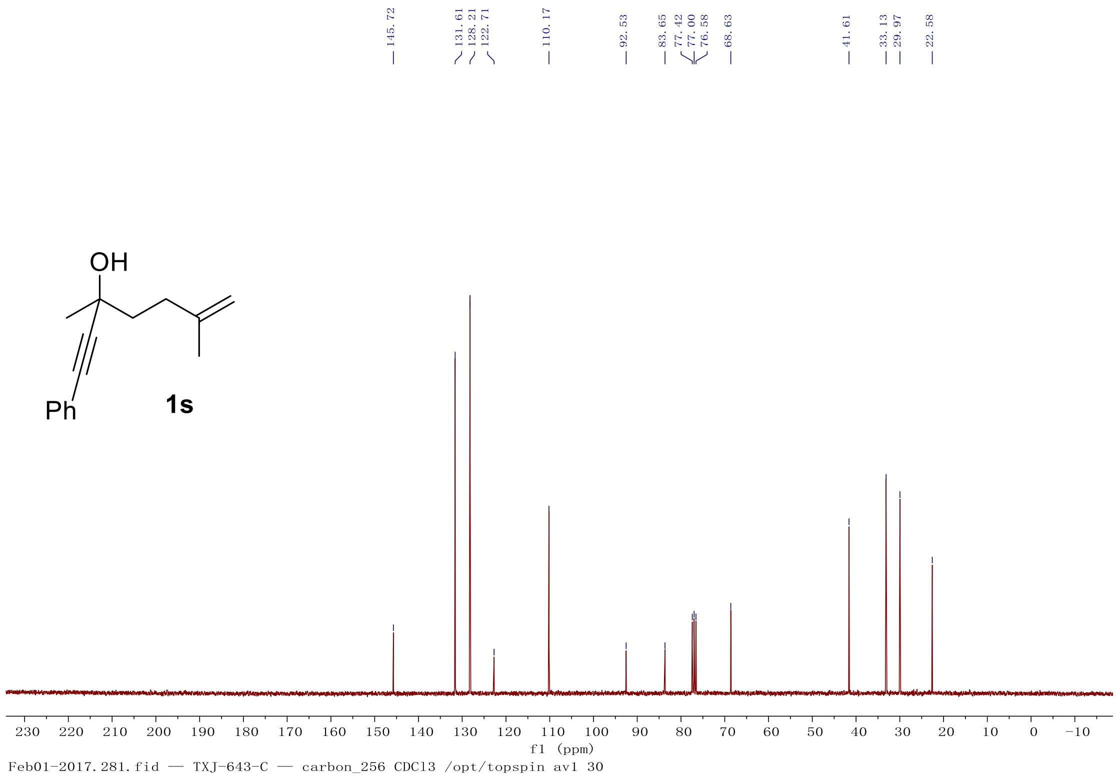

145.72
131.61
128.21
122.71
110.17
92.53
83.65
77.42
77.00
76.58
68.63
41.61
33.13
29.97
22.58
OH
Ph
1s
230 220 210 200 190 180 170 160 150 140 130 120 110 100 90 80 70 60 50 40 30 20 10 0 -10
f1 (ppm)
Feb01-2017.281.fid — TXJ-643-C — carbon_256 CDCl3 /opt/topspin av1 30

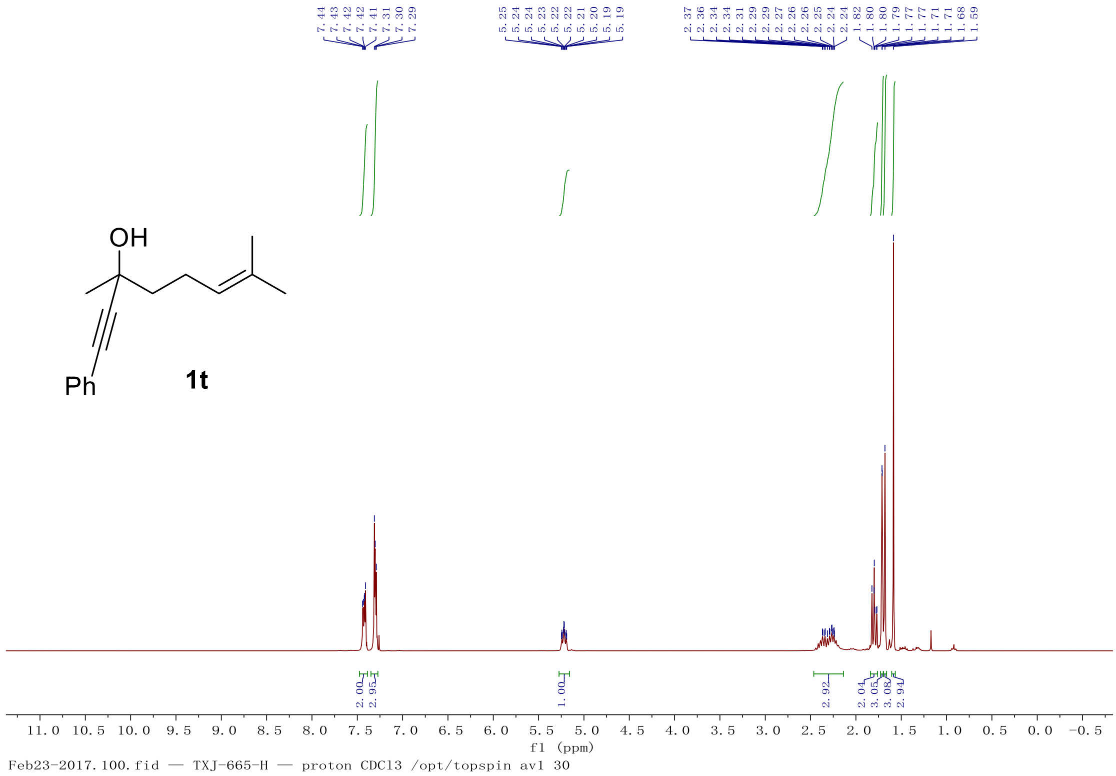

7.44
7.43
7.42
7.42
7.41
7.31
7.30
7.29
5.25
5.24
5.24
5.23
5.22
5.22
5.21
5.20
5.19
5.19
2.37
2.36
2.34
2.34
2.31
2.29
2.29
2.27
2.26
2.26
2.25
2.24
2.24
1.82
1.80
1.80
1.79
1.77
1.77
1.71
1.68
1.59
OH
Ph
1t
2.00
2.95
1.00
2.92
2.04
3.05
3.08
2.94
11.0 10.5 10.0 9.5 9.0 8.5 8.0 7.5 7.0 6.5 6.0 5.5 5.0 4.5 4.0 3.5 3.0 2.5 2.0 1.5 1.0 0.5 0.0 -0.5
f1 (ppm)
Feb23-2017.100.fid — TXJ-665-H — proton CDCl3 /opt/topspin av1 30

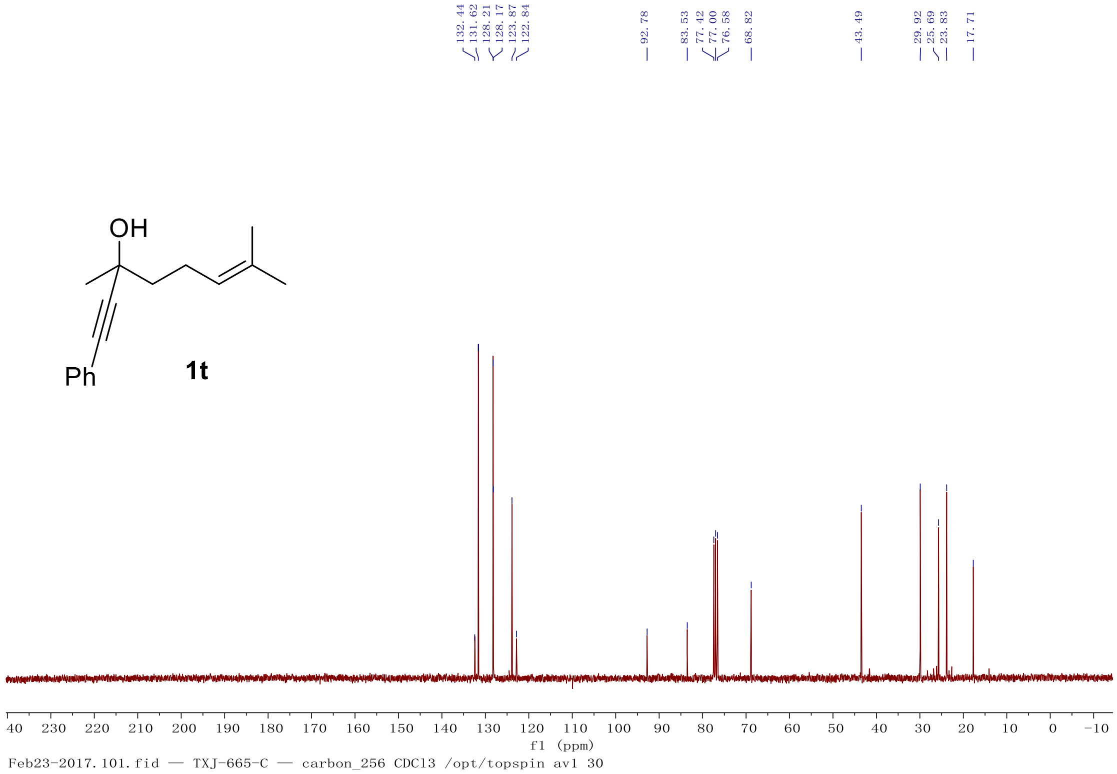
132.44
131.62
128.21
128.17
123.87
122.84
92.78
83.53
77.42
77.00
76.58
68.82
43.49
29.92
25.69
23.83
17.71
OH
Ph
1t
f1 (ppm)
Feb23-2017.101.fid — TXJ-665-C — carbon_256 CDCl3 /opt/topspin av1 30

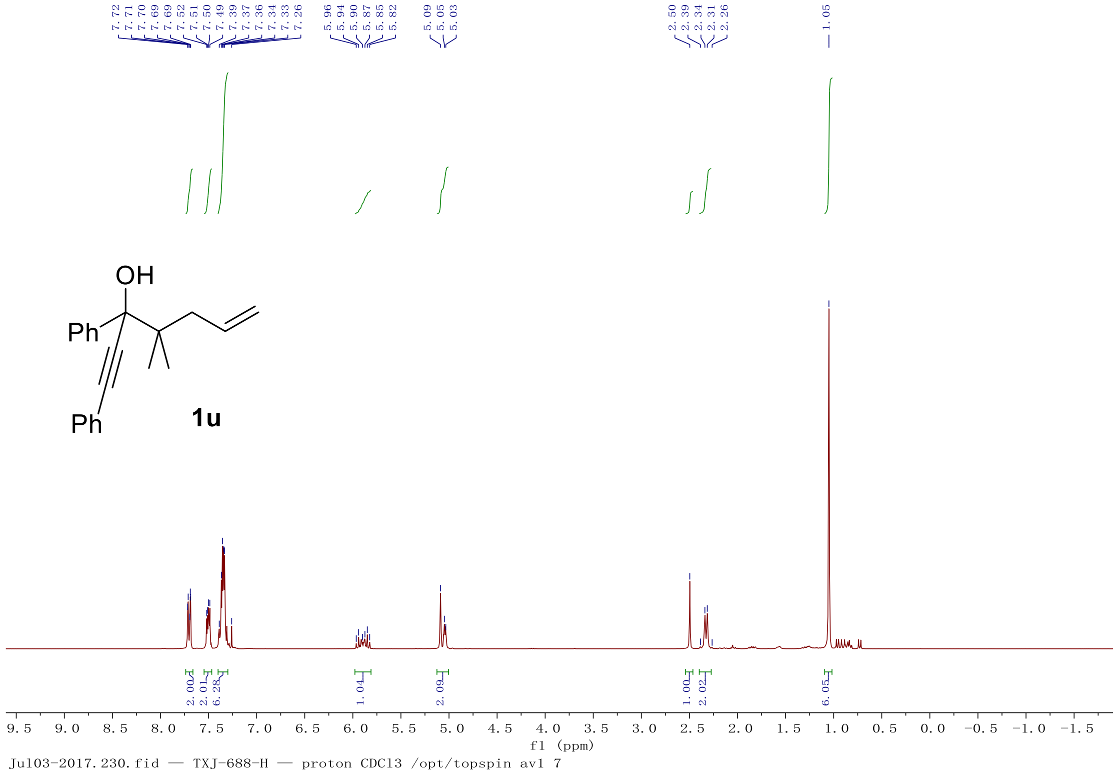
7.72
7.71
7.70
7.69
7.69
7.52
7.51
7.50
7.49
7.39
7.37
7.36
7.34
7.33
7.26
5.96
5.94
5.90
5.87
5.85
5.82
5.09
5.05
5.03
2.50
2.39
2.34
2.31
2.26
1.05
OH
Ph
Ph
1u
2.00
2.01
6.28
1.04
2.09
1.00
2.02
6.05
f1 (ppm)
Jul03-2017.230.fid — TXJ-688-H — proton CDCl3 /opt/topspin av1 7

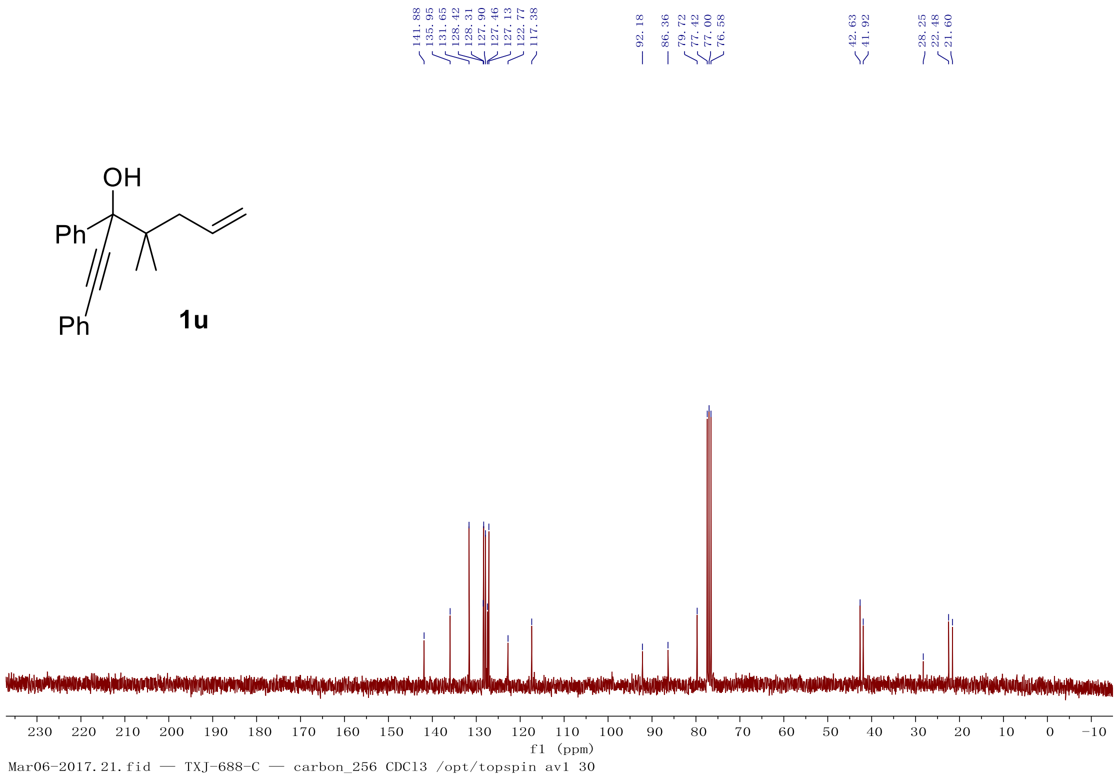
141.88
135.95
131.65
128.42
128.31
127.90
127.46
127.13
122.77
117.38
92.18
86.36
79.72
77.42
77.00
76.58
42.63
41.92
28.25
22.48
21.60
OH
Ph
Ph
1u
f1 (ppm)
Mar06-2017.21.fid — TXJ-688-C — carbon_256 CDCl3 /opt/topspin av1 30

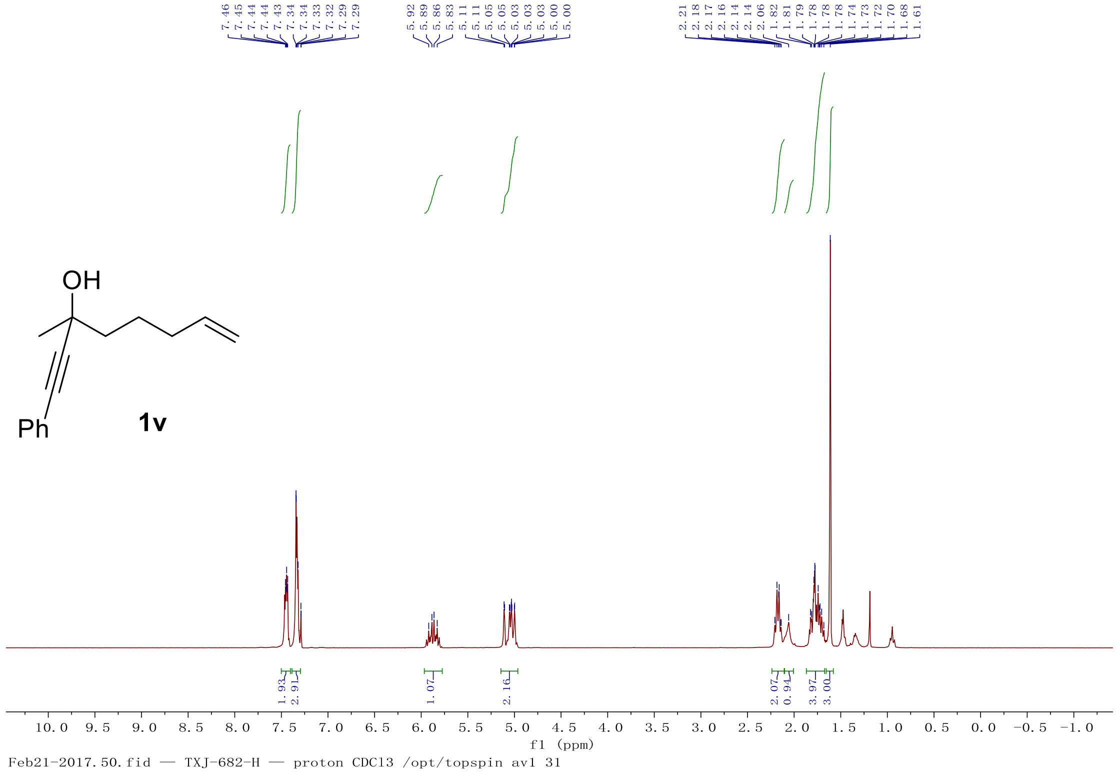
7.46
7.45
7.44
7.44
7.43
7.34
7.34
7.33
7.32
7.29
7.29
5.92
5.89
5.86
5.83
5.11
5.11
5.05
5.05
5.03
5.03
5.00
5.00
2.21
2.18
2.17
2.16
2.14
2.14
2.06
1.82
1.81
1.79
1.78
1.78
1.74
1.73
1.72
1.70
1.68
1.61
OH
Ph
1v
1.93
2.91
1.07
2.16
2.07
0.94
3.97
3.00
f1 (ppm)
Feb21-2017.50.fid — TXJ-682-H — proton CDCl3 /opt/topspin av1 31

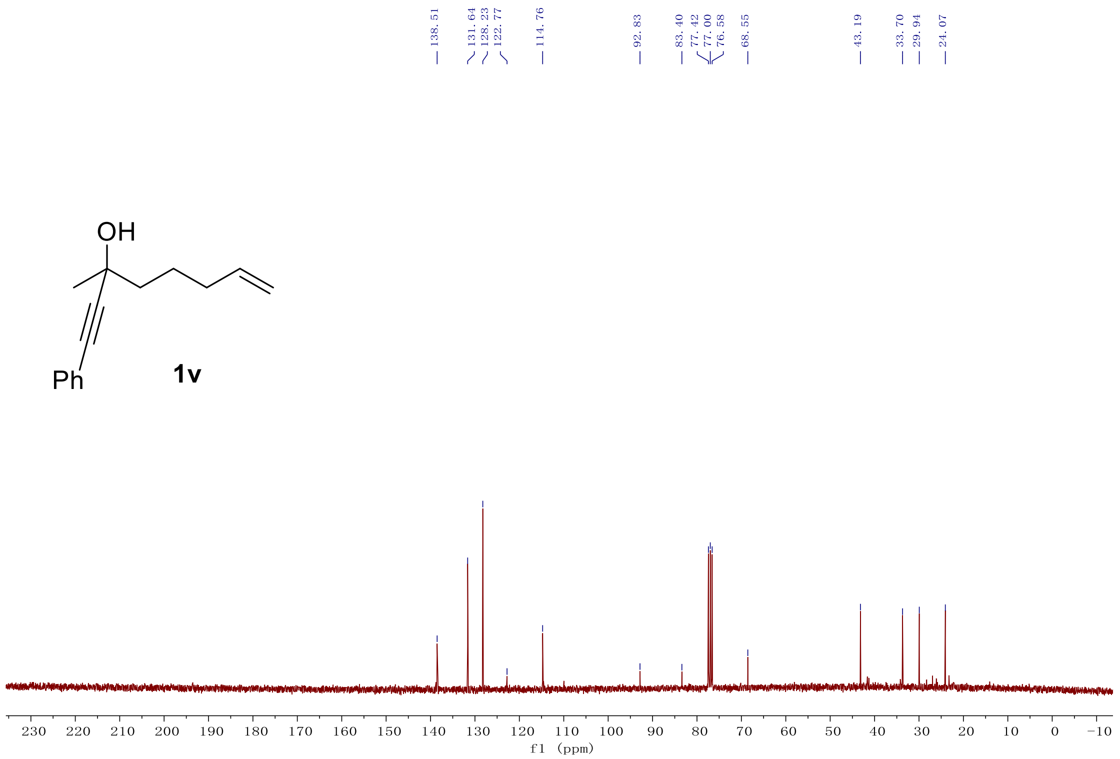

Feb21-2017.51.fid — TXJ-682-C — carbon_256 CDCl3 /opt/topspin av1 31

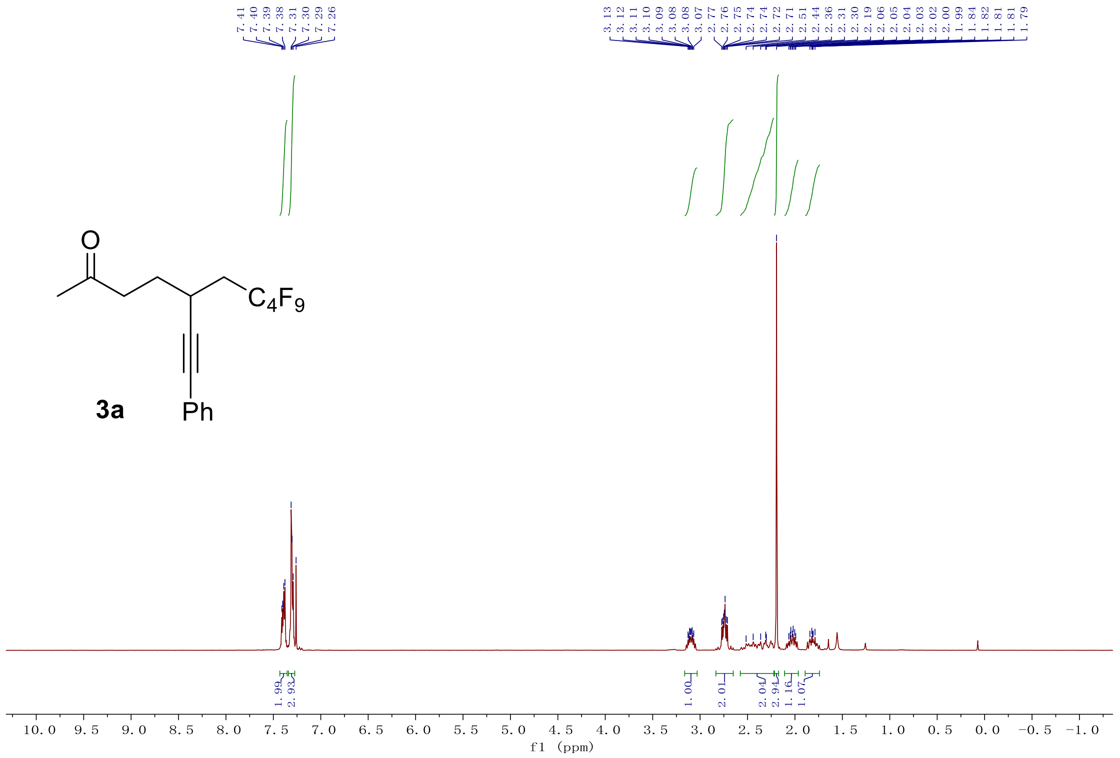

Dec22-2016.70.fid — stu tang txj 551p — proton CDCl3 /opt/topspin av1 26

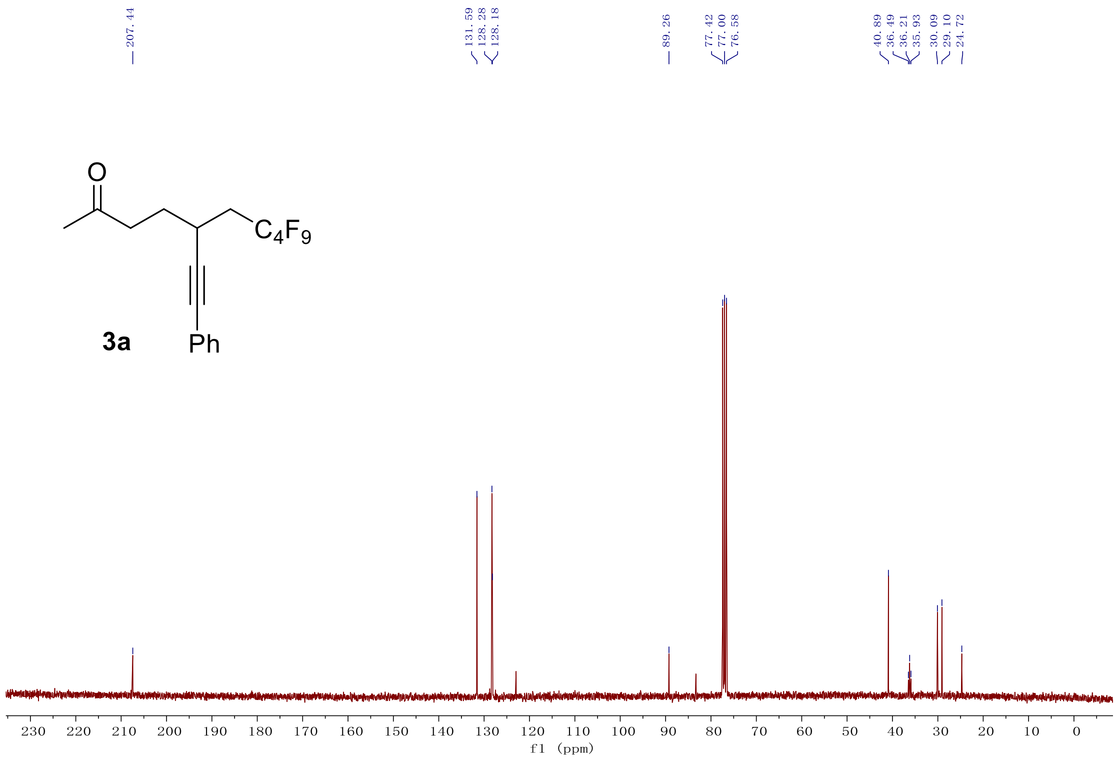

Dec23-2016.131.fid — TXJ-551-C — carbon CDCl3 /opt/topspin av1 31

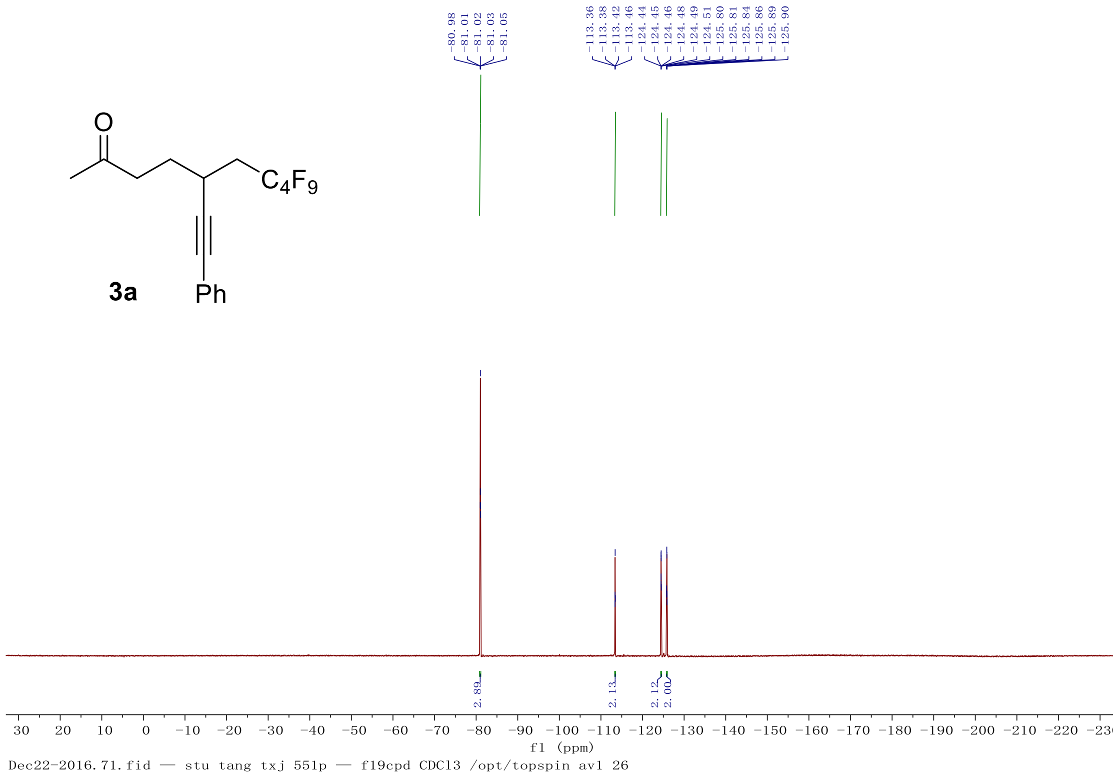

Dec22-2016.71.fid — stu tang txj 551p — f19cpd CDCl3 /opt/topspin av1 26

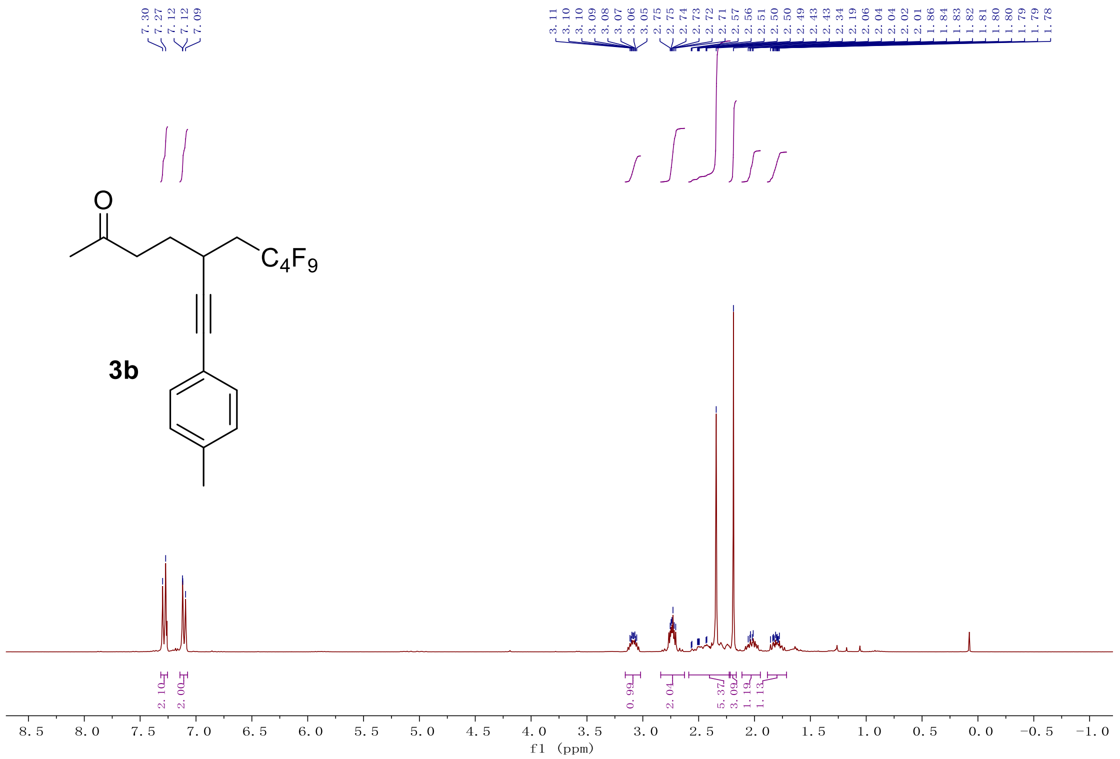

Jan12-2017.420.fid — TXJ-597-H — proton CDCl3 /opt/topspin av1 34

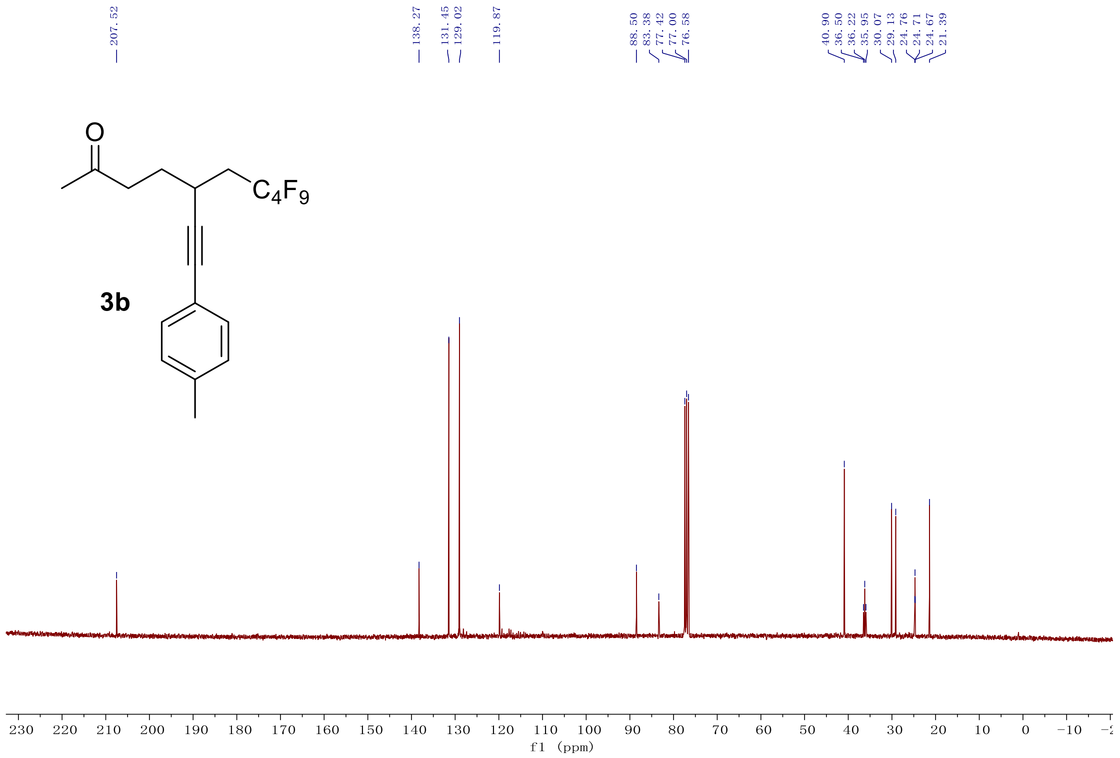

Jan12-2017.421.fid — TXJ-597-C — carbon CDCl3 /opt/topspin av1 34

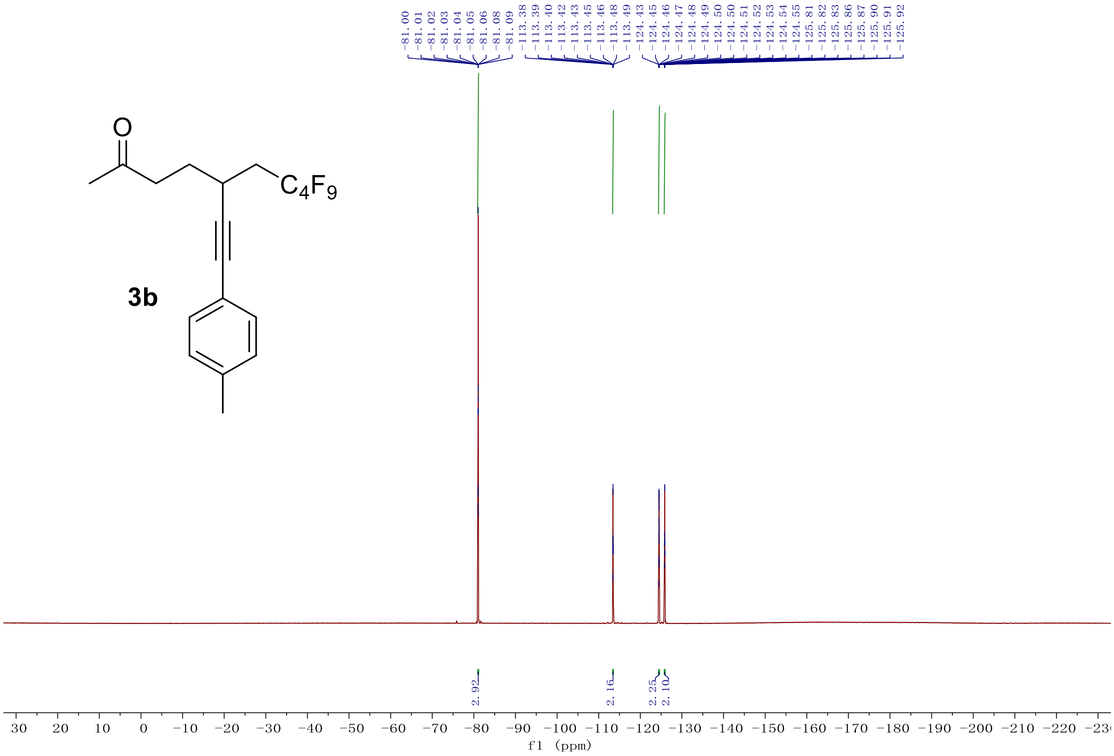

Jan12-2017.425.fid — TXJ-597-F — f19cpd CDCl3 /opt/topspin av1 34

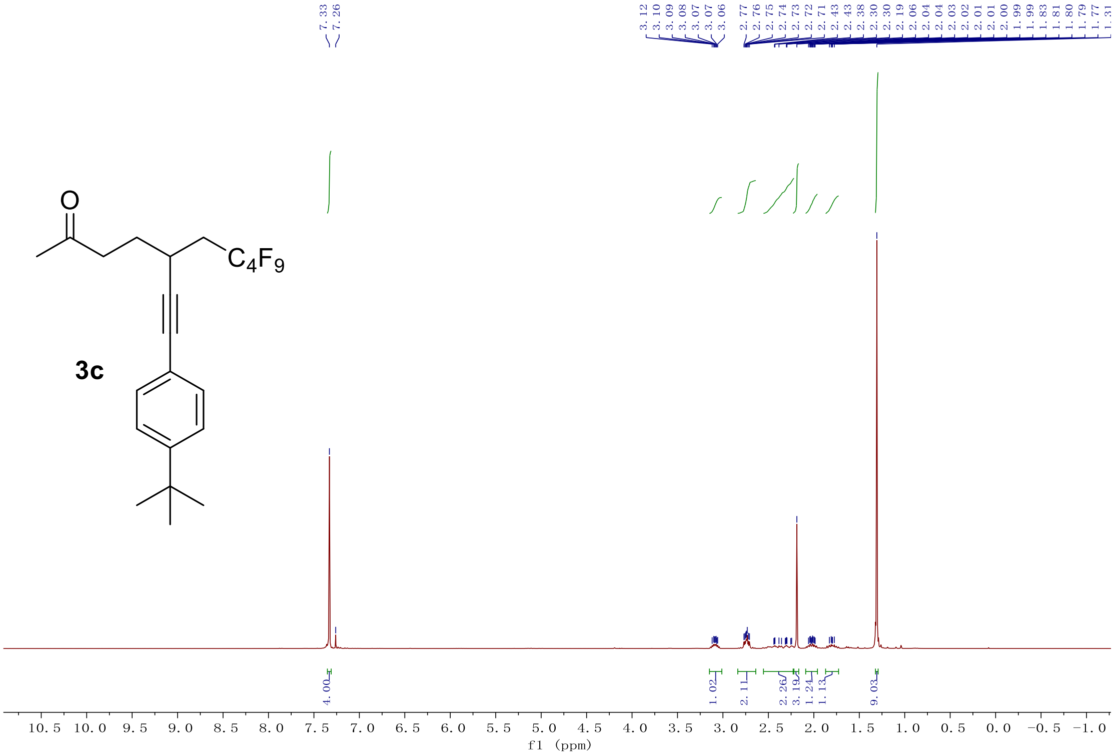

Jan25-2017.554.fid — TXJ-638-H — proton CDCl3 /opt/topspin av1 42

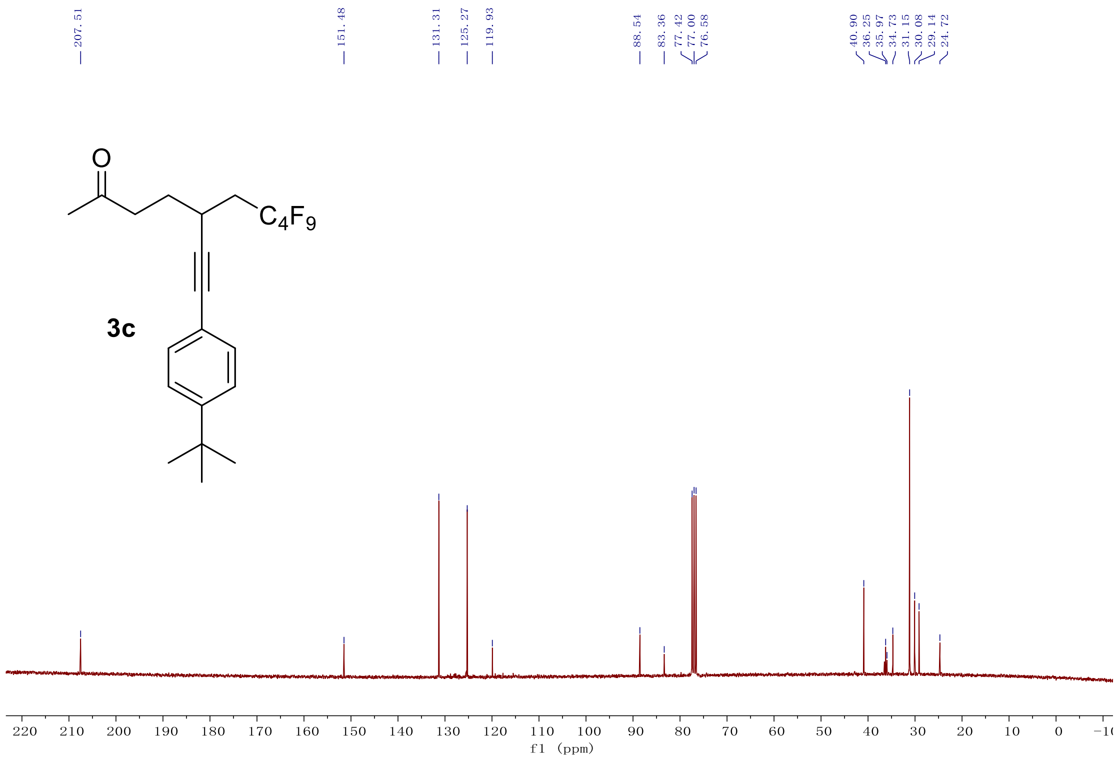

Jan25-2017.551.fid — TXJ-638-C — carbon CDCl3 /opt/topspin av1 42

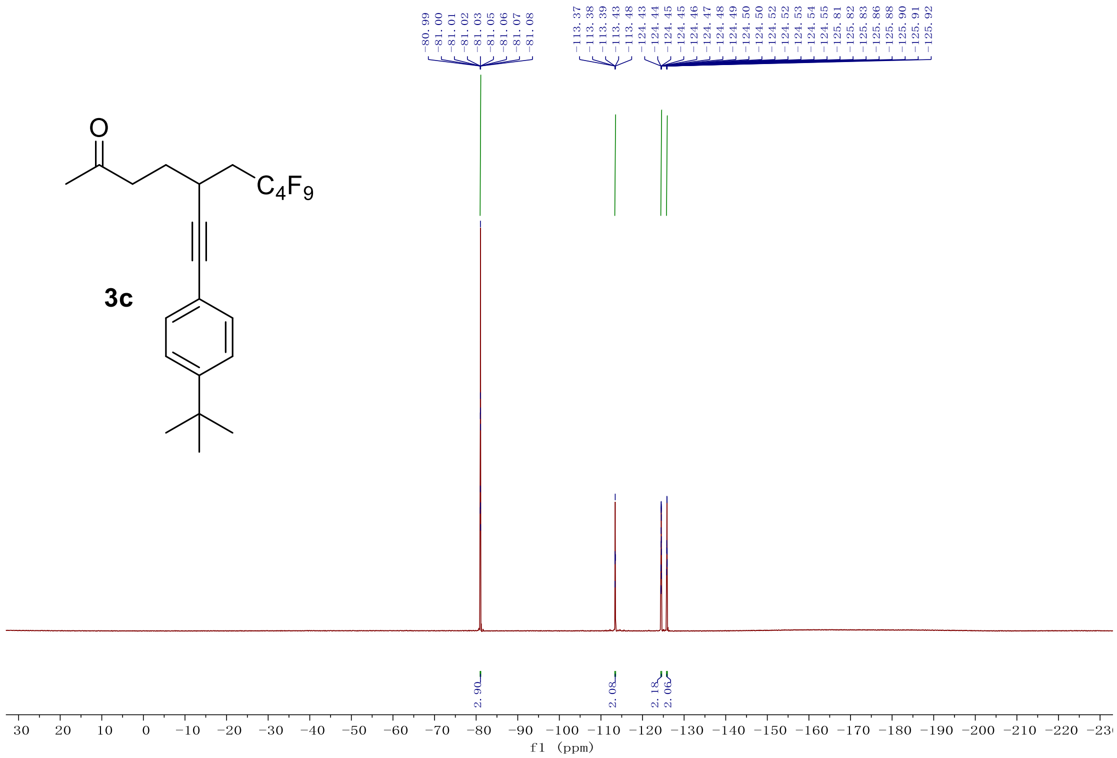

Jan25-2017.555.fid — TXJ-638-F — f19cpd CDCl3 /opt/topspin av1 42

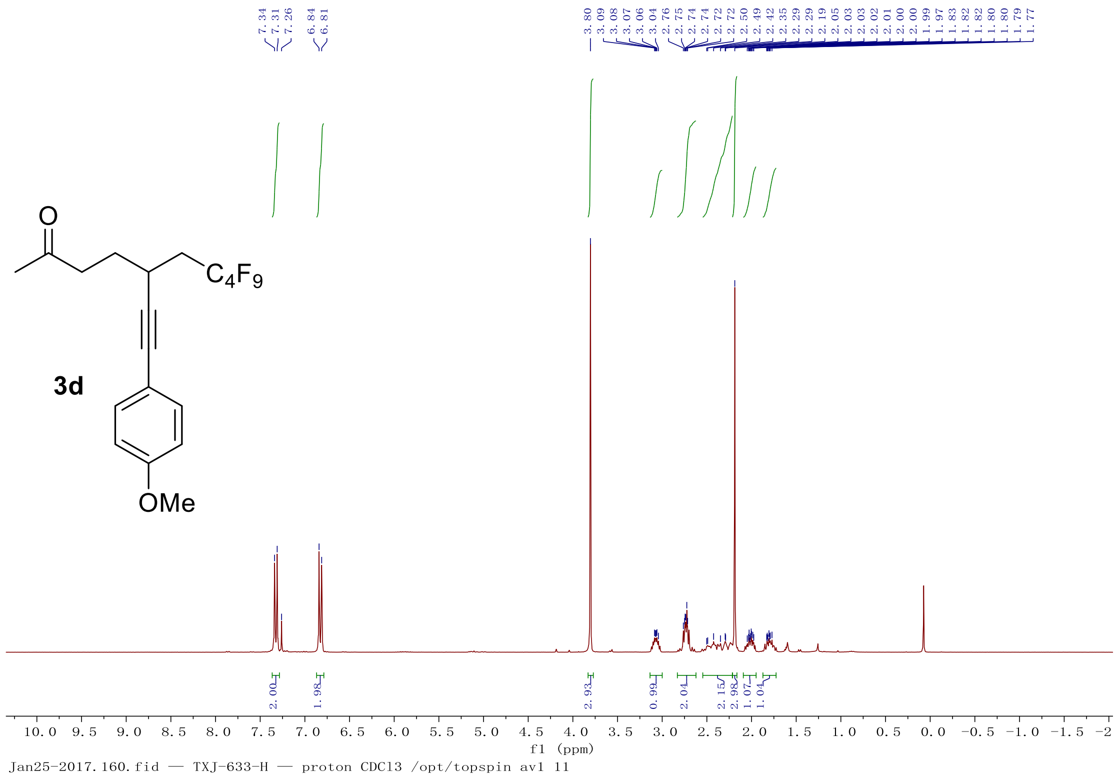

7.34
7.31
7.26
6.84
6.81
3.80
3.09
3.08
3.07
3.06
3.04
2.76
2.75
2.74
2.74
2.72
2.72
2.50
2.49
2.42
2.35
2.29
2.29
2.19
2.05
2.03
2.02
2.01
2.00
1.99
1.97
1.83
1.82
1.80
1.79
1.77
C4F9
3d
OMe
2.00
1.98
2.93
0.99
2.04
2.15
2.98
1.07
1.04
f1 (ppm)
Jan25-2017.160.fid — TXJ-633-H — proton CDCl3 /opt/topspin av1 11

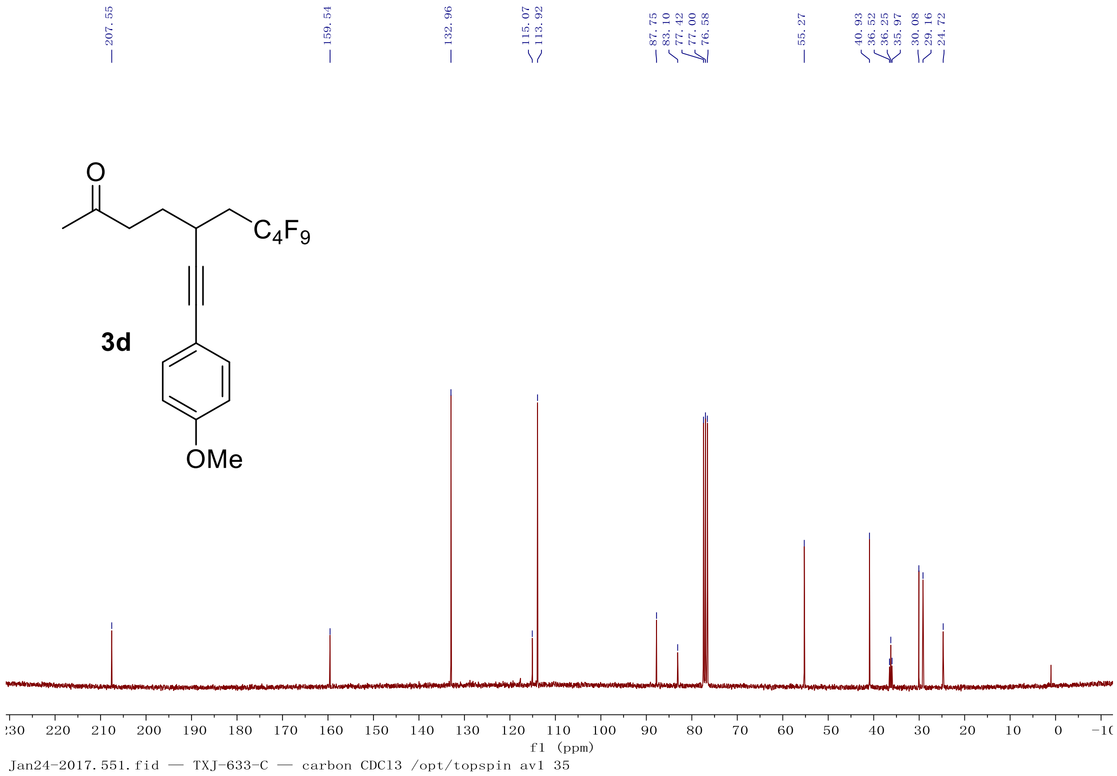

207.55
159.54
132.96
115.07
113.92
87.75
83.10
77.42
77.00
76.58
55.27
40.93
36.52
36.25
35.97
30.08
29.16
24.72
C4F9
3d
OMe
f1 (ppm)
Jan24-2017.551.fid — TXJ-633-C — carbon CDCl3 /opt/topspin av1 35

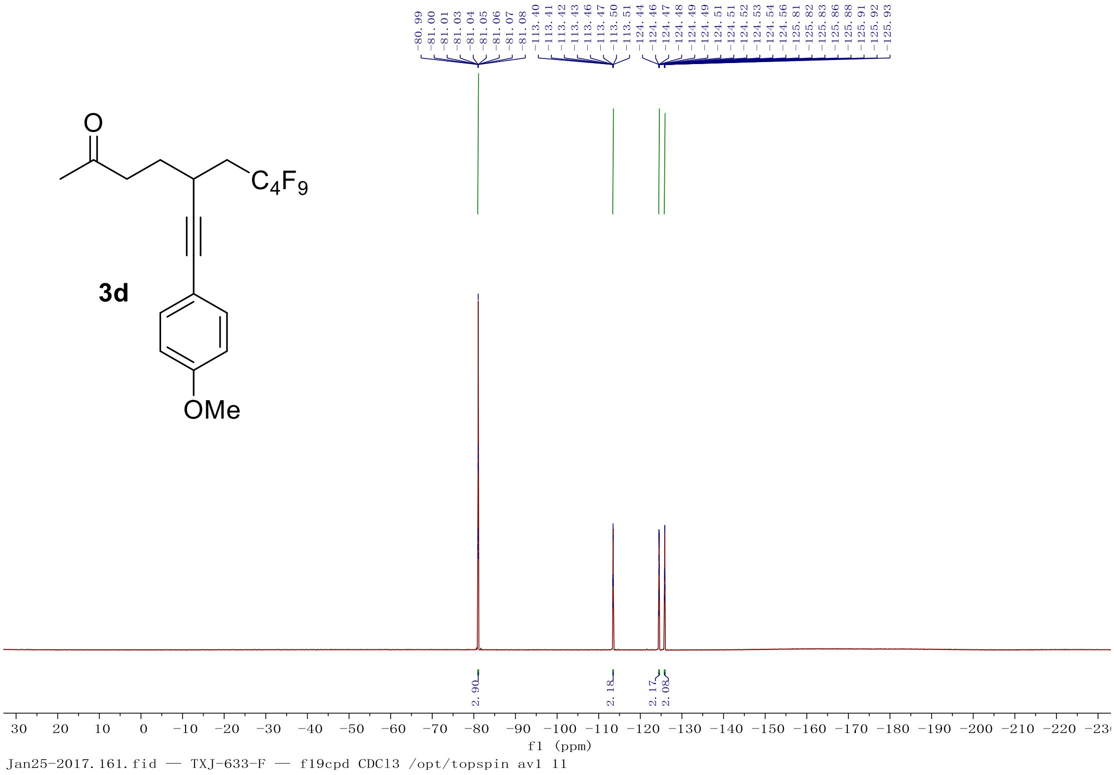
-80.99
-81.00
-81.01
-81.03
-81.04
-81.05
-81.06
-81.07
-81.08
-113.40
-113.41
-113.42
-113.43
-113.46
-113.47
-113.50
-113.51
-124.44
-124.46
-124.47
-124.48
-124.49
-124.51
-124.52
-124.53
-124.54
-124.56
-125.81
-125.82
-125.83
-125.86
-125.88
-125.91
-125.92
-125.93
O
C4F9
3d
OMe
2.90
2.18
2.17
2.08
30 20 10 0 -10 -20 -30 -40 -50 -60 -70 -80 -90 -100 -110 -120 -130 -140 -150 -160 -170 -180 -190 -200 -210 -220
f1 (ppm)
Jan25-2017.161.fid — TXJ-633-F — f19cpd CDCl3 /opt/topspin av1 11

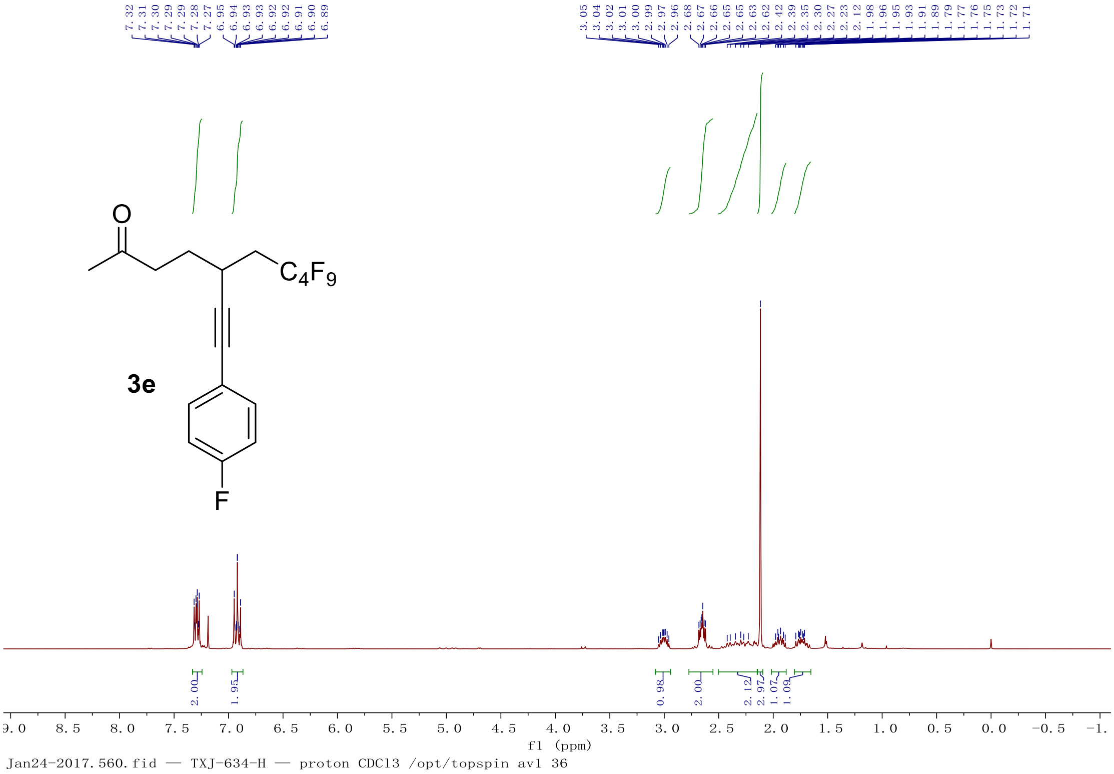
7.32
7.31
7.30
7.29
7.29
7.28
7.27
6.95
6.94
6.93
6.93
6.92
6.92
6.91
6.90
6.89
3.05
3.04
3.02
3.01
3.00
2.99
2.97
2.96
2.68
2.67
2.66
2.65
2.63
2.62
2.42
2.39
2.35
2.30
2.27
2.23
2.12
1.98
1.96
1.95
1.93
1.91
1.89
1.79
1.77
1.76
1.75
1.73
1.72
1.71
O
C4F9
3e
F
2.00
1.95
0.98
2.00
2.12
2.97
1.07
1.09
8.5 8.0 7.5 7.0 6.5 6.0 5.5 5.0 4.5 4.0 3.5 3.0 2.5 2.0 1.5 1.0 0.5 0.0 -0.5
f1 (ppm)
Jan24-2017.560.fid — TXJ-634-H — proton CDCl3 /opt/topspin av1 36

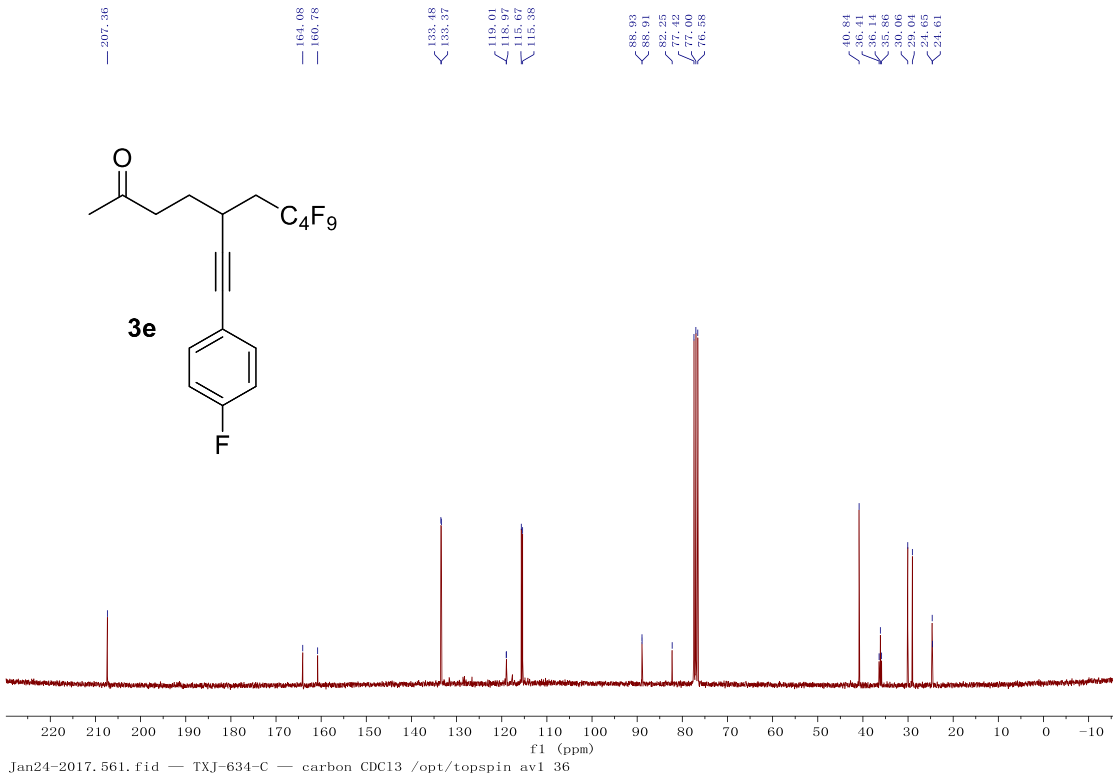
3e
Jan24-2017.561.fid — TXJ-634-C — carbon CDCl3 /opt/topspin av1 36

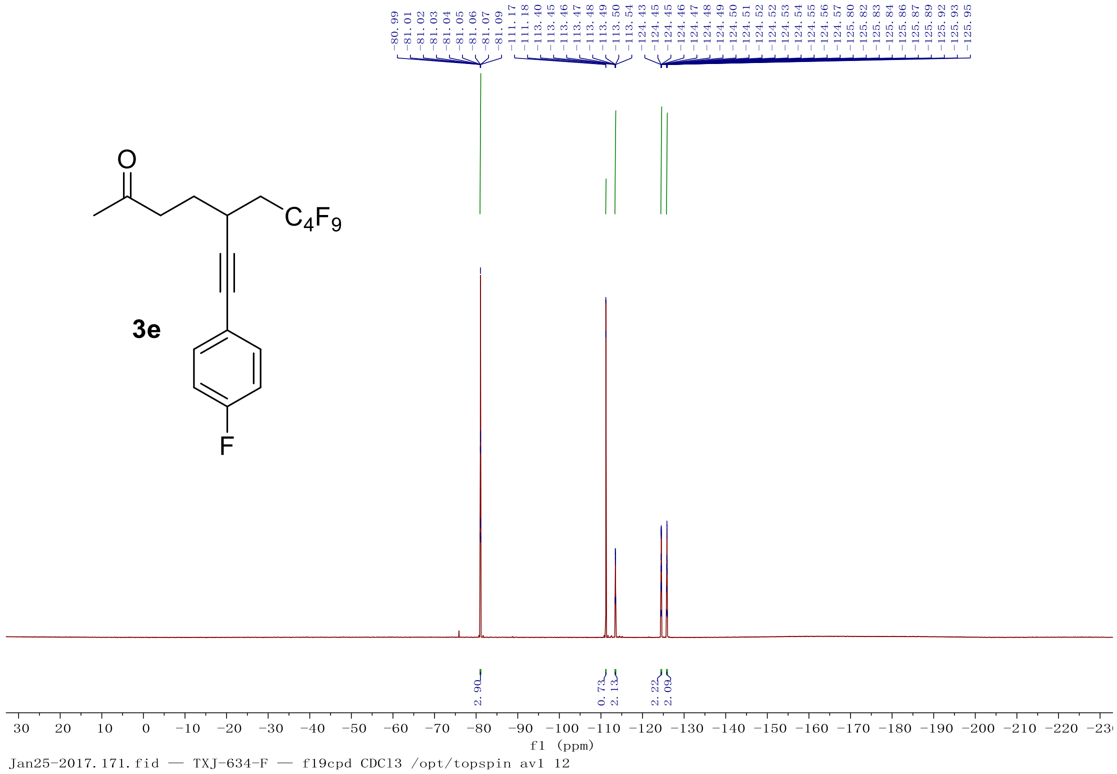
3e
Jan25-2017.171.fid — TXJ-634-F — f19cpd CDCl3 /opt/topspin av1 12

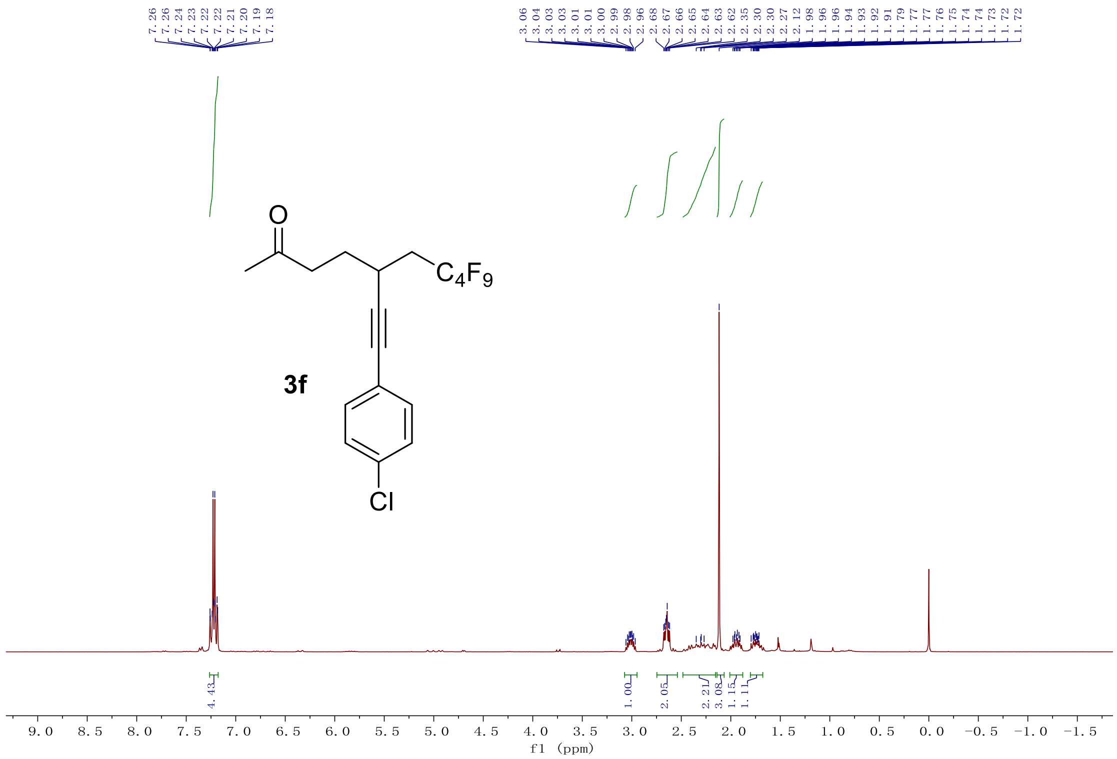

Jan25-2017.544.fid — TXJ-637-H — proton CDCl3 /opt/topspin av1 41

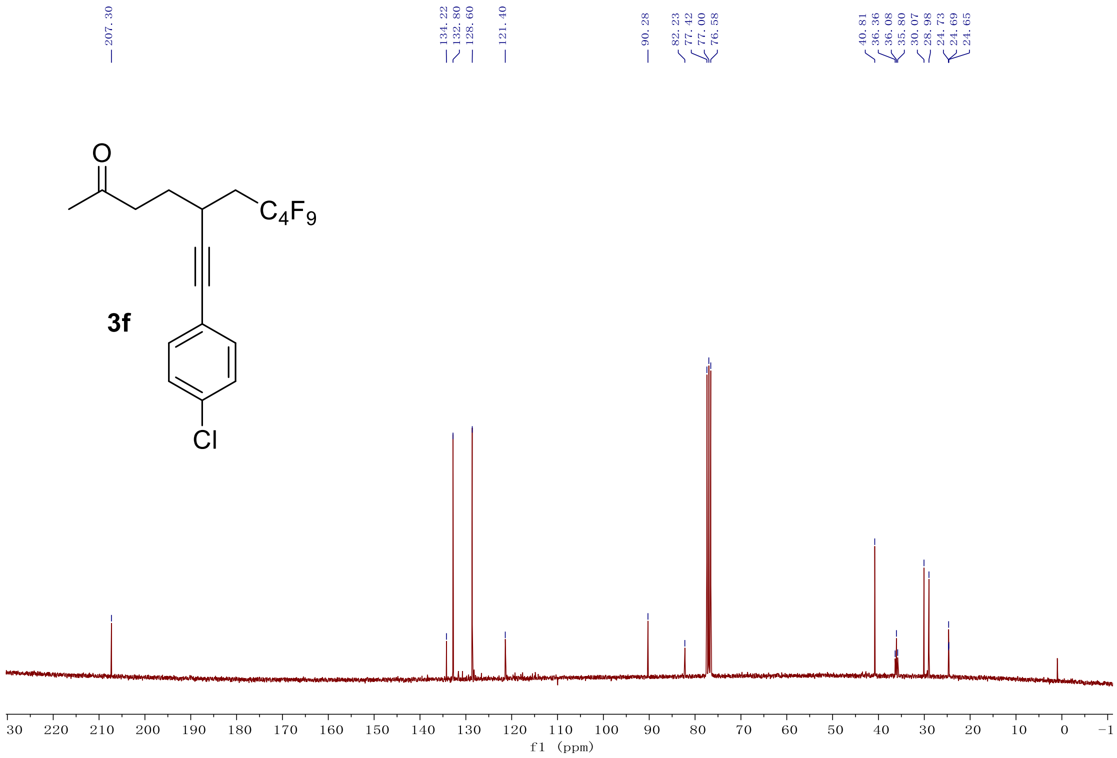

Jan25-2017.541.fid — TXJ-637-C — carbon CDCl3 /opt/topspin av1 41

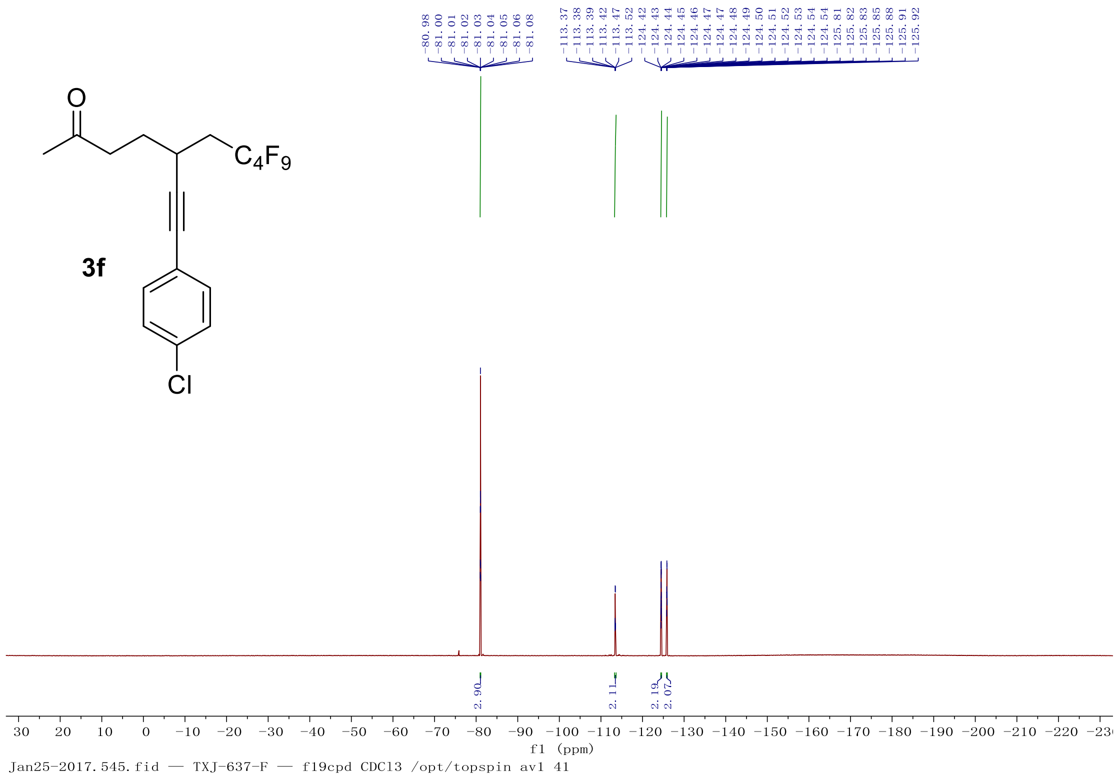

3f
-80.98
-81.00
-81.01
-81.02
-81.03
-81.04
-81.05
-81.06
-81.08
-113.37
-113.38
-113.39
-113.42
-113.47
-113.52
-124.42
-124.43
-124.44
-124.45
-124.46
-124.47
-124.47
-124.48
-124.49
-124.50
-124.51
-124.52
-124.53
-124.54
-124.54
-125.81
-125.82
-125.83
-125.85
-125.88
-125.91
-125.92
2.90
2.11
2.19
2.07
f1 (ppm)
Jan25-2017.545.fid — TXJ-637-F — f19cpd CDCl3 /opt/topspin av1 41

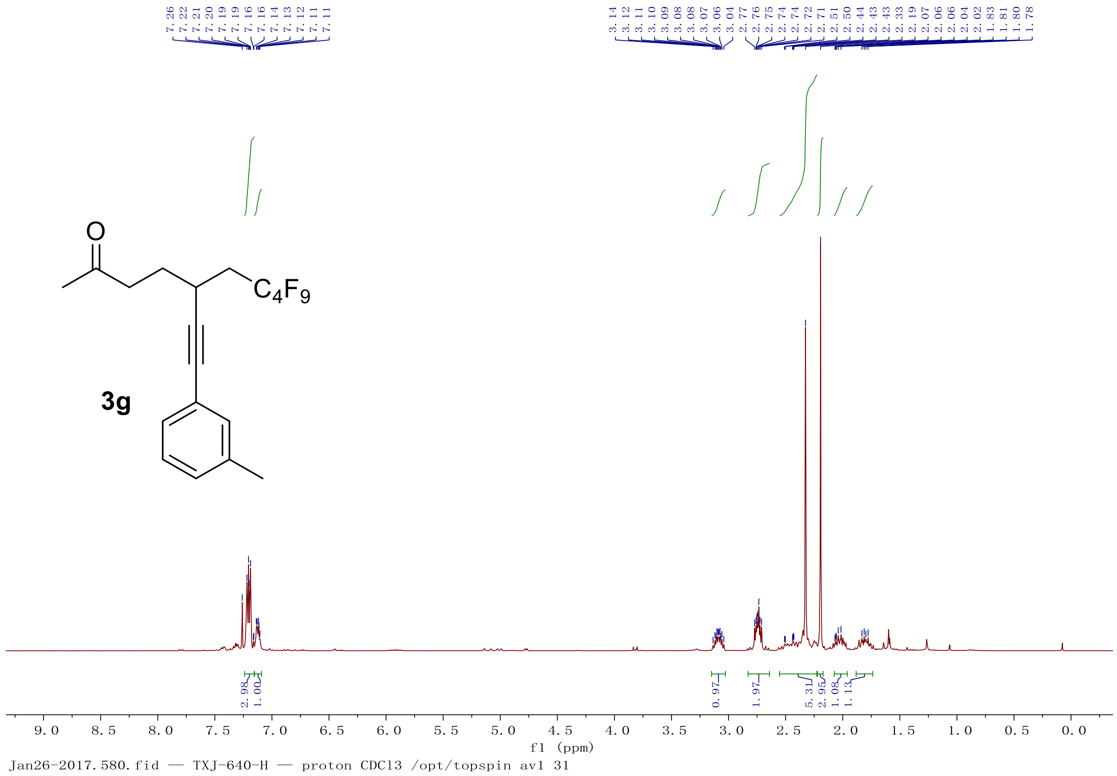

3g
7.26
7.22
7.21
7.20
7.19
7.19
7.16
7.16
7.14
7.13
7.12
7.11
7.11
3.14
3.12
3.11
3.10
3.09
3.08
3.08
3.07
3.06
3.04
2.77
2.76
2.75
2.74
2.74
2.72
2.71
2.51
2.50
2.44
2.43
2.43
2.33
2.19
2.07
2.06
2.04
2.02
1.83
1.81
1.80
1.78
2.98
1.00
0.97
1.97
5.31
2.95
1.08
1.13
f1 (ppm)
Jan26-2017.580.fid — TXJ-640-H — proton CDCl3 /opt/topspin av1 31

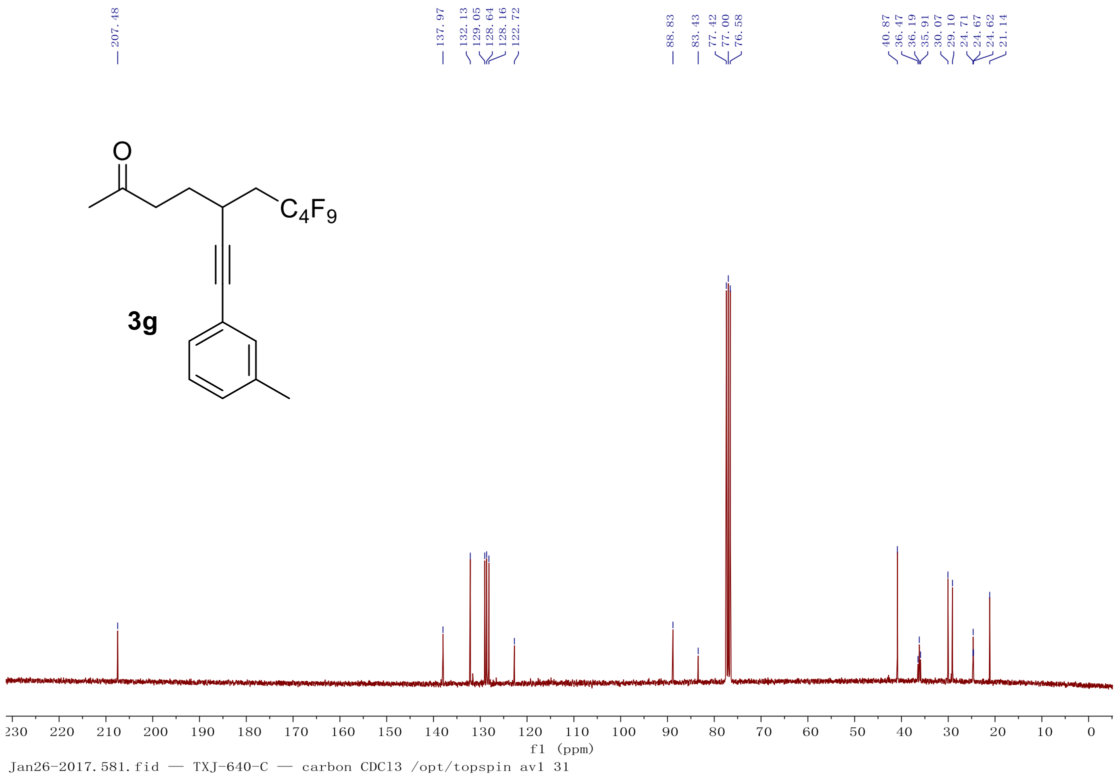

207.48
137.97
132.13
129.05
128.64
128.16
122.72
88.83
83.43
77.42
77.00
76.58
40.87
36.47
36.19
35.91
30.07
29.10
24.71
24.67
24.62
21.14
O
C4F9
3g
230 220 210 200 190 180 170 160 150 140 130 120 110 100 90 80 70 60 50 40 30 20 10 0
f1 (ppm)
Jan26-2017.581.fid — TXJ-640-C — carbon CDCl3 /opt/topspin av1 31

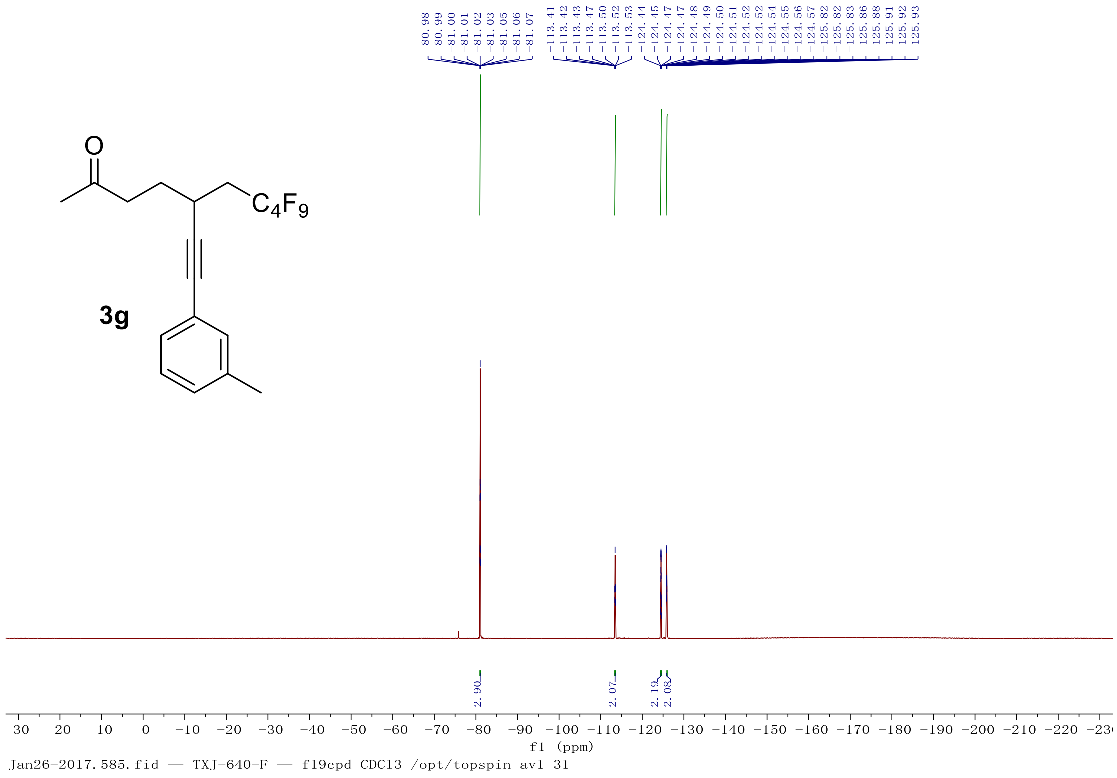

-80.98
-80.99
-81.00
-81.01
-81.02
-81.03
-81.05
-81.06
-81.07
-113.41
-113.42
-113.43
-113.47
-113.50
-113.52
-113.53
-124.44
-124.45
-124.47
-124.47
-124.48
-124.49
-124.50
-124.51
-124.52
-124.52
-124.54
-124.55
-124.56
-124.57
-125.82
-125.82
-125.83
-125.86
-125.88
-125.91
-125.92
-125.93
O
C4F9
3g
2.90
2.07
2.19
2.08
30 20 10 0 -10 -20 -30 -40 -50 -60 -70 -80 -90 -100 -110 -120 -130 -140 -150 -160 -170 -180 -190 -200 -210 -220
f1 (ppm)
Jan26-2017.585.fid — TXJ-640-F — f19cpd CDCl3 /opt/topspin av1 31

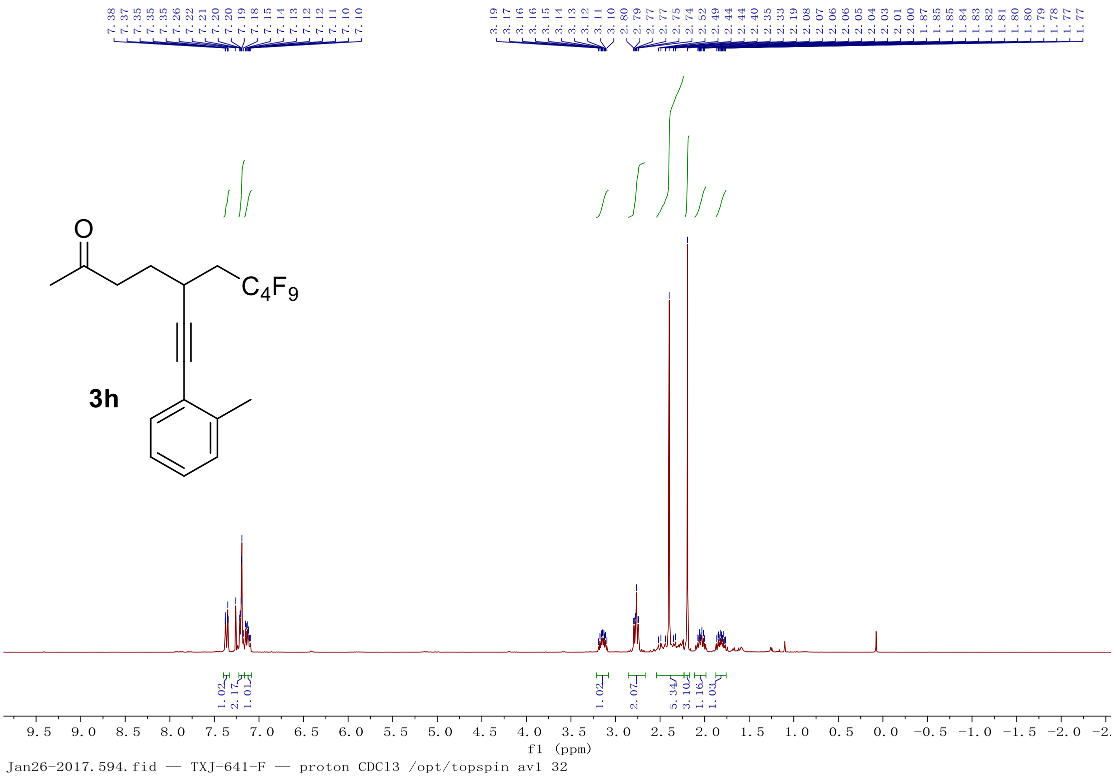

7.38 7.37 7.35 7.35 7.35 7.26 7.22 7.21 7.20 7.20 7.19 7.18 7.15 7.14 7.13 7.12 7.12 7.11 7.10 7.10
3.19 3.17 3.16 3.15 3.14 3.13 3.12 3.11 3.10 2.80 2.79 2.77 2.75 2.74 2.52 2.49 2.44 2.44 2.40 2.35 2.33 2.19 2.08 2.07 2.06 2.06 2.05 2.04 2.03 2.01 2.00 1.87 1.85 1.85 1.84 1.83 1.82 1.81 1.80 1.80 1.79 1.78 1.77 1.77
O
C_4F_9
3h
1.02 2.17 1.01
1.02 2.07 5.34 3.10 1.16 1.03
9.5 9.0 8.5 8.0 7.5 7.0 6.5 6.0 5.5 5.0 4.5 4.0 3.5 3.0 2.5 2.0 1.5 1.0 0.5 0.0 -0.5 -1.0 -1.5 -2.0 -2.
f1 (ppm)
Jan26-2017.594.fid — TXJ-641-F — proton CDCl3 /opt/topspin av1 32

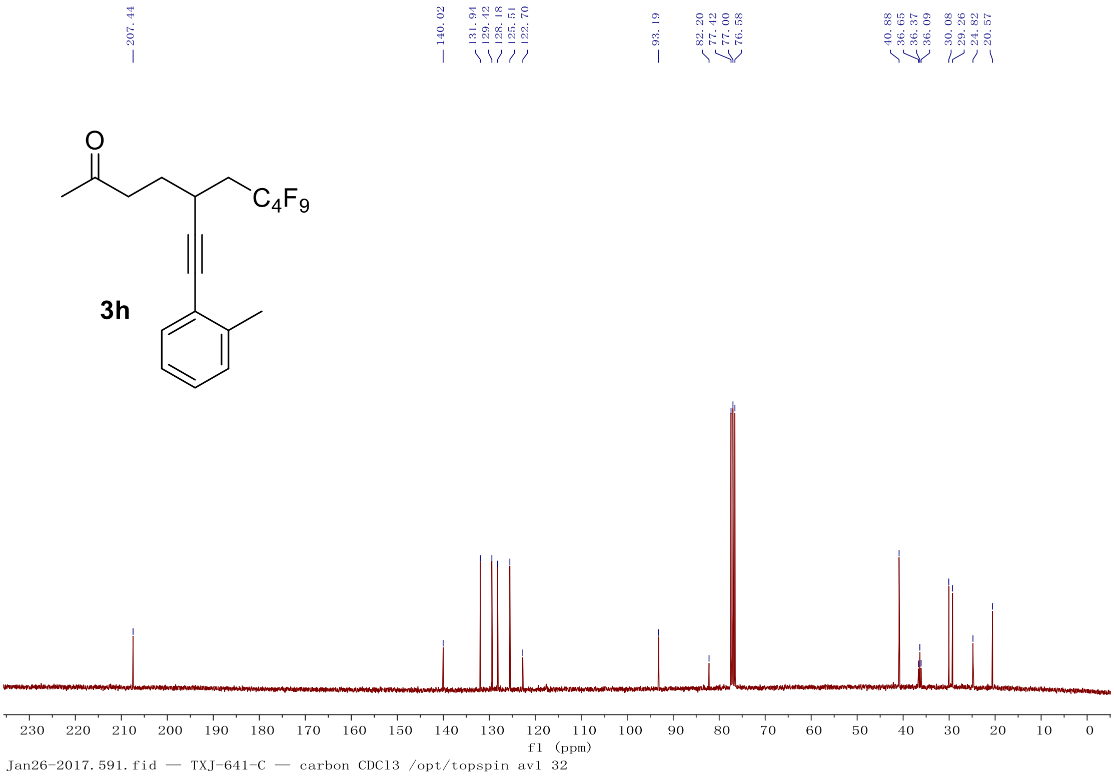

207.44
140.02 131.94 129.42 128.18 125.51 122.70
93.19
82.20 77.42 77.00 76.58
40.88 36.65 36.37 36.09 30.08 29.26 24.82 20.57
O
C_4F_9
3h
230 220 210 200 190 180 170 160 150 140 130 120 110 100 90 80 70 60 50 40 30 20 10 0
f1 (ppm)
Jan26-2017.591.fid — TXJ-641-C — carbon CDCl3 /opt/topspin av1 32

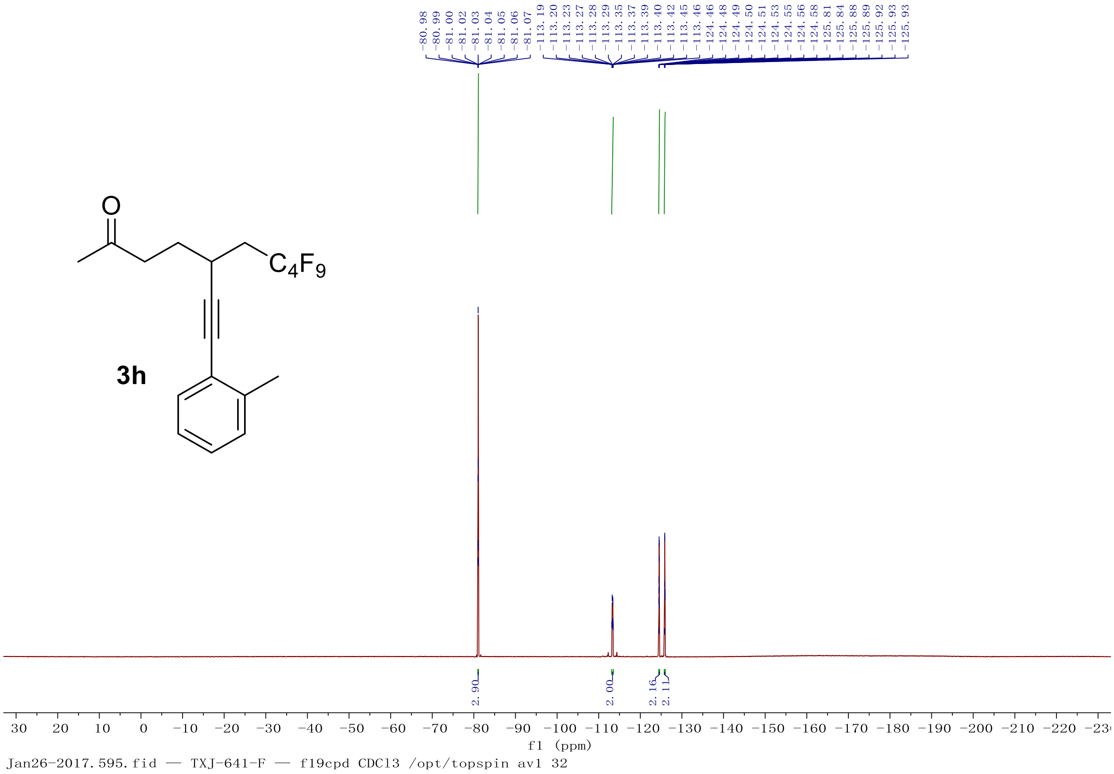
-80.98 -80.99 -81.00 -81.02 -81.03 -81.04 -81.05 -81.06 -81.07 -113.19 -113.20 -113.23 -113.27 -113.28 -113.29 -113.35 -113.37 -113.39 -113.40 -113.42 -113.45 -113.46 -124.46 -124.48 -124.49 -124.50 -124.51 -124.53 -124.55 -124.56 -124.58 -125.81 -125.84 -125.88 -125.89 -125.92 -125.93 -125.93
O
C4F9
3h
2.90 2.00 2.16 2.11
30 20 10 0 -10 -20 -30 -40 -50 -60 -70 -80 -90 -100 -110 -120 -130 -140 -150 -160 -170 -180 -190 -200 -210 -220
f1 (ppm)
Jan26-2017.595.fid — TXJ-641-F — f19cpd CDCl3 /opt/topspin av1 32

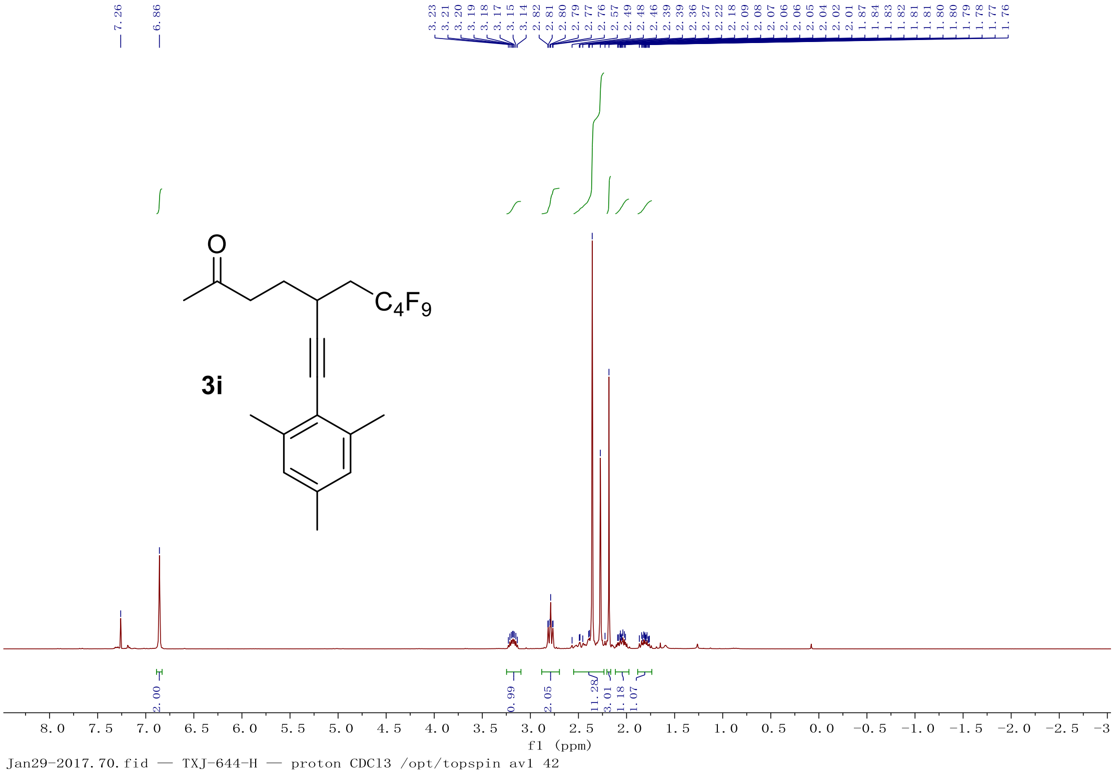
7.26 6.86 3.23 3.21 3.20 3.19 3.18 3.17 3.15 3.14 2.82 2.81 2.80 2.79 2.77 2.76 2.57 2.49 2.48 2.46 2.39 2.36 2.27 2.22 2.18 2.09 2.08 2.07 2.06 2.06 2.05 2.04 2.02 2.01 1.87 1.84 1.83 1.82 1.81 1.81 1.80 1.79 1.78 1.77 1.76
O
C4F9
3i
2.00 0.99 2.05 11.28 3.01 1.18 1.07
8.0 7.5 7.0 6.5 6.0 5.5 5.0 4.5 4.0 3.5 3.0 2.5 2.0 1.5 1.0 0.5 0.0 -0.5 -1.0 -1.5 -2.0 -2.5 -3
f1 (ppm)
Jan29-2017.70.fid — TXJ-644-H — proton CDCl3 /opt/topspin av1 42

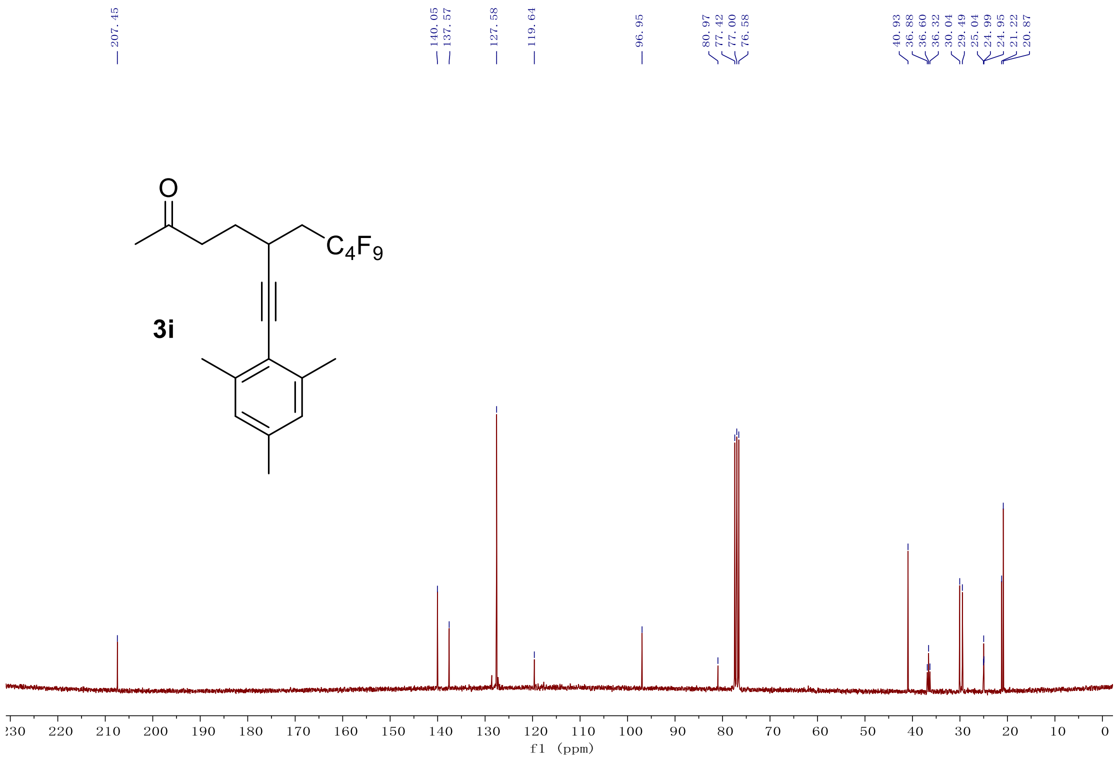

Jan29-2017.71.fid — TXJ-644-C — carbon CDCl3 /opt/topspin av1 42

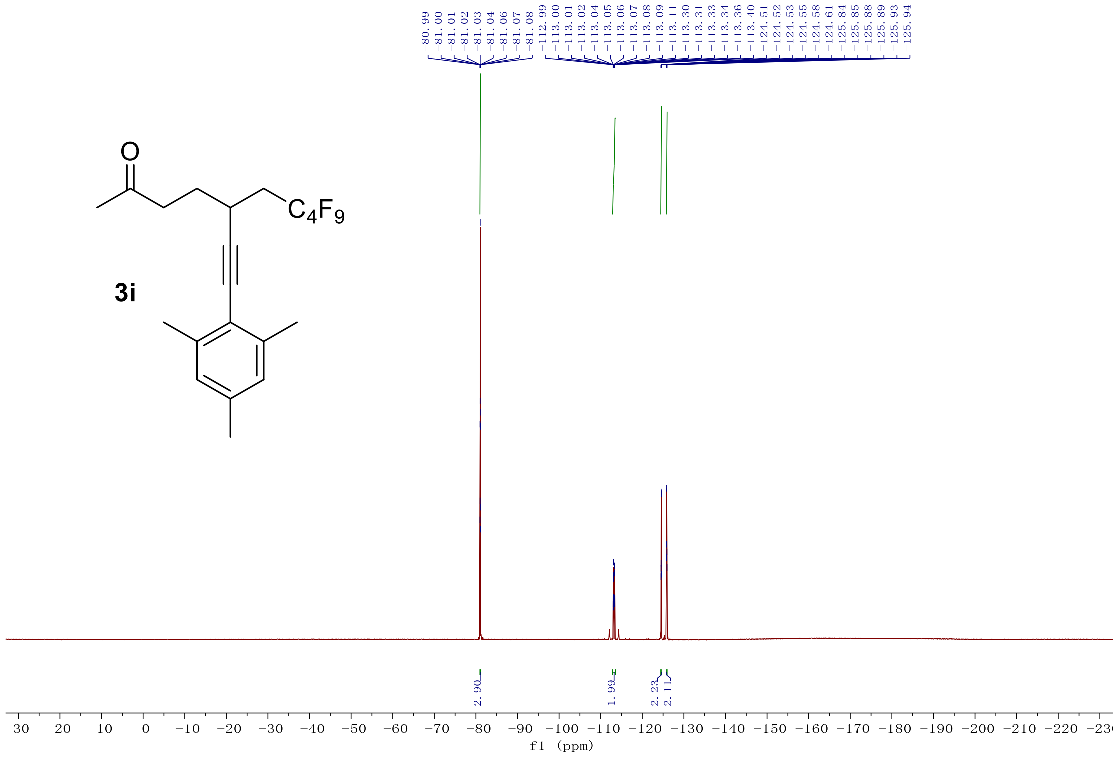

Jan29-2017.75.fid — TXJ-644-F — f19cpd CDCl3 /opt/topspin av1 42

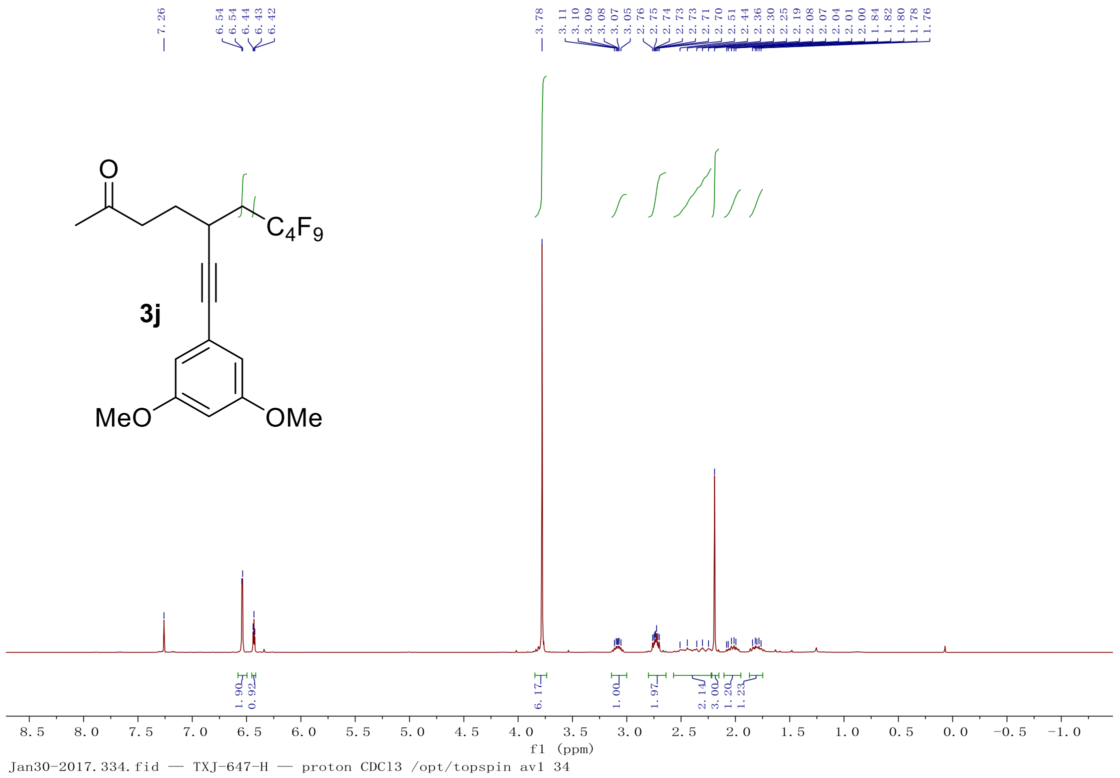
7.26
6.54 6.54 6.44 6.43 6.42
3.78
3.11 3.10 3.09 3.08 3.07 3.05 2.76 2.75 2.74 2.73 2.73 2.71 2.70 2.51 2.44 2.36 2.30 2.25 2.19 2.08 2.07 2.04 2.01 2.00 1.84 1.82 1.80 1.78 1.76
O
C4F9
3j
MeO
OMe
1.90 0.92 6.17 1.00 1.97 2.14 3.00 1.20 1.23
8.5 8.0 7.5 7.0 6.5 6.0 5.5 5.0 4.5 4.0 3.5 3.0 2.5 2.0 1.5 1.0 0.5 0.0 -0.5 -1.0
f1 (ppm)
Jan30-2017.334.fid — TXJ-647-H — proton CDCl3 /opt/topspin av1 34

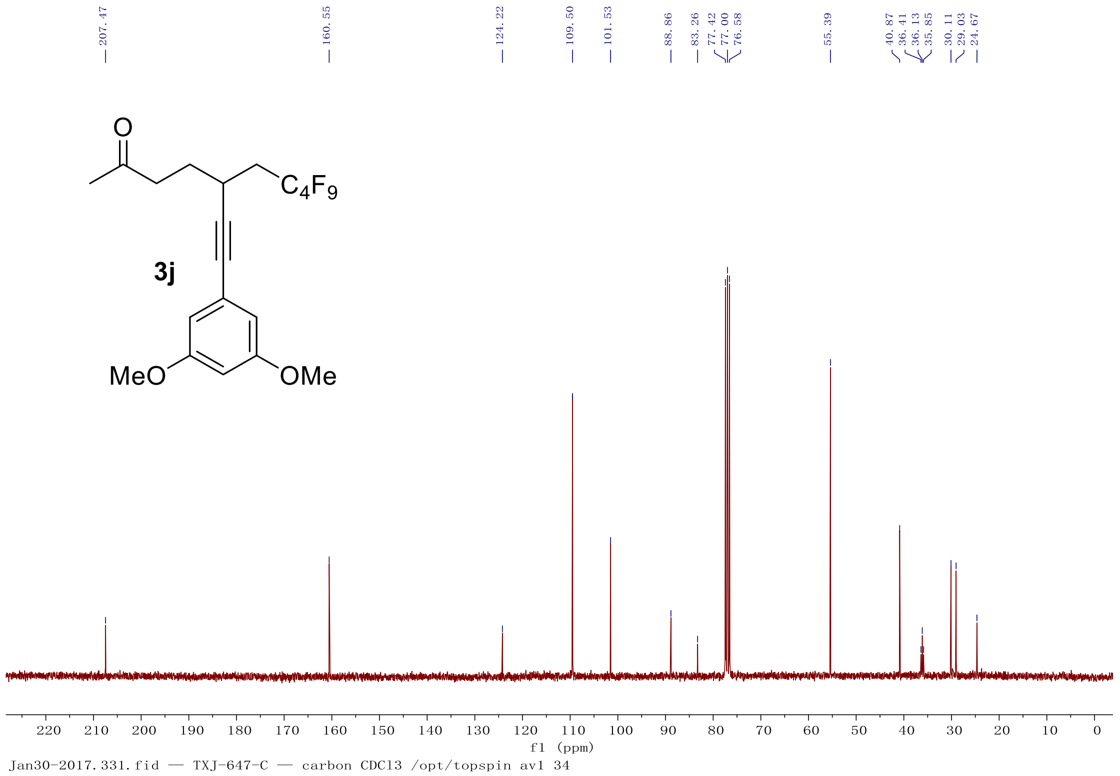
207.47
160.55
124.22
109.50
101.53
88.86
83.26
77.42 77.00 76.58
55.39
40.87 36.41 36.13 35.85
30.11 29.03 24.67
O
C4F9
3j
MeO
OMe
220 210 200 190 180 170 160 150 140 130 120 110 100 90 80 70 60 50 40 30 20 10 0
f1 (ppm)
Jan30-2017.331.fid — TXJ-647-C — carbon CDCl3 /opt/topspin av1 34

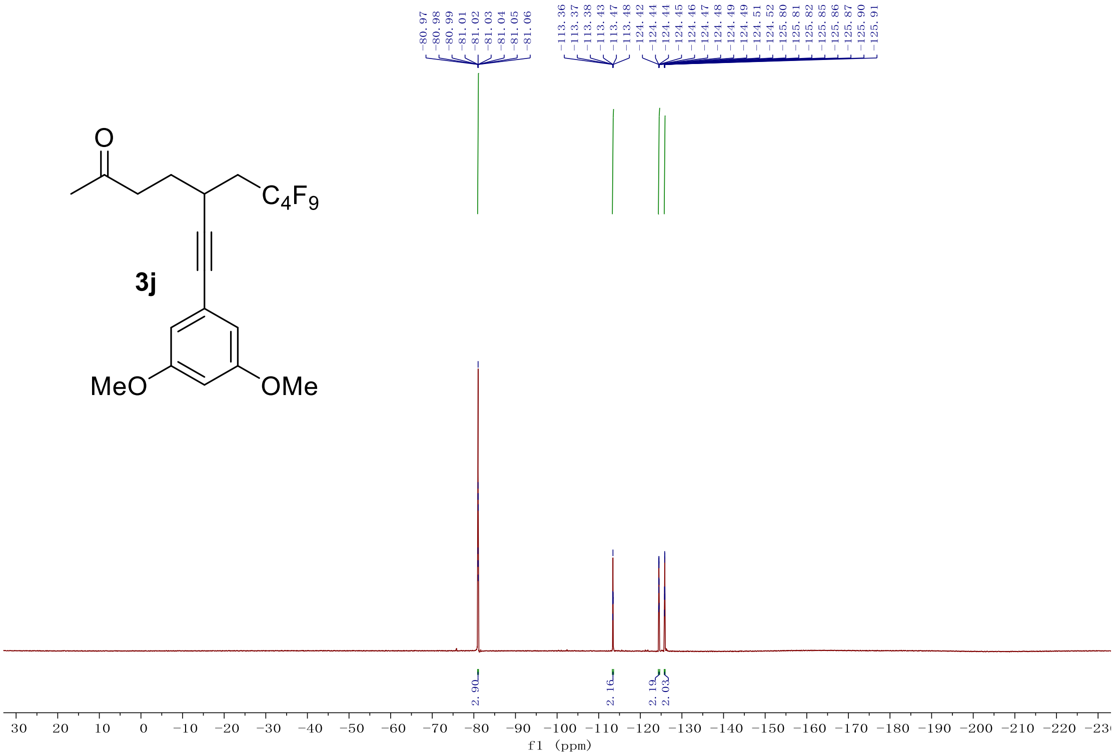

Jan30-2017.335.fid — TXJ-647-F — f19cpd CDCl3 /opt/topspin av1 34

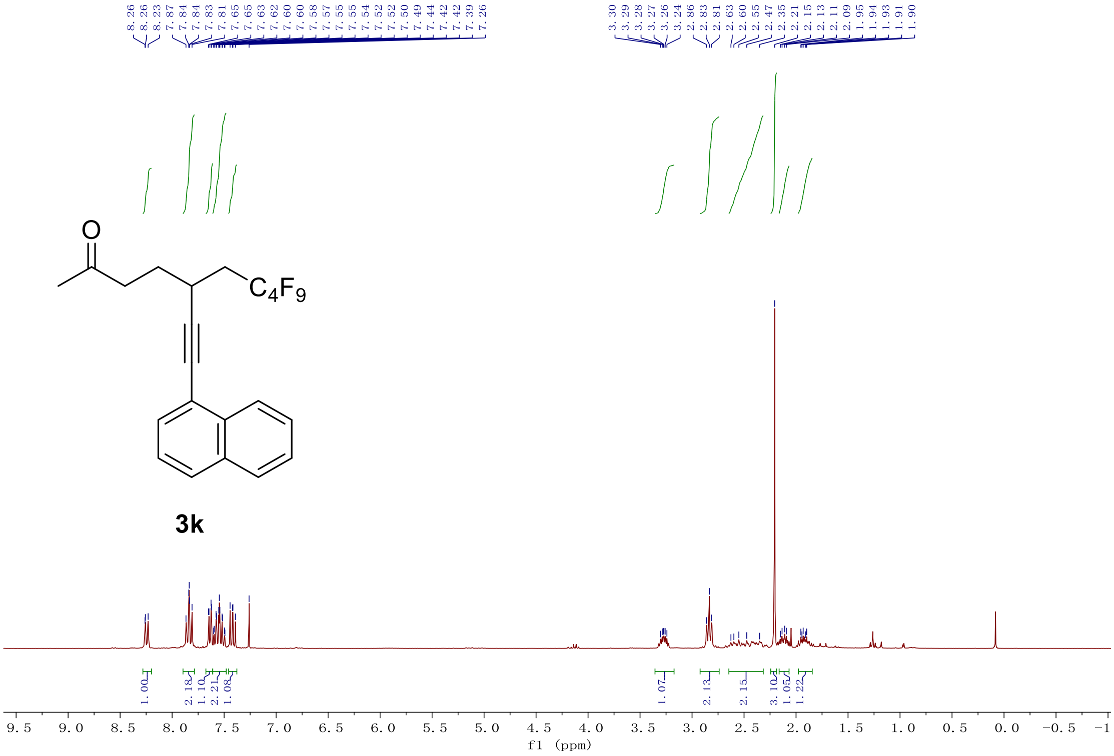

Jan30-2017.344.fid — TXJ-648-H — proton CDCl3 /opt/topspin av1 35

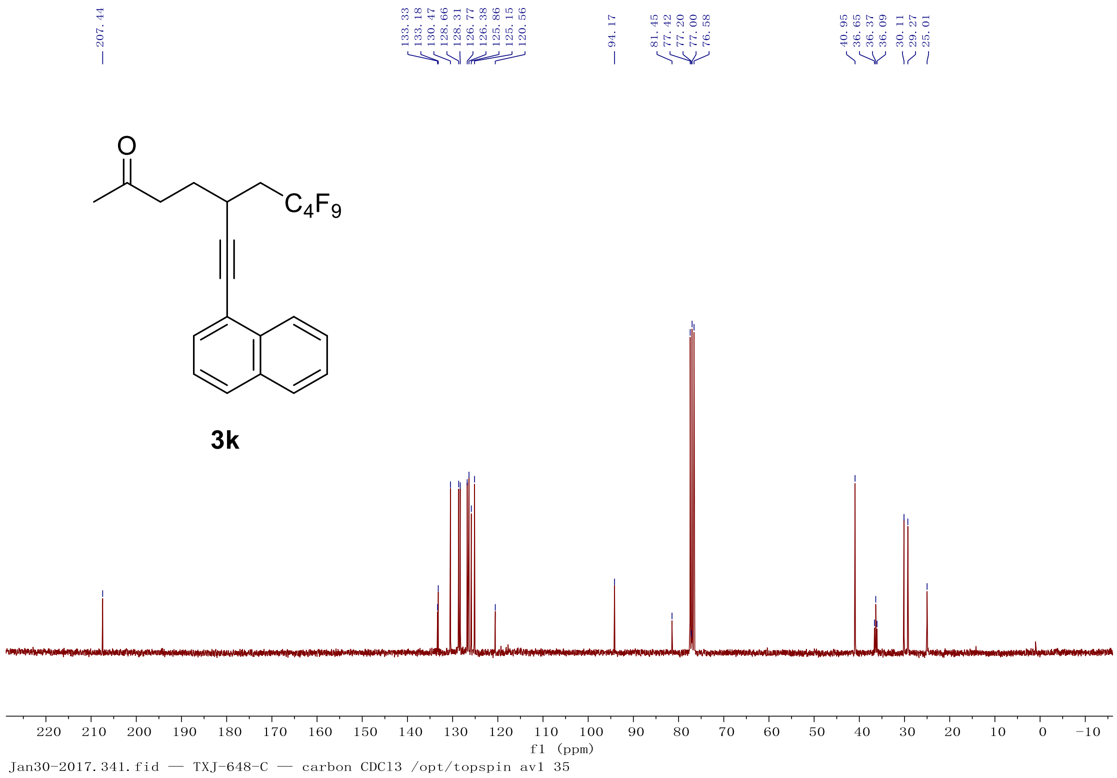
207.44
133.33
133.18
130.47
128.66
128.31
126.77
126.38
125.86
125.15
120.56
94.17
81.45
77.42
77.20
77.00
76.58
40.95
36.65
36.37
36.09
30.11
29.27
25.01
O
C_4F_9
3k
f1 (ppm)
Jan30-2017.341.fid — TXJ-648-C — carbon CDCl3 /opt/topspin av1 35

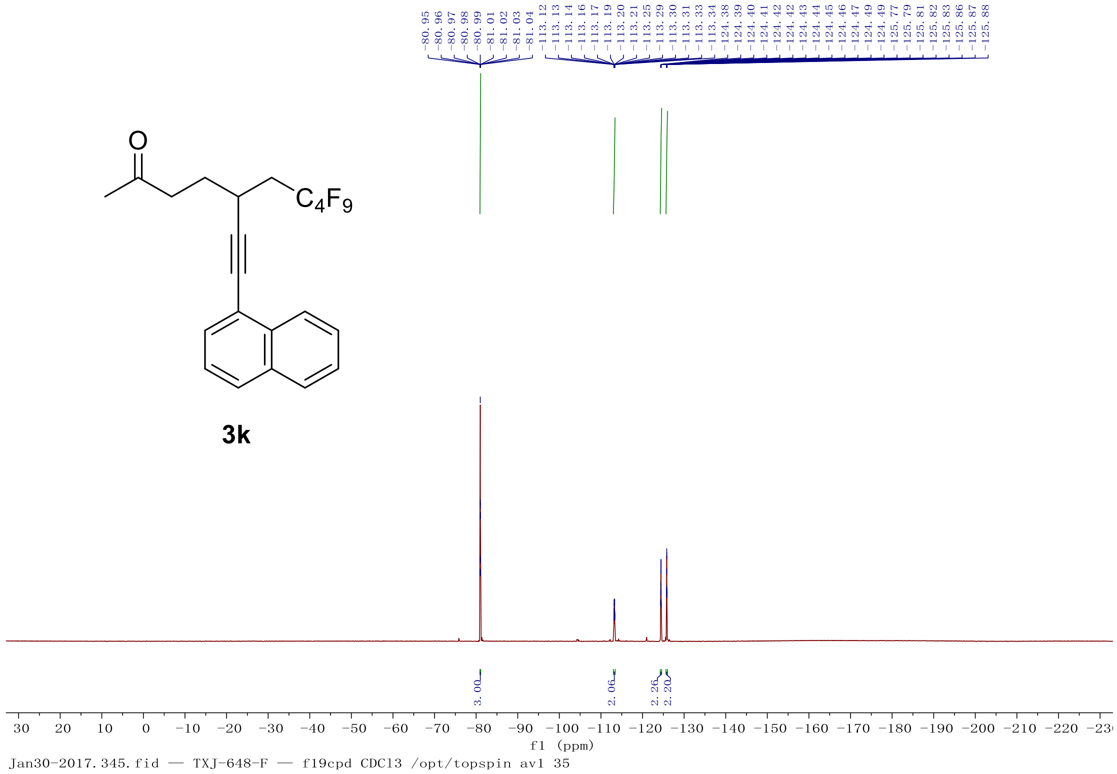
O
C_4F_9
3k
3.00
2.06
2.26
2.20
f1 (ppm)
Jan30-2017.345.fid — TXJ-648-F — f19cpd CDCl3 /opt/topspin av1 35

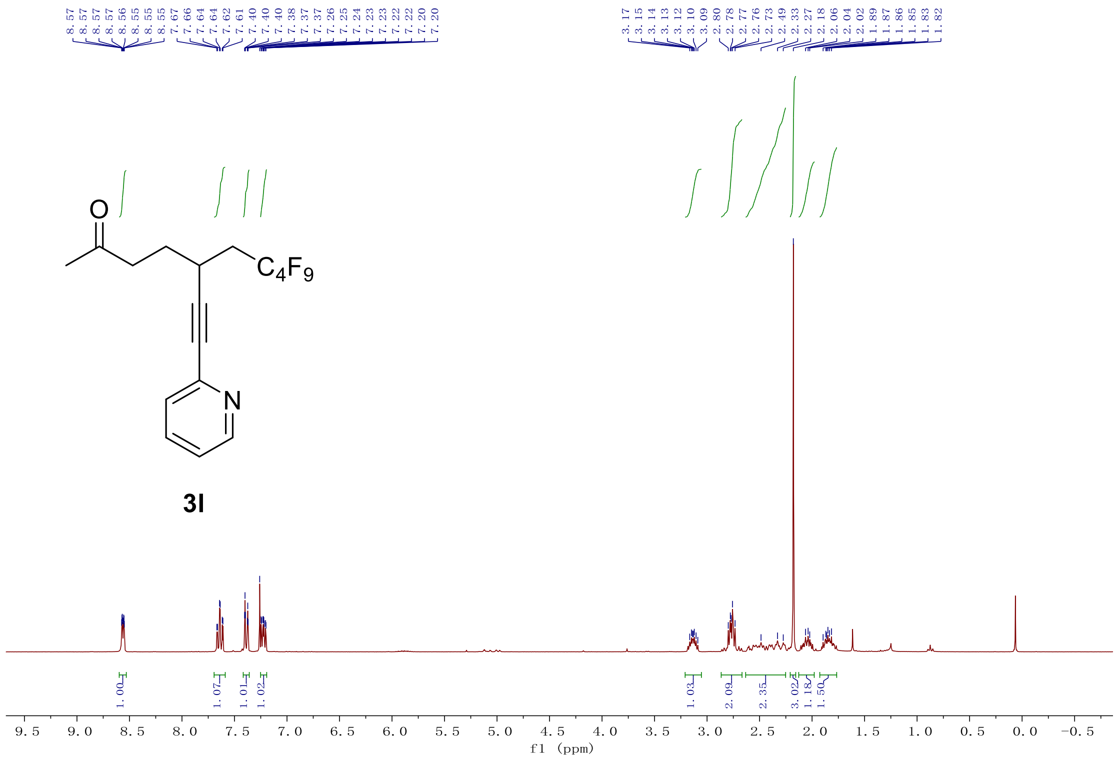

Feb02-2017.294.fid — TXJ-652-H — proton CDCl3 /opt/topspin av1 31

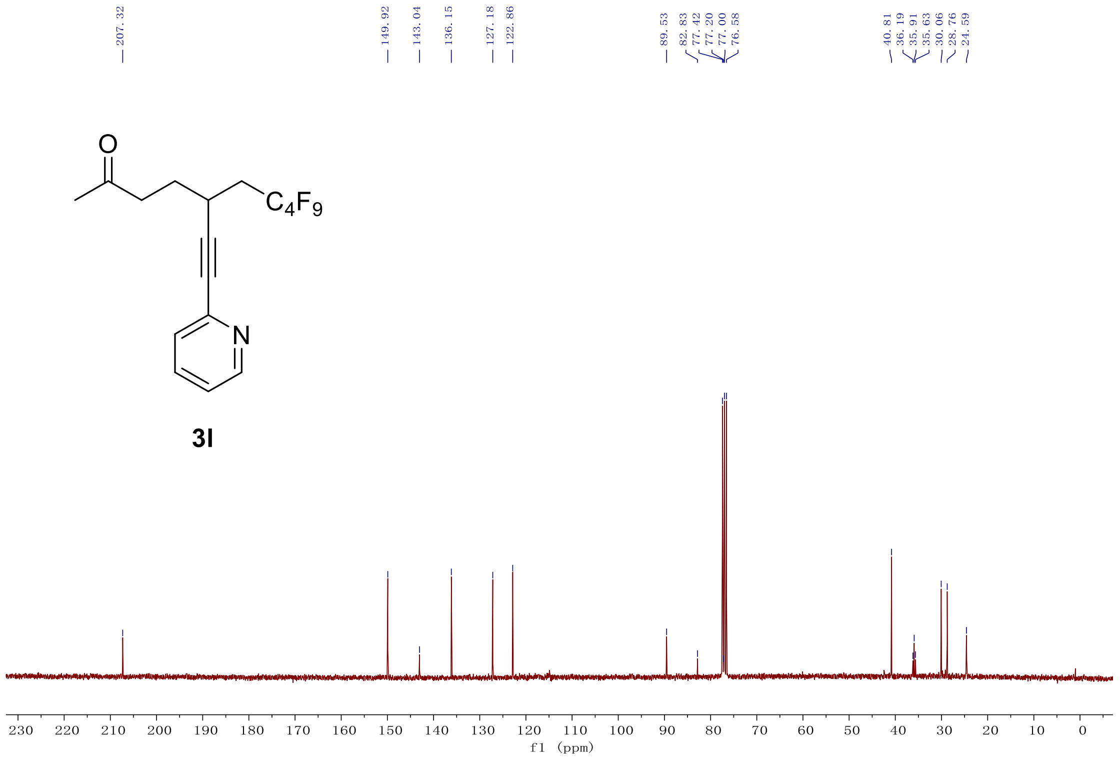

Feb02-2017.291.fid — TXJ-652-C — carbon CDCl3 /opt/topspin av1 31

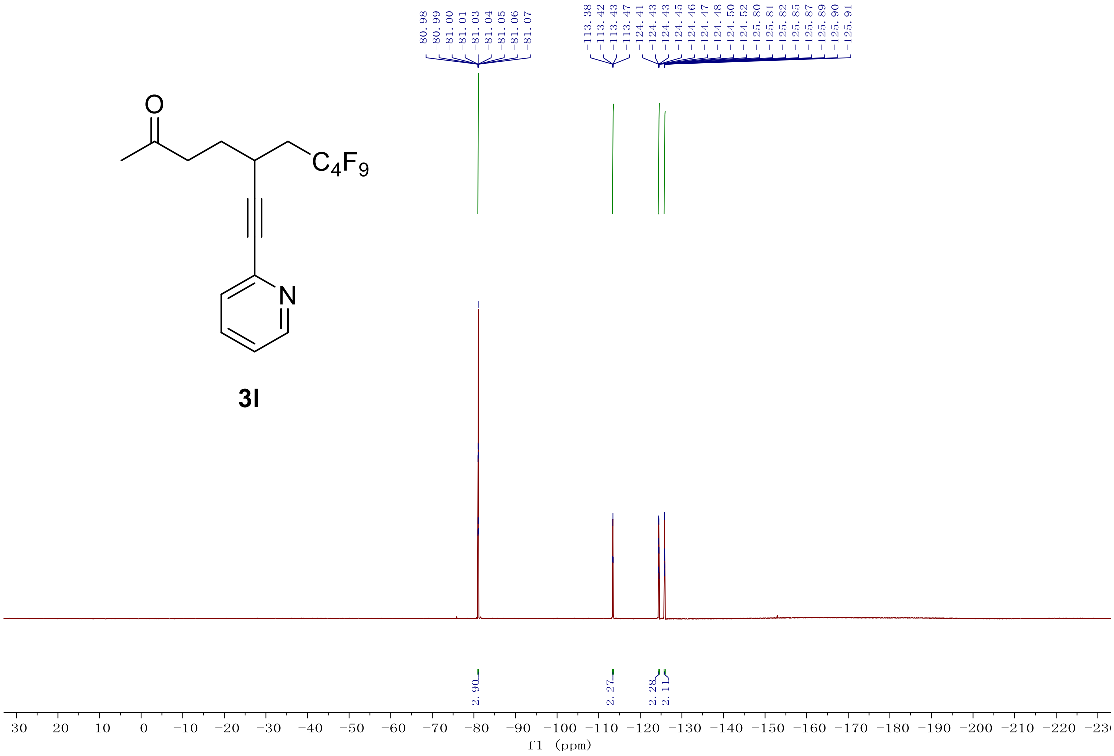

3l

Feb02-2017.295.fid — TXJ-652-F — f19cpd CDCl3 /opt/topspin av1 31

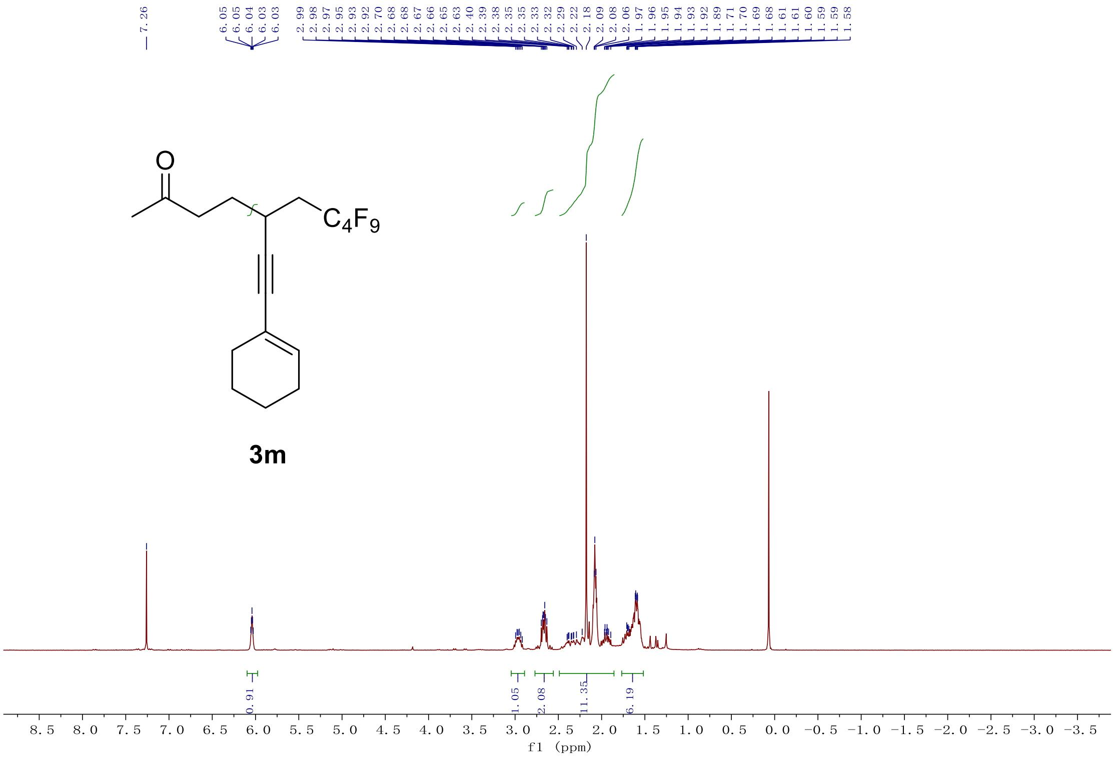

3m

Feb01-2017.294.fid — TXJ-649-H — proton CDCl3 /opt/topspin av1 31

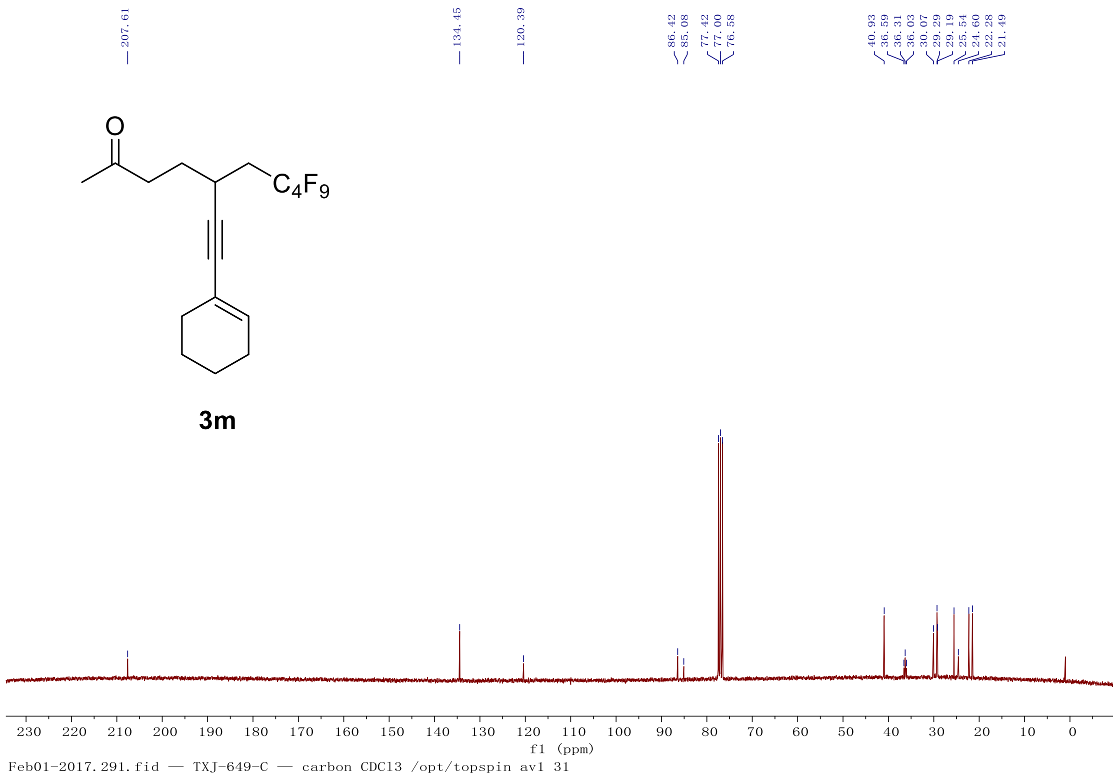

207.61
134.45
120.39
86.42
85.08
77.42
77.00
76.58
40.93
36.59
36.31
36.03
30.07
29.29
29.19
25.54
24.60
22.28
21.49
f1 (ppm)
Feb01-2017.291.fid — TXJ-649-C — carbon CDCl3 /opt/topspin av1 31

3m

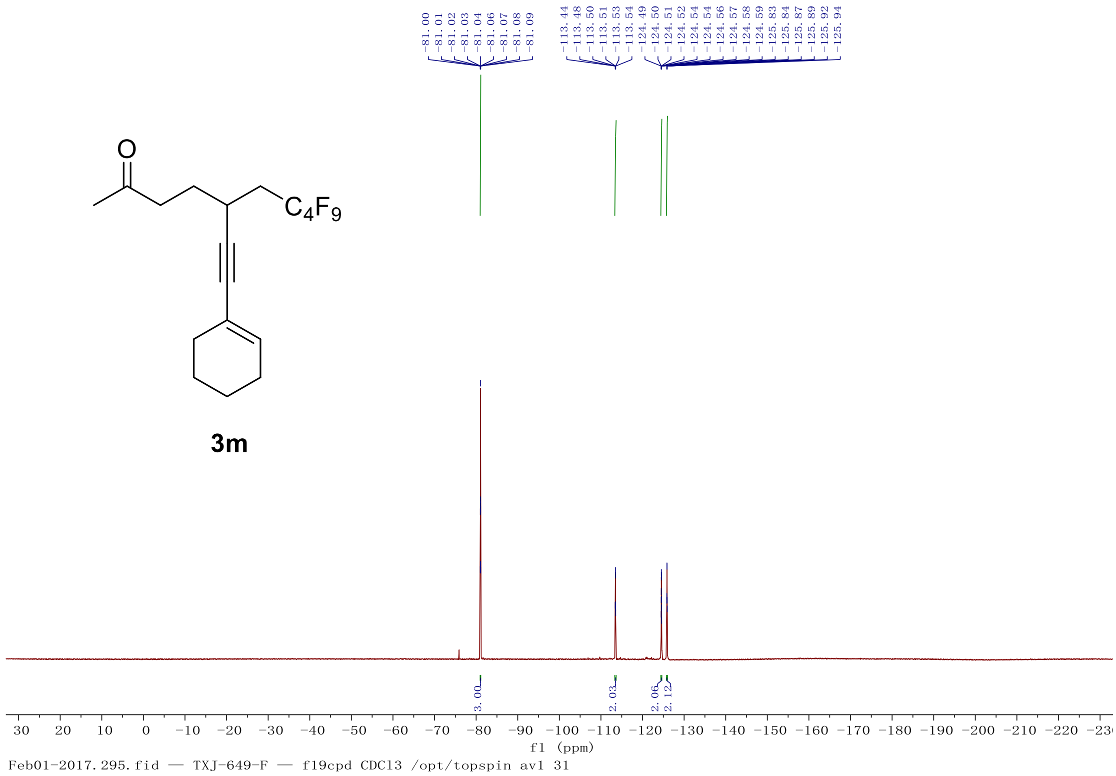

-81.00
-81.01
-81.02
-81.03
-81.04
-81.06
-81.07
-81.08
-81.09
-113.44
-113.48
-113.50
-113.51
-113.53
-113.54
-124.49
-124.50
-124.51
-124.52
-124.54
-124.54
-124.56
-124.57
-124.58
-124.59
-125.83
-125.84
-125.87
-125.89
-125.92
-125.94
3.00
2.03
2.06
2.12
f1 (ppm)
Feb01-2017.295.fid — TXJ-649-F — f19cpd CDCl3 /opt/topspin av1 31

3m

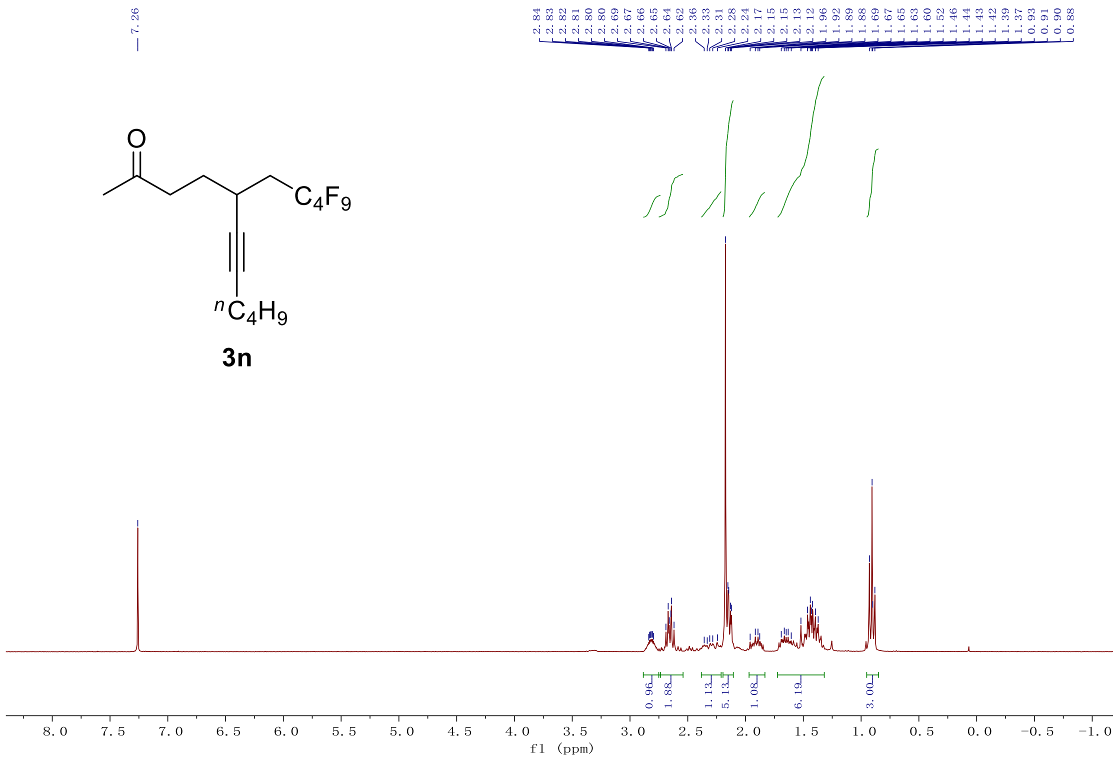

Feb01-2017.304.fid — TXJ-650-H — proton CDCl3 /opt/topspin av1 32

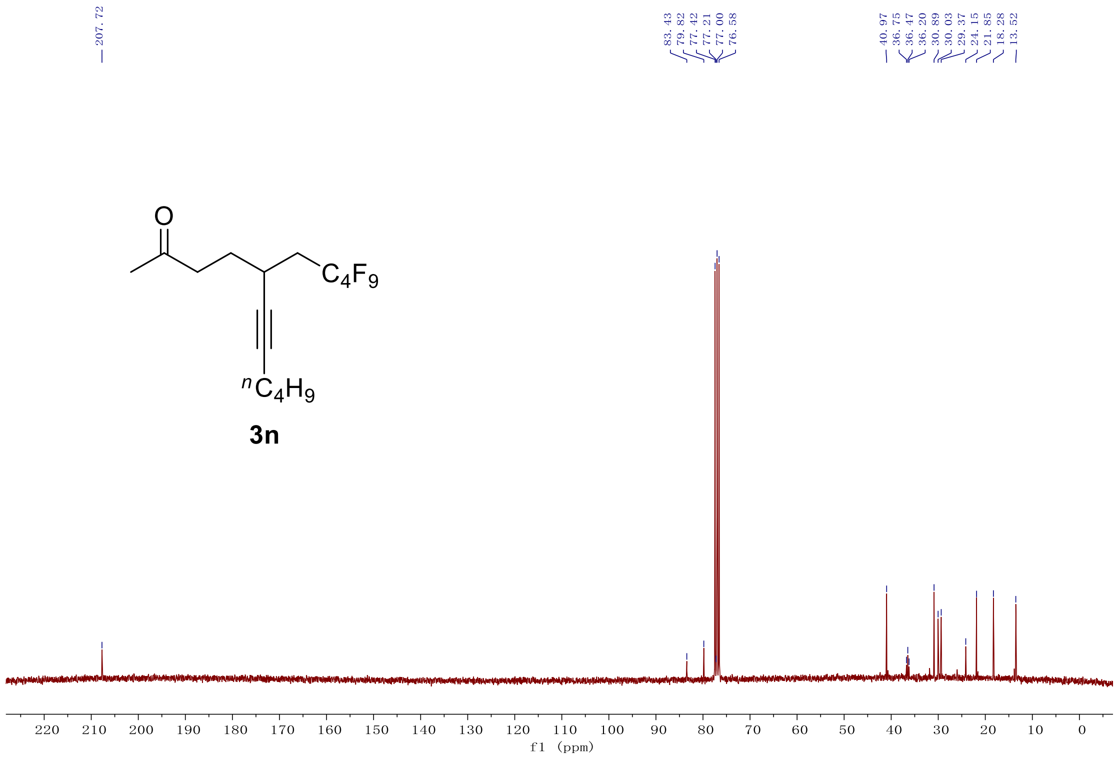

Feb01-2017.301.fid — TXJ-650-C — carbon CDCl3 /opt/topspin av1 32

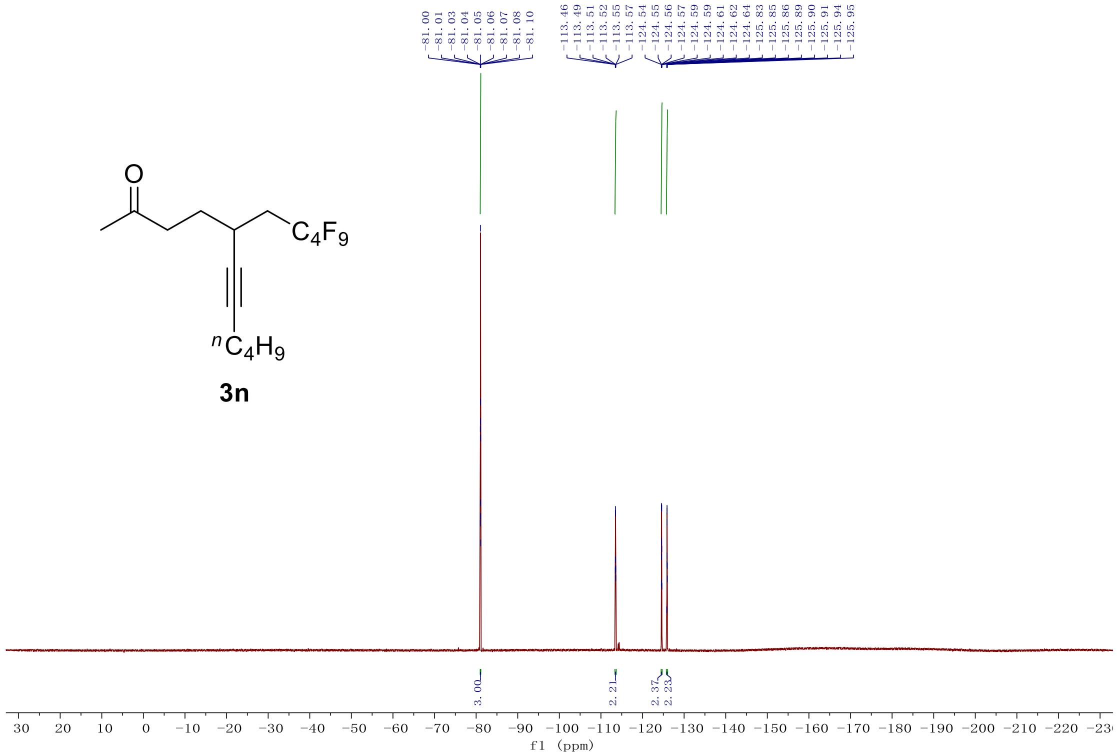

3n

Feb01-2017.305.fid — TXJ-650-F — f19cpd CDCl3 /opt/topspin av1 32

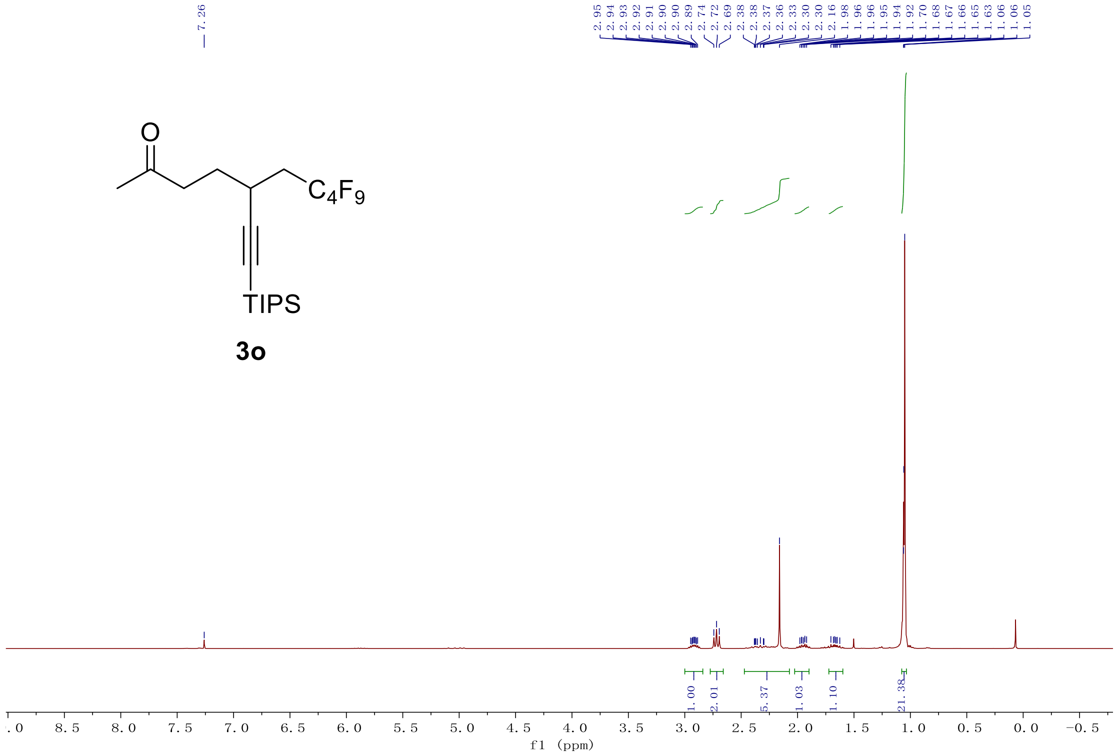

3o

Mar13-2017.500.fid — TXJ-719-H — proton CDCl3 /opt/topspin av1 34

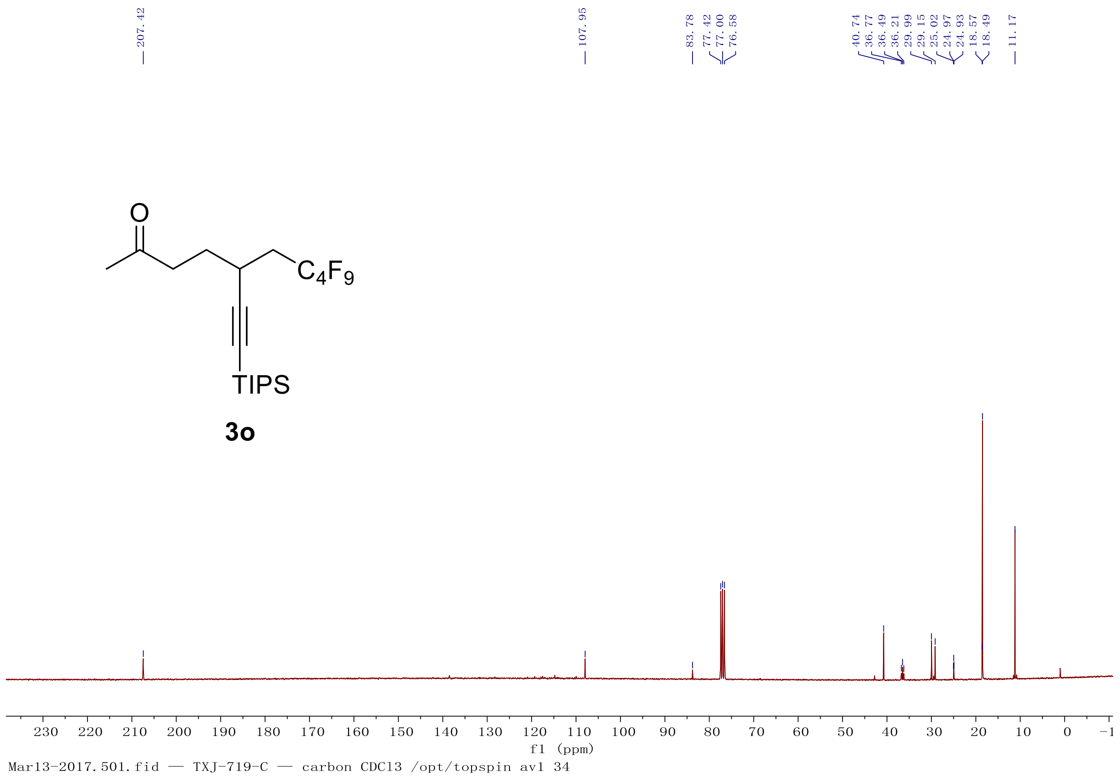
207.42
107.95
83.78
77.42
77.00
76.58
40.74
36.77
36.49
36.21
29.99
29.15
25.02
24.97
24.93
18.57
18.49
11.17
O
C4F9
TIPS
3o
f1 (ppm)
Mar13-2017.501.fid — TXJ-719-C — carbon CDCl3 /opt/topspin av1 34

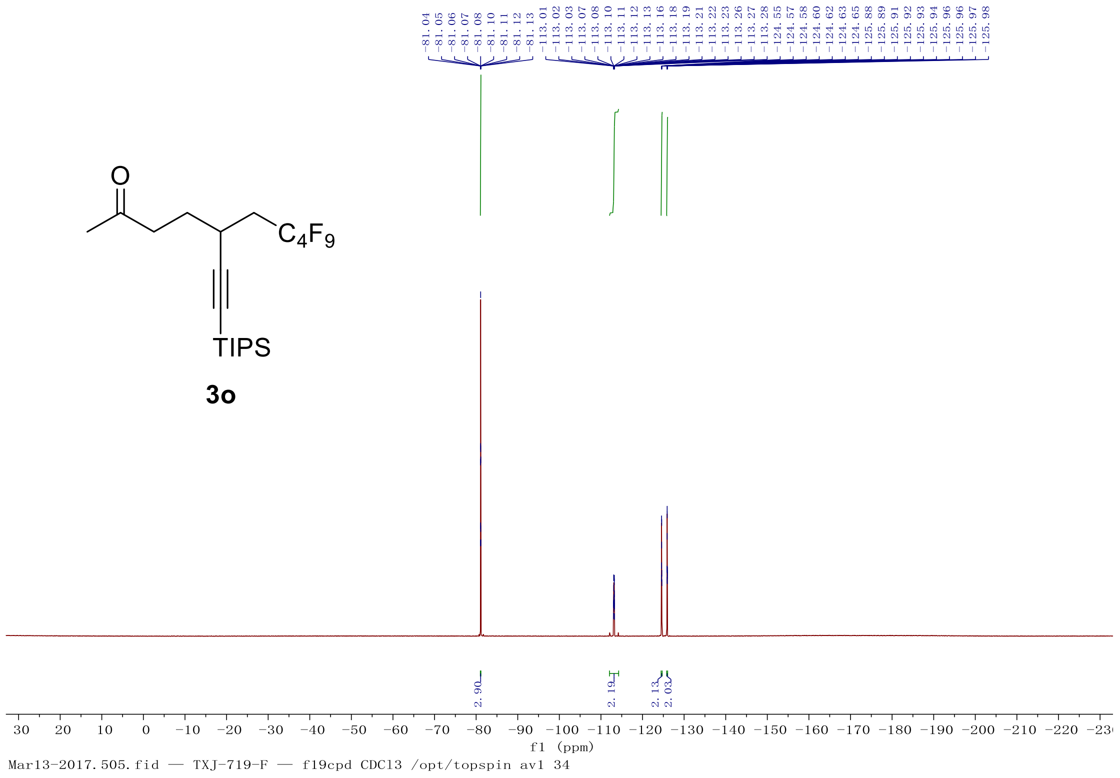
-81.04
-81.05
-81.06
-81.07
-81.08
-81.10
-81.11
-81.12
-81.13
-113.01
-113.02
-113.03
-113.07
-113.08
-113.10
-113.11
-113.12
-113.13
-113.16
-113.18
-113.19
-113.21
-113.22
-113.23
-113.26
-113.27
-113.28
-124.55
-124.57
-124.58
-124.60
-124.62
-124.63
-124.65
-125.88
-125.89
-125.91
-125.92
-125.93
-125.94
-125.96
-125.96
-125.97
-125.98
O
C4F9
TIPS
3o
2.90
2.19
2.13
2.03
f1 (ppm)
Mar13-2017.505.fid — TXJ-719-F — f19cpd CDCl3 /opt/topspin av1 34

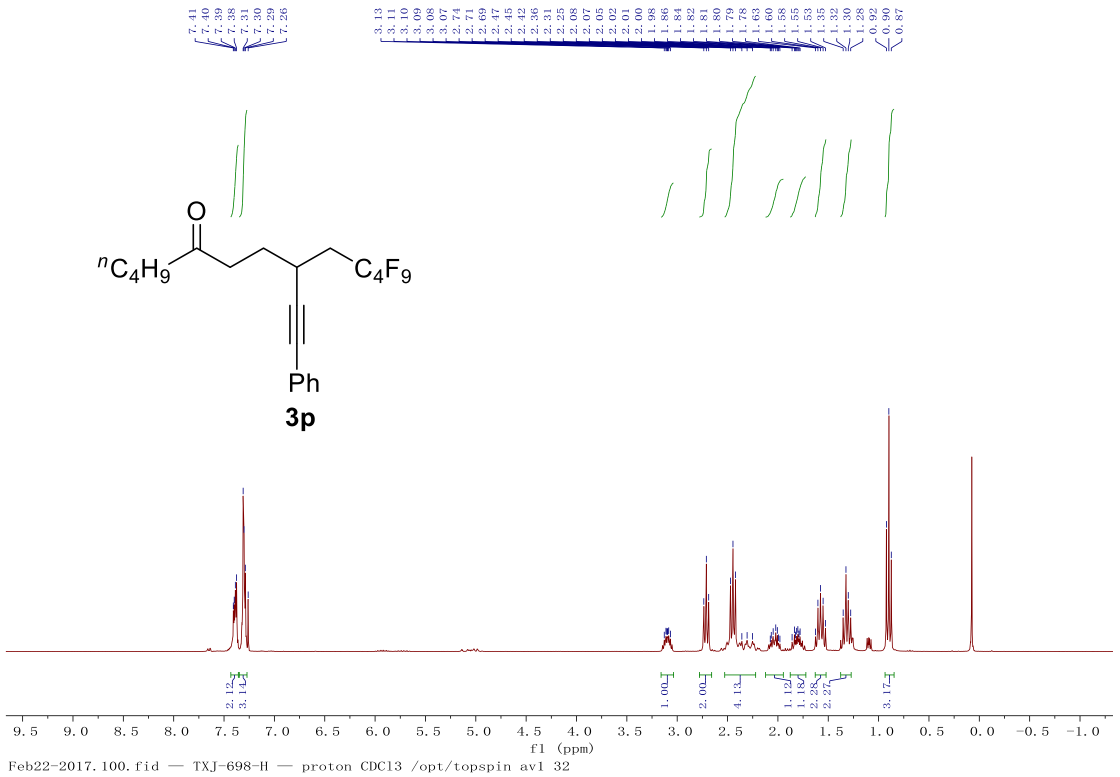

7.41 7.40 7.39 7.38 7.31 7.30 7.29 7.26
3.13 3.11 3.10 3.09 3.08 3.07 2.74 2.71 2.69 2.47 2.45 2.42 2.36 2.31 2.25 2.08 2.07 2.05 2.02 2.01 2.00 1.98 1.86 1.84 1.82 1.81 1.80 1.79 1.78 1.63 1.60 1.58 1.55 1.53 1.35 1.32 1.30 1.28 0.92 0.90 0.87
O
$^nC_4H_9$
C_4F_9
Ph
3p
2.12 3.14 1.00 2.00 4.13 1.12 1.18 2.28 2.27 3.17
f1 (ppm)
Feb22-2017.100.fid — TXJ-698-H — proton CDCl3 /opt/topspin av1 32

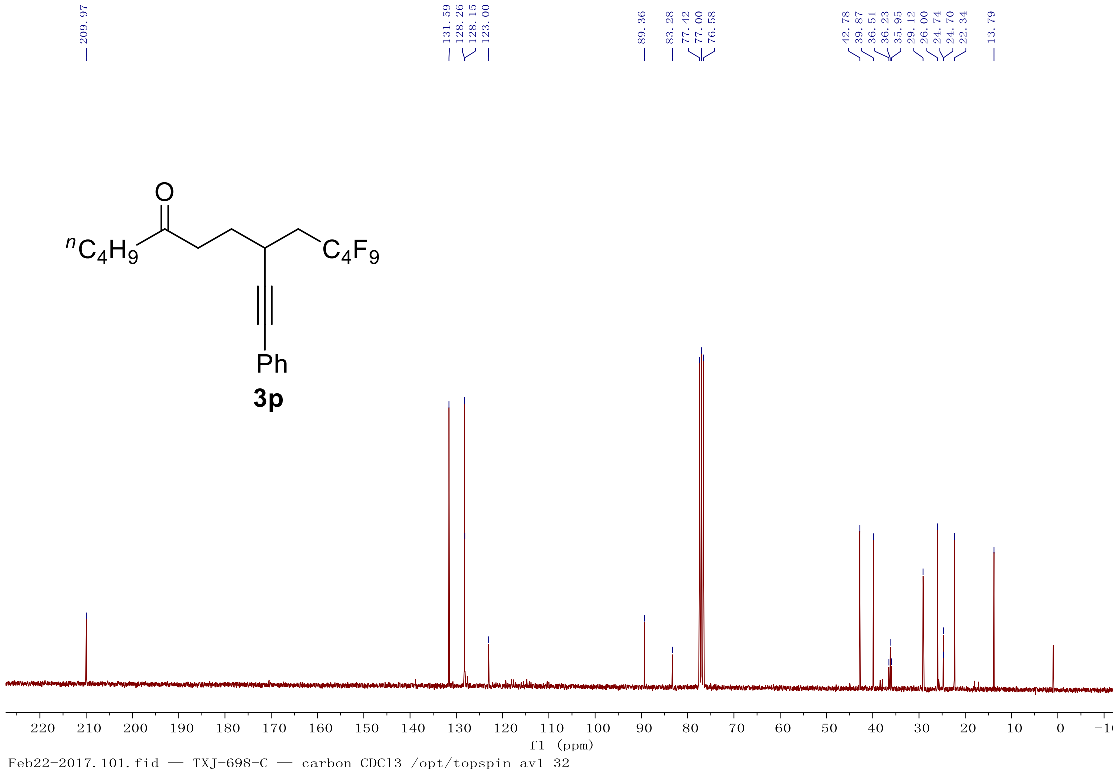

209.97
131.59 128.26 128.15 123.00
89.36
83.28 77.42 77.00 76.58
42.78 39.87 36.51 36.23 35.95 29.12 26.00 24.74 24.70 22.34
13.79
O
$^nC_4H_9$
C_4F_9
Ph
3p
f1 (ppm)
Feb22-2017.101.fid — TXJ-698-C — carbon CDCl3 /opt/topspin av1 32

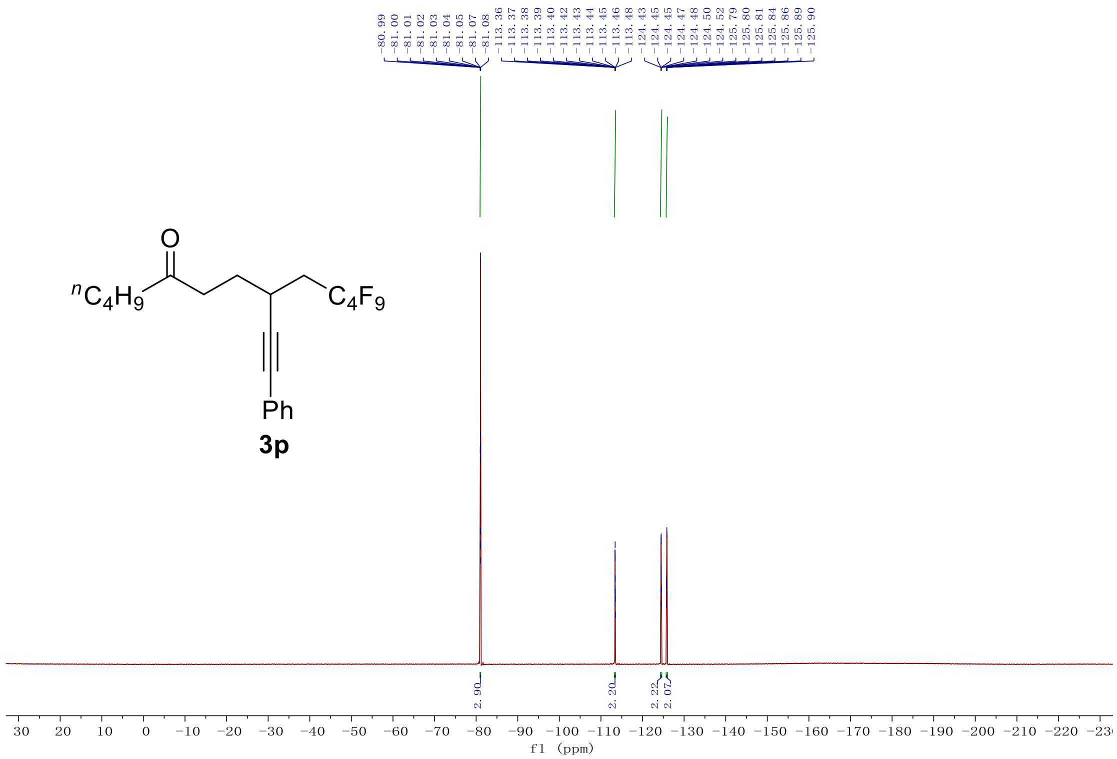

Feb22-2017.105.fid — TXJ-698-F — f19cpd CDCl3 /opt/topspin av1 32

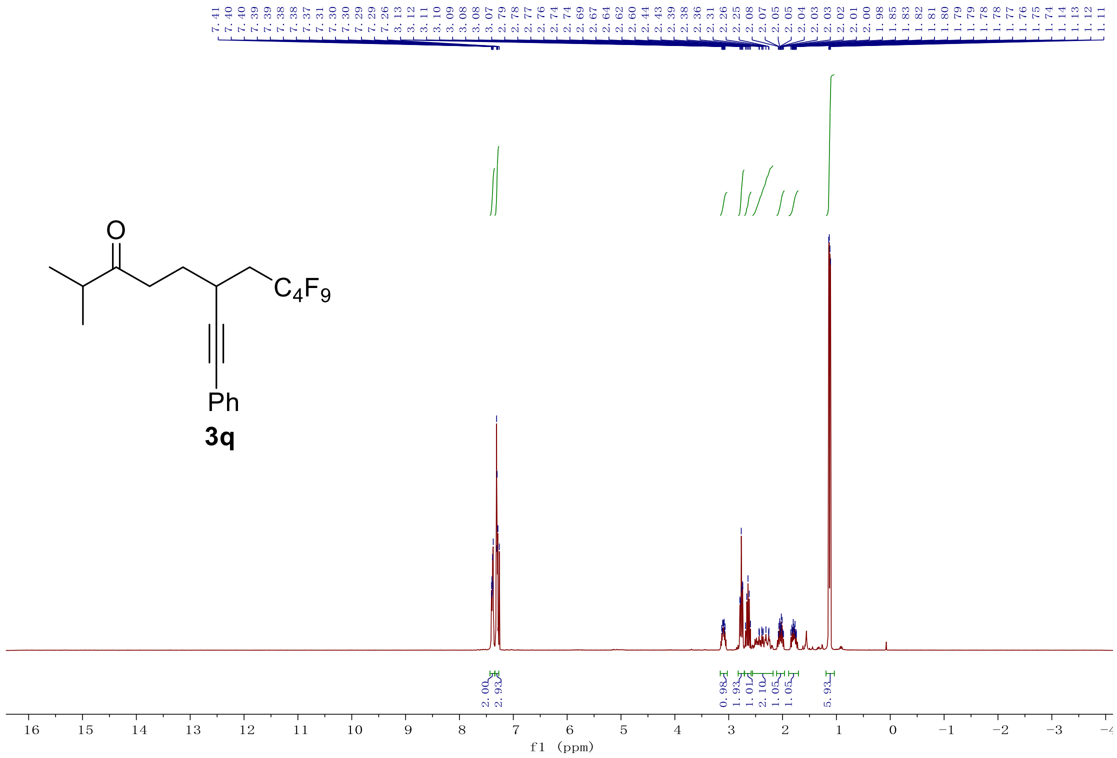

Feb23-2017.114.fid — TXJ-699-H — proton CDCl3 /opt/topspin av1 31

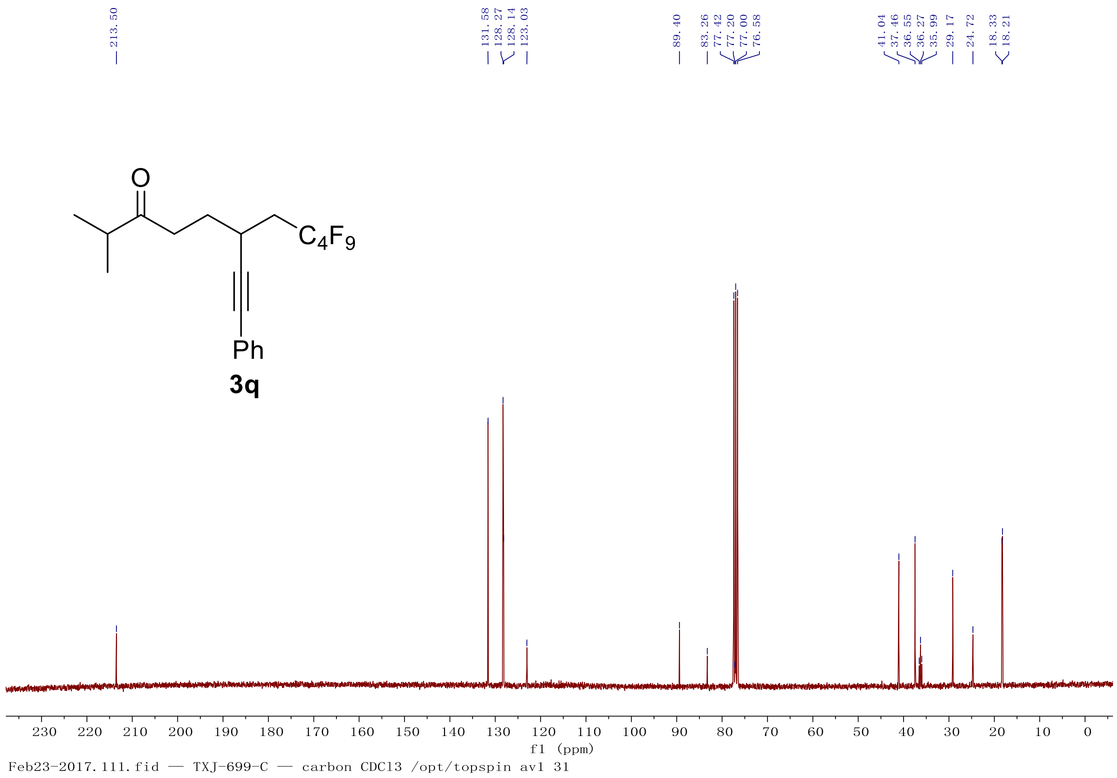
213.50
131.58
128.27
128.14
123.03
89.40
83.26
77.42
77.20
77.00
76.58
41.04
37.46
36.55
36.27
35.99
29.17
24.72
18.33
18.21
O
C4F9
Ph
3q
230 220 210 200 190 180 170 160 150 140 130 120 110 100 90 80 70 60 50 40 30 20 10 0
f1 (ppm)
Feb23-2017.111.fid — TXJ-699-C — carbon CDCl3 /opt/topspin av1 31

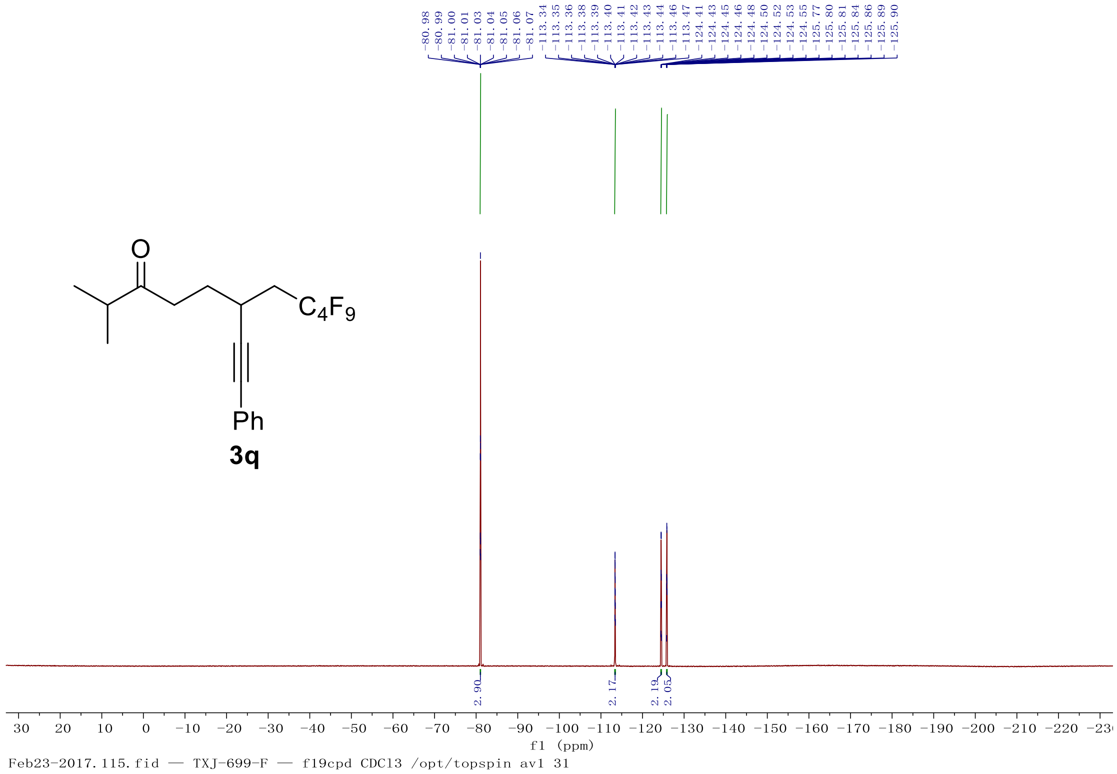
-80.98
-80.99
-81.00
-81.01
-81.03
-81.04
-81.05
-81.06
-81.07
-113.34
-113.35
-113.36
-113.38
-113.39
-113.40
-113.41
-113.42
-113.43
-113.44
-113.46
-113.47
-124.41
-124.43
-124.45
-124.46
-124.48
-124.50
-124.52
-124.53
-124.55
-125.77
-125.80
-125.81
-125.84
-125.86
-125.89
-125.90
O
C4F9
Ph
3q
2.90
2.17
2.19
2.05
30 20 10 0 -10 -20 -30 -40 -50 -60 -70 -80 -90 -100 -110 -120 -130 -140 -150 -160 -170 -180 -190 -200 -210 -220
f1 (ppm)
Feb23-2017.115.fid — TXJ-699-F — f19cpd CDCl3 /opt/topspin av1 31

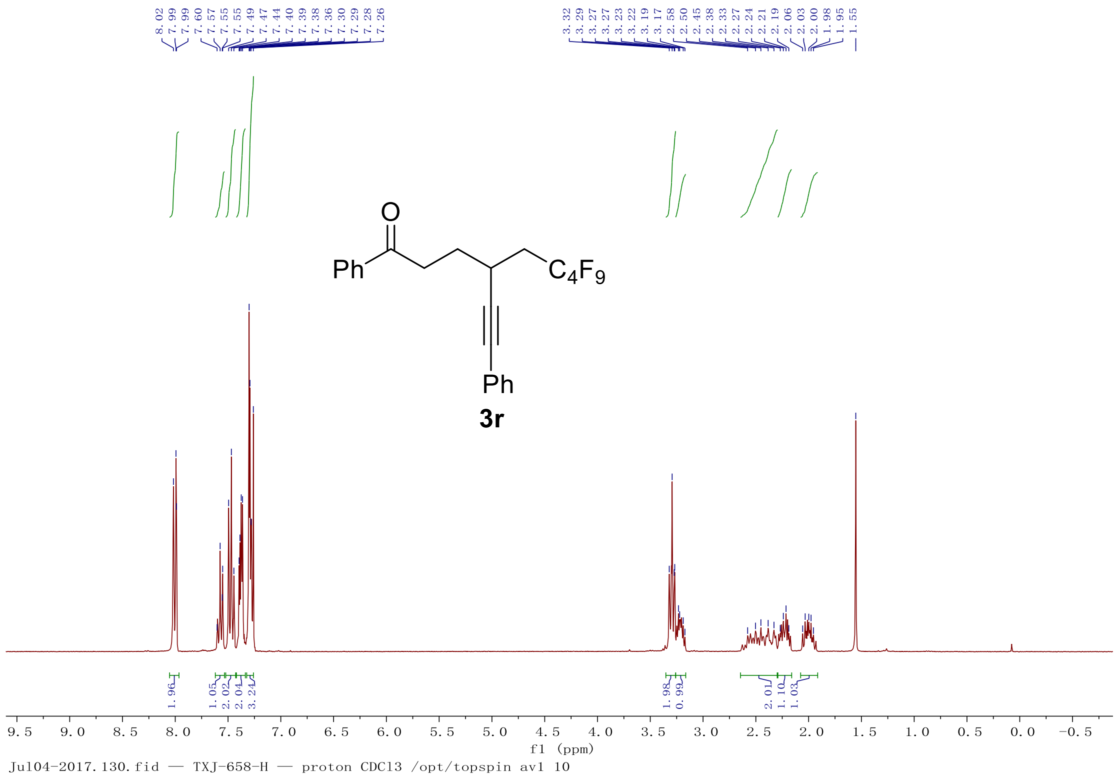
8.02 7.99 7.99 7.60 7.57 7.55 7.55 7.49 7.47 7.44 7.40 7.39 7.38 7.36 7.30 7.29 7.28 7.26
3.32 3.29 3.27 3.27 3.23 3.22 3.19 3.17 2.58 2.50 2.45 2.38 2.33 2.27 2.24 2.21 2.19 2.06 2.03 2.00 1.98 1.95 1.55
O
Ph
C4F9
Ph
3r
1.96 1.05 2.02 2.04 3.24 1.98 0.99 2.01 1.10 1.03
9.5 9.0 8.5 8.0 7.5 7.0 6.5 6.0 5.5 5.0 4.5 4.0 3.5 3.0 2.5 2.0 1.5 1.0 0.5 0.0 -0.5
f1 (ppm)
Jul04-2017.130.fid — TXJ-658-H — proton CDCl3 /opt/topspin av1 10

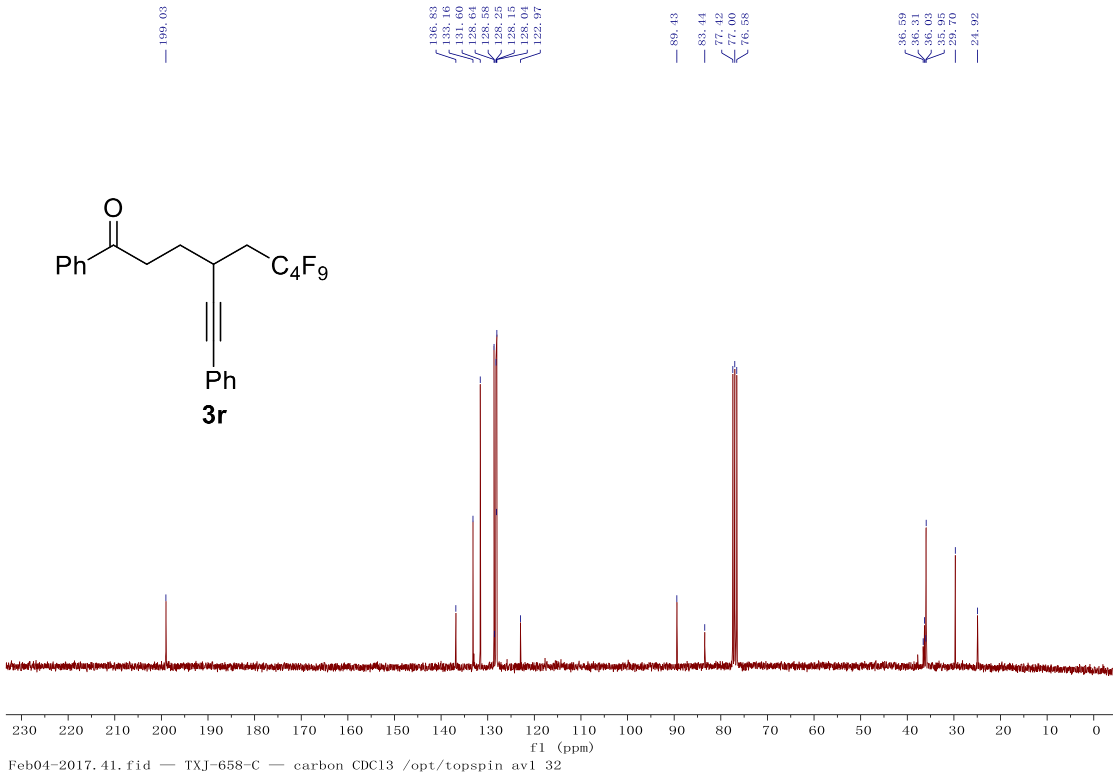
199.03
136.83 133.16 131.60 128.64 128.58 128.25 128.15 128.04 122.97
89.43 83.44 77.42 77.00 76.58
36.59 36.31 36.03 35.95 29.70 24.92
O
Ph
C4F9
Ph
3r
230 220 210 200 190 180 170 160 150 140 130 120 110 100 90 80 70 60 50 40 30 20 10 0
f1 (ppm)
Feb04-2017.41.fid — TXJ-658-C — carbon CDCl3 /opt/topspin av1 32

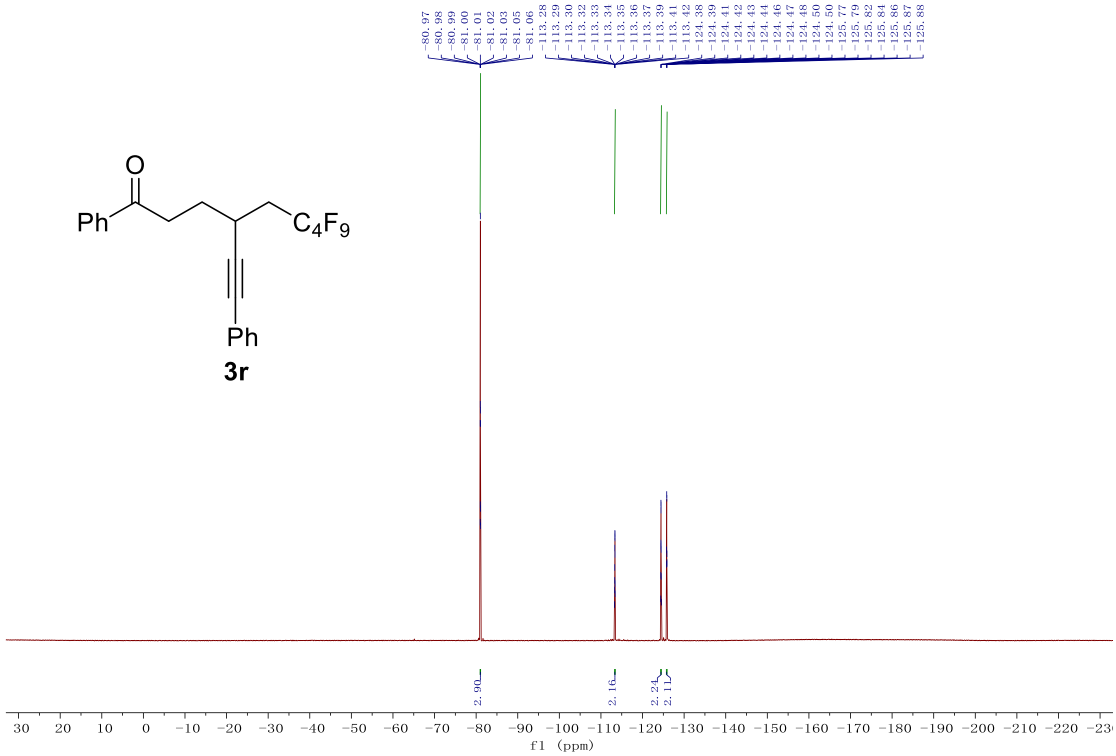

Feb04-2017.45.fid — TXJ-658-F — f19cpd CDCl3 /opt/topspin av1 32

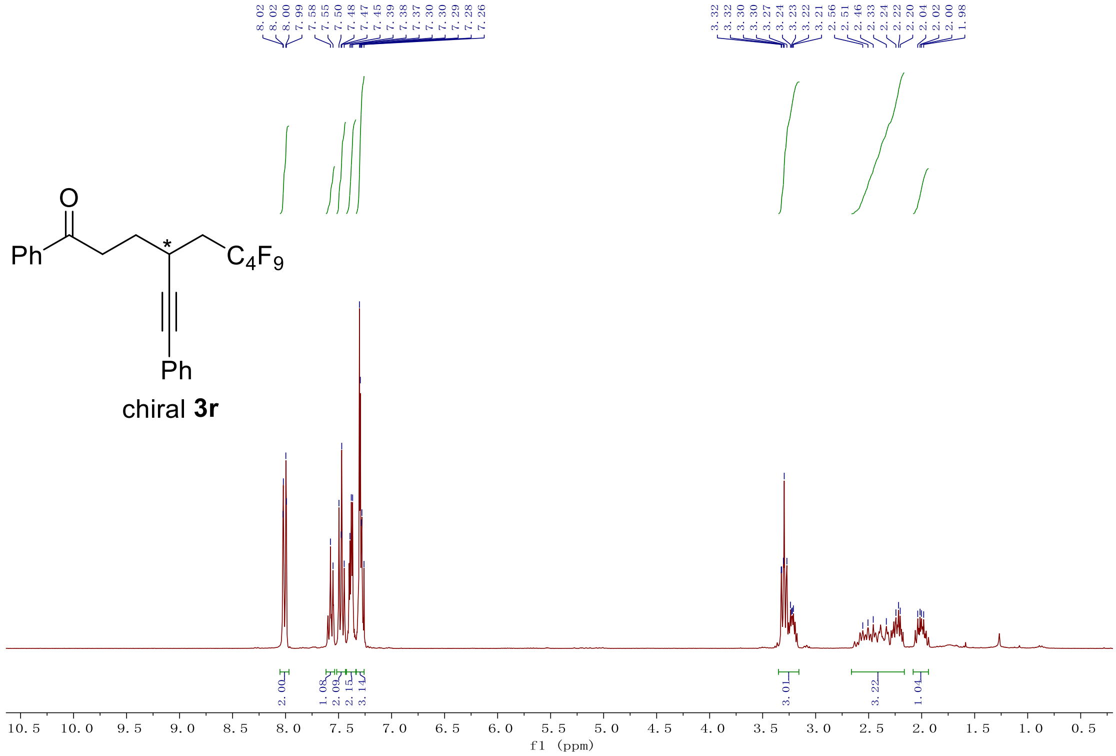

May10-2017.250.fid — TXJ-745-H — proton CDCl3 /opt/topspin av1 2

Data File C:\CHEM32\1\DATA\TANG\TXJ658000004.D
Sample Name: TXJ658

===

Acq. Operator : prekel
Acq. Instrument : Instrument 1 Location : Vial 1
Injection Date : 11.04.2017 15:31:48
Inj Volume : 5.0 µl
Acq. Method : C:\CHEM32\1\METHODS\RP CHIRAL\RP CHIRAL_65-35.M
Last changed : 11.04.2017 15:31:35 by prekel
(modified after loading)
Analysis Method : C:\CHEM32\1\METHODS\RP NEUTRAL 1ML\RP_65-35_1.M
Last changed : 24.06.2016 10:35:17 by prekel
Method Info : Standard Method RP_65:35_1
Flow: 1mL/min; 20°C; Injection 5µL; time: 50min
Solvent System:MeCN/H2O=65/35
isocratic

Sample Info : OJ-RH
ACN/H2O

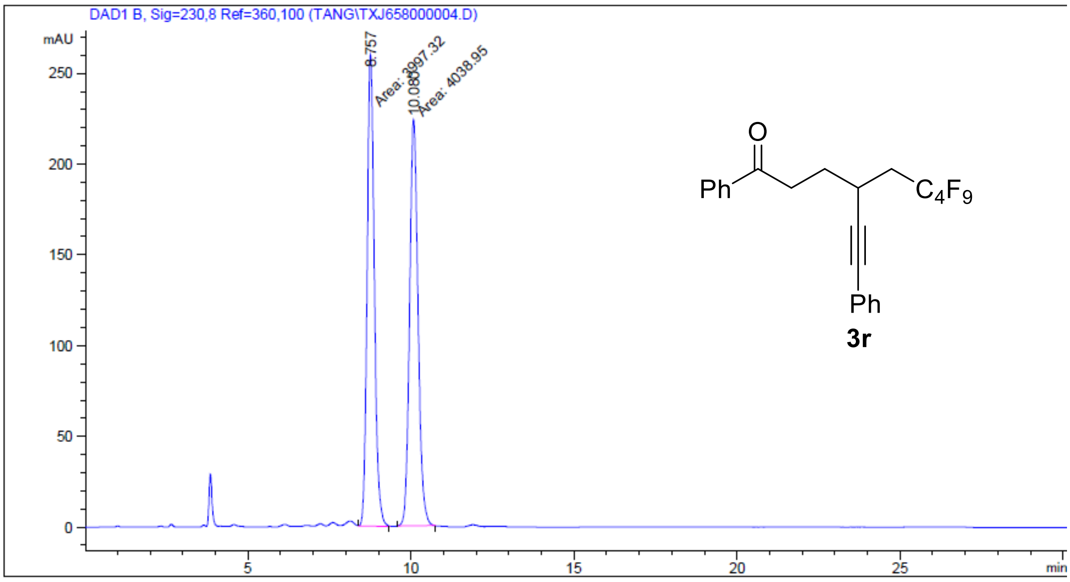

===
Area Percent Report
===

Sorted By : Signal
Multiplier: : 1.0000
Dilution: : 1.0000
Use Multiplier & Dilution Factor with ISTDs

Data File C:\CHEM32\1\DATA\TANG\TXJ745000001.D
Sample Name: TXJ745

===

Acq. Operator : prekel
Acq. Instrument : Instrument 1 Location : Vial 1
Injection Date : 11.04.2017 16:05:49
Inj Volume : 5.0 µl
Acq. Method : C:\CHEM32\1\METHODS\RP CHIRAL\RP CHIRAL_65-35.M
Last changed : 11.04.2017 16:03:39 by prekel
(modified after loading)
Analysis Method : C:\CHEM32\1\METHODS\RP NEUTRAL 1ML\RP_65-35_1.M
Last changed : 24.06.2016 10:35:17 by prekel
Method Info : Standard Method RP_65:35_1
Flow: 1mL/min; 20°C; Injection 5µL; time: 50min
Solvent System:MeCN/H2O=65/35
isocratic

Sample Info : OJ-RH
ACN/H2O

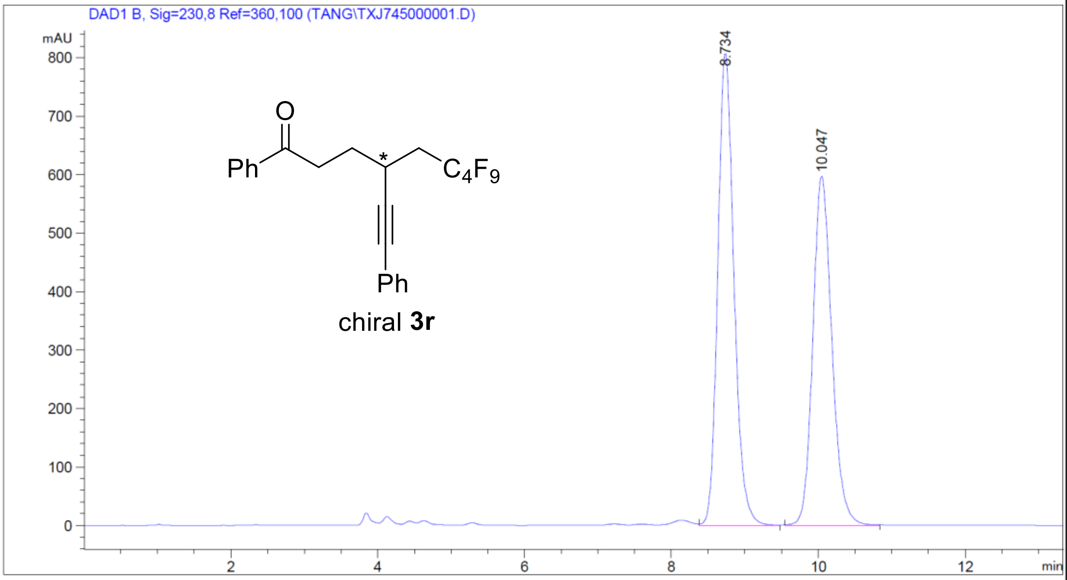

===
Area Percent Report
===

Sorted By : Signal
Multiplier: : 1.0000
Dilution: : 1.0000
Use Multiplier & Dilution Factor with ISTDs

Data File C:\CHEM32\1\DATA\TANG\TXJ745000001.D
Sample Name: TXJ745

Signal 1: DAD1 B, Sig=230,8 Ref=360,100

Peak #	RetTime [min]	Type	Width [min]	Area [mAU*s]	Height [mAU]	Area %
1	8.734	VB	0.2367	1.23521e4	806.58051	53.4568
2	10.047	BB	0.2783	1.07546e4	596.59650	46.5432

Totals : 2.31067e4 1403.17700

===
*** End of Report ***

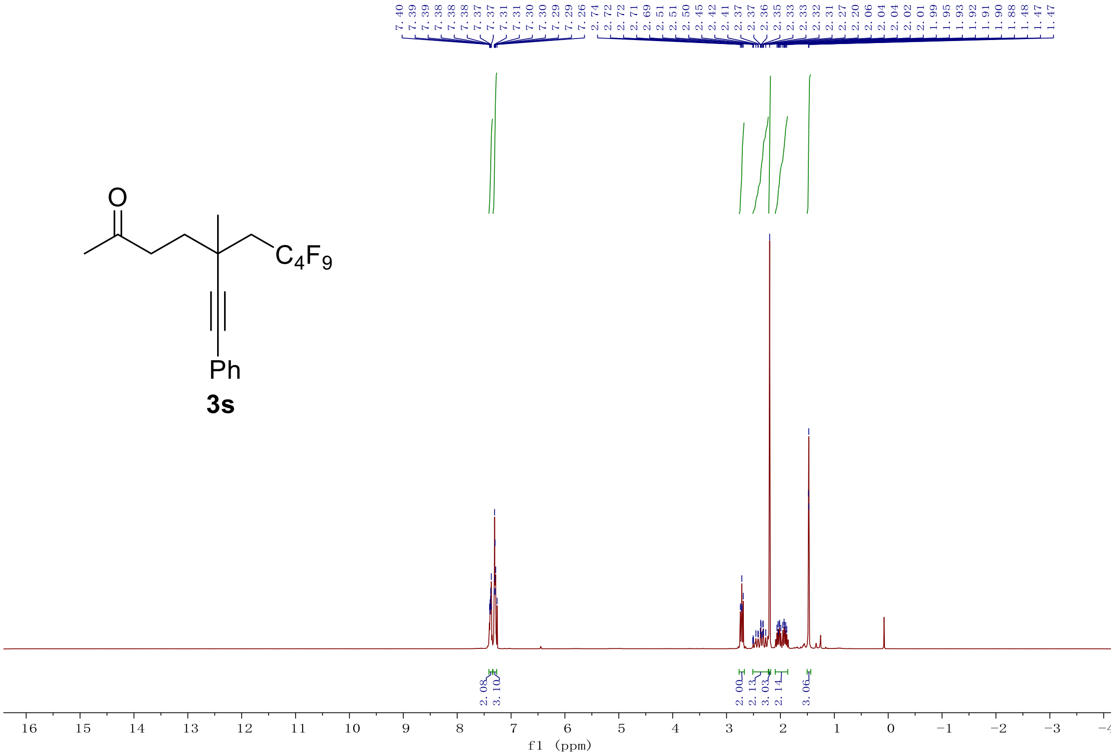

Feb25-2017.14.fid — TXJ-701-H — proton CDCl3 /opt/topspin av1 32

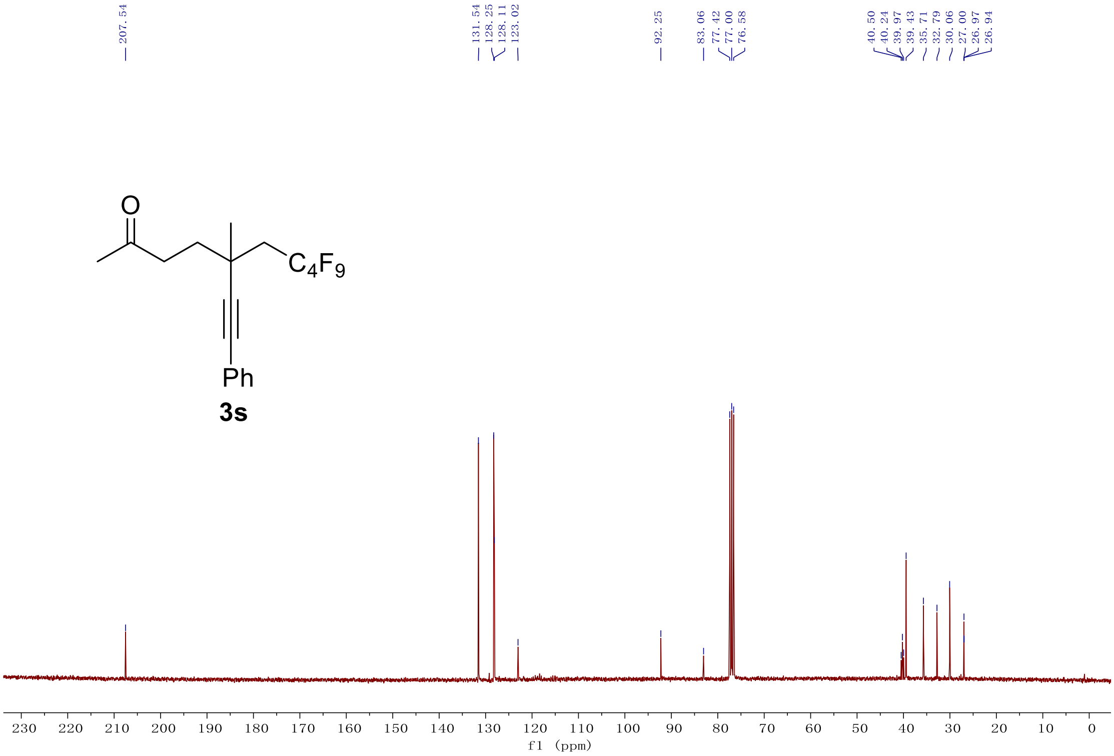

Feb25-2017.11.fid — TXJ-701-C — carbon CDCl3 /opt/topspin av1 32

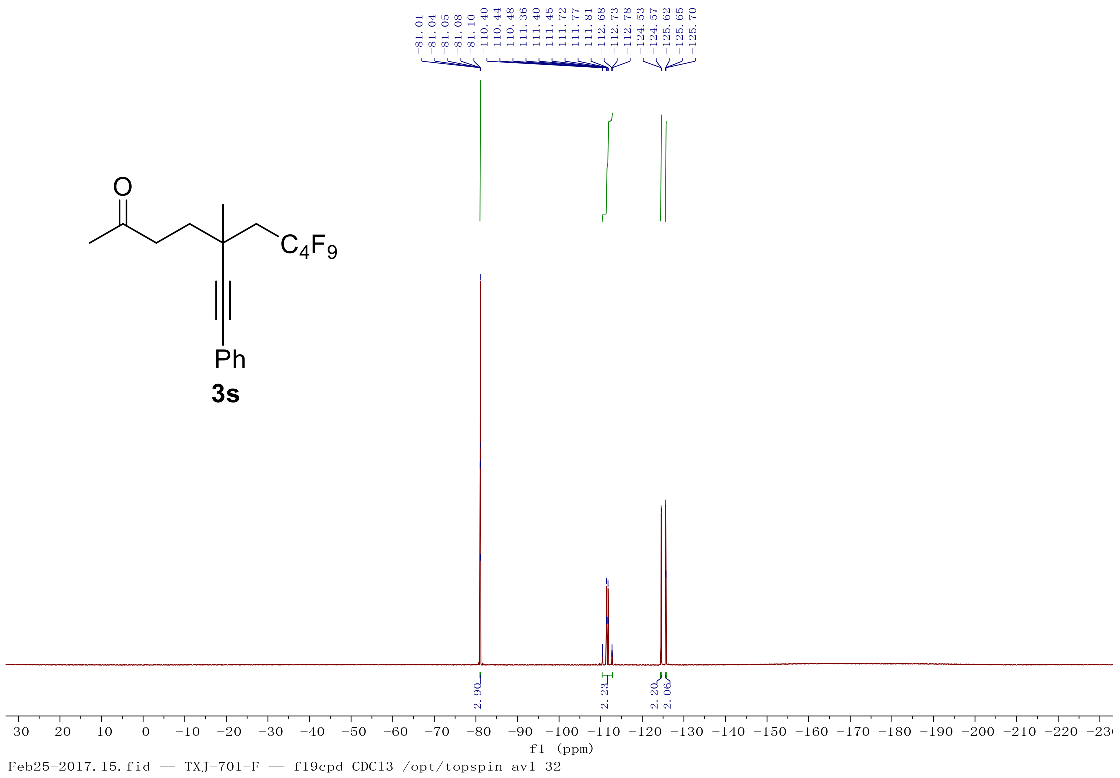
-81.01
-81.04
-81.05
-81.08
-81.10
-110.40
-110.44
-110.48
-111.36
-111.40
-111.45
-111.72
-111.77
-111.81
-112.68
-112.73
-112.78
-124.53
-124.57
-125.62
-125.65
-125.70
O
C4F9
Ph
3s
2.90
2.23
2.20
2.06
f1 (ppm)
Feb25-2017.15.fid — TXJ-701-F — f19cpd CDCl3 /opt/topspin av1 32

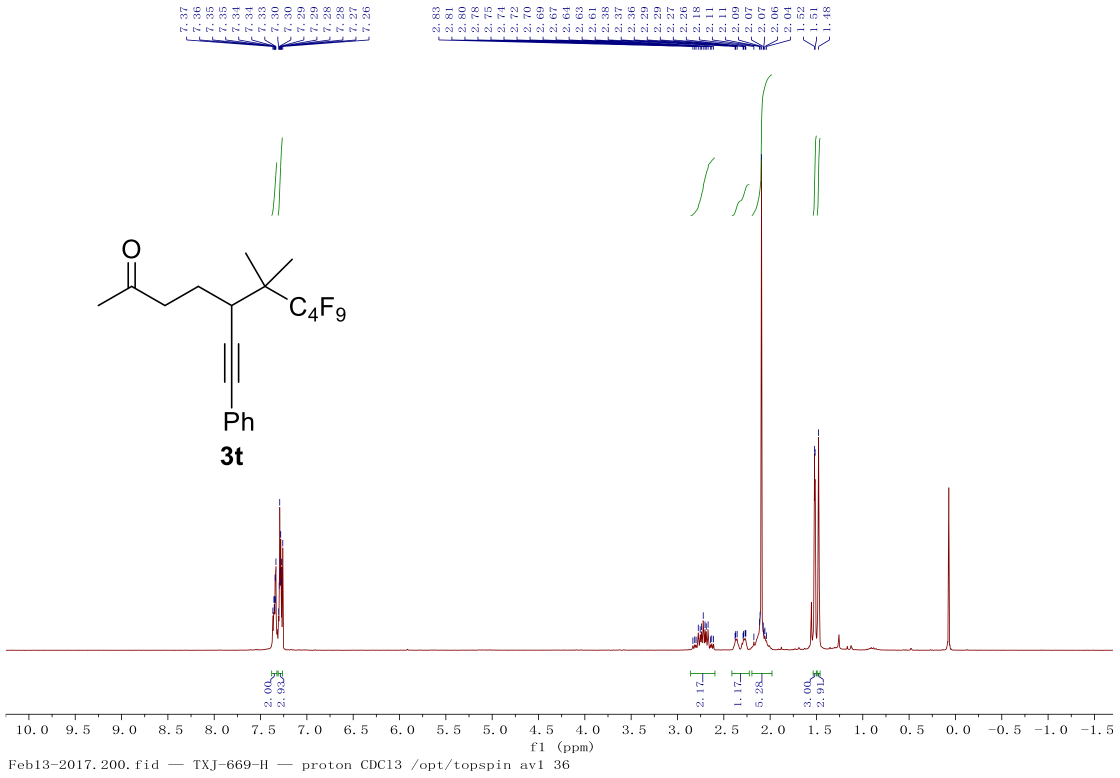
7.37
7.36
7.35
7.34
7.34
7.33
7.30
7.30
7.29
7.29
7.28
7.28
7.27
7.26
2.83
2.81
2.80
2.78
2.75
2.74
2.72
2.70
2.69
2.67
2.64
2.63
2.61
2.38
2.37
2.36
2.29
2.29
2.27
2.26
2.18
2.11
2.09
2.07
2.06
2.04
1.52
1.51
1.48
O
C4F9
Ph
3t
2.00
2.93
2.17
1.17
5.28
3.00
2.91
f1 (ppm)
Feb13-2017.200.fid — TXJ-669-H — proton CDCl3 /opt/topspin av1 36

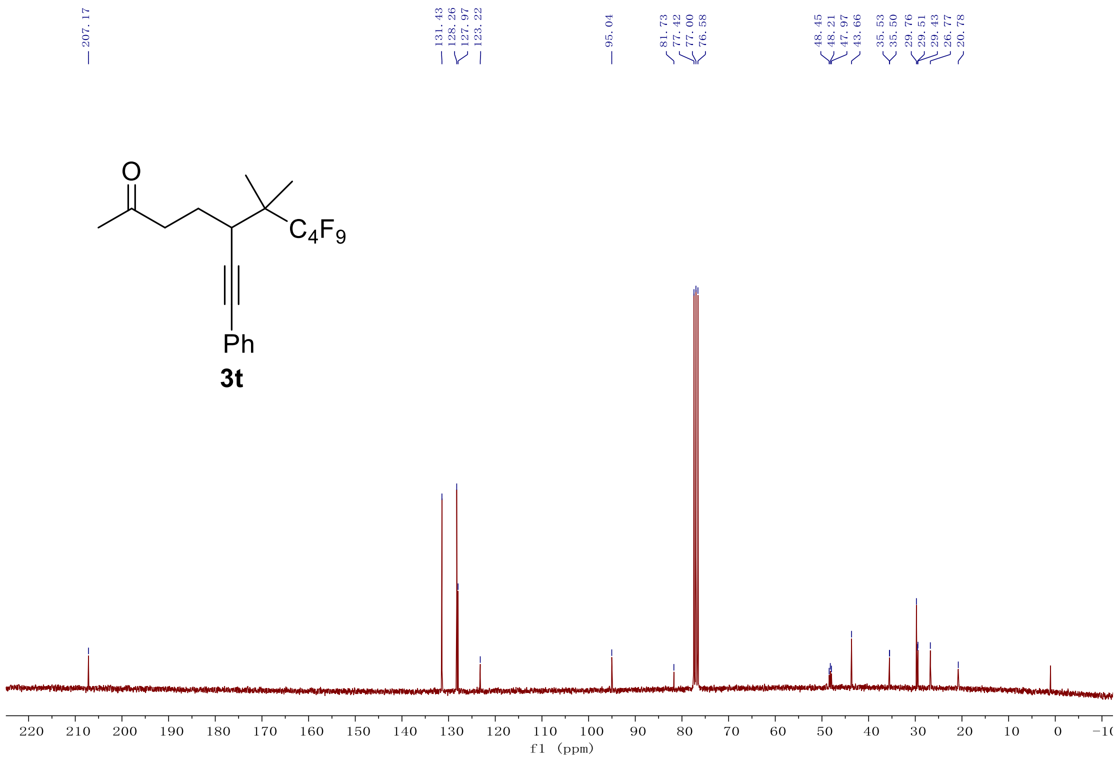

Feb13-2017.201.fid — TXJ-669-C — carbon CDCl3 /opt/topspin av1 36

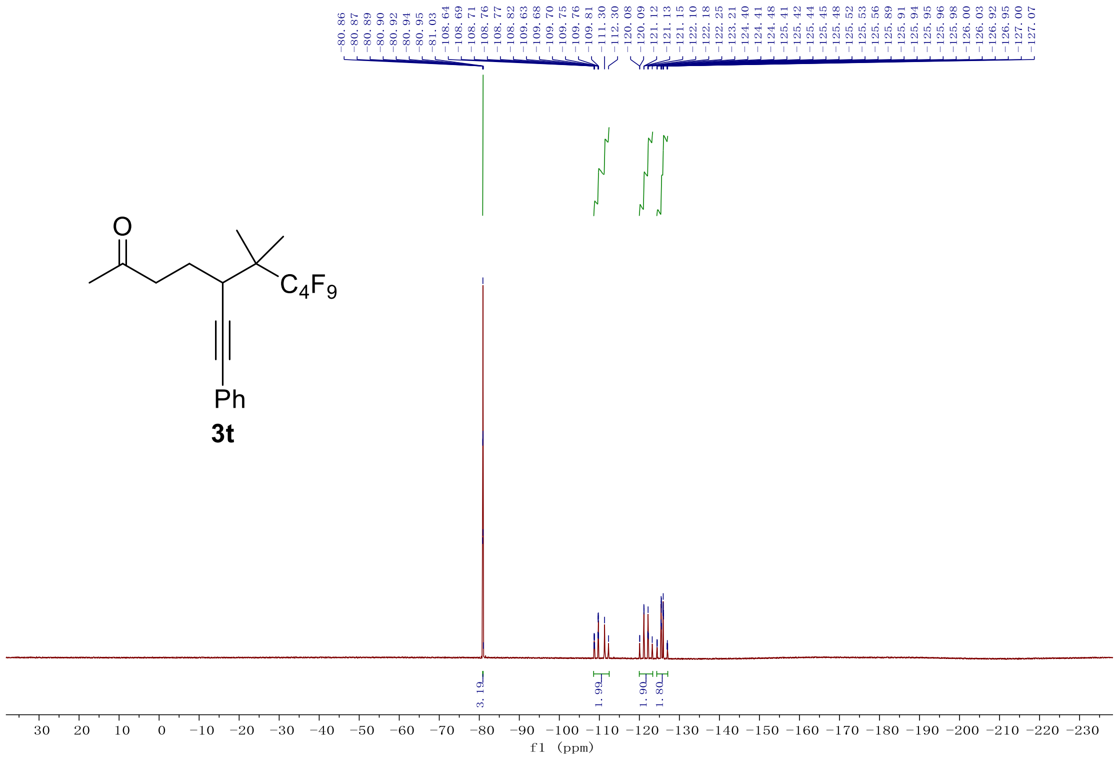

Feb10-2017.881.fid — stu tang txj 669 — f19cpd CDCl3 /opt/topspin av1 59

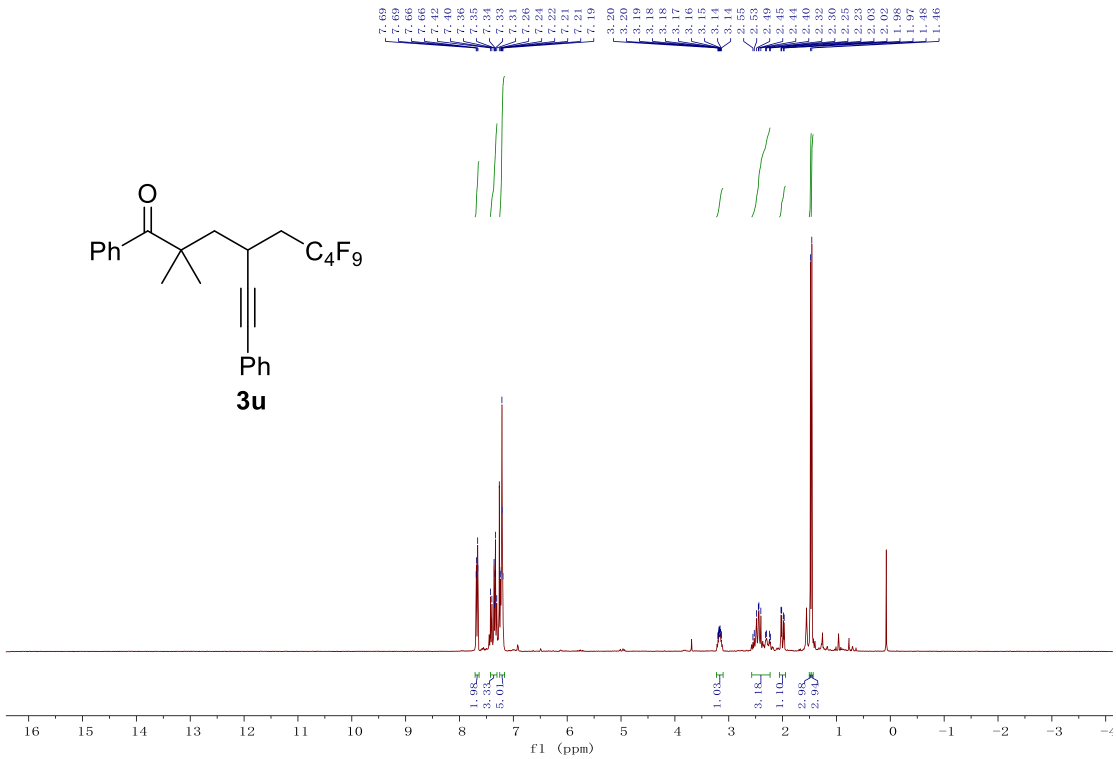

Feb19-2017.50.fid — TXJ-690-H — proton CDCl3 /opt/topspin av1 50

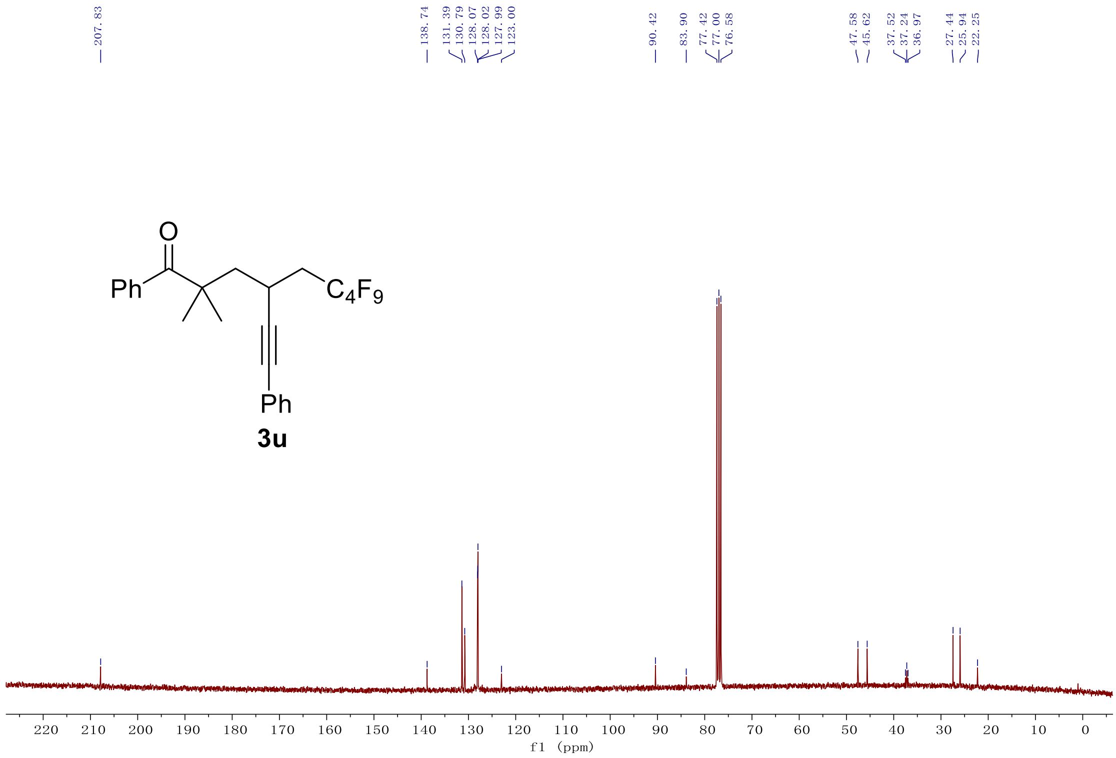

Feb19-2017.51.fid — TXJ-690-C — carbon CDCl3 /opt/topspin av1 50

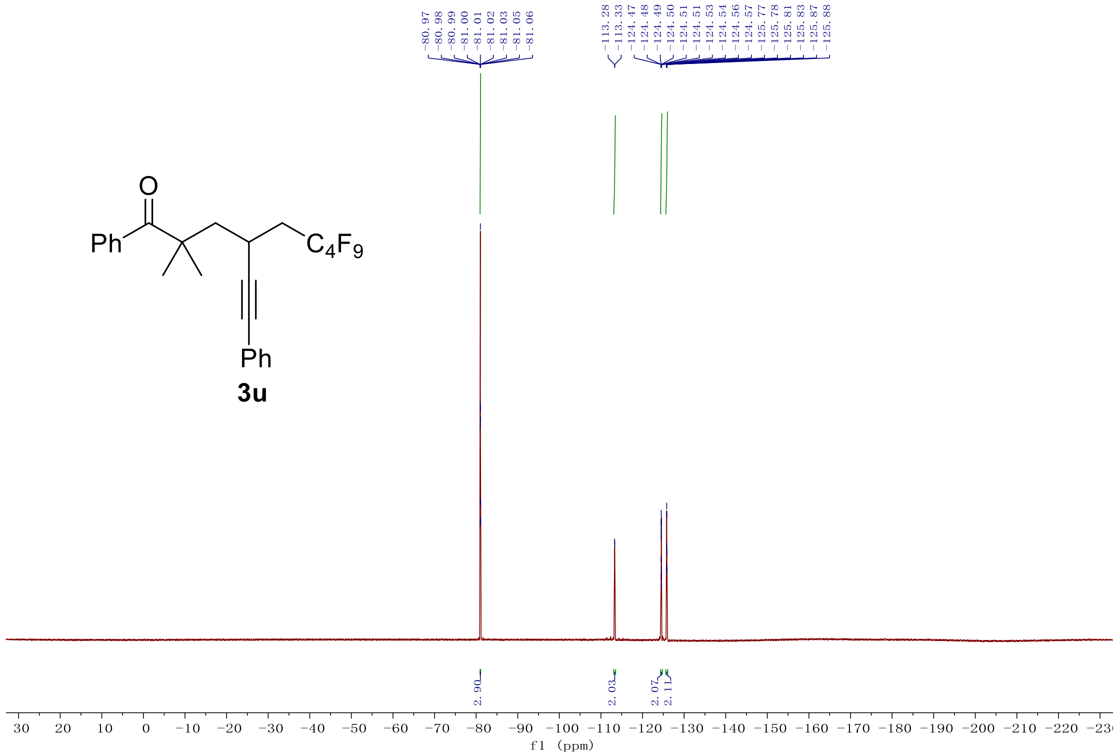

Feb19-2017.55.fid — TXJ-690-F — f19cpd CDCl3 /opt/topspin av1 50

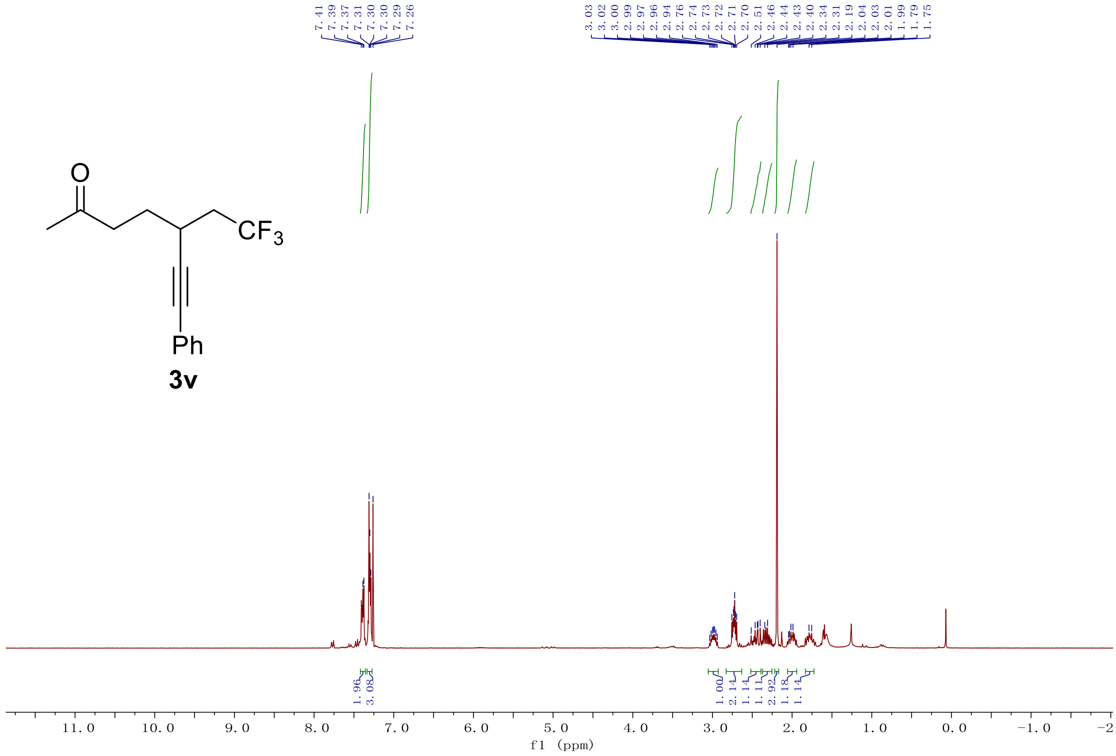

May10-2017.230.fid — TXJ-767-H — proton CDCl3 /opt/topspin av1 30

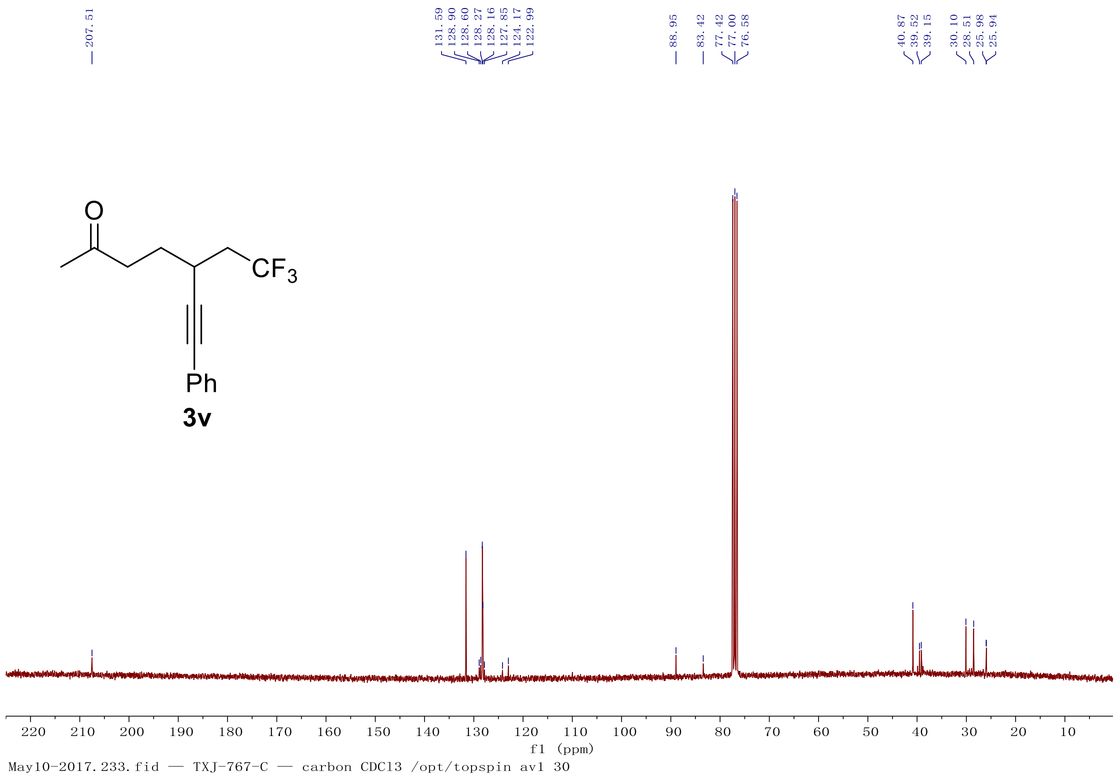

207.51
131.59
128.90
128.60
128.27
128.16
127.85
124.17
122.99
88.95
83.42
77.42
77.00
76.58
40.87
39.52
39.15
30.10
28.51
25.98
25.94
O
CF3
Ph
3v
f1 (ppm)
May10-2017.233.fid — TXJ-767-C — carbon CDCl3 /opt/topspin av1 30

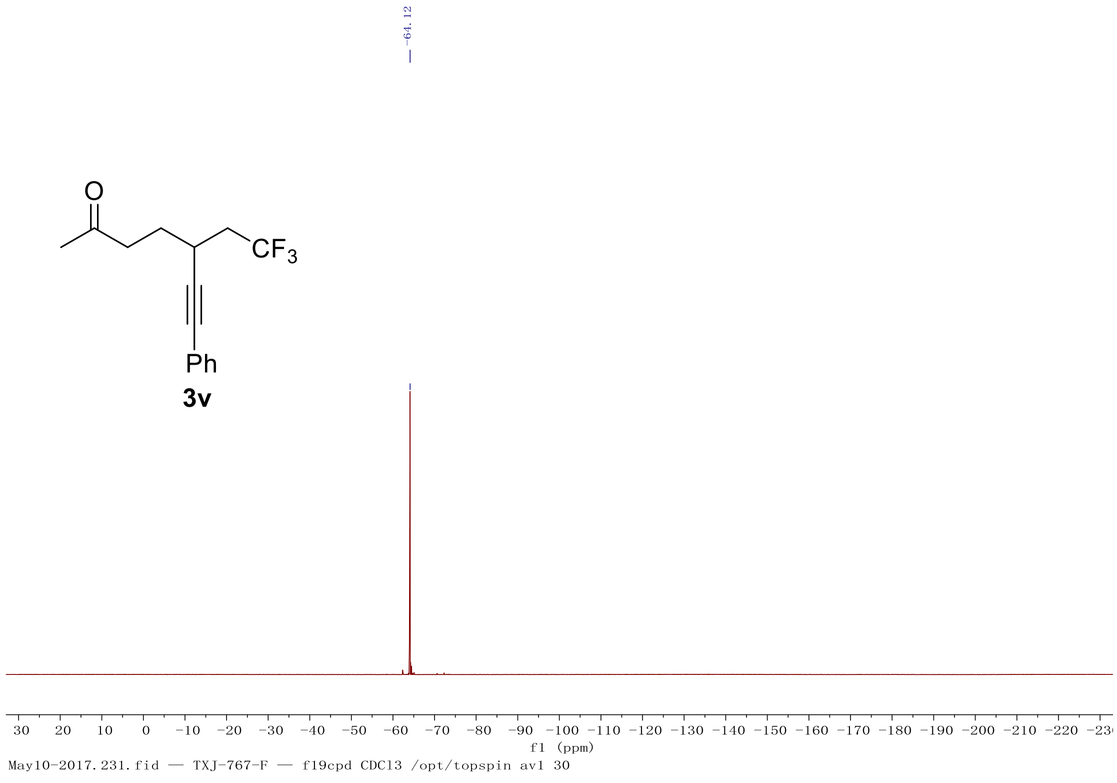

-64.12
O
CF3
Ph
3v
f1 (ppm)
May10-2017.231.fid — TXJ-767-F — f19cpd CDCl3 /opt/topspin av1 30

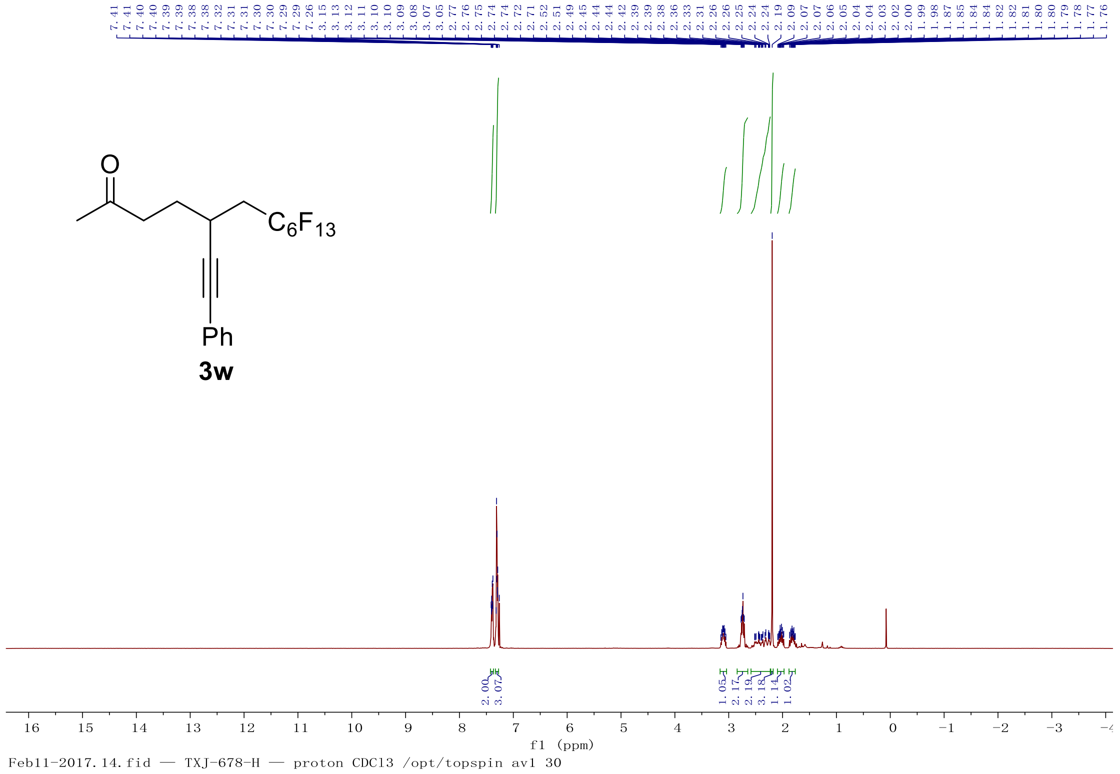
7.41 7.41 7.40 7.39 7.39 7.38 7.38 7.32 7.31 7.31 7.30 7.30 7.29 7.29 7.26 3.15 3.13 3.12 3.11 3.10 3.10 3.09 3.08 3.07 3.05 2.77 2.76 2.75 2.74 2.74 2.72 2.71 2.52 2.51 2.49 2.45 2.44 2.44 2.42 2.39 2.39 2.38 2.36 2.33 2.31 2.26 2.26 2.25 2.24 2.24 2.19 2.09 2.07 2.07 2.06 2.05 2.04 2.04 2.03 2.02 2.00 1.99 1.98 1.87 1.85 1.84 1.84 1.82 1.81 1.80 1.79 1.78 1.77 1.76
O
C6F13
Ph
3w
2.00
3.07
1.05
2.17
2.19
3.18
1.14
1.02
16 15 14 13 12 11 10 9 8 7 6 5 4 3 2 1 0 −1 −2 −3
f1 (ppm)
Feb11-2017.14.fid — TXJ-678-H — proton CDCl3 /opt/topspin av1 30

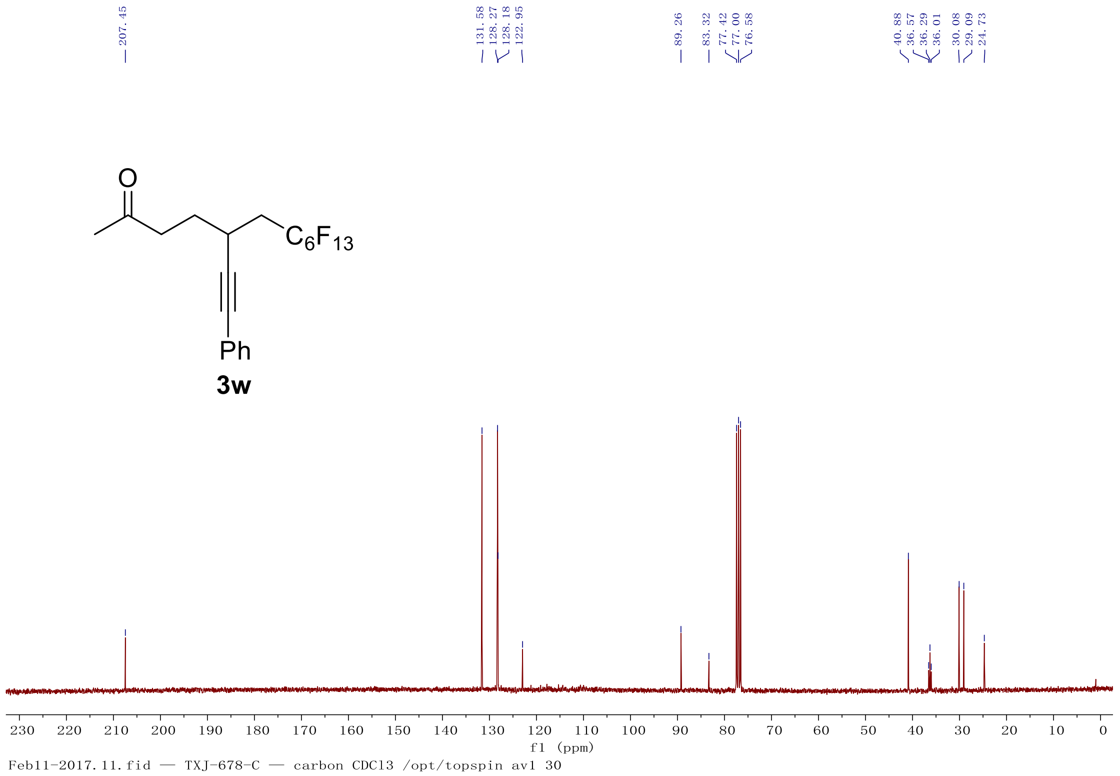
207.45
131.58 128.27 128.18 122.95
89.26
83.32 77.42 77.00 76.58
40.88 36.57 36.29 36.01
30.08 29.09 24.73
O
C6F13
Ph
3w
230 220 210 200 190 180 170 160 150 140 130 120 110 100 90 80 70 60 50 40 30 20 10 0
f1 (ppm)
Feb11-2017.11.fid — TXJ-678-C — carbon CDCl3 /opt/topspin av1 30

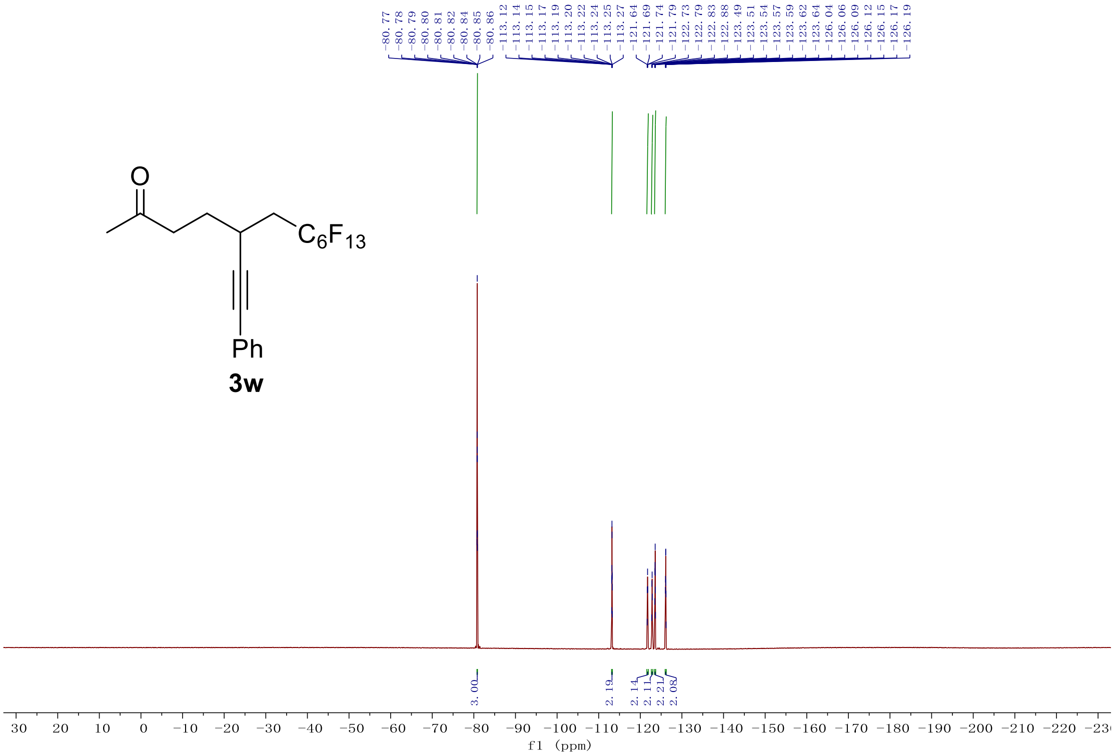

Feb11-2017.15.fid — TXJ-678-F — f19cpd CDCl3 /opt/topspin av1 30

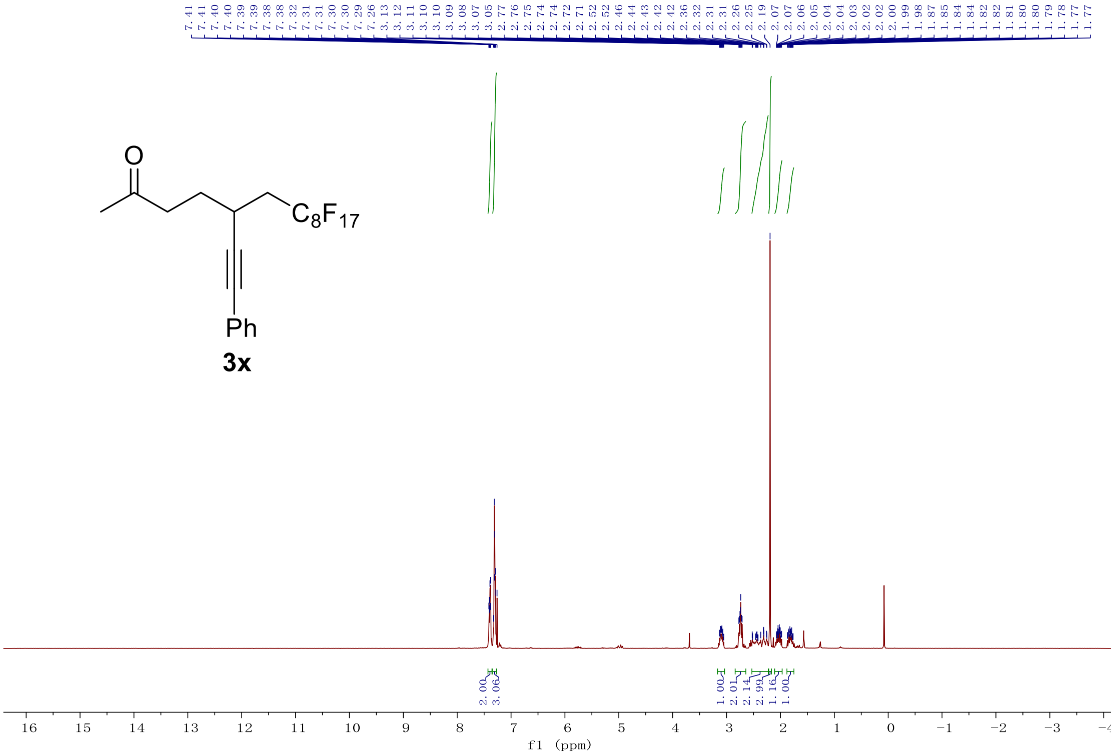

Feb11-2017.24.fid — TXJ-679-H — proton CDCl3 /opt/topspin av1 31

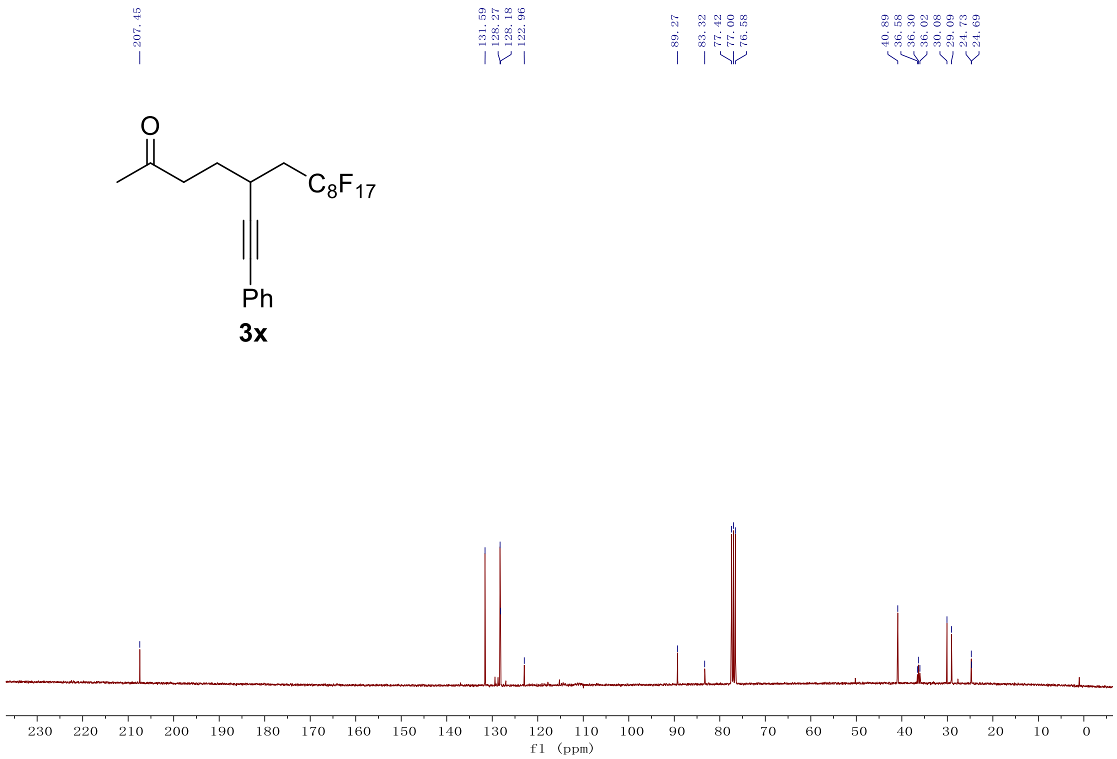

Feb13-2017.191.fid — TXJ-679-C — carbon CDCl3 /opt/topspin av1 35

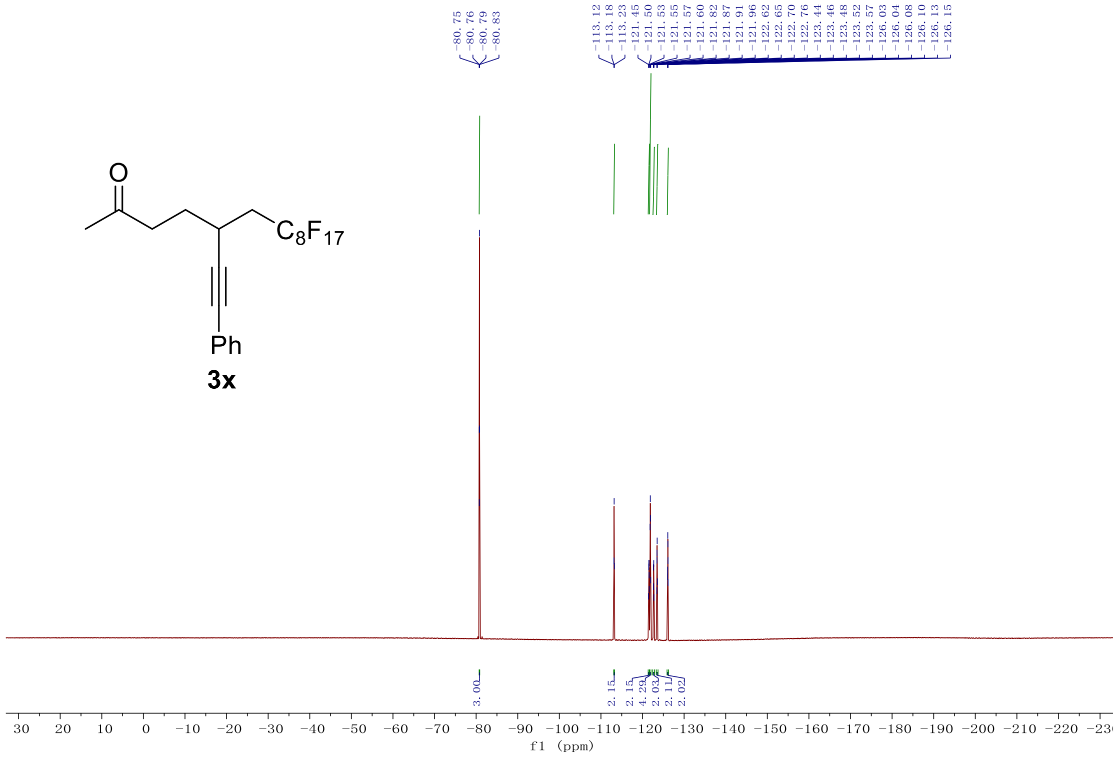

Feb11-2017.25.fid — TXJ-679-F — f19cpd CDCl3 /opt/topspin av1 31

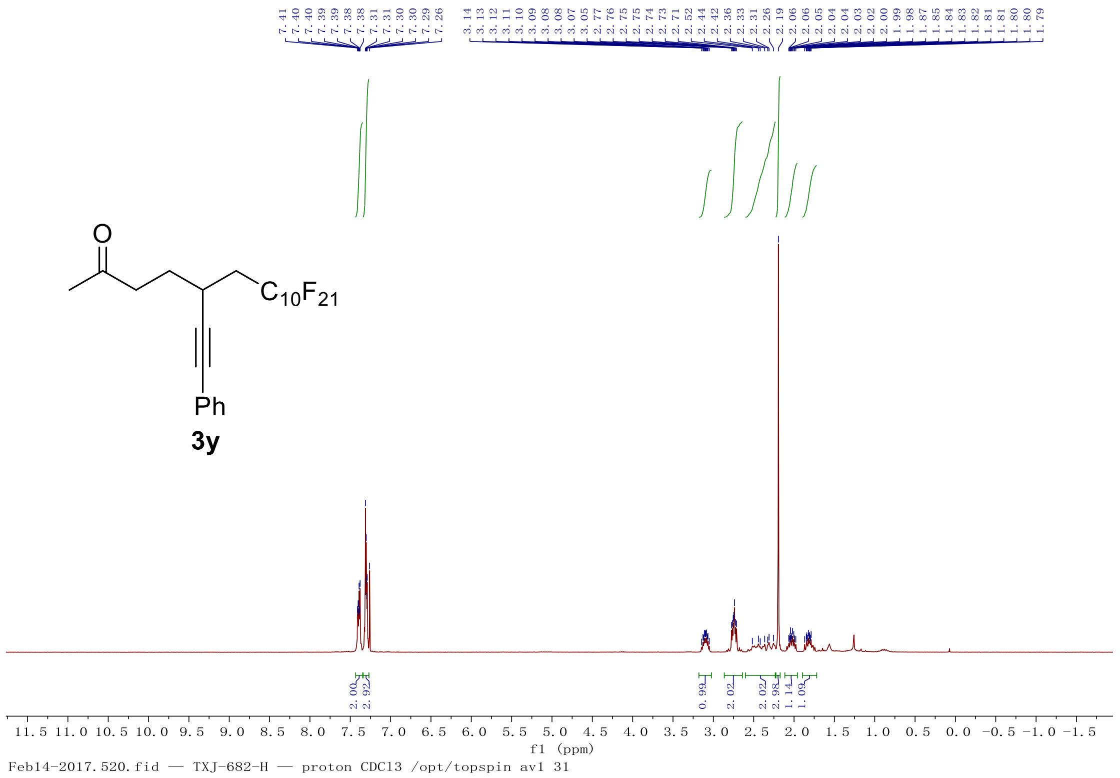

C10F21
Ph
3y
2.00
2.92
0.99
2.02
2.02
2.98
1.14
1.09
f1 (ppm)
Feb14-2017.520.fid — TXJ-682-H — proton CDCl3 /opt/topspin av1 31

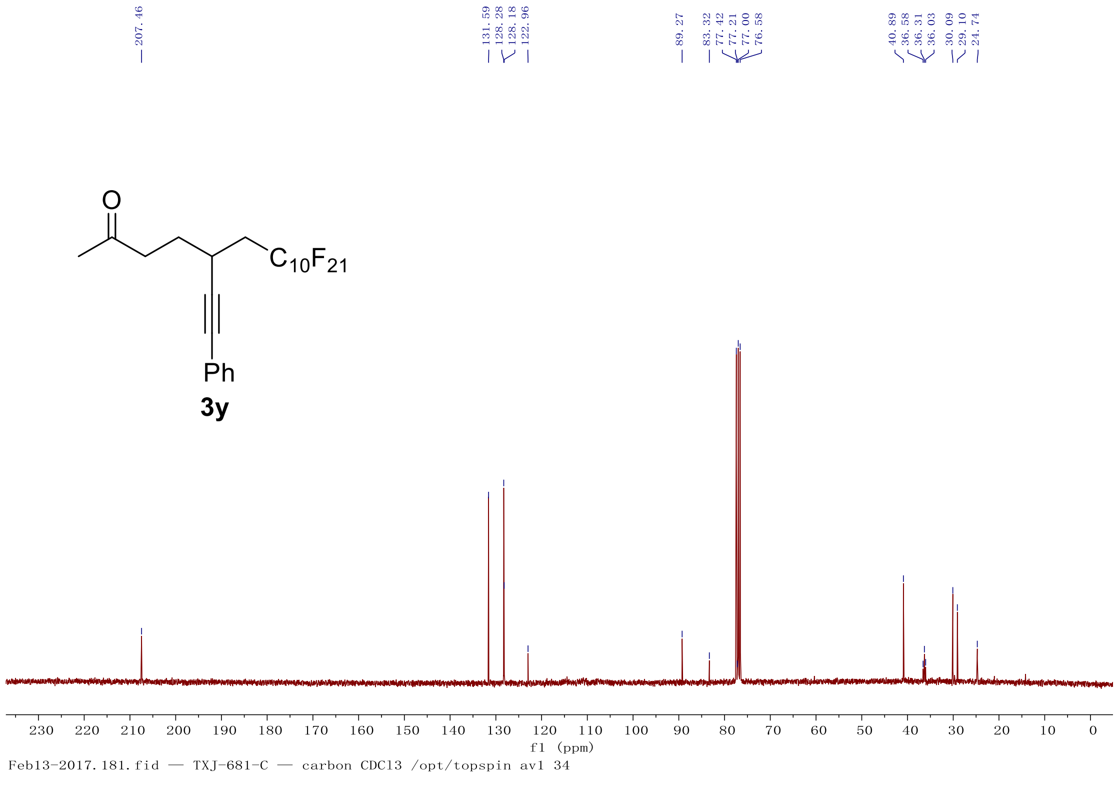

207.46
131.59
128.28
128.18
122.96
89.27
83.32
77.42
77.21
77.00
76.58
40.89
36.58
36.31
36.03
30.09
29.10
24.74
C10F21
Ph
3y
f1 (ppm)
Feb13-2017.181.fid — TXJ-681-C — carbon CDCl3 /opt/topspin av1 34

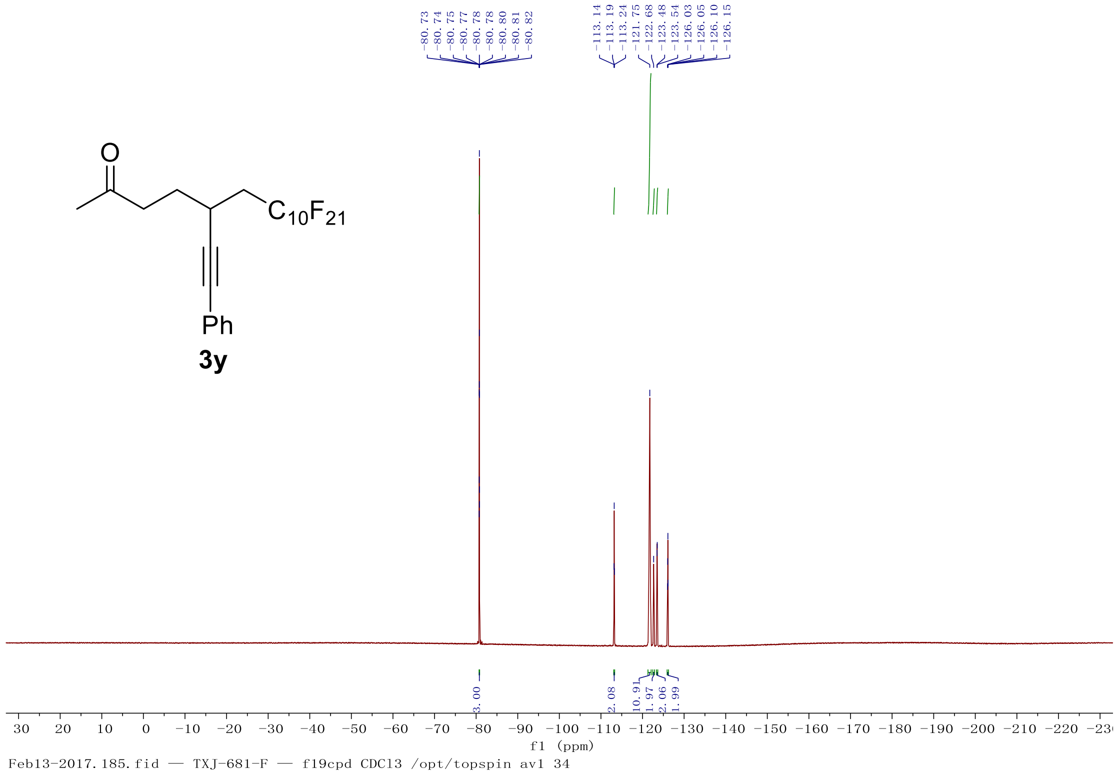

O
$C_{10}F_{21}$
Ph
f1 (ppm)
Feb13-2017.185.fid — TXJ-681-F — f19cpd CDCl3 /opt/topspin av1 34

3y

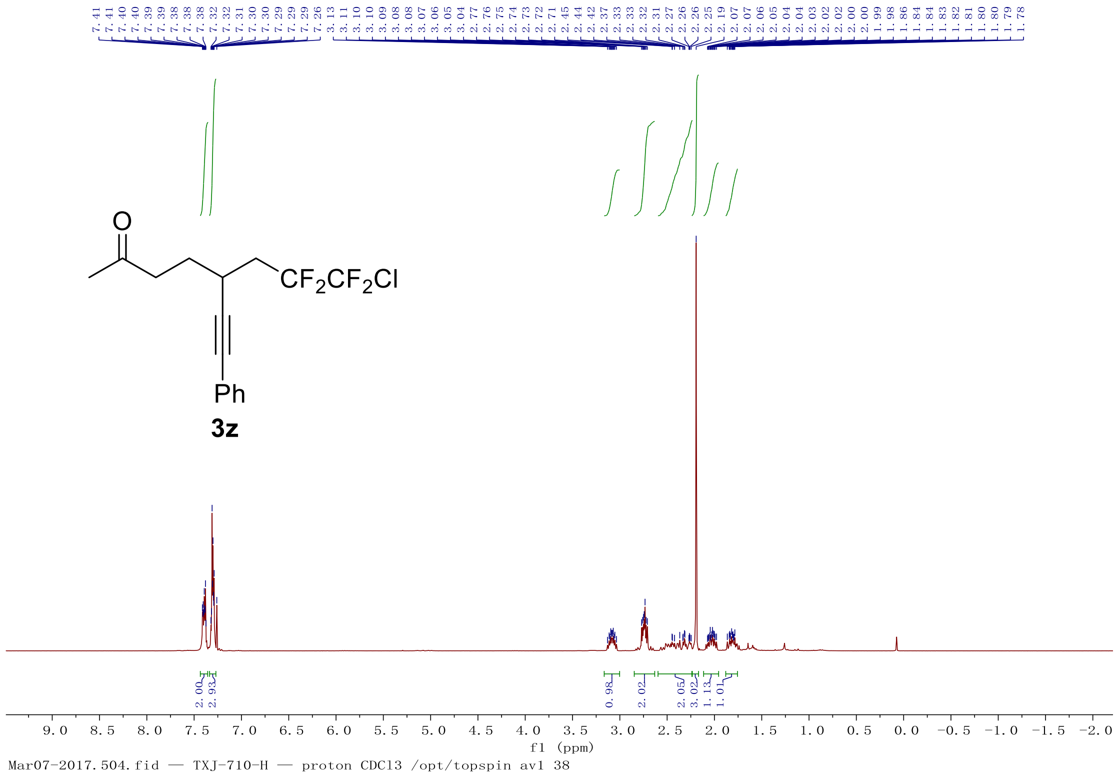

O
CF_2CF_2Cl
Ph
f1 (ppm)
Mar07-2017.504.fid — TXJ-710-H — proton CDCl3 /opt/topspin av1 38

3z

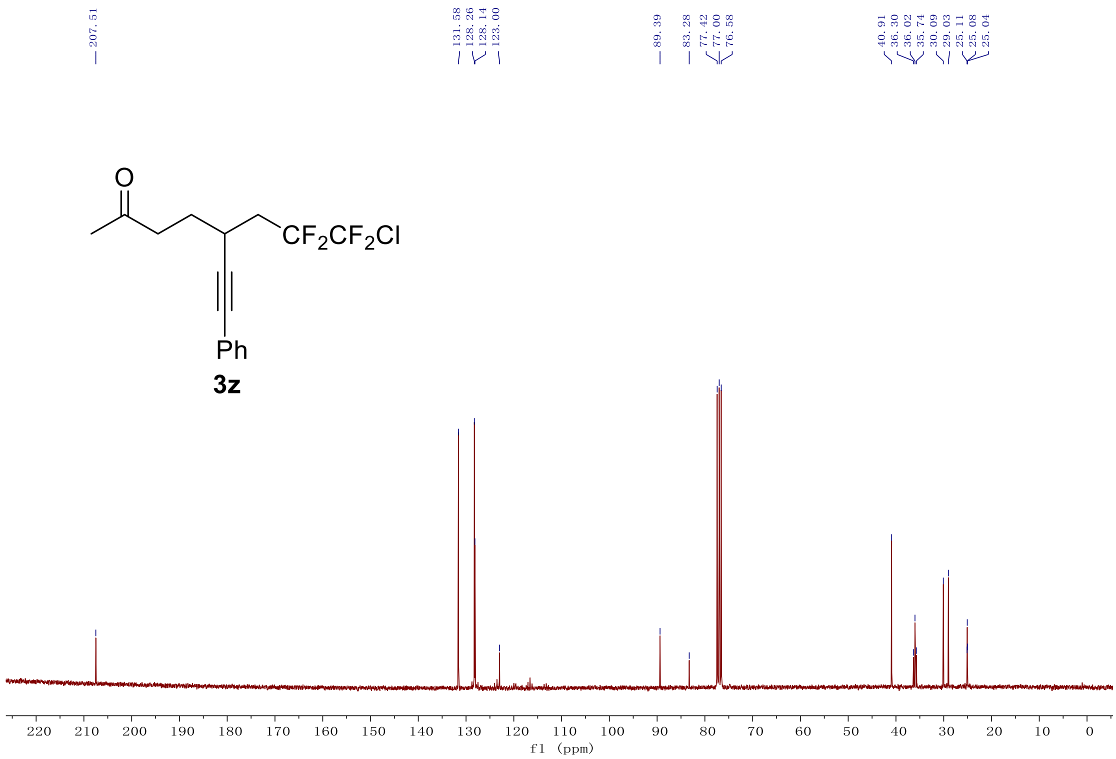

Mar07-2017.501.fid — TXJ-710-C — carbon CDCl3 /opt/topspin av1 38

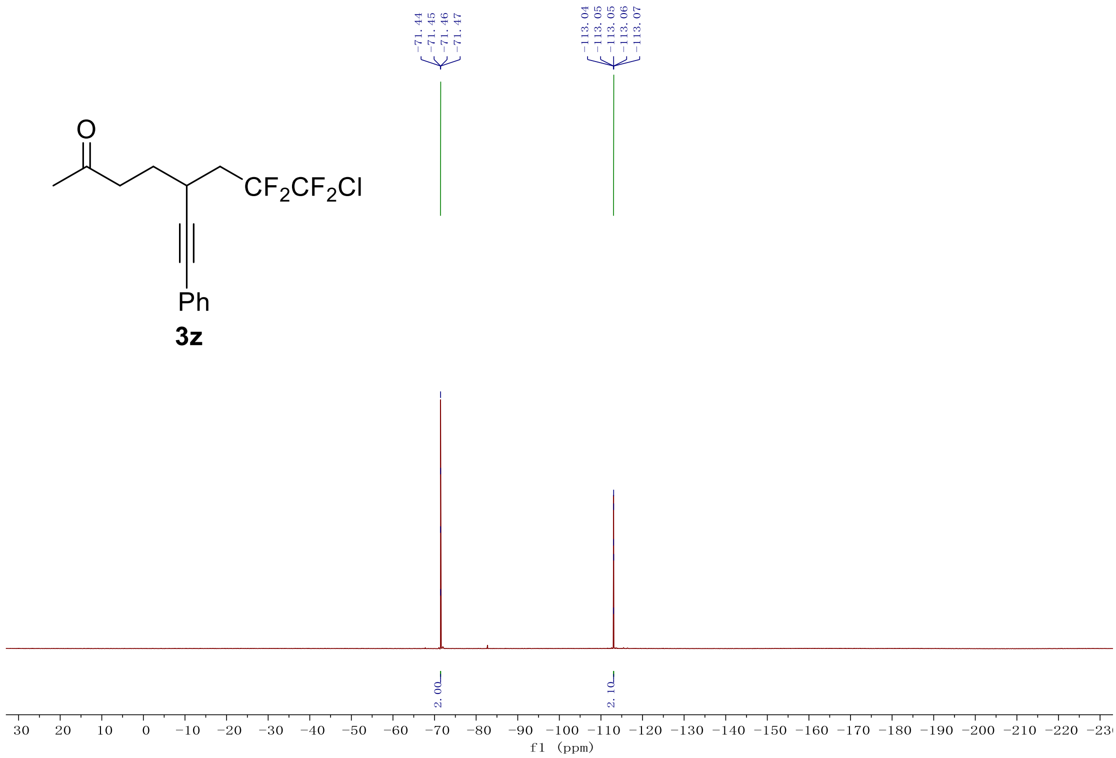

Mar07-2017.505.fid — TXJ-710-F — f19cpd CDCl3 /opt/topspin av1 38

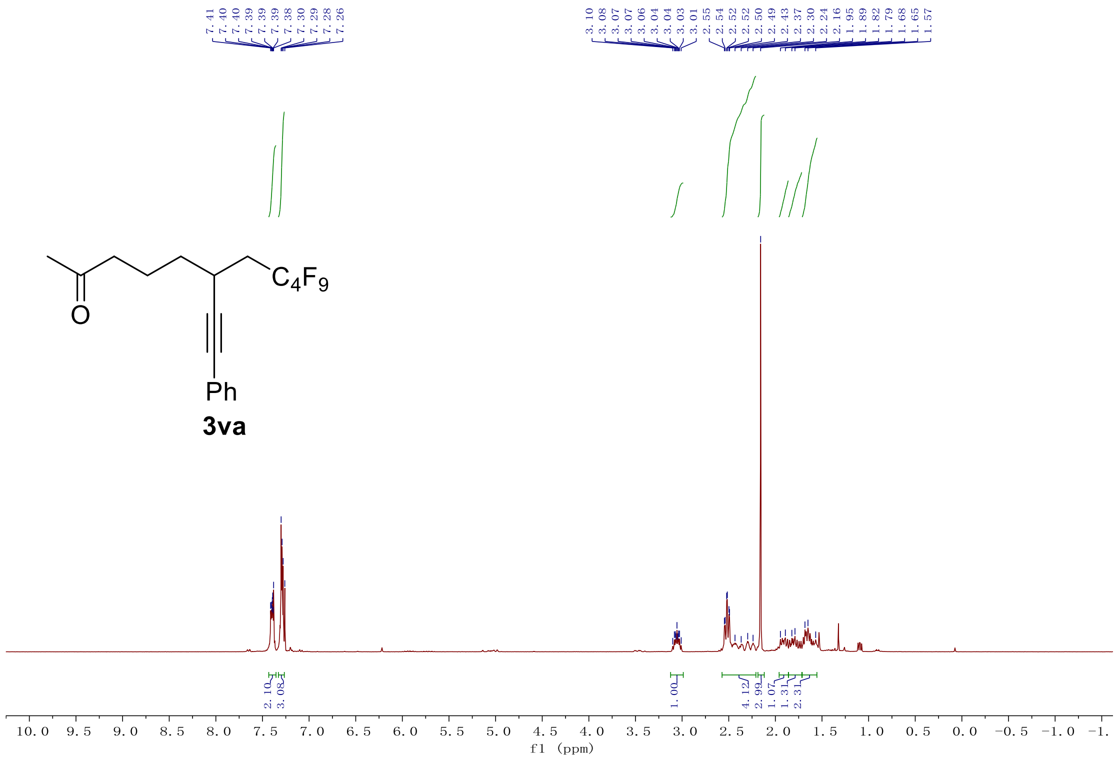

Feb23-2017.124.fid — TXJ-700-H — proton CDCl3 /opt/topspin av1 32

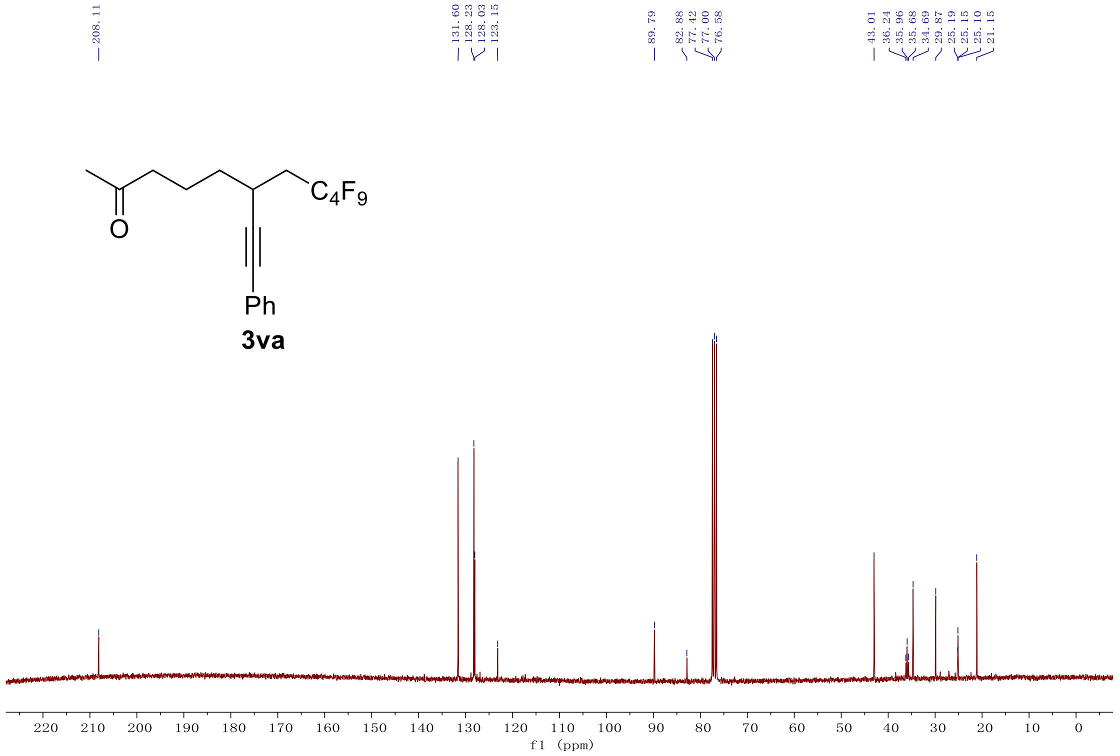

Feb23-2017.121.fid — TXJ-700-C — carbon CDCl3 /opt/topspin av1 32

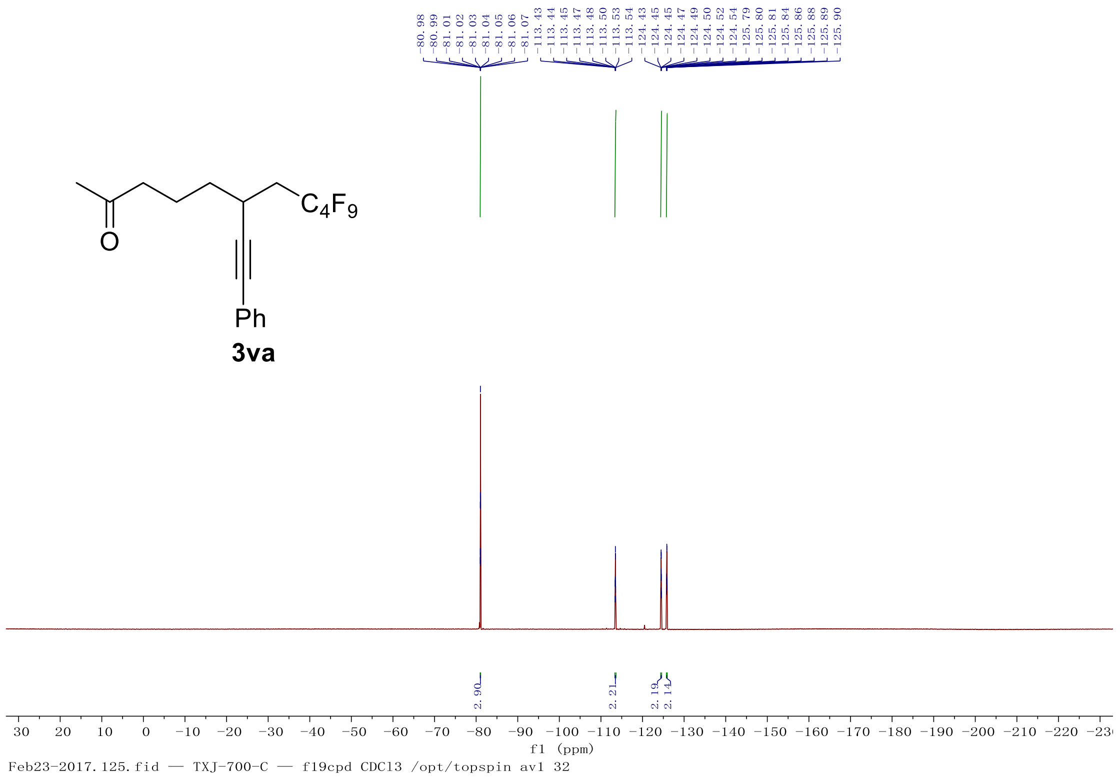

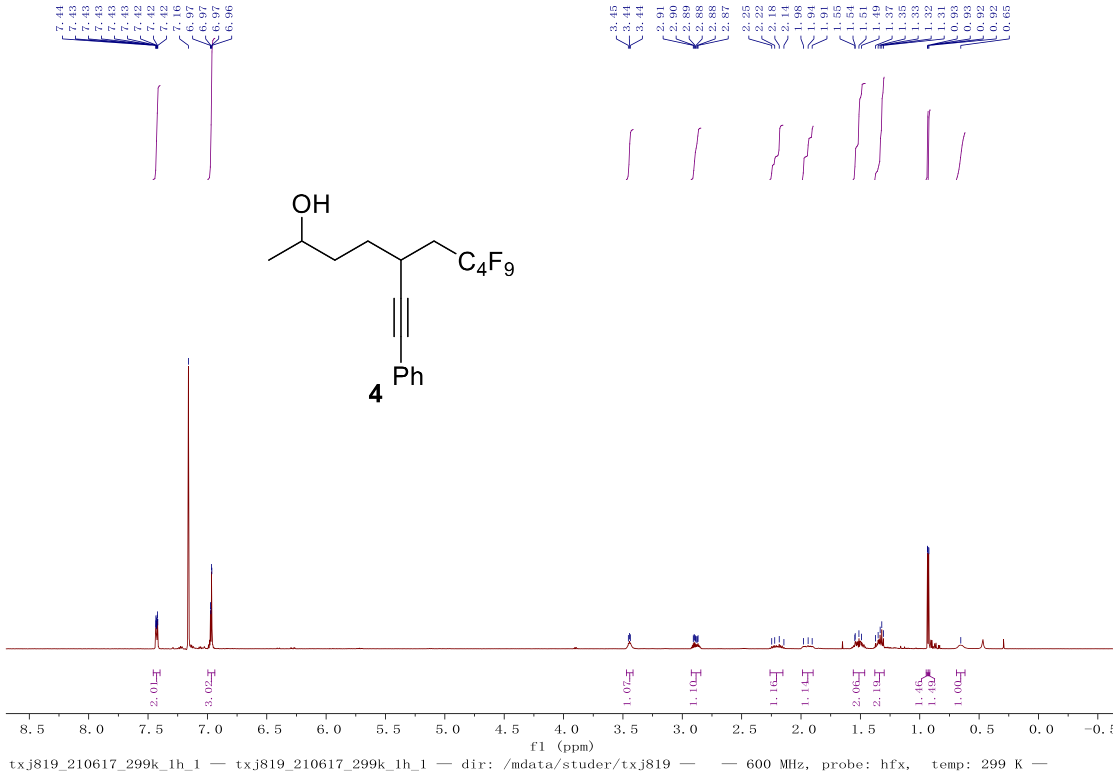

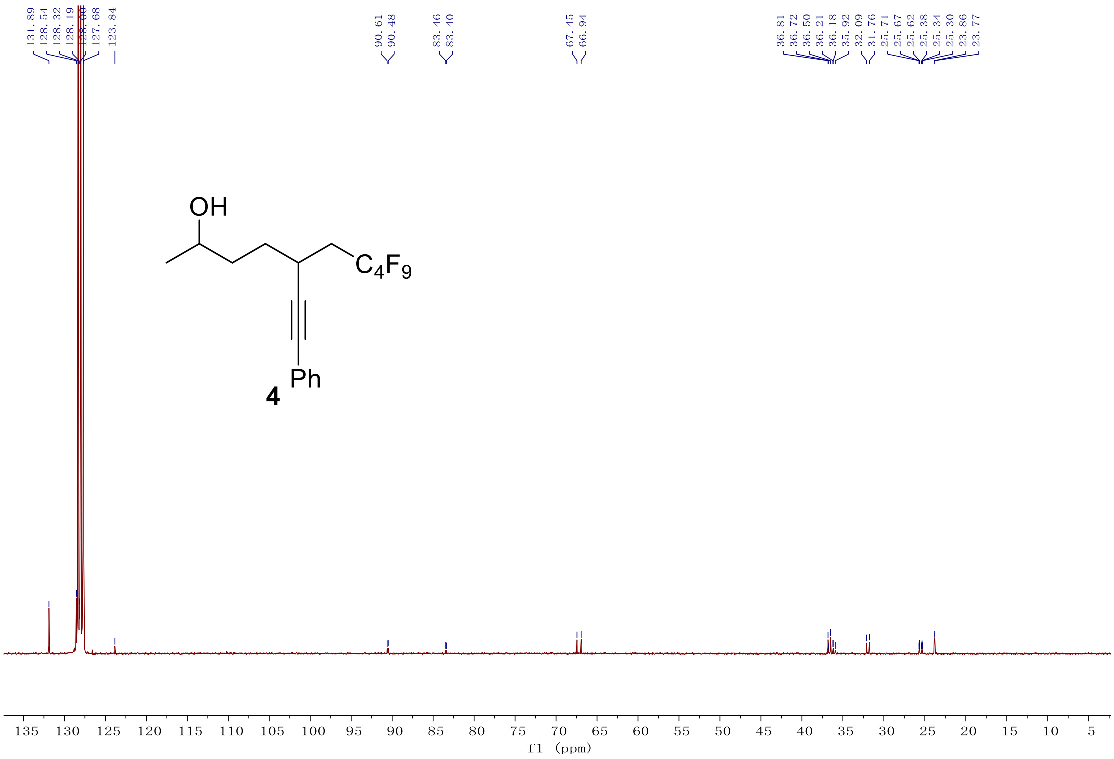

Jun22-2017.91.fid — TXJ-819-C — carbon C6D6 /opt/topspin av1 30

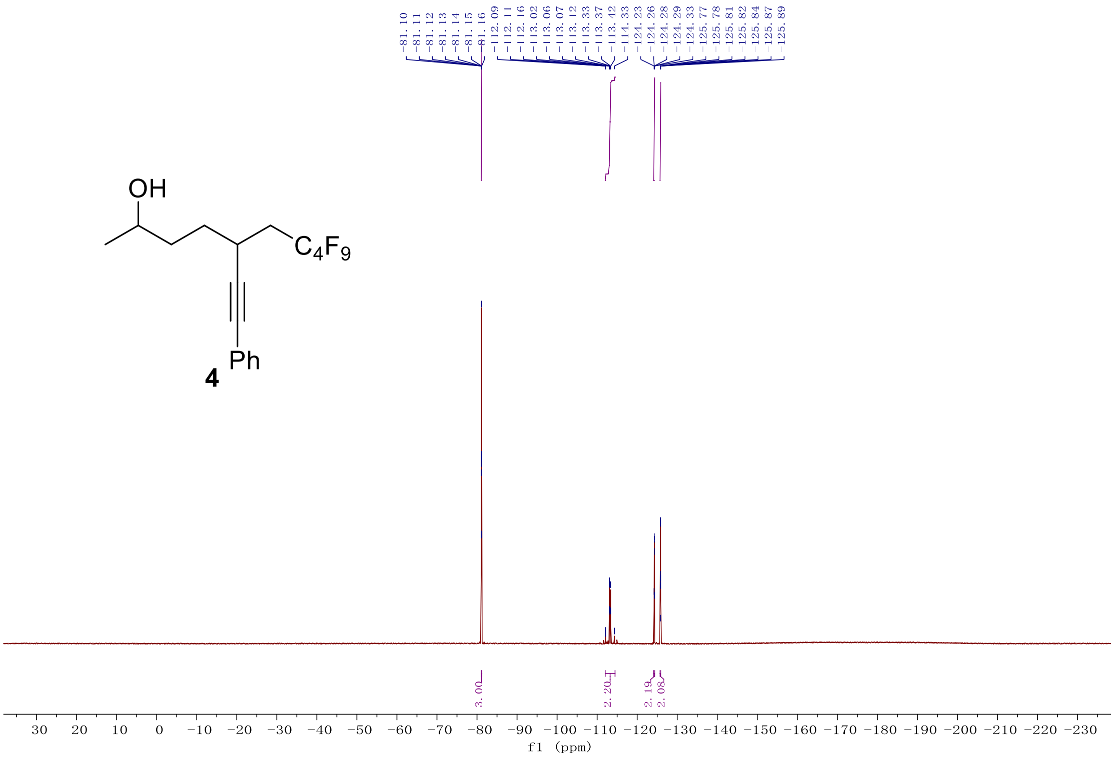

Jun20-2017.703.fid — stu jang txj 819 — f19cpd C6D6 /opt/topspin av1 23

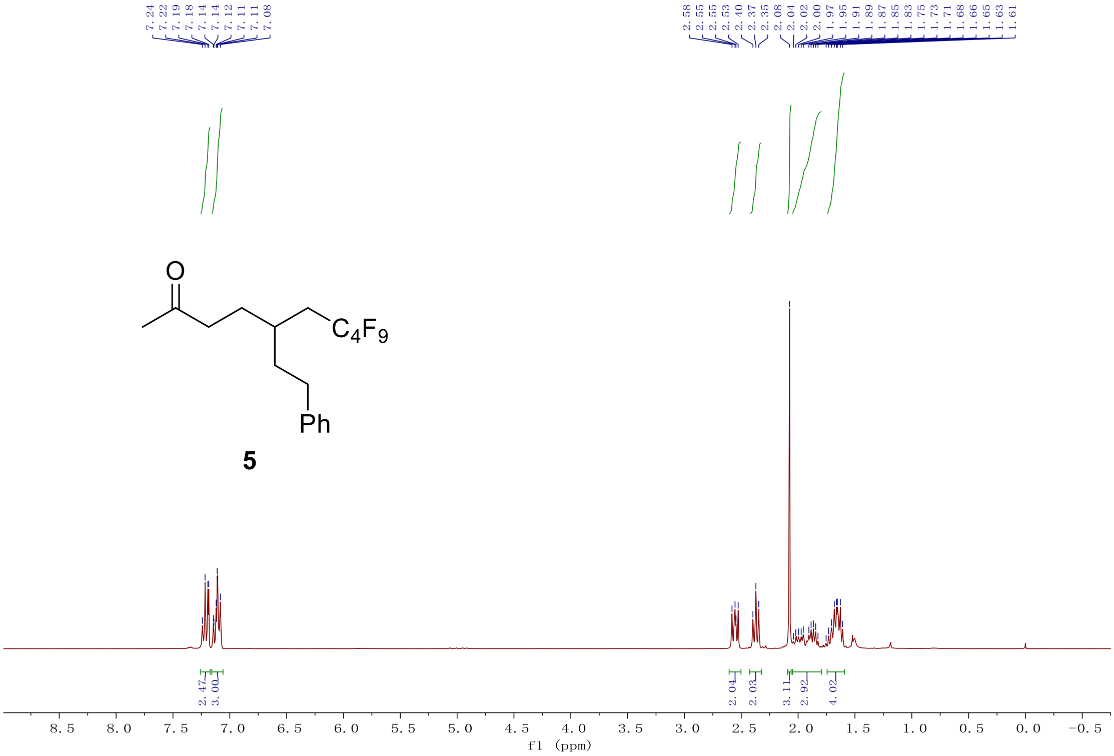

Jul14-2017.300.fid — TXJ-871-H — proton CDCl3 /opt/topspin av1 44

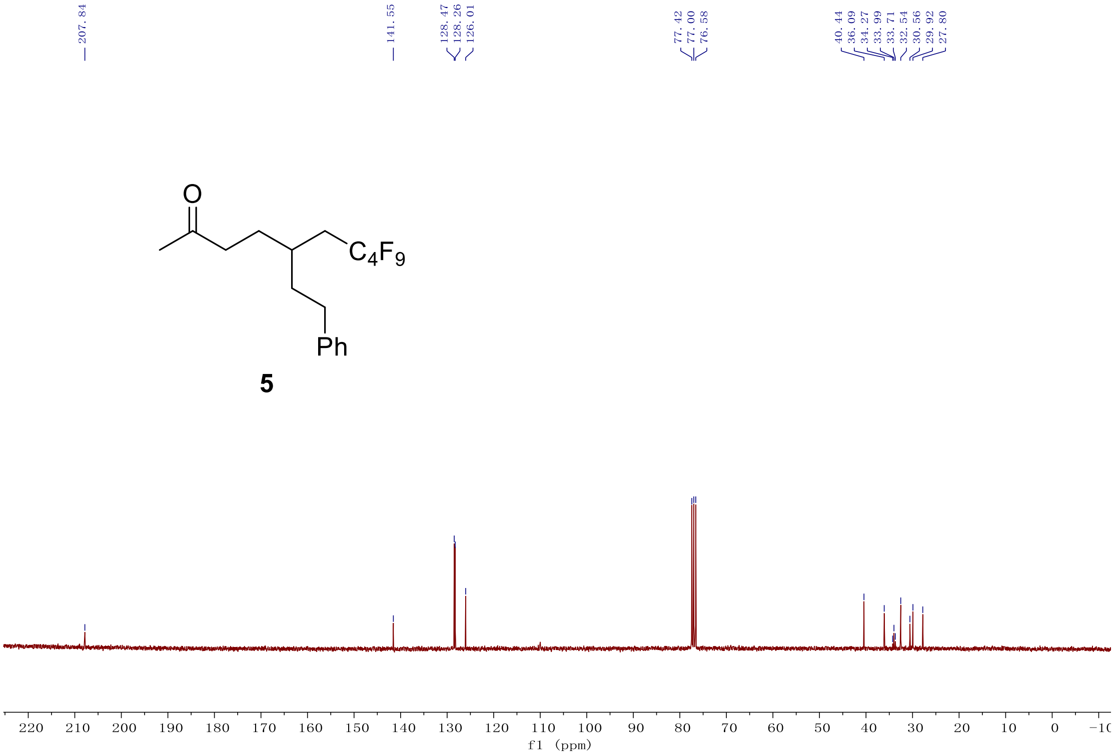

Jul14-2017.303.fid — TXJ-871-C — carbon_256 CDCl3 /opt/topspin av1 44

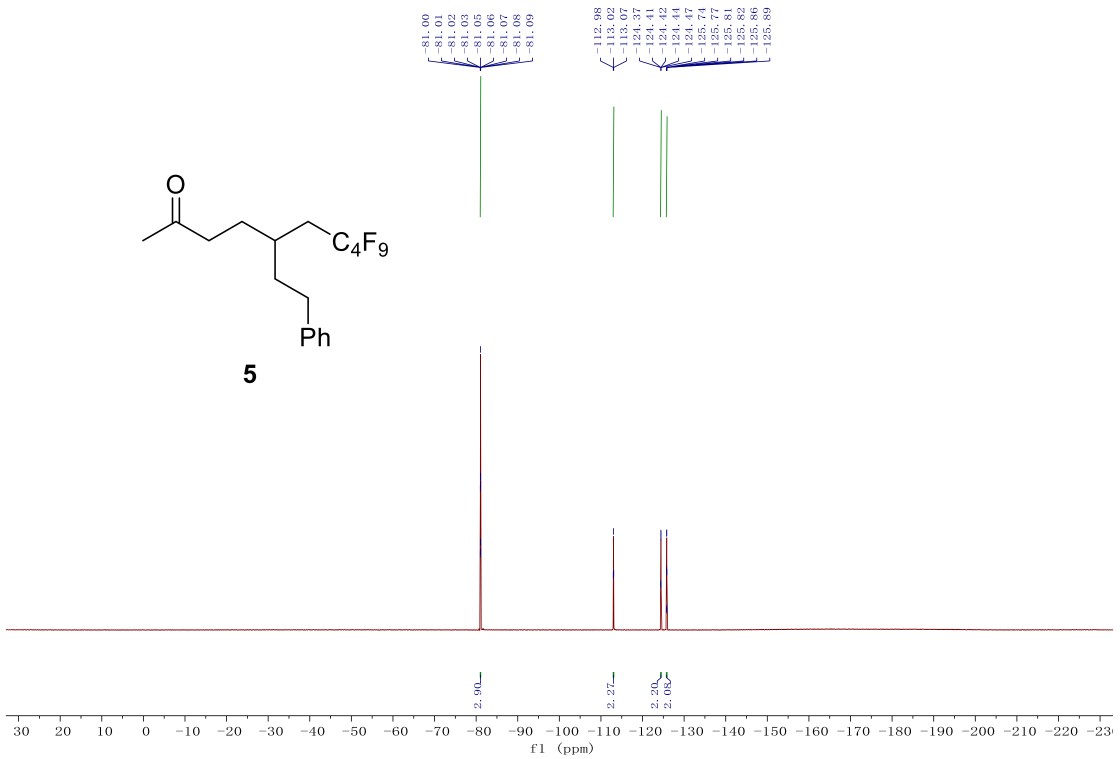

Jul14-2017.301.fid — TXJ-871-F — f19cpd CDCl3 /opt/topspin av1 44

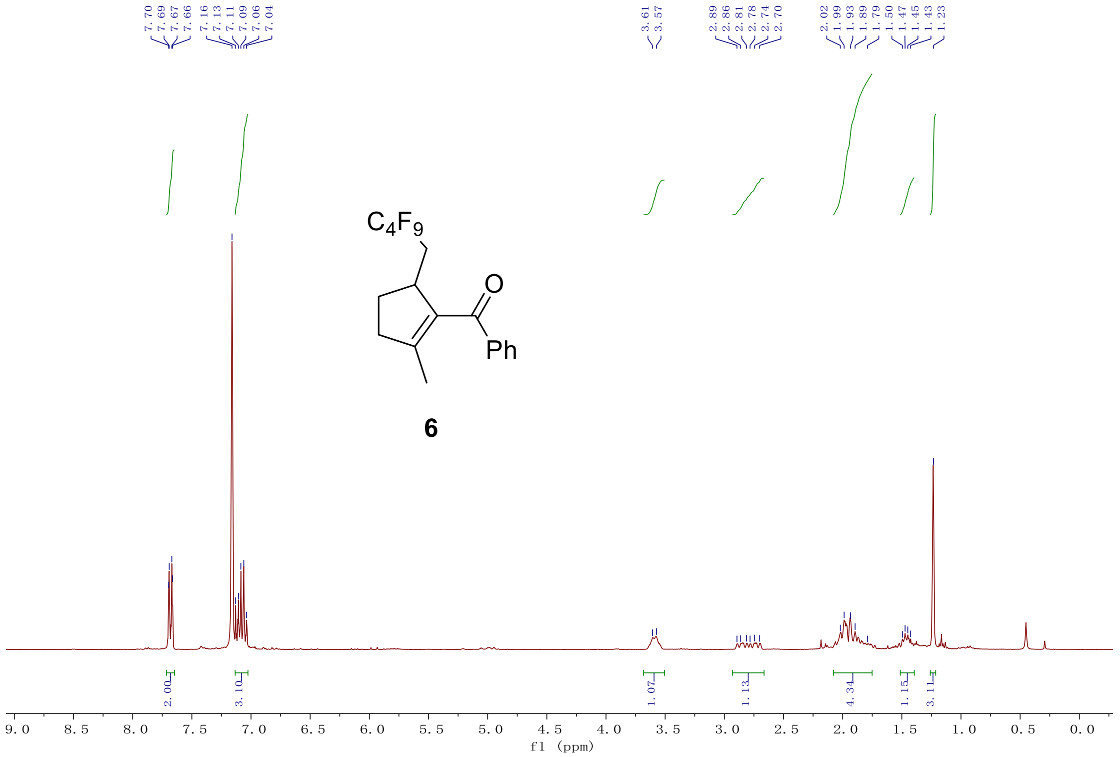

Jun22-2017.100.fid — TXJ-921-H — proton C6D6 /opt/topspin av1 31

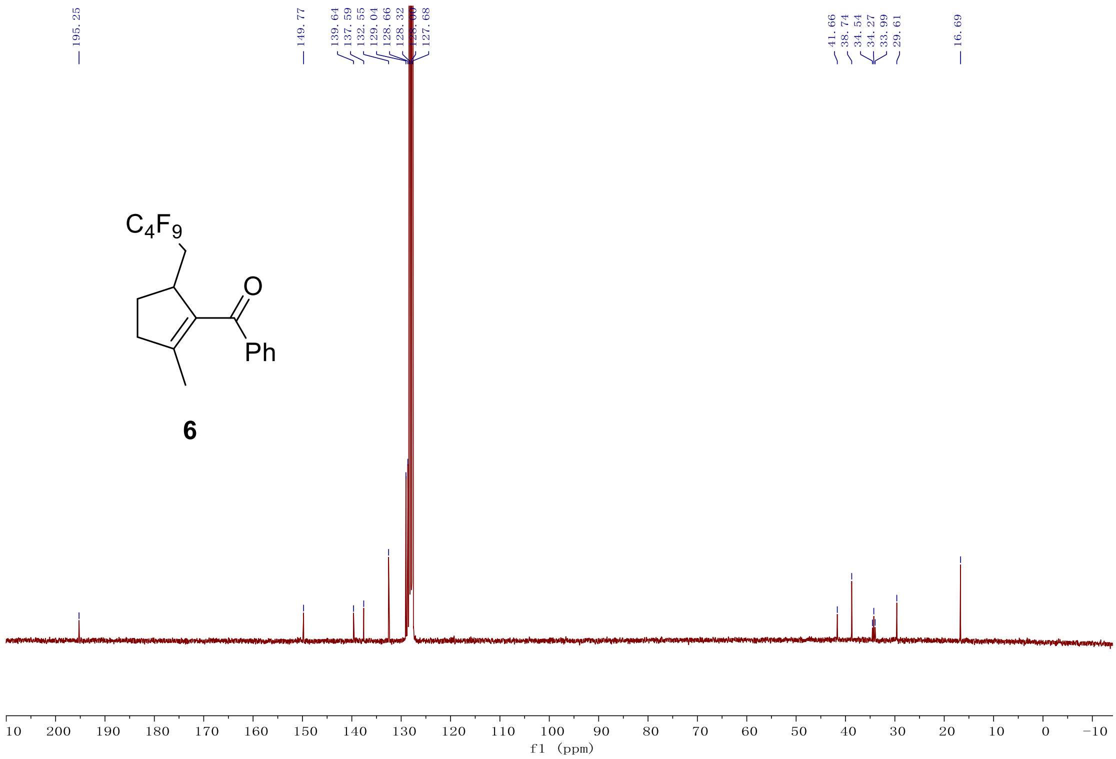

Jun22-2017.103.fid — TXJ-921-C — carbon C6D6 /opt/topspin av1 31

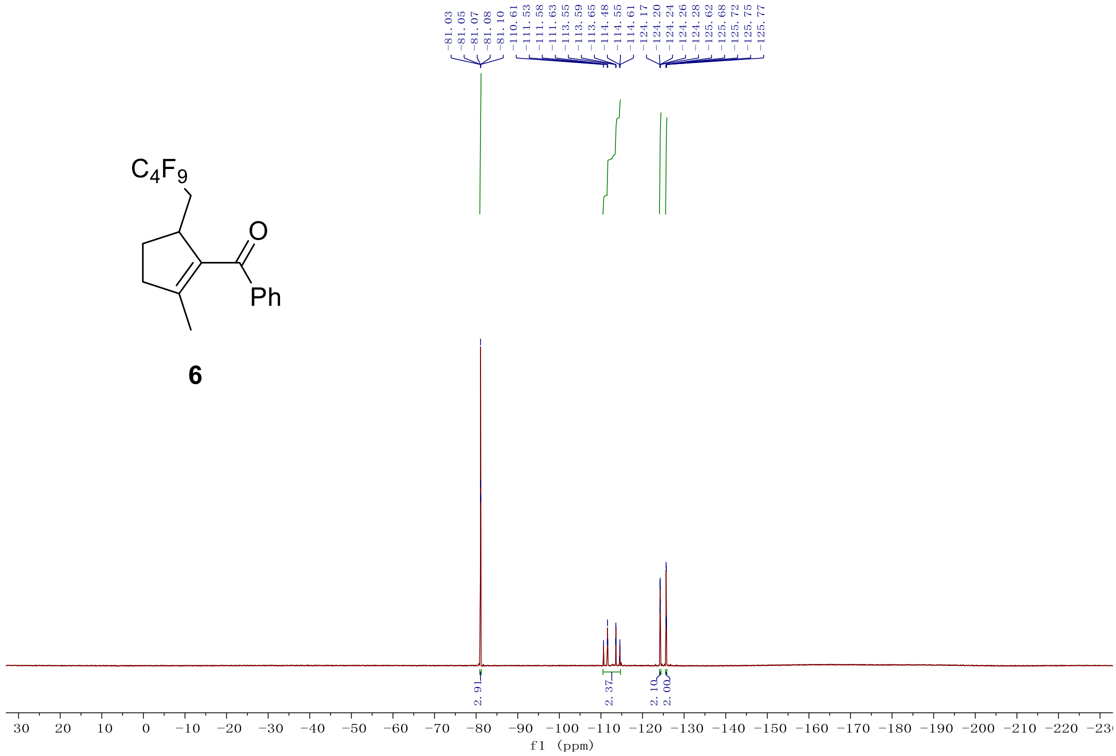

Jun22-2017.101.fid — TXJ-921-F — f19cpd C6D6 /opt/topspin av1 31

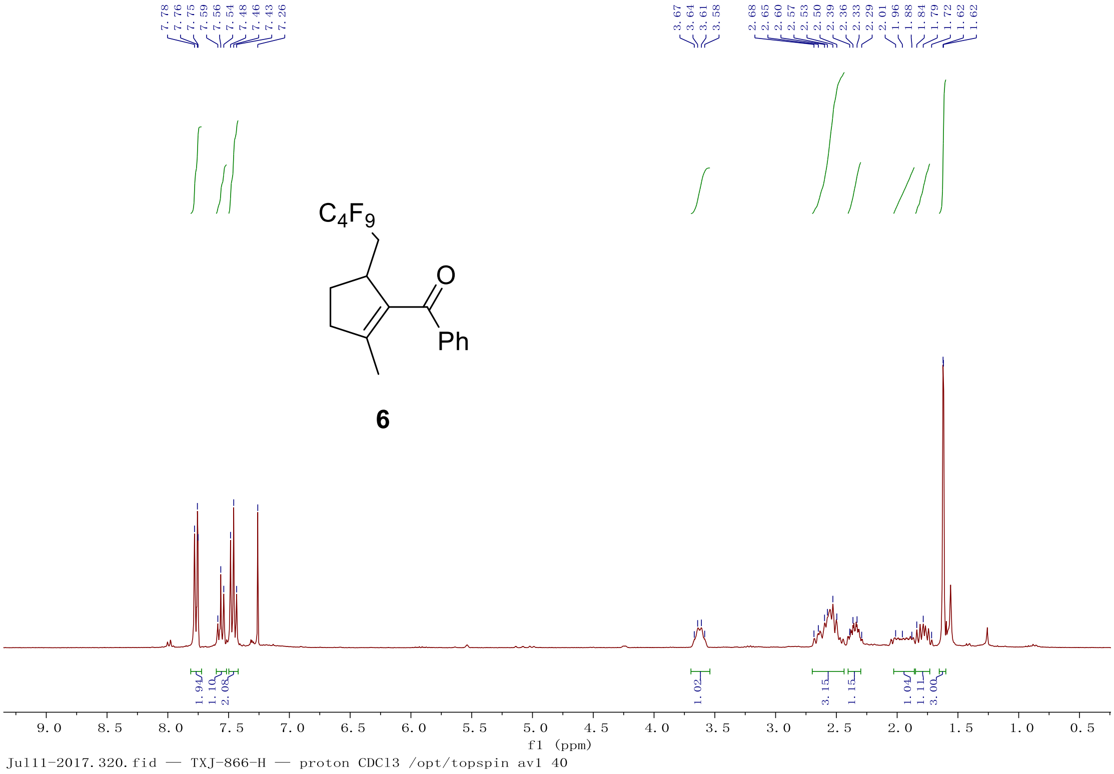
7.78 7.76 7.75 7.59 7.56 7.54 7.48 7.46 7.43 7.26
3.67 3.64 3.61 3.58
2.68 2.65 2.60 2.57 2.53 2.50 2.39 2.36 2.33 2.29 2.01 1.96 1.88 1.84 1.79 1.72 1.62 1.62
C4F9
O
Ph
6
1.94 1.10 2.08 1.02 3.15 1.15 1.04 1.11 3.00
9.0 8.5 8.0 7.5 7.0 6.5 6.0 5.5 5.0 4.5 4.0 3.5 3.0 2.5 2.0 1.5 1.0 0.5
f1 (ppm)
Jul11-2017.320.fid — TXJ-866-H — proton CDCl3 /opt/topspin av1 40

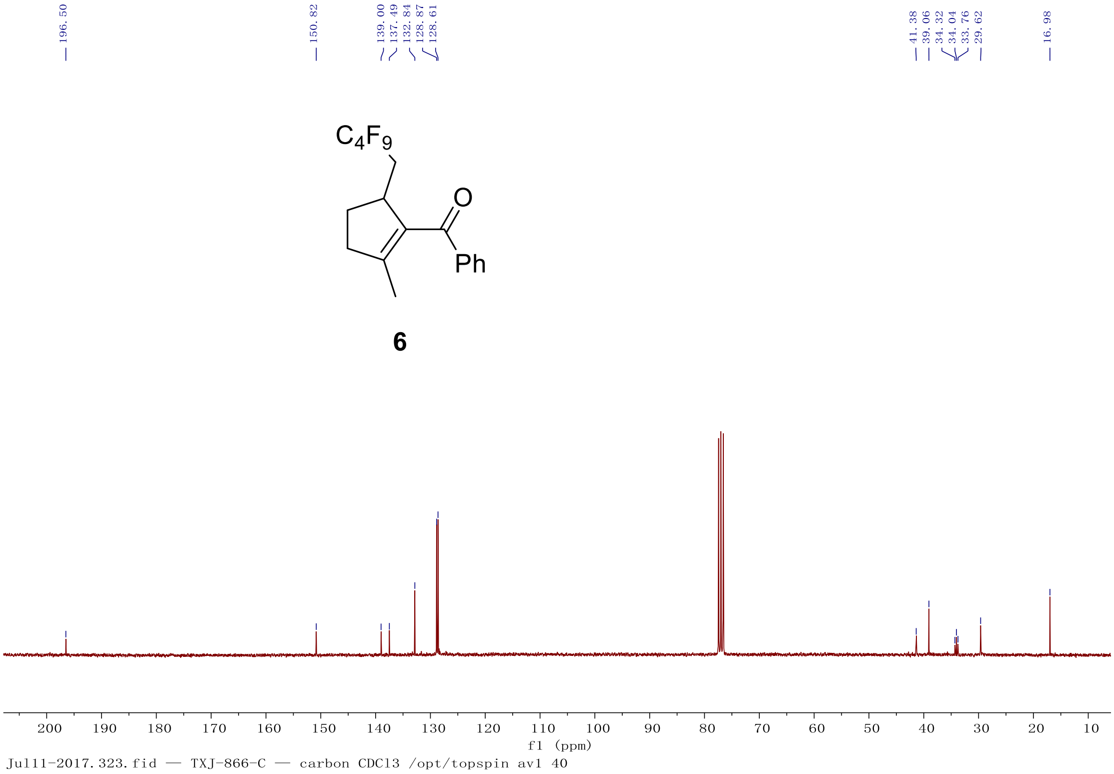
196.50
150.82
139.00 137.49 132.84 128.87 128.61
41.38 39.06 34.32 34.04 33.76 29.62
16.98
C4F9
O
Ph
6
200 190 180 170 160 150 140 130 120 110 100 90 80 70 60 50 40 30 20 10
f1 (ppm)
Jul11-2017.323.fid — TXJ-866-C — carbon CDCl3 /opt/topspin av1 40

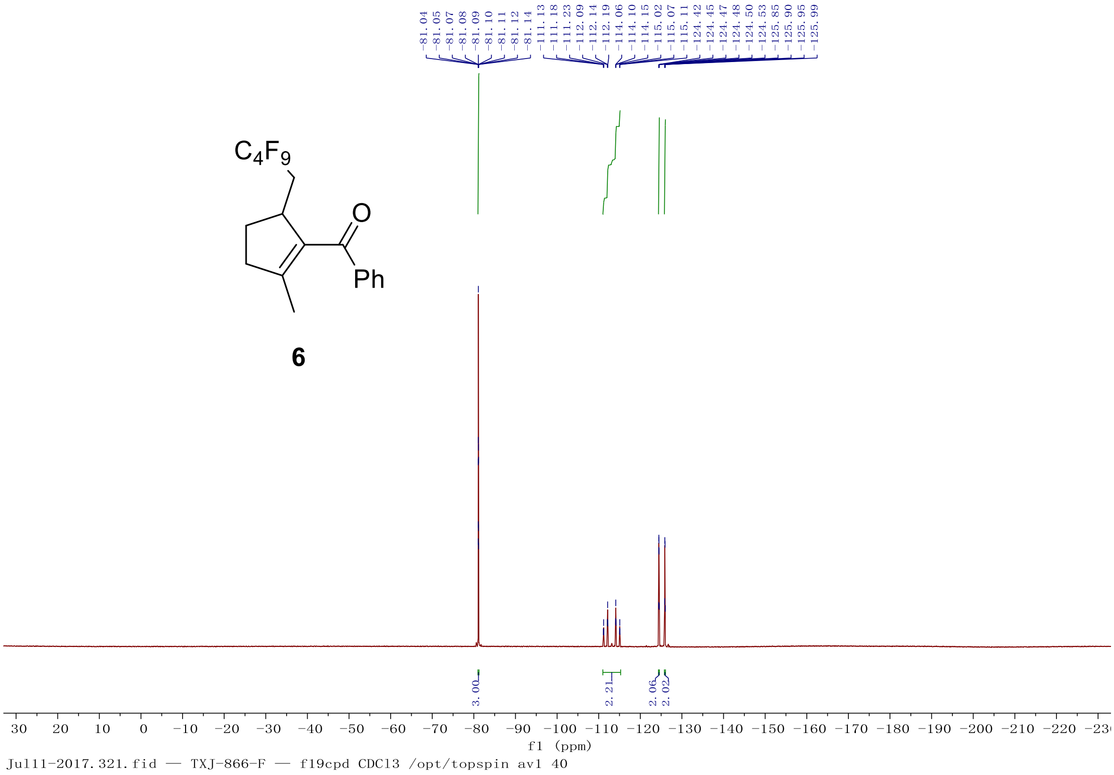

-81.04
-81.05
-81.07
-81.08
-81.09
-81.10
-81.11
-81.12
-81.14
-111.13
-111.18
-111.23
-112.09
-112.14
-112.19
-114.06
-114.10
-114.15
-115.02
-115.07
-115.11
-124.42
-124.45
-124.47
-124.48
-124.50
-124.53
-125.85
-125.90
-125.95
-125.99
C_4F_9
O
Ph
6
3.00
2.21
2.06
2.02
30 20 10 0 −10 −20 −30 −40 −50 −60 −70 −80 −90 −100 −110 −120 −130 −140 −150 −160 −170 −180 −190 −200 −210 −220
f1 (ppm)
Jul11-2017.321.fid — TXJ-866-F — f19cpd CDCl3 /opt/topspin av1 40
